# Supplementary material for: Global and Chinese epidemiologic study of polycystic ovary syndrome in women of childbearing age, 1990–2021, and projections to 2035: Based on the Global Burden of Disease 2021 study
Source: PLoS One. 2025 Aug 19;20(8):e0329090. doi: 10.1371/journal.pone.0329090 (PMC12364318; doi:10.1371/journal.pone.0329090)
Supplement: S1 Table — (DOCX) [file pone.0329090.s001.docx]

| **Supplementary Table 1** Global and Regional Incidence of Polycystic Ovarian Syndrome Among Women Aged 15-49 Years: A Comprehensive Analysis from 1990 to 2021 | | | | | | | | | |
| --- | --- | --- | --- | --- | --- | --- | --- | --- | --- |
| **Measure** | **Location** | **Sex** | **Age** | **Cause** | **Metric** | **Year** | **Value** | **Upper** | **Lower** |
| Incidence | Global | Female | 15-49 years | Polycystic ovarian syndrome | Rate | 1990 | 58.84372549 | 87.96440589 | 38.18307048 |
| Incidence | Global | Female | 15-49 years | Polycystic ovarian syndrome | Rate | 1991 | 58.27655824 | 87.03175354 | 37.86776723 |
| Incidence | Global | Female | 15-49 years | Polycystic ovarian syndrome | Rate | 1992 | 57.72156371 | 86.10191228 | 37.56256161 |
| Incidence | Global | Female | 15-49 years | Polycystic ovarian syndrome | Rate | 1993 | 57.22788398 | 85.28683012 | 37.28671733 |
| Incidence | Global | Female | 15-49 years | Polycystic ovarian syndrome | Rate | 1994 | 56.87398428 | 84.70220637 | 37.07347941 |
| Incidence | Global | Female | 15-49 years | Polycystic ovarian syndrome | Rate | 1995 | 56.67656808 | 84.36797802 | 36.96449367 |
| Incidence | Global | Female | 15-49 years | Polycystic ovarian syndrome | Rate | 1996 | 56.66853198 | 84.25255082 | 36.99663161 |
| Incidence | Global | Female | 15-49 years | Polycystic ovarian syndrome | Rate | 1997 | 56.89152208 | 84.49662542 | 37.17922119 |
| Incidence | Global | Female | 15-49 years | Polycystic ovarian syndrome | Rate | 1998 | 57.18715173 | 84.84336773 | 37.40868066 |
| Incidence | Global | Female | 15-49 years | Polycystic ovarian syndrome | Rate | 1999 | 57.52710946 | 85.25187398 | 37.66018005 |
| Incidence | Global | Female | 15-49 years | Polycystic ovarian syndrome | Rate | 2000 | 57.7610071 | 85.59414759 | 37.83124591 |
| Incidence | Global | Female | 15-49 years | Polycystic ovarian syndrome | Rate | 2001 | 58.16542958 | 86.18809707 | 38.15103021 |
| Incidence | Global | Female | 15-49 years | Polycystic ovarian syndrome | Rate | 2002 | 58.69121024 | 86.94173092 | 38.54226222 |
| Incidence | Global | Female | 15-49 years | Polycystic ovarian syndrome | Rate | 2003 | 59.18593185 | 87.64569401 | 38.89369541 |
| Incidence | Global | Female | 15-49 years | Polycystic ovarian syndrome | Rate | 2004 | 59.52506032 | 88.11229108 | 39.15326908 |
| Incidence | Global | Female | 15-49 years | Polycystic ovarian syndrome | Rate | 2005 | 59.58150201 | 88.13730383 | 39.22464593 |
| Incidence | Global | Female | 15-49 years | Polycystic ovarian syndrome | Rate | 2006 | 59.35628653 | 87.44861376 | 39.18425518 |
| Incidence | Global | Female | 15-49 years | Polycystic ovarian syndrome | Rate | 2007 | 58.99807282 | 86.53711712 | 39.01364706 |
| Incidence | Global | Female | 15-49 years | Polycystic ovarian syndrome | Rate | 2008 | 58.52924778 | 85.53195666 | 38.77016371 |
| Incidence | Global | Female | 15-49 years | Polycystic ovarian syndrome | Rate | 2009 | 58.00253077 | 84.53387705 | 38.472749 |
| Incidence | Global | Female | 15-49 years | Polycystic ovarian syndrome | Rate | 2010 | 57.45728145 | 83.58793708 | 38.13820691 |
| Incidence | Global | Female | 15-49 years | Polycystic ovarian syndrome | Rate | 2011 | 57.0426531 | 83.10701567 | 37.85817677 |
| Incidence | Global | Female | 15-49 years | Polycystic ovarian syndrome | Rate | 2012 | 56.82552424 | 82.99355319 | 37.69370956 |
| Incidence | Global | Female | 15-49 years | Polycystic ovarian syndrome | Rate | 2013 | 56.75279307 | 83.14675187 | 37.61233396 |
| Incidence | Global | Female | 15-49 years | Polycystic ovarian syndrome | Rate | 2014 | 56.74970503 | 83.41584076 | 37.57175826 |
| Incidence | Global | Female | 15-49 years | Polycystic ovarian syndrome | Rate | 2015 | 56.80176764 | 83.53386167 | 37.56819275 |
| Incidence | Global | Female | 15-49 years | Polycystic ovarian syndrome | Rate | 2016 | 57.03263802 | 83.89126663 | 37.6690287 |
| Incidence | Global | Female | 15-49 years | Polycystic ovarian syndrome | Rate | 2017 | 57.53822361 | 84.7281765 | 37.93194523 |
| Incidence | Global | Female | 15-49 years | Polycystic ovarian syndrome | Rate | 2018 | 58.20015789 | 85.69408289 | 38.29142537 |
| Incidence | Global | Female | 15-49 years | Polycystic ovarian syndrome | Rate | 2019 | 58.89951513 | 86.69279909 | 38.65531086 |
| Incidence | Global | Female | 15-49 years | Polycystic ovarian syndrome | Rate | 2020 | 59.82339356 | 88.26899928 | 39.1670188 |
| Incidence | Global | Female | 15-49 years | Polycystic ovarian syndrome | Rate | 2021 | 60.29562183 | 89.31281613 | 39.34850659 |
| Incidence | Eastern Europe | Female | 15-49 years | Polycystic ovarian syndrome | Rate | 1990 | 10.46150105 | 14.50781849 | 7.41615177 |
| Incidence | Eastern Europe | Female | 15-49 years | Polycystic ovarian syndrome | Rate | 1991 | 10.58552673 | 14.66316809 | 7.51233603 |
| Incidence | Eastern Europe | Female | 15-49 years | Polycystic ovarian syndrome | Rate | 1992 | 10.66715697 | 14.75872757 | 7.5631024 |
| Incidence | Eastern Europe | Female | 15-49 years | Polycystic ovarian syndrome | Rate | 1993 | 10.67498574 | 14.74276737 | 7.564501604 |
| Incidence | Eastern Europe | Female | 15-49 years | Polycystic ovarian syndrome | Rate | 1994 | 10.6533935 | 14.70297537 | 7.540316795 |
| Incidence | Eastern Europe | Female | 15-49 years | Polycystic ovarian syndrome | Rate | 1995 | 10.62252286 | 14.64751905 | 7.506799749 |
| Incidence | Eastern Europe | Female | 15-49 years | Polycystic ovarian syndrome | Rate | 1996 | 10.62876639 | 14.62907071 | 7.530427753 |
| Incidence | Eastern Europe | Female | 15-49 years | Polycystic ovarian syndrome | Rate | 1997 | 10.73563469 | 14.76830434 | 7.584788468 |
| Incidence | Eastern Europe | Female | 15-49 years | Polycystic ovarian syndrome | Rate | 1998 | 10.88456742 | 15.00060487 | 7.702514845 |
| Incidence | Eastern Europe | Female | 15-49 years | Polycystic ovarian syndrome | Rate | 1999 | 11.06908085 | 15.37336829 | 7.833517642 |
| Incidence | Eastern Europe | Female | 15-49 years | Polycystic ovarian syndrome | Rate | 2000 | 11.25329184 | 15.74653832 | 7.934177797 |
| Incidence | Eastern Europe | Female | 15-49 years | Polycystic ovarian syndrome | Rate | 2001 | 11.43761777 | 16.02151069 | 8.074930677 |
| Incidence | Eastern Europe | Female | 15-49 years | Polycystic ovarian syndrome | Rate | 2002 | 11.61145286 | 16.2710176 | 8.158078077 |
| Incidence | Eastern Europe | Female | 15-49 years | Polycystic ovarian syndrome | Rate | 2003 | 11.71946069 | 16.3912878 | 8.227840204 |
| Incidence | Eastern Europe | Female | 15-49 years | Polycystic ovarian syndrome | Rate | 2004 | 11.76710216 | 16.39303078 | 8.271738352 |
| Incidence | Eastern Europe | Female | 15-49 years | Polycystic ovarian syndrome | Rate | 2005 | 11.74797998 | 16.34145457 | 8.261634523 |
| Incidence | Eastern Europe | Female | 15-49 years | Polycystic ovarian syndrome | Rate | 2006 | 11.65240912 | 16.17247239 | 8.149395001 |
| Incidence | Eastern Europe | Female | 15-49 years | Polycystic ovarian syndrome | Rate | 2007 | 11.49490223 | 15.97557583 | 8.037748918 |
| Incidence | Eastern Europe | Female | 15-49 years | Polycystic ovarian syndrome | Rate | 2008 | 11.29706934 | 15.69703962 | 7.915120733 |
| Incidence | Eastern Europe | Female | 15-49 years | Polycystic ovarian syndrome | Rate | 2009 | 11.08510159 | 15.31177715 | 7.786844188 |
| Incidence | Eastern Europe | Female | 15-49 years | Polycystic ovarian syndrome | Rate | 2010 | 10.85893166 | 15.00813042 | 7.637856576 |
| Incidence | Eastern Europe | Female | 15-49 years | Polycystic ovarian syndrome | Rate | 2011 | 10.63309839 | 14.73116051 | 7.538248194 |
| Incidence | Eastern Europe | Female | 15-49 years | Polycystic ovarian syndrome | Rate | 2012 | 10.43030304 | 14.4036826 | 7.424389561 |
| Incidence | Eastern Europe | Female | 15-49 years | Polycystic ovarian syndrome | Rate | 2013 | 10.25848016 | 14.15564072 | 7.284806101 |
| Incidence | Eastern Europe | Female | 15-49 years | Polycystic ovarian syndrome | Rate | 2014 | 10.10730205 | 13.90252166 | 7.183540522 |
| Incidence | Eastern Europe | Female | 15-49 years | Polycystic ovarian syndrome | Rate | 2015 | 9.994746612 | 13.70620337 | 7.067636356 |
| Incidence | Eastern Europe | Female | 15-49 years | Polycystic ovarian syndrome | Rate | 2016 | 9.986296766 | 13.63736696 | 7.089005886 |
| Incidence | Eastern Europe | Female | 15-49 years | Polycystic ovarian syndrome | Rate | 2017 | 10.0908403 | 13.769934 | 7.178996914 |
| Incidence | Eastern Europe | Female | 15-49 years | Polycystic ovarian syndrome | Rate | 2018 | 10.24885365 | 14.03109113 | 7.325185281 |
| Incidence | Eastern Europe | Female | 15-49 years | Polycystic ovarian syndrome | Rate | 2019 | 10.39589341 | 14.27576675 | 7.435134651 |
| Incidence | Eastern Europe | Female | 15-49 years | Polycystic ovarian syndrome | Rate | 2020 | 10.45816852 | 14.4289617 | 7.496801602 |
| Incidence | Eastern Europe | Female | 15-49 years | Polycystic ovarian syndrome | Rate | 2021 | 10.37227138 | 14.27125177 | 7.423157461 |
| Incidence | Central Europe | Female | 15-49 years | Polycystic ovarian syndrome | Rate | 1990 | 7.340624745 | 10.88071702 | 4.861151392 |
| Incidence | Central Europe | Female | 15-49 years | Polycystic ovarian syndrome | Rate | 1991 | 7.463380305 | 11.04557637 | 4.935256089 |
| Incidence | Central Europe | Female | 15-49 years | Polycystic ovarian syndrome | Rate | 1992 | 7.573071431 | 11.20117923 | 5.0019647 |
| Incidence | Central Europe | Female | 15-49 years | Polycystic ovarian syndrome | Rate | 1993 | 7.65431269 | 11.28685655 | 5.049221521 |
| Incidence | Central Europe | Female | 15-49 years | Polycystic ovarian syndrome | Rate | 1994 | 7.708098135 | 11.33307335 | 5.077319823 |
| Incidence | Central Europe | Female | 15-49 years | Polycystic ovarian syndrome | Rate | 1995 | 7.724581873 | 11.31422786 | 5.089409794 |
| Incidence | Central Europe | Female | 15-49 years | Polycystic ovarian syndrome | Rate | 1996 | 7.735021323 | 11.32132135 | 5.087050311 |
| Incidence | Central Europe | Female | 15-49 years | Polycystic ovarian syndrome | Rate | 1997 | 7.769732889 | 11.36884075 | 5.101381428 |
| Incidence | Central Europe | Female | 15-49 years | Polycystic ovarian syndrome | Rate | 1998 | 7.808929817 | 11.39966285 | 5.123059862 |
| Incidence | Central Europe | Female | 15-49 years | Polycystic ovarian syndrome | Rate | 1999 | 7.84059485 | 11.42971265 | 5.154764384 |
| Incidence | Central Europe | Female | 15-49 years | Polycystic ovarian syndrome | Rate | 2000 | 7.864418304 | 11.45034299 | 5.182436718 |
| Incidence | Central Europe | Female | 15-49 years | Polycystic ovarian syndrome | Rate | 2001 | 7.873633941 | 11.44029299 | 5.170016911 |
| Incidence | Central Europe | Female | 15-49 years | Polycystic ovarian syndrome | Rate | 2002 | 7.876094199 | 11.41597959 | 5.155058001 |
| Incidence | Central Europe | Female | 15-49 years | Polycystic ovarian syndrome | Rate | 2003 | 7.8700167 | 11.38186736 | 5.147100894 |
| Incidence | Central Europe | Female | 15-49 years | Polycystic ovarian syndrome | Rate | 2004 | 7.841368173 | 11.31138664 | 5.13001368 |
| Incidence | Central Europe | Female | 15-49 years | Polycystic ovarian syndrome | Rate | 2005 | 7.796022396 | 11.20898036 | 5.103485183 |
| Incidence | Central Europe | Female | 15-49 years | Polycystic ovarian syndrome | Rate | 2006 | 7.713623529 | 11.03558774 | 5.06279548 |
| Incidence | Central Europe | Female | 15-49 years | Polycystic ovarian syndrome | Rate | 2007 | 7.577472914 | 10.78600719 | 4.979708223 |
| Incidence | Central Europe | Female | 15-49 years | Polycystic ovarian syndrome | Rate | 2008 | 7.392273235 | 10.42973608 | 4.8687436 |
| Incidence | Central Europe | Female | 15-49 years | Polycystic ovarian syndrome | Rate | 2009 | 7.182486664 | 10.01224535 | 4.763254477 |
| Incidence | Central Europe | Female | 15-49 years | Polycystic ovarian syndrome | Rate | 2010 | 6.963431346 | 9.660757636 | 4.648683908 |
| Incidence | Central Europe | Female | 15-49 years | Polycystic ovarian syndrome | Rate | 2011 | 6.745017873 | 9.382659292 | 4.549642498 |
| Incidence | Central Europe | Female | 15-49 years | Polycystic ovarian syndrome | Rate | 2012 | 6.518195105 | 9.103620624 | 4.441288402 |
| Incidence | Central Europe | Female | 15-49 years | Polycystic ovarian syndrome | Rate | 2013 | 6.309061928 | 8.816149283 | 4.339269481 |
| Incidence | Central Europe | Female | 15-49 years | Polycystic ovarian syndrome | Rate | 2014 | 6.139002774 | 8.581184592 | 4.266143816 |
| Incidence | Central Europe | Female | 15-49 years | Polycystic ovarian syndrome | Rate | 2015 | 6.030172319 | 8.448363035 | 4.217111267 |
| Incidence | Central Europe | Female | 15-49 years | Polycystic ovarian syndrome | Rate | 2016 | 5.967529329 | 8.342728301 | 4.175469971 |
| Incidence | Central Europe | Female | 15-49 years | Polycystic ovarian syndrome | Rate | 2017 | 5.930276999 | 8.256914524 | 4.142592718 |
| Incidence | Central Europe | Female | 15-49 years | Polycystic ovarian syndrome | Rate | 2018 | 5.928088184 | 8.20861736 | 4.131397115 |
| Incidence | Central Europe | Female | 15-49 years | Polycystic ovarian syndrome | Rate | 2019 | 5.945612279 | 8.191783461 | 4.136646797 |
| Incidence | Central Europe | Female | 15-49 years | Polycystic ovarian syndrome | Rate | 2020 | 6.049145487 | 8.350554082 | 4.228850624 |
| Incidence | Central Europe | Female | 15-49 years | Polycystic ovarian syndrome | Rate | 2021 | 6.224737203 | 8.747188234 | 4.245855356 |
| Incidence | East Asia | Female | 15-49 years | Polycystic ovarian syndrome | Rate | 1990 | 41.95001178 | 61.05304746 | 27.95494542 |
| Incidence | East Asia | Female | 15-49 years | Polycystic ovarian syndrome | Rate | 1991 | 40.46250968 | 58.56804087 | 27.02626147 |
| Incidence | East Asia | Female | 15-49 years | Polycystic ovarian syndrome | Rate | 1992 | 39.08291858 | 56.41928501 | 26.14556173 |
| Incidence | East Asia | Female | 15-49 years | Polycystic ovarian syndrome | Rate | 1993 | 37.9055719 | 54.54213562 | 25.34129154 |
| Incidence | East Asia | Female | 15-49 years | Polycystic ovarian syndrome | Rate | 1994 | 37.06553719 | 53.22791568 | 24.70947208 |
| Incidence | East Asia | Female | 15-49 years | Polycystic ovarian syndrome | Rate | 1995 | 36.6019871 | 52.58841566 | 24.36406474 |
| Incidence | East Asia | Female | 15-49 years | Polycystic ovarian syndrome | Rate | 1996 | 36.60870415 | 52.71394056 | 24.38884024 |
| Incidence | East Asia | Female | 15-49 years | Polycystic ovarian syndrome | Rate | 1997 | 37.12123115 | 53.57514173 | 24.74149814 |
| Incidence | East Asia | Female | 15-49 years | Polycystic ovarian syndrome | Rate | 1998 | 38.04312375 | 54.99184127 | 25.33837126 |
| Incidence | East Asia | Female | 15-49 years | Polycystic ovarian syndrome | Rate | 1999 | 39.55480688 | 57.4788597 | 26.24381928 |
| Incidence | East Asia | Female | 15-49 years | Polycystic ovarian syndrome | Rate | 2000 | 41.36779976 | 60.43924405 | 27.3281616 |
| Incidence | East Asia | Female | 15-49 years | Polycystic ovarian syndrome | Rate | 2001 | 43.78158481 | 64.13651365 | 28.85571245 |
| Incidence | East Asia | Female | 15-49 years | Polycystic ovarian syndrome | Rate | 2002 | 46.44477917 | 68.6992547 | 30.44180453 |
| Incidence | East Asia | Female | 15-49 years | Polycystic ovarian syndrome | Rate | 2003 | 48.89274584 | 72.70656329 | 31.88582883 |
| Incidence | East Asia | Female | 15-49 years | Polycystic ovarian syndrome | Rate | 2004 | 50.65251517 | 75.53978306 | 32.93600115 |
| Incidence | East Asia | Female | 15-49 years | Polycystic ovarian syndrome | Rate | 2005 | 51.35397252 | 76.75086382 | 33.35048045 |
| Incidence | East Asia | Female | 15-49 years | Polycystic ovarian syndrome | Rate | 2006 | 50.77521936 | 75.64481321 | 33.0479988 |
| Incidence | East Asia | Female | 15-49 years | Polycystic ovarian syndrome | Rate | 2007 | 49.22634594 | 73.16805111 | 32.15860846 |
| Incidence | East Asia | Female | 15-49 years | Polycystic ovarian syndrome | Rate | 2008 | 47.01032325 | 69.70333916 | 30.81415746 |
| Incidence | East Asia | Female | 15-49 years | Polycystic ovarian syndrome | Rate | 2009 | 44.48789063 | 65.62154091 | 29.26857577 |
| Incidence | East Asia | Female | 15-49 years | Polycystic ovarian syndrome | Rate | 2010 | 42.05174185 | 61.57455191 | 27.69944911 |
| Incidence | East Asia | Female | 15-49 years | Polycystic ovarian syndrome | Rate | 2011 | 40.08986843 | 58.42965814 | 26.41060457 |
| Incidence | East Asia | Female | 15-49 years | Polycystic ovarian syndrome | Rate | 2012 | 38.62494615 | 56.15983811 | 25.44192995 |
| Incidence | East Asia | Female | 15-49 years | Polycystic ovarian syndrome | Rate | 2013 | 37.5794549 | 54.60010355 | 24.77464313 |
| Incidence | East Asia | Female | 15-49 years | Polycystic ovarian syndrome | Rate | 2014 | 36.87100125 | 53.65731915 | 24.33153558 |
| Incidence | East Asia | Female | 15-49 years | Polycystic ovarian syndrome | Rate | 2015 | 36.49339932 | 53.27084244 | 24.10062838 |
| Incidence | East Asia | Female | 15-49 years | Polycystic ovarian syndrome | Rate | 2016 | 36.53607278 | 53.3329191 | 24.17168929 |
| Incidence | East Asia | Female | 15-49 years | Polycystic ovarian syndrome | Rate | 2017 | 37.01512749 | 54.06510814 | 24.51020274 |
| Incidence | East Asia | Female | 15-49 years | Polycystic ovarian syndrome | Rate | 2018 | 37.76116175 | 55.28072841 | 25.02306016 |
| Incidence | East Asia | Female | 15-49 years | Polycystic ovarian syndrome | Rate | 2019 | 38.63529225 | 56.77611815 | 25.57316051 |
| Incidence | East Asia | Female | 15-49 years | Polycystic ovarian syndrome | Rate | 2020 | 39.68378648 | 58.42139786 | 26.13295398 |
| Incidence | East Asia | Female | 15-49 years | Polycystic ovarian syndrome | Rate | 2021 | 40.95514063 | 60.65731647 | 26.79265353 |
| Incidence | Oceania | Female | 15-49 years | Polycystic ovarian syndrome | Rate | 1990 | 62.06435537 | 92.13231445 | 40.01292358 |
| Incidence | Oceania | Female | 15-49 years | Polycystic ovarian syndrome | Rate | 1991 | 63.45758043 | 94.6555586 | 40.9383893 |
| Incidence | Oceania | Female | 15-49 years | Polycystic ovarian syndrome | Rate | 1992 | 64.73551566 | 97.57810226 | 41.88475341 |
| Incidence | Oceania | Female | 15-49 years | Polycystic ovarian syndrome | Rate | 1993 | 65.82503737 | 99.76660662 | 42.52374667 |
| Incidence | Oceania | Female | 15-49 years | Polycystic ovarian syndrome | Rate | 1994 | 66.76429799 | 101.4388397 | 43.16127448 |
| Incidence | Oceania | Female | 15-49 years | Polycystic ovarian syndrome | Rate | 1995 | 67.55048861 | 103.3046976 | 43.74240274 |
| Incidence | Oceania | Female | 15-49 years | Polycystic ovarian syndrome | Rate | 1996 | 68.20467164 | 103.9253015 | 44.01412782 |
| Incidence | Oceania | Female | 15-49 years | Polycystic ovarian syndrome | Rate | 1997 | 68.94771797 | 104.7670751 | 44.5747268 |
| Incidence | Oceania | Female | 15-49 years | Polycystic ovarian syndrome | Rate | 1998 | 69.56828686 | 105.2658469 | 45.11883315 |
| Incidence | Oceania | Female | 15-49 years | Polycystic ovarian syndrome | Rate | 1999 | 70.05997337 | 105.3156078 | 45.97365082 |
| Incidence | Oceania | Female | 15-49 years | Polycystic ovarian syndrome | Rate | 2000 | 70.4841713 | 105.5503303 | 46.35255502 |
| Incidence | Oceania | Female | 15-49 years | Polycystic ovarian syndrome | Rate | 2001 | 71.33194823 | 107.115823 | 46.64077533 |
| Incidence | Oceania | Female | 15-49 years | Polycystic ovarian syndrome | Rate | 2002 | 72.42431917 | 109.1291879 | 47.15201186 |
| Incidence | Oceania | Female | 15-49 years | Polycystic ovarian syndrome | Rate | 2003 | 73.47748997 | 110.5674949 | 47.54754267 |
| Incidence | Oceania | Female | 15-49 years | Polycystic ovarian syndrome | Rate | 2004 | 74.22792953 | 111.6361169 | 48.14635933 |
| Incidence | Oceania | Female | 15-49 years | Polycystic ovarian syndrome | Rate | 2005 | 74.46477266 | 112.080644 | 48.42222077 |
| Incidence | Oceania | Female | 15-49 years | Polycystic ovarian syndrome | Rate | 2006 | 74.31100267 | 111.3136043 | 48.44157024 |
| Incidence | Oceania | Female | 15-49 years | Polycystic ovarian syndrome | Rate | 2007 | 74.00636226 | 110.9568672 | 48.12959863 |
| Incidence | Oceania | Female | 15-49 years | Polycystic ovarian syndrome | Rate | 2008 | 73.63439652 | 110.4637418 | 47.77878881 |
| Incidence | Oceania | Female | 15-49 years | Polycystic ovarian syndrome | Rate | 2009 | 73.23930199 | 110.7900761 | 47.42673103 |
| Incidence | Oceania | Female | 15-49 years | Polycystic ovarian syndrome | Rate | 2010 | 72.87255109 | 111.096842 | 47.14232791 |
| Incidence | Oceania | Female | 15-49 years | Polycystic ovarian syndrome | Rate | 2011 | 72.53969529 | 110.2422877 | 46.9293705 |
| Incidence | Oceania | Female | 15-49 years | Polycystic ovarian syndrome | Rate | 2012 | 72.26169461 | 109.3772986 | 46.96109411 |
| Incidence | Oceania | Female | 15-49 years | Polycystic ovarian syndrome | Rate | 2013 | 71.98460433 | 109.4573575 | 47.01816119 |
| Incidence | Oceania | Female | 15-49 years | Polycystic ovarian syndrome | Rate | 2014 | 71.67348909 | 108.8915798 | 46.95001283 |
| Incidence | Oceania | Female | 15-49 years | Polycystic ovarian syndrome | Rate | 2015 | 71.29322207 | 108.7221001 | 46.43063332 |
| Incidence | Oceania | Female | 15-49 years | Polycystic ovarian syndrome | Rate | 2016 | 71.07455296 | 108.1264469 | 46.16536565 |
| Incidence | Oceania | Female | 15-49 years | Polycystic ovarian syndrome | Rate | 2017 | 71.0202319 | 107.2577017 | 45.79512143 |
| Incidence | Oceania | Female | 15-49 years | Polycystic ovarian syndrome | Rate | 2018 | 70.88686116 | 106.8369856 | 45.44627058 |
| Incidence | Oceania | Female | 15-49 years | Polycystic ovarian syndrome | Rate | 2019 | 70.43280071 | 106.7375421 | 44.96770002 |
| Incidence | Oceania | Female | 15-49 years | Polycystic ovarian syndrome | Rate | 2020 | 68.91795342 | 106.0472563 | 44.88118105 |
| Incidence | Oceania | Female | 15-49 years | Polycystic ovarian syndrome | Rate | 2021 | 69.85511681 | 107.301301 | 45.63020314 |
| Incidence | Southeast Asia | Female | 15-49 years | Polycystic ovarian syndrome | Rate | 1990 | 83.26382813 | 127.80169 | 52.40939305 |
| Incidence | Southeast Asia | Female | 15-49 years | Polycystic ovarian syndrome | Rate | 1991 | 83.04370598 | 127.5910097 | 52.09833875 |
| Incidence | Southeast Asia | Female | 15-49 years | Polycystic ovarian syndrome | Rate | 1992 | 83.05592982 | 127.6116973 | 51.98808067 |
| Incidence | Southeast Asia | Female | 15-49 years | Polycystic ovarian syndrome | Rate | 1993 | 83.29988865 | 127.9011843 | 52.18100333 |
| Incidence | Southeast Asia | Female | 15-49 years | Polycystic ovarian syndrome | Rate | 1994 | 83.72658976 | 128.5490335 | 52.49295453 |
| Incidence | Southeast Asia | Female | 15-49 years | Polycystic ovarian syndrome | Rate | 1995 | 84.26529193 | 129.3678275 | 52.8811516 |
| Incidence | Southeast Asia | Female | 15-49 years | Polycystic ovarian syndrome | Rate | 1996 | 85.07128016 | 130.1546831 | 53.63837436 |
| Incidence | Southeast Asia | Female | 15-49 years | Polycystic ovarian syndrome | Rate | 1997 | 86.1173933 | 131.2776595 | 54.59698848 |
| Incidence | Southeast Asia | Female | 15-49 years | Polycystic ovarian syndrome | Rate | 1998 | 87.22139152 | 132.4556855 | 55.61091029 |
| Incidence | Southeast Asia | Female | 15-49 years | Polycystic ovarian syndrome | Rate | 1999 | 88.20612827 | 133.7999262 | 56.6825768 |
| Incidence | Southeast Asia | Female | 15-49 years | Polycystic ovarian syndrome | Rate | 2000 | 88.88316471 | 135.0545262 | 57.50802113 |
| Incidence | Southeast Asia | Female | 15-49 years | Polycystic ovarian syndrome | Rate | 2001 | 89.55829508 | 135.9213687 | 57.91500495 |
| Incidence | Southeast Asia | Female | 15-49 years | Polycystic ovarian syndrome | Rate | 2002 | 90.40943881 | 137.1424772 | 58.38828692 |
| Incidence | Southeast Asia | Female | 15-49 years | Polycystic ovarian syndrome | Rate | 2003 | 91.45163452 | 138.7499412 | 58.93623038 |
| Incidence | Southeast Asia | Female | 15-49 years | Polycystic ovarian syndrome | Rate | 2004 | 92.64463902 | 140.6811047 | 59.66301269 |
| Incidence | Southeast Asia | Female | 15-49 years | Polycystic ovarian syndrome | Rate | 2005 | 93.97894787 | 142.6264465 | 60.52719189 |
| Incidence | Southeast Asia | Female | 15-49 years | Polycystic ovarian syndrome | Rate | 2006 | 95.60643299 | 145.8537566 | 61.35414781 |
| Incidence | Southeast Asia | Female | 15-49 years | Polycystic ovarian syndrome | Rate | 2007 | 97.51122209 | 149.2699009 | 62.17947524 |
| Incidence | Southeast Asia | Female | 15-49 years | Polycystic ovarian syndrome | Rate | 2008 | 99.36032995 | 152.1241183 | 62.9572665 |
| Incidence | Southeast Asia | Female | 15-49 years | Polycystic ovarian syndrome | Rate | 2009 | 100.8730877 | 154.509041 | 63.53283225 |
| Incidence | Southeast Asia | Female | 15-49 years | Polycystic ovarian syndrome | Rate | 2010 | 101.8947758 | 156.6003269 | 63.93704325 |
| Incidence | Southeast Asia | Female | 15-49 years | Polycystic ovarian syndrome | Rate | 2011 | 102.645602 | 158.7990053 | 64.48643901 |
| Incidence | Southeast Asia | Female | 15-49 years | Polycystic ovarian syndrome | Rate | 2012 | 103.27605 | 160.3863492 | 64.82933271 |
| Incidence | Southeast Asia | Female | 15-49 years | Polycystic ovarian syndrome | Rate | 2013 | 103.7283828 | 161.6214403 | 65.09690918 |
| Incidence | Southeast Asia | Female | 15-49 years | Polycystic ovarian syndrome | Rate | 2014 | 104.0354566 | 162.1581708 | 65.20465192 |
| Incidence | Southeast Asia | Female | 15-49 years | Polycystic ovarian syndrome | Rate | 2015 | 104.216653 | 162.8504547 | 65.18666845 |
| Incidence | Southeast Asia | Female | 15-49 years | Polycystic ovarian syndrome | Rate | 2016 | 104.4030146 | 162.9569162 | 65.26337865 |
| Incidence | Southeast Asia | Female | 15-49 years | Polycystic ovarian syndrome | Rate | 2017 | 104.6417558 | 163.2441965 | 65.34475375 |
| Incidence | Southeast Asia | Female | 15-49 years | Polycystic ovarian syndrome | Rate | 2018 | 104.7737111 | 163.6006852 | 65.38386979 |
| Incidence | Southeast Asia | Female | 15-49 years | Polycystic ovarian syndrome | Rate | 2019 | 104.6556878 | 162.9367953 | 65.44741745 |
| Incidence | Southeast Asia | Female | 15-49 years | Polycystic ovarian syndrome | Rate | 2020 | 103.9948749 | 162.2464608 | 65.7113739 |
| Incidence | Southeast Asia | Female | 15-49 years | Polycystic ovarian syndrome | Rate | 2021 | 101.0339036 | 155.9918144 | 63.57626341 |
| Incidence | Central Asia | Female | 15-49 years | Polycystic ovarian syndrome | Rate | 1990 | 16.90042198 | 24.76431593 | 11.0468163 |
| Incidence | Central Asia | Female | 15-49 years | Polycystic ovarian syndrome | Rate | 1991 | 17.02849546 | 24.87564308 | 11.06617483 |
| Incidence | Central Asia | Female | 15-49 years | Polycystic ovarian syndrome | Rate | 1992 | 17.10948053 | 24.96428609 | 11.10278588 |
| Incidence | Central Asia | Female | 15-49 years | Polycystic ovarian syndrome | Rate | 1993 | 17.11291515 | 25.05837122 | 11.08051617 |
| Incidence | Central Asia | Female | 15-49 years | Polycystic ovarian syndrome | Rate | 1994 | 17.0708997 | 25.09547795 | 11.02794565 |
| Incidence | Central Asia | Female | 15-49 years | Polycystic ovarian syndrome | Rate | 1995 | 17.00367001 | 24.89674819 | 10.97871246 |
| Incidence | Central Asia | Female | 15-49 years | Polycystic ovarian syndrome | Rate | 1996 | 16.93485142 | 24.90673617 | 10.92859965 |
| Incidence | Central Asia | Female | 15-49 years | Polycystic ovarian syndrome | Rate | 1997 | 16.92616433 | 24.7759474 | 10.94894329 |
| Incidence | Central Asia | Female | 15-49 years | Polycystic ovarian syndrome | Rate | 1998 | 16.98038922 | 24.84070508 | 11.00516644 |
| Incidence | Central Asia | Female | 15-49 years | Polycystic ovarian syndrome | Rate | 1999 | 17.077514 | 25.15075678 | 11.06214782 |
| Incidence | Central Asia | Female | 15-49 years | Polycystic ovarian syndrome | Rate | 2000 | 17.25229781 | 25.55087682 | 11.18051944 |
| Incidence | Central Asia | Female | 15-49 years | Polycystic ovarian syndrome | Rate | 2001 | 17.52868343 | 25.99500248 | 11.36323711 |
| Incidence | Central Asia | Female | 15-49 years | Polycystic ovarian syndrome | Rate | 2002 | 17.84070198 | 26.40718201 | 11.57489637 |
| Incidence | Central Asia | Female | 15-49 years | Polycystic ovarian syndrome | Rate | 2003 | 18.13714382 | 26.78842564 | 11.77796178 |
| Incidence | Central Asia | Female | 15-49 years | Polycystic ovarian syndrome | Rate | 2004 | 18.36753293 | 27.13819575 | 11.91574643 |
| Incidence | Central Asia | Female | 15-49 years | Polycystic ovarian syndrome | Rate | 2005 | 18.50918457 | 27.35298261 | 11.98877967 |
| Incidence | Central Asia | Female | 15-49 years | Polycystic ovarian syndrome | Rate | 2006 | 18.5848471 | 27.23329959 | 12.05689078 |
| Incidence | Central Asia | Female | 15-49 years | Polycystic ovarian syndrome | Rate | 2007 | 18.60470742 | 27.14211574 | 12.04076694 |
| Incidence | Central Asia | Female | 15-49 years | Polycystic ovarian syndrome | Rate | 2008 | 18.54400772 | 26.98106446 | 12.02043942 |
| Incidence | Central Asia | Female | 15-49 years | Polycystic ovarian syndrome | Rate | 2009 | 18.41835679 | 26.63109463 | 11.93822614 |
| Incidence | Central Asia | Female | 15-49 years | Polycystic ovarian syndrome | Rate | 2010 | 18.21820667 | 26.36267705 | 11.84313475 |
| Incidence | Central Asia | Female | 15-49 years | Polycystic ovarian syndrome | Rate | 2011 | 17.96332495 | 25.95563587 | 11.66903012 |
| Incidence | Central Asia | Female | 15-49 years | Polycystic ovarian syndrome | Rate | 2012 | 17.70684852 | 25.60090609 | 11.52460334 |
| Incidence | Central Asia | Female | 15-49 years | Polycystic ovarian syndrome | Rate | 2013 | 17.45941668 | 25.28316194 | 11.35206531 |
| Incidence | Central Asia | Female | 15-49 years | Polycystic ovarian syndrome | Rate | 2014 | 17.20305222 | 24.89599493 | 11.23581348 |
| Incidence | Central Asia | Female | 15-49 years | Polycystic ovarian syndrome | Rate | 2015 | 16.91777326 | 24.52055428 | 11.03897318 |
| Incidence | Central Asia | Female | 15-49 years | Polycystic ovarian syndrome | Rate | 2016 | 16.65740267 | 24.04489608 | 10.91639117 |
| Incidence | Central Asia | Female | 15-49 years | Polycystic ovarian syndrome | Rate | 2017 | 16.46681495 | 23.69414891 | 10.8081586 |
| Incidence | Central Asia | Female | 15-49 years | Polycystic ovarian syndrome | Rate | 2018 | 16.37075595 | 23.46449341 | 10.76405164 |
| Incidence | Central Asia | Female | 15-49 years | Polycystic ovarian syndrome | Rate | 2019 | 16.36899392 | 23.48166308 | 10.80967896 |
| Incidence | Central Asia | Female | 15-49 years | Polycystic ovarian syndrome | Rate | 2020 | 16.47123933 | 23.89592306 | 10.8655693 |
| Incidence | Central Asia | Female | 15-49 years | Polycystic ovarian syndrome | Rate | 2021 | 16.42662299 | 23.74532693 | 10.74896693 |
| Incidence | Australasia | Female | 15-49 years | Polycystic ovarian syndrome | Rate | 1990 | 173.8664542 | 279.3046528 | 106.5734344 |
| Incidence | Australasia | Female | 15-49 years | Polycystic ovarian syndrome | Rate | 1991 | 169.5641382 | 274.3460199 | 104.5899403 |
| Incidence | Australasia | Female | 15-49 years | Polycystic ovarian syndrome | Rate | 1992 | 165.7274798 | 270.8225338 | 102.8217323 |
| Incidence | Australasia | Female | 15-49 years | Polycystic ovarian syndrome | Rate | 1993 | 162.8239836 | 269.3263986 | 100.7675658 |
| Incidence | Australasia | Female | 15-49 years | Polycystic ovarian syndrome | Rate | 1994 | 160.9765995 | 265.8569824 | 99.54296871 |
| Incidence | Australasia | Female | 15-49 years | Polycystic ovarian syndrome | Rate | 1995 | 159.7399924 | 262.8595297 | 98.87946732 |
| Incidence | Australasia | Female | 15-49 years | Polycystic ovarian syndrome | Rate | 1996 | 160.0069777 | 262.3602071 | 98.94149796 |
| Incidence | Australasia | Female | 15-49 years | Polycystic ovarian syndrome | Rate | 1997 | 160.9885497 | 263.4436938 | 99.58781286 |
| Incidence | Australasia | Female | 15-49 years | Polycystic ovarian syndrome | Rate | 1998 | 162.482378 | 262.5776947 | 100.4253331 |
| Incidence | Australasia | Female | 15-49 years | Polycystic ovarian syndrome | Rate | 1999 | 163.9660273 | 260.0557655 | 101.8776271 |
| Incidence | Australasia | Female | 15-49 years | Polycystic ovarian syndrome | Rate | 2000 | 165.5506783 | 263.54876 | 103.8190567 |
| Incidence | Australasia | Female | 15-49 years | Polycystic ovarian syndrome | Rate | 2001 | 165.7814738 | 254.5676545 | 106.4225119 |
| Incidence | Australasia | Female | 15-49 years | Polycystic ovarian syndrome | Rate | 2002 | 164.5612765 | 243.0999786 | 106.801148 |
| Incidence | Australasia | Female | 15-49 years | Polycystic ovarian syndrome | Rate | 2003 | 162.8314981 | 233.902104 | 108.7234206 |
| Incidence | Australasia | Female | 15-49 years | Polycystic ovarian syndrome | Rate | 2004 | 161.7153584 | 229.2196509 | 110.6072165 |
| Incidence | Australasia | Female | 15-49 years | Polycystic ovarian syndrome | Rate | 2005 | 161.7497458 | 230.0788165 | 110.6388214 |
| Incidence | Australasia | Female | 15-49 years | Polycystic ovarian syndrome | Rate | 2006 | 163.0274095 | 229.3434674 | 111.8551451 |
| Incidence | Australasia | Female | 15-49 years | Polycystic ovarian syndrome | Rate | 2007 | 164.7462125 | 230.3835546 | 112.5543906 |
| Incidence | Australasia | Female | 15-49 years | Polycystic ovarian syndrome | Rate | 2008 | 166.1884808 | 233.8826771 | 112.1302944 |
| Incidence | Australasia | Female | 15-49 years | Polycystic ovarian syndrome | Rate | 2009 | 166.8387836 | 232.7973317 | 111.8930302 |
| Incidence | Australasia | Female | 15-49 years | Polycystic ovarian syndrome | Rate | 2010 | 166.4776153 | 235.2515209 | 111.9382954 |
| Incidence | Australasia | Female | 15-49 years | Polycystic ovarian syndrome | Rate | 2011 | 165.4753808 | 236.6347935 | 110.0136387 |
| Incidence | Australasia | Female | 15-49 years | Polycystic ovarian syndrome | Rate | 2012 | 164.9250464 | 240.9216696 | 108.0768419 |
| Incidence | Australasia | Female | 15-49 years | Polycystic ovarian syndrome | Rate | 2013 | 164.2557716 | 244.952165 | 106.8684042 |
| Incidence | Australasia | Female | 15-49 years | Polycystic ovarian syndrome | Rate | 2014 | 163.5532476 | 252.0974217 | 103.936309 |
| Incidence | Australasia | Female | 15-49 years | Polycystic ovarian syndrome | Rate | 2015 | 162.5294929 | 261.619412 | 97.89001825 |
| Incidence | Australasia | Female | 15-49 years | Polycystic ovarian syndrome | Rate | 2016 | 161.6897642 | 261.7213153 | 96.65528748 |
| Incidence | Australasia | Female | 15-49 years | Polycystic ovarian syndrome | Rate | 2017 | 160.7302488 | 261.2940157 | 96.58835456 |
| Incidence | Australasia | Female | 15-49 years | Polycystic ovarian syndrome | Rate | 2018 | 160.1683946 | 257.8870516 | 97.4914684 |
| Incidence | Australasia | Female | 15-49 years | Polycystic ovarian syndrome | Rate | 2019 | 159.8625212 | 256.8360498 | 96.90922101 |
| Incidence | Australasia | Female | 15-49 years | Polycystic ovarian syndrome | Rate | 2020 | 160.7734231 | 258.0901949 | 98.04713097 |
| Incidence | Australasia | Female | 15-49 years | Polycystic ovarian syndrome | Rate | 2021 | 160.4996095 | 261.57463 | 96.32048106 |
| Incidence | High-income Asia Pacific | Female | 15-49 years | Polycystic ovarian syndrome | Rate | 1990 | 238.4342853 | 392.1988042 | 131.0965223 |
| Incidence | High-income Asia Pacific | Female | 15-49 years | Polycystic ovarian syndrome | Rate | 1991 | 234.5398056 | 386.6565014 | 129.5335551 |
| Incidence | High-income Asia Pacific | Female | 15-49 years | Polycystic ovarian syndrome | Rate | 1992 | 228.7938537 | 376.8579012 | 126.8502446 |
| Incidence | High-income Asia Pacific | Female | 15-49 years | Polycystic ovarian syndrome | Rate | 1993 | 221.4542647 | 364.570507 | 123.1972312 |
| Incidence | High-income Asia Pacific | Female | 15-49 years | Polycystic ovarian syndrome | Rate | 1994 | 214.499118 | 353.3635065 | 119.7269942 |
| Incidence | High-income Asia Pacific | Female | 15-49 years | Polycystic ovarian syndrome | Rate | 1995 | 208.5782528 | 342.7162615 | 116.9855806 |
| Incidence | High-income Asia Pacific | Female | 15-49 years | Polycystic ovarian syndrome | Rate | 1996 | 203.9256558 | 334.4056507 | 114.7906401 |
| Incidence | High-income Asia Pacific | Female | 15-49 years | Polycystic ovarian syndrome | Rate | 1997 | 202.8045861 | 332.1677933 | 114.1486081 |
| Incidence | High-income Asia Pacific | Female | 15-49 years | Polycystic ovarian syndrome | Rate | 1998 | 202.5796887 | 331.3439601 | 113.7580857 |
| Incidence | High-income Asia Pacific | Female | 15-49 years | Polycystic ovarian syndrome | Rate | 1999 | 202.0382038 | 330.1793619 | 113.2736131 |
| Incidence | High-income Asia Pacific | Female | 15-49 years | Polycystic ovarian syndrome | Rate | 2000 | 199.640178 | 325.8292255 | 111.9419441 |
| Incidence | High-income Asia Pacific | Female | 15-49 years | Polycystic ovarian syndrome | Rate | 2001 | 197.8098232 | 319.7984335 | 111.5085833 |
| Incidence | High-income Asia Pacific | Female | 15-49 years | Polycystic ovarian syndrome | Rate | 2002 | 197.9730818 | 315.9958443 | 113.0297039 |
| Incidence | High-income Asia Pacific | Female | 15-49 years | Polycystic ovarian syndrome | Rate | 2003 | 198.1125755 | 311.3827701 | 115.0031206 |
| Incidence | High-income Asia Pacific | Female | 15-49 years | Polycystic ovarian syndrome | Rate | 2004 | 197.4345243 | 306.2976851 | 115.4729294 |
| Incidence | High-income Asia Pacific | Female | 15-49 years | Polycystic ovarian syndrome | Rate | 2005 | 195.7525489 | 301.0586998 | 114.9473985 |
| Incidence | High-income Asia Pacific | Female | 15-49 years | Polycystic ovarian syndrome | Rate | 2006 | 193.2913709 | 295.3864712 | 113.9463747 |
| Incidence | High-income Asia Pacific | Female | 15-49 years | Polycystic ovarian syndrome | Rate | 2007 | 191.1264913 | 290.4475657 | 113.1560437 |
| Incidence | High-income Asia Pacific | Female | 15-49 years | Polycystic ovarian syndrome | Rate | 2008 | 189.5321501 | 285.8953298 | 112.425231 |
| Incidence | High-income Asia Pacific | Female | 15-49 years | Polycystic ovarian syndrome | Rate | 2009 | 189.2101175 | 284.4200563 | 112.4923053 |
| Incidence | High-income Asia Pacific | Female | 15-49 years | Polycystic ovarian syndrome | Rate | 2010 | 189.5681567 | 285.0803291 | 113.0715457 |
| Incidence | High-income Asia Pacific | Female | 15-49 years | Polycystic ovarian syndrome | Rate | 2011 | 191.0848935 | 288.8873828 | 113.5801899 |
| Incidence | High-income Asia Pacific | Female | 15-49 years | Polycystic ovarian syndrome | Rate | 2012 | 194.0593086 | 295.1710837 | 114.2240824 |
| Incidence | High-income Asia Pacific | Female | 15-49 years | Polycystic ovarian syndrome | Rate | 2013 | 198.082488 | 303.6424762 | 115.2327732 |
| Incidence | High-income Asia Pacific | Female | 15-49 years | Polycystic ovarian syndrome | Rate | 2014 | 200.6342274 | 310.184985 | 115.8689635 |
| Incidence | High-income Asia Pacific | Female | 15-49 years | Polycystic ovarian syndrome | Rate | 2015 | 201.7828363 | 312.5572248 | 116.0994517 |
| Incidence | High-income Asia Pacific | Female | 15-49 years | Polycystic ovarian syndrome | Rate | 2016 | 200.0624387 | 310.4020423 | 115.0667436 |
| Incidence | High-income Asia Pacific | Female | 15-49 years | Polycystic ovarian syndrome | Rate | 2017 | 197.8462885 | 308.2067048 | 113.2568838 |
| Incidence | High-income Asia Pacific | Female | 15-49 years | Polycystic ovarian syndrome | Rate | 2018 | 195.2652229 | 306.5721026 | 111.8699518 |
| Incidence | High-income Asia Pacific | Female | 15-49 years | Polycystic ovarian syndrome | Rate | 2019 | 193.5644789 | 305.285573 | 110.7452053 |
| Incidence | High-income Asia Pacific | Female | 15-49 years | Polycystic ovarian syndrome | Rate | 2020 | 194.4914882 | 304.9225536 | 110.2404407 |
| Incidence | High-income Asia Pacific | Female | 15-49 years | Polycystic ovarian syndrome | Rate | 2021 | 197.2362262 | 306.0081708 | 112.7638671 |
| Incidence | Caribbean | Female | 15-49 years | Polycystic ovarian syndrome | Rate | 1990 | 46.59824332 | 69.4366702 | 30.85296331 |
| Incidence | Caribbean | Female | 15-49 years | Polycystic ovarian syndrome | Rate | 1991 | 45.82282401 | 68.31218412 | 30.39034234 |
| Incidence | Caribbean | Female | 15-49 years | Polycystic ovarian syndrome | Rate | 1992 | 44.97866137 | 67.0466174 | 29.87676506 |
| Incidence | Caribbean | Female | 15-49 years | Polycystic ovarian syndrome | Rate | 1993 | 44.18511183 | 65.64426782 | 29.38887809 |
| Incidence | Caribbean | Female | 15-49 years | Polycystic ovarian syndrome | Rate | 1994 | 43.51625933 | 64.4197954 | 28.97690903 |
| Incidence | Caribbean | Female | 15-49 years | Polycystic ovarian syndrome | Rate | 1995 | 43.00423729 | 63.58875029 | 28.6636756 |
| Incidence | Caribbean | Female | 15-49 years | Polycystic ovarian syndrome | Rate | 1996 | 42.7121842 | 62.92068689 | 28.51200143 |
| Incidence | Caribbean | Female | 15-49 years | Polycystic ovarian syndrome | Rate | 1997 | 42.69676042 | 62.68864601 | 28.47180112 |
| Incidence | Caribbean | Female | 15-49 years | Polycystic ovarian syndrome | Rate | 1998 | 42.93888922 | 62.90595648 | 28.59899173 |
| Incidence | Caribbean | Female | 15-49 years | Polycystic ovarian syndrome | Rate | 1999 | 43.35279155 | 63.27060536 | 28.89960385 |
| Incidence | Caribbean | Female | 15-49 years | Polycystic ovarian syndrome | Rate | 2000 | 43.83871272 | 63.86908175 | 29.27818114 |
| Incidence | Caribbean | Female | 15-49 years | Polycystic ovarian syndrome | Rate | 2001 | 44.4107996 | 64.76967339 | 29.55436755 |
| Incidence | Caribbean | Female | 15-49 years | Polycystic ovarian syndrome | Rate | 2002 | 45.042837 | 65.95860922 | 30.04215168 |
| Incidence | Caribbean | Female | 15-49 years | Polycystic ovarian syndrome | Rate | 2003 | 45.70946571 | 67.3070406 | 30.48719018 |
| Incidence | Caribbean | Female | 15-49 years | Polycystic ovarian syndrome | Rate | 2004 | 46.33939668 | 68.7235213 | 30.81728414 |
| Incidence | Caribbean | Female | 15-49 years | Polycystic ovarian syndrome | Rate | 2005 | 46.8172646 | 69.72391816 | 30.90002201 |
| Incidence | Caribbean | Female | 15-49 years | Polycystic ovarian syndrome | Rate | 2006 | 47.13310085 | 70.32850237 | 31.08845552 |
| Incidence | Caribbean | Female | 15-49 years | Polycystic ovarian syndrome | Rate | 2007 | 47.28459639 | 70.59840096 | 31.20325039 |
| Incidence | Caribbean | Female | 15-49 years | Polycystic ovarian syndrome | Rate | 2008 | 47.21802244 | 70.44786469 | 31.20791789 |
| Incidence | Caribbean | Female | 15-49 years | Polycystic ovarian syndrome | Rate | 2009 | 46.96382258 | 69.83007517 | 31.12545543 |
| Incidence | Caribbean | Female | 15-49 years | Polycystic ovarian syndrome | Rate | 2010 | 46.57471987 | 68.98855719 | 30.95285674 |
| Incidence | Caribbean | Female | 15-49 years | Polycystic ovarian syndrome | Rate | 2011 | 46.17727243 | 68.19191531 | 30.73121216 |
| Incidence | Caribbean | Female | 15-49 years | Polycystic ovarian syndrome | Rate | 2012 | 45.89368527 | 67.57578226 | 30.57017295 |
| Incidence | Caribbean | Female | 15-49 years | Polycystic ovarian syndrome | Rate | 2013 | 45.72152339 | 67.16283968 | 30.40760818 |
| Incidence | Caribbean | Female | 15-49 years | Polycystic ovarian syndrome | Rate | 2014 | 45.63184244 | 66.99620473 | 30.30141924 |
| Incidence | Caribbean | Female | 15-49 years | Polycystic ovarian syndrome | Rate | 2015 | 45.5894473 | 66.92282716 | 30.21569318 |
| Incidence | Caribbean | Female | 15-49 years | Polycystic ovarian syndrome | Rate | 2016 | 45.62106882 | 67.1343841 | 30.24688942 |
| Incidence | Caribbean | Female | 15-49 years | Polycystic ovarian syndrome | Rate | 2017 | 45.67337803 | 67.3142047 | 30.53139018 |
| Incidence | Caribbean | Female | 15-49 years | Polycystic ovarian syndrome | Rate | 2018 | 45.65267339 | 67.27893432 | 30.47244446 |
| Incidence | Caribbean | Female | 15-49 years | Polycystic ovarian syndrome | Rate | 2019 | 45.49157103 | 67.0258407 | 30.37839388 |
| Incidence | Caribbean | Female | 15-49 years | Polycystic ovarian syndrome | Rate | 2020 | 45.25348253 | 67.59882421 | 29.86870597 |
| Incidence | Caribbean | Female | 15-49 years | Polycystic ovarian syndrome | Rate | 2021 | 44.6705273 | 66.10962853 | 30.09286381 |
| Incidence | Western Europe | Female | 15-49 years | Polycystic ovarian syndrome | Rate | 1990 | 93.6257308 | 141.6961676 | 63.99775038 |
| Incidence | Western Europe | Female | 15-49 years | Polycystic ovarian syndrome | Rate | 1991 | 92.2692306 | 140.1269818 | 63.09559035 |
| Incidence | Western Europe | Female | 15-49 years | Polycystic ovarian syndrome | Rate | 1992 | 90.61879103 | 137.4059894 | 62.10009895 |
| Incidence | Western Europe | Female | 15-49 years | Polycystic ovarian syndrome | Rate | 1993 | 89.20975188 | 135.0402308 | 61.23872425 |
| Incidence | Western Europe | Female | 15-49 years | Polycystic ovarian syndrome | Rate | 1994 | 88.17440625 | 133.258599 | 60.50391847 |
| Incidence | Western Europe | Female | 15-49 years | Polycystic ovarian syndrome | Rate | 1995 | 87.33942656 | 131.8115209 | 59.94533761 |
| Incidence | Western Europe | Female | 15-49 years | Polycystic ovarian syndrome | Rate | 1996 | 86.8168838 | 131.1272973 | 59.71384909 |
| Incidence | Western Europe | Female | 15-49 years | Polycystic ovarian syndrome | Rate | 1997 | 86.73895881 | 130.8724982 | 59.8221566 |
| Incidence | Western Europe | Female | 15-49 years | Polycystic ovarian syndrome | Rate | 1998 | 86.75489174 | 130.4941686 | 59.98708841 |
| Incidence | Western Europe | Female | 15-49 years | Polycystic ovarian syndrome | Rate | 1999 | 86.52044295 | 129.6428995 | 59.80327604 |
| Incidence | Western Europe | Female | 15-49 years | Polycystic ovarian syndrome | Rate | 2000 | 86.13457721 | 128.6480009 | 59.45031757 |
| Incidence | Western Europe | Female | 15-49 years | Polycystic ovarian syndrome | Rate | 2001 | 85.80192822 | 128.0348207 | 58.97518132 |
| Incidence | Western Europe | Female | 15-49 years | Polycystic ovarian syndrome | Rate | 2002 | 85.66625989 | 127.9947222 | 58.57893813 |
| Incidence | Western Europe | Female | 15-49 years | Polycystic ovarian syndrome | Rate | 2003 | 85.76191815 | 128.3338714 | 58.54088125 |
| Incidence | Western Europe | Female | 15-49 years | Polycystic ovarian syndrome | Rate | 2004 | 86.03294137 | 128.8466301 | 58.53797809 |
| Incidence | Western Europe | Female | 15-49 years | Polycystic ovarian syndrome | Rate | 2005 | 86.22480876 | 129.3657267 | 58.55153284 |
| Incidence | Western Europe | Female | 15-49 years | Polycystic ovarian syndrome | Rate | 2006 | 86.13559917 | 129.1848674 | 58.66560518 |
| Incidence | Western Europe | Female | 15-49 years | Polycystic ovarian syndrome | Rate | 2007 | 85.87271609 | 128.6883854 | 58.47645046 |
| Incidence | Western Europe | Female | 15-49 years | Polycystic ovarian syndrome | Rate | 2008 | 85.39518015 | 127.7980372 | 58.11904334 |
| Incidence | Western Europe | Female | 15-49 years | Polycystic ovarian syndrome | Rate | 2009 | 84.63798369 | 126.5941935 | 57.48086067 |
| Incidence | Western Europe | Female | 15-49 years | Polycystic ovarian syndrome | Rate | 2010 | 83.83434858 | 125.2714893 | 56.89764998 |
| Incidence | Western Europe | Female | 15-49 years | Polycystic ovarian syndrome | Rate | 2011 | 83.32944487 | 124.6224431 | 56.63735479 |
| Incidence | Western Europe | Female | 15-49 years | Polycystic ovarian syndrome | Rate | 2012 | 83.17807123 | 124.846035 | 56.60239851 |
| Incidence | Western Europe | Female | 15-49 years | Polycystic ovarian syndrome | Rate | 2013 | 83.18380108 | 125.6031052 | 56.74443883 |
| Incidence | Western Europe | Female | 15-49 years | Polycystic ovarian syndrome | Rate | 2014 | 83.5827716 | 126.2372011 | 57.17090497 |
| Incidence | Western Europe | Female | 15-49 years | Polycystic ovarian syndrome | Rate | 2015 | 84.36730945 | 127.3726225 | 57.5646001 |
| Incidence | Western Europe | Female | 15-49 years | Polycystic ovarian syndrome | Rate | 2016 | 85.39158264 | 129.1140747 | 58.0442668 |
| Incidence | Western Europe | Female | 15-49 years | Polycystic ovarian syndrome | Rate | 2017 | 86.4835634 | 131.0995488 | 58.71460561 |
| Incidence | Western Europe | Female | 15-49 years | Polycystic ovarian syndrome | Rate | 2018 | 87.61683199 | 133.0519474 | 59.33542462 |
| Incidence | Western Europe | Female | 15-49 years | Polycystic ovarian syndrome | Rate | 2019 | 88.62787083 | 134.7317735 | 59.56906645 |
| Incidence | Western Europe | Female | 15-49 years | Polycystic ovarian syndrome | Rate | 2020 | 88.99719066 | 136.3614595 | 60.36954057 |
| Incidence | Western Europe | Female | 15-49 years | Polycystic ovarian syndrome | Rate | 2021 | 90.61605592 | 137.7576093 | 60.99156263 |
| Incidence | High-income North America | Female | 15-49 years | Polycystic ovarian syndrome | Rate | 1990 | 98.41489642 | 164.9411888 | 59.06525931 |
| Incidence | High-income North America | Female | 15-49 years | Polycystic ovarian syndrome | Rate | 1991 | 99.00968067 | 164.7107702 | 59.2618491 |
| Incidence | High-income North America | Female | 15-49 years | Polycystic ovarian syndrome | Rate | 1992 | 100.6654224 | 166.2365412 | 60.2016164 |
| Incidence | High-income North America | Female | 15-49 years | Polycystic ovarian syndrome | Rate | 1993 | 103.257669 | 169.6975399 | 61.91591138 |
| Incidence | High-income North America | Female | 15-49 years | Polycystic ovarian syndrome | Rate | 1994 | 105.9231167 | 174.3024369 | 63.48863962 |
| Incidence | High-income North America | Female | 15-49 years | Polycystic ovarian syndrome | Rate | 1995 | 108.1528206 | 178.6002442 | 64.41137036 |
| Incidence | High-income North America | Female | 15-49 years | Polycystic ovarian syndrome | Rate | 1996 | 109.4155861 | 181.0340211 | 65.62806271 |
| Incidence | High-income North America | Female | 15-49 years | Polycystic ovarian syndrome | Rate | 1997 | 109.8875149 | 181.8843561 | 66.16200795 |
| Incidence | High-income North America | Female | 15-49 years | Polycystic ovarian syndrome | Rate | 1998 | 109.3659723 | 181.1321202 | 66.14389295 |
| Incidence | High-income North America | Female | 15-49 years | Polycystic ovarian syndrome | Rate | 1999 | 108.0071599 | 179.2664315 | 65.51672554 |
| Incidence | High-income North America | Female | 15-49 years | Polycystic ovarian syndrome | Rate | 2000 | 106.2821419 | 176.9112644 | 64.42569861 |
| Incidence | High-income North America | Female | 15-49 years | Polycystic ovarian syndrome | Rate | 2001 | 104.428786 | 173.821411 | 63.50331299 |
| Incidence | High-income North America | Female | 15-49 years | Polycystic ovarian syndrome | Rate | 2002 | 102.3101509 | 169.6476286 | 62.86271668 |
| Incidence | High-income North America | Female | 15-49 years | Polycystic ovarian syndrome | Rate | 2003 | 100.2001502 | 165.9979725 | 62.14467023 |
| Incidence | High-income North America | Female | 15-49 years | Polycystic ovarian syndrome | Rate | 2004 | 98.4989659 | 162.1204455 | 61.97696489 |
| Incidence | High-income North America | Female | 15-49 years | Polycystic ovarian syndrome | Rate | 2005 | 97.08351829 | 158.4302574 | 61.43041808 |
| Incidence | High-income North America | Female | 15-49 years | Polycystic ovarian syndrome | Rate | 2006 | 94.95732871 | 151.2886901 | 61.68388579 |
| Incidence | High-income North America | Female | 15-49 years | Polycystic ovarian syndrome | Rate | 2007 | 91.94497253 | 139.5346669 | 60.88094542 |
| Incidence | High-income North America | Female | 15-49 years | Polycystic ovarian syndrome | Rate | 2008 | 88.53905496 | 132.5006612 | 59.47275304 |
| Incidence | High-income North America | Female | 15-49 years | Polycystic ovarian syndrome | Rate | 2009 | 85.45926995 | 123.3861848 | 58.01913618 |
| Incidence | High-income North America | Female | 15-49 years | Polycystic ovarian syndrome | Rate | 2010 | 83.64392268 | 120.459449 | 56.61172552 |
| Incidence | High-income North America | Female | 15-49 years | Polycystic ovarian syndrome | Rate | 2011 | 83.02720491 | 119.5699198 | 56.23330367 |
| Incidence | High-income North America | Female | 15-49 years | Polycystic ovarian syndrome | Rate | 2012 | 82.88707095 | 119.3124202 | 56.46934083 |
| Incidence | High-income North America | Female | 15-49 years | Polycystic ovarian syndrome | Rate | 2013 | 83.42657688 | 120.2774646 | 57.15826831 |
| Incidence | High-income North America | Female | 15-49 years | Polycystic ovarian syndrome | Rate | 2014 | 84.62854285 | 121.5848899 | 58.00214513 |
| Incidence | High-income North America | Female | 15-49 years | Polycystic ovarian syndrome | Rate | 2015 | 86.41524134 | 122.7426255 | 59.35524103 |
| Incidence | High-income North America | Female | 15-49 years | Polycystic ovarian syndrome | Rate | 2016 | 91.14460664 | 129.5861982 | 62.76166255 |
| Incidence | High-income North America | Female | 15-49 years | Polycystic ovarian syndrome | Rate | 2017 | 100.370906 | 143.1779119 | 68.48170758 |
| Incidence | High-income North America | Female | 15-49 years | Polycystic ovarian syndrome | Rate | 2018 | 112.355207 | 160.7902378 | 75.12871213 |
| Incidence | High-income North America | Female | 15-49 years | Polycystic ovarian syndrome | Rate | 2019 | 125.4092826 | 180.0198867 | 82.03713182 |
| Incidence | High-income North America | Female | 15-49 years | Polycystic ovarian syndrome | Rate | 2020 | 142.7783408 | 209.73552 | 89.02121146 |
| Incidence | High-income North America | Female | 15-49 years | Polycystic ovarian syndrome | Rate | 2021 | 156.7070688 | 241.3596268 | 93.55381993 |
| Incidence | Southern Latin America | Female | 15-49 years | Polycystic ovarian syndrome | Rate | 1990 | 58.41677824 | 91.44953513 | 36.1424681 |
| Incidence | Southern Latin America | Female | 15-49 years | Polycystic ovarian syndrome | Rate | 1991 | 59.83526074 | 93.98626452 | 36.9618876 |
| Incidence | Southern Latin America | Female | 15-49 years | Polycystic ovarian syndrome | Rate | 1992 | 61.11067798 | 95.78267149 | 37.94449468 |
| Incidence | Southern Latin America | Female | 15-49 years | Polycystic ovarian syndrome | Rate | 1993 | 62.41157847 | 97.61900515 | 38.83695681 |
| Incidence | Southern Latin America | Female | 15-49 years | Polycystic ovarian syndrome | Rate | 1994 | 63.75134908 | 99.52183698 | 39.67284767 |
| Incidence | Southern Latin America | Female | 15-49 years | Polycystic ovarian syndrome | Rate | 1995 | 65.04210368 | 101.7294288 | 40.41180027 |
| Incidence | Southern Latin America | Female | 15-49 years | Polycystic ovarian syndrome | Rate | 1996 | 66.48860608 | 103.8853834 | 41.6282433 |
| Incidence | Southern Latin America | Female | 15-49 years | Polycystic ovarian syndrome | Rate | 1997 | 68.19758149 | 106.2776642 | 42.9771724 |
| Incidence | Southern Latin America | Female | 15-49 years | Polycystic ovarian syndrome | Rate | 1998 | 69.8714788 | 109.696388 | 43.94409275 |
| Incidence | Southern Latin America | Female | 15-49 years | Polycystic ovarian syndrome | Rate | 1999 | 71.31335778 | 111.5442514 | 44.74891589 |
| Incidence | Southern Latin America | Female | 15-49 years | Polycystic ovarian syndrome | Rate | 2000 | 72.53465111 | 113.8558064 | 45.21819757 |
| Incidence | Southern Latin America | Female | 15-49 years | Polycystic ovarian syndrome | Rate | 2001 | 73.73315017 | 114.7045963 | 46.26654236 |
| Incidence | Southern Latin America | Female | 15-49 years | Polycystic ovarian syndrome | Rate | 2002 | 75.14331556 | 116.6135373 | 47.34346755 |
| Incidence | Southern Latin America | Female | 15-49 years | Polycystic ovarian syndrome | Rate | 2003 | 76.78428911 | 118.7438535 | 48.22407402 |
| Incidence | Southern Latin America | Female | 15-49 years | Polycystic ovarian syndrome | Rate | 2004 | 78.45361555 | 121.491064 | 49.64985249 |
| Incidence | Southern Latin America | Female | 15-49 years | Polycystic ovarian syndrome | Rate | 2005 | 79.81735669 | 123.5670408 | 50.39027602 |
| Incidence | Southern Latin America | Female | 15-49 years | Polycystic ovarian syndrome | Rate | 2006 | 80.8185431 | 124.5470161 | 51.03108456 |
| Incidence | Southern Latin America | Female | 15-49 years | Polycystic ovarian syndrome | Rate | 2007 | 81.54003686 | 125.3522302 | 50.80743969 |
| Incidence | Southern Latin America | Female | 15-49 years | Polycystic ovarian syndrome | Rate | 2008 | 81.87072446 | 125.9153434 | 50.84046497 |
| Incidence | Southern Latin America | Female | 15-49 years | Polycystic ovarian syndrome | Rate | 2009 | 81.76092218 | 126.5923283 | 50.70212234 |
| Incidence | Southern Latin America | Female | 15-49 years | Polycystic ovarian syndrome | Rate | 2010 | 81.16461836 | 125.8764313 | 50.30786973 |
| Incidence | Southern Latin America | Female | 15-49 years | Polycystic ovarian syndrome | Rate | 2011 | 80.33781779 | 125.538989 | 50.04633635 |
| Incidence | Southern Latin America | Female | 15-49 years | Polycystic ovarian syndrome | Rate | 2012 | 79.55864035 | 124.5045464 | 49.48236011 |
| Incidence | Southern Latin America | Female | 15-49 years | Polycystic ovarian syndrome | Rate | 2013 | 78.7998308 | 123.9279259 | 49.0601281 |
| Incidence | Southern Latin America | Female | 15-49 years | Polycystic ovarian syndrome | Rate | 2014 | 78.01776399 | 123.0467231 | 48.62433506 |
| Incidence | Southern Latin America | Female | 15-49 years | Polycystic ovarian syndrome | Rate | 2015 | 77.15935977 | 122.2813508 | 48.39810353 |
| Incidence | Southern Latin America | Female | 15-49 years | Polycystic ovarian syndrome | Rate | 2016 | 76.12734885 | 118.8953056 | 47.68777554 |
| Incidence | Southern Latin America | Female | 15-49 years | Polycystic ovarian syndrome | Rate | 2017 | 74.96292496 | 117.2532717 | 47.10232593 |
| Incidence | Southern Latin America | Female | 15-49 years | Polycystic ovarian syndrome | Rate | 2018 | 73.90914958 | 114.3500519 | 46.46371489 |
| Incidence | Southern Latin America | Female | 15-49 years | Polycystic ovarian syndrome | Rate | 2019 | 73.07836539 | 113.1880492 | 45.72304522 |
| Incidence | Southern Latin America | Female | 15-49 years | Polycystic ovarian syndrome | Rate | 2020 | 73.00850757 | 114.4381393 | 45.59341292 |
| Incidence | Southern Latin America | Female | 15-49 years | Polycystic ovarian syndrome | Rate | 2021 | 72.04381649 | 113.1548879 | 45.12037323 |
| Incidence | Andean Latin America | Female | 15-49 years | Polycystic ovarian syndrome | Rate | 1990 | 97.25816114 | 156.8136772 | 64.37704318 |
| Incidence | Andean Latin America | Female | 15-49 years | Polycystic ovarian syndrome | Rate | 1991 | 97.37251922 | 154.6080022 | 64.63542222 |
| Incidence | Andean Latin America | Female | 15-49 years | Polycystic ovarian syndrome | Rate | 1992 | 97.63799826 | 154.1547522 | 65.06088409 |
| Incidence | Andean Latin America | Female | 15-49 years | Polycystic ovarian syndrome | Rate | 1993 | 98.0660387 | 154.5478115 | 65.28516905 |
| Incidence | Andean Latin America | Female | 15-49 years | Polycystic ovarian syndrome | Rate | 1994 | 98.5820525 | 154.778278 | 65.54391126 |
| Incidence | Andean Latin America | Female | 15-49 years | Polycystic ovarian syndrome | Rate | 1995 | 99.1349394 | 154.8826015 | 66.13627732 |
| Incidence | Andean Latin America | Female | 15-49 years | Polycystic ovarian syndrome | Rate | 1996 | 100.0682349 | 154.7463502 | 67.39201547 |
| Incidence | Andean Latin America | Female | 15-49 years | Polycystic ovarian syndrome | Rate | 1997 | 101.4515835 | 155.9692032 | 69.27707966 |
| Incidence | Andean Latin America | Female | 15-49 years | Polycystic ovarian syndrome | Rate | 1998 | 102.8616219 | 158.1738823 | 70.47328473 |
| Incidence | Andean Latin America | Female | 15-49 years | Polycystic ovarian syndrome | Rate | 1999 | 103.9331179 | 160.7478235 | 71.23867266 |
| Incidence | Andean Latin America | Female | 15-49 years | Polycystic ovarian syndrome | Rate | 2000 | 104.2209893 | 162.3469867 | 71.28823721 |
| Incidence | Andean Latin America | Female | 15-49 years | Polycystic ovarian syndrome | Rate | 2001 | 103.8967803 | 162.8194879 | 70.84858665 |
| Incidence | Andean Latin America | Female | 15-49 years | Polycystic ovarian syndrome | Rate | 2002 | 103.4043267 | 163.2642902 | 69.93680756 |
| Incidence | Andean Latin America | Female | 15-49 years | Polycystic ovarian syndrome | Rate | 2003 | 102.895897 | 164.4868482 | 69.20116004 |
| Incidence | Andean Latin America | Female | 15-49 years | Polycystic ovarian syndrome | Rate | 2004 | 102.4688258 | 165.4866703 | 68.49312643 |
| Incidence | Andean Latin America | Female | 15-49 years | Polycystic ovarian syndrome | Rate | 2005 | 102.187629 | 165.5842192 | 68.24220664 |
| Incidence | Andean Latin America | Female | 15-49 years | Polycystic ovarian syndrome | Rate | 2006 | 102.2660157 | 164.9215706 | 68.35515795 |
| Incidence | Andean Latin America | Female | 15-49 years | Polycystic ovarian syndrome | Rate | 2007 | 102.7658173 | 164.0835552 | 69.50451759 |
| Incidence | Andean Latin America | Female | 15-49 years | Polycystic ovarian syndrome | Rate | 2008 | 103.3322472 | 163.2570617 | 70.23544119 |
| Incidence | Andean Latin America | Female | 15-49 years | Polycystic ovarian syndrome | Rate | 2009 | 103.5311206 | 164.3436992 | 70.65383416 |
| Incidence | Andean Latin America | Female | 15-49 years | Polycystic ovarian syndrome | Rate | 2010 | 103.0494056 | 162.6501227 | 69.66095563 |
| Incidence | Andean Latin America | Female | 15-49 years | Polycystic ovarian syndrome | Rate | 2011 | 101.888188 | 159.590352 | 69.90018051 |
| Incidence | Andean Latin America | Female | 15-49 years | Polycystic ovarian syndrome | Rate | 2012 | 100.2555157 | 155.3515899 | 69.09024835 |
| Incidence | Andean Latin America | Female | 15-49 years | Polycystic ovarian syndrome | Rate | 2013 | 98.41795195 | 150.8430205 | 67.83847148 |
| Incidence | Andean Latin America | Female | 15-49 years | Polycystic ovarian syndrome | Rate | 2014 | 96.64294533 | 148.4945637 | 66.22593716 |
| Incidence | Andean Latin America | Female | 15-49 years | Polycystic ovarian syndrome | Rate | 2015 | 95.11801812 | 146.0725517 | 65.20960714 |
| Incidence | Andean Latin America | Female | 15-49 years | Polycystic ovarian syndrome | Rate | 2016 | 93.96088805 | 142.3692678 | 64.89735358 |
| Incidence | Andean Latin America | Female | 15-49 years | Polycystic ovarian syndrome | Rate | 2017 | 92.97583636 | 139.9809818 | 64.53474562 |
| Incidence | Andean Latin America | Female | 15-49 years | Polycystic ovarian syndrome | Rate | 2018 | 91.80054512 | 138.0205617 | 63.5867878 |
| Incidence | Andean Latin America | Female | 15-49 years | Polycystic ovarian syndrome | Rate | 2019 | 90.31375226 | 135.7292338 | 62.82803076 |
| Incidence | Andean Latin America | Female | 15-49 years | Polycystic ovarian syndrome | Rate | 2020 | 88.83557756 | 135.5800956 | 60.87528896 |
| Incidence | Andean Latin America | Female | 15-49 years | Polycystic ovarian syndrome | Rate | 2021 | 88.49491916 | 134.5712897 | 60.58860019 |
| Incidence | North Africa and Middle East | Female | 15-49 years | Polycystic ovarian syndrome | Rate | 1990 | 75.04090792 | 115.0554752 | 48.5911484 |
| Incidence | North Africa and Middle East | Female | 15-49 years | Polycystic ovarian syndrome | Rate | 1991 | 74.98743768 | 114.9521733 | 48.34908672 |
| Incidence | North Africa and Middle East | Female | 15-49 years | Polycystic ovarian syndrome | Rate | 1992 | 74.89714461 | 114.8445752 | 48.09588746 |
| Incidence | North Africa and Middle East | Female | 15-49 years | Polycystic ovarian syndrome | Rate | 1993 | 74.85061213 | 115.2302531 | 48.00205444 |
| Incidence | North Africa and Middle East | Female | 15-49 years | Polycystic ovarian syndrome | Rate | 1994 | 74.97756759 | 115.3795242 | 48.10774367 |
| Incidence | North Africa and Middle East | Female | 15-49 years | Polycystic ovarian syndrome | Rate | 1995 | 75.42740896 | 116.2564248 | 48.46112551 |
| Incidence | North Africa and Middle East | Female | 15-49 years | Polycystic ovarian syndrome | Rate | 1996 | 76.12922679 | 117.0718232 | 48.89440578 |
| Incidence | North Africa and Middle East | Female | 15-49 years | Polycystic ovarian syndrome | Rate | 1997 | 77.00987705 | 118.6425448 | 49.59080387 |
| Incidence | North Africa and Middle East | Female | 15-49 years | Polycystic ovarian syndrome | Rate | 1998 | 77.87436432 | 119.8091986 | 50.37526534 |
| Incidence | North Africa and Middle East | Female | 15-49 years | Polycystic ovarian syndrome | Rate | 1999 | 78.53810759 | 120.904933 | 50.93339654 |
| Incidence | North Africa and Middle East | Female | 15-49 years | Polycystic ovarian syndrome | Rate | 2000 | 78.81374409 | 121.4311731 | 51.06976972 |
| Incidence | North Africa and Middle East | Female | 15-49 years | Polycystic ovarian syndrome | Rate | 2001 | 79.12521786 | 122.1196288 | 51.30180955 |
| Incidence | North Africa and Middle East | Female | 15-49 years | Polycystic ovarian syndrome | Rate | 2002 | 79.5200406 | 122.9776999 | 51.5272014 |
| Incidence | North Africa and Middle East | Female | 15-49 years | Polycystic ovarian syndrome | Rate | 2003 | 79.73892856 | 123.615025 | 51.64720626 |
| Incidence | North Africa and Middle East | Female | 15-49 years | Polycystic ovarian syndrome | Rate | 2004 | 79.60303213 | 123.4578893 | 51.52662012 |
| Incidence | North Africa and Middle East | Female | 15-49 years | Polycystic ovarian syndrome | Rate | 2005 | 78.89902111 | 122.0579317 | 51.01067122 |
| Incidence | North Africa and Middle East | Female | 15-49 years | Polycystic ovarian syndrome | Rate | 2006 | 77.74669497 | 120.0710412 | 50.47179349 |
| Incidence | North Africa and Middle East | Female | 15-49 years | Polycystic ovarian syndrome | Rate | 2007 | 76.47619791 | 117.8470755 | 49.83963341 |
| Incidence | North Africa and Middle East | Female | 15-49 years | Polycystic ovarian syndrome | Rate | 2008 | 75.23868172 | 115.5972361 | 49.06456561 |
| Incidence | North Africa and Middle East | Female | 15-49 years | Polycystic ovarian syndrome | Rate | 2009 | 74.09756899 | 113.5429513 | 48.31362453 |
| Incidence | North Africa and Middle East | Female | 15-49 years | Polycystic ovarian syndrome | Rate | 2010 | 73.02419792 | 111.7314892 | 47.6100115 |
| Incidence | North Africa and Middle East | Female | 15-49 years | Polycystic ovarian syndrome | Rate | 2011 | 72.06289371 | 110.3841925 | 47.05681025 |
| Incidence | North Africa and Middle East | Female | 15-49 years | Polycystic ovarian syndrome | Rate | 2012 | 71.2804912 | 109.1405045 | 46.6197623 |
| Incidence | North Africa and Middle East | Female | 15-49 years | Polycystic ovarian syndrome | Rate | 2013 | 70.61320357 | 108.0555285 | 46.24170947 |
| Incidence | North Africa and Middle East | Female | 15-49 years | Polycystic ovarian syndrome | Rate | 2014 | 70.04055876 | 107.095589 | 45.8487136 |
| Incidence | North Africa and Middle East | Female | 15-49 years | Polycystic ovarian syndrome | Rate | 2015 | 69.54268538 | 106.2412285 | 45.43309673 |
| Incidence | North Africa and Middle East | Female | 15-49 years | Polycystic ovarian syndrome | Rate | 2016 | 68.97272049 | 106.0888674 | 45.02800552 |
| Incidence | North Africa and Middle East | Female | 15-49 years | Polycystic ovarian syndrome | Rate | 2017 | 68.40906993 | 105.6200857 | 44.63048495 |
| Incidence | North Africa and Middle East | Female | 15-49 years | Polycystic ovarian syndrome | Rate | 2018 | 68.05378898 | 104.7233411 | 44.34219357 |
| Incidence | North Africa and Middle East | Female | 15-49 years | Polycystic ovarian syndrome | Rate | 2019 | 68.04726061 | 104.9225666 | 44.22879861 |
| Incidence | North Africa and Middle East | Female | 15-49 years | Polycystic ovarian syndrome | Rate | 2020 | 68.64333346 | 105.7934099 | 45.0599003 |
| Incidence | North Africa and Middle East | Female | 15-49 years | Polycystic ovarian syndrome | Rate | 2021 | 68.05809326 | 104.7582121 | 44.57682089 |
| Incidence | Central Latin America | Female | 15-49 years | Polycystic ovarian syndrome | Rate | 1990 | 96.59873828 | 137.3937526 | 67.94792366 |
| Incidence | Central Latin America | Female | 15-49 years | Polycystic ovarian syndrome | Rate | 1991 | 98.60831327 | 140.8737307 | 69.68360738 |
| Incidence | Central Latin America | Female | 15-49 years | Polycystic ovarian syndrome | Rate | 1992 | 99.77758269 | 142.8981525 | 70.66162289 |
| Incidence | Central Latin America | Female | 15-49 years | Polycystic ovarian syndrome | Rate | 1993 | 99.99700266 | 143.5779386 | 70.94850038 |
| Incidence | Central Latin America | Female | 15-49 years | Polycystic ovarian syndrome | Rate | 1994 | 99.32990284 | 142.9142548 | 70.41634937 |
| Incidence | Central Latin America | Female | 15-49 years | Polycystic ovarian syndrome | Rate | 1995 | 98.00485014 | 141.1284082 | 69.39522711 |
| Incidence | Central Latin America | Female | 15-49 years | Polycystic ovarian syndrome | Rate | 1996 | 96.10479458 | 137.7097019 | 68.18505077 |
| Incidence | Central Latin America | Female | 15-49 years | Polycystic ovarian syndrome | Rate | 1997 | 93.82877601 | 133.5059856 | 66.81855766 |
| Incidence | Central Latin America | Female | 15-49 years | Polycystic ovarian syndrome | Rate | 1998 | 91.5455101 | 129.3731388 | 65.42509806 |
| Incidence | Central Latin America | Female | 15-49 years | Polycystic ovarian syndrome | Rate | 1999 | 89.51228951 | 126.0421681 | 64.12631469 |
| Incidence | Central Latin America | Female | 15-49 years | Polycystic ovarian syndrome | Rate | 2000 | 87.81424299 | 123.5013903 | 63.12470585 |
| Incidence | Central Latin America | Female | 15-49 years | Polycystic ovarian syndrome | Rate | 2001 | 86.3998169 | 121.1970727 | 62.0296402 |
| Incidence | Central Latin America | Female | 15-49 years | Polycystic ovarian syndrome | Rate | 2002 | 84.96235274 | 119.0952937 | 60.94777829 |
| Incidence | Central Latin America | Female | 15-49 years | Polycystic ovarian syndrome | Rate | 2003 | 83.56589584 | 116.7077334 | 59.93482832 |
| Incidence | Central Latin America | Female | 15-49 years | Polycystic ovarian syndrome | Rate | 2004 | 82.2927886 | 114.3402333 | 59.1852451 |
| Incidence | Central Latin America | Female | 15-49 years | Polycystic ovarian syndrome | Rate | 2005 | 81.18180753 | 112.4997249 | 58.56138415 |
| Incidence | Central Latin America | Female | 15-49 years | Polycystic ovarian syndrome | Rate | 2006 | 80.12630811 | 111.0107888 | 58.11604136 |
| Incidence | Central Latin America | Female | 15-49 years | Polycystic ovarian syndrome | Rate | 2007 | 79.10186003 | 109.5588194 | 57.53163835 |
| Incidence | Central Latin America | Female | 15-49 years | Polycystic ovarian syndrome | Rate | 2008 | 78.17781042 | 108.5900517 | 57.0145493 |
| Incidence | Central Latin America | Female | 15-49 years | Polycystic ovarian syndrome | Rate | 2009 | 77.385627 | 107.8142253 | 56.70090615 |
| Incidence | Central Latin America | Female | 15-49 years | Polycystic ovarian syndrome | Rate | 2010 | 76.7987387 | 106.9835886 | 56.25733041 |
| Incidence | Central Latin America | Female | 15-49 years | Polycystic ovarian syndrome | Rate | 2011 | 76.389642 | 106.2673938 | 55.82240123 |
| Incidence | Central Latin America | Female | 15-49 years | Polycystic ovarian syndrome | Rate | 2012 | 76.03075085 | 105.9285746 | 55.36757366 |
| Incidence | Central Latin America | Female | 15-49 years | Polycystic ovarian syndrome | Rate | 2013 | 75.67875379 | 104.8628594 | 55.00542353 |
| Incidence | Central Latin America | Female | 15-49 years | Polycystic ovarian syndrome | Rate | 2014 | 75.3141868 | 104.615873 | 54.49004988 |
| Incidence | Central Latin America | Female | 15-49 years | Polycystic ovarian syndrome | Rate | 2015 | 74.91602344 | 104.1029683 | 53.88937408 |
| Incidence | Central Latin America | Female | 15-49 years | Polycystic ovarian syndrome | Rate | 2016 | 74.83634492 | 104.0727811 | 53.73187244 |
| Incidence | Central Latin America | Female | 15-49 years | Polycystic ovarian syndrome | Rate | 2017 | 75.15864518 | 104.6683656 | 53.82855775 |
| Incidence | Central Latin America | Female | 15-49 years | Polycystic ovarian syndrome | Rate | 2018 | 75.56786846 | 105.3959085 | 54.06548925 |
| Incidence | Central Latin America | Female | 15-49 years | Polycystic ovarian syndrome | Rate | 2019 | 75.67655255 | 105.5821679 | 54.07715764 |
| Incidence | Central Latin America | Female | 15-49 years | Polycystic ovarian syndrome | Rate | 2020 | 75.40835445 | 106.4894331 | 54.00702779 |
| Incidence | Central Latin America | Female | 15-49 years | Polycystic ovarian syndrome | Rate | 2021 | 75.35424588 | 106.5927319 | 53.80532921 |
| Incidence | Tropical Latin America | Female | 15-49 years | Polycystic ovarian syndrome | Rate | 1990 | 26.82591238 | 39.39032032 | 18.43775919 |
| Incidence | Tropical Latin America | Female | 15-49 years | Polycystic ovarian syndrome | Rate | 1991 | 27.36684498 | 40.27386149 | 18.79605658 |
| Incidence | Tropical Latin America | Female | 15-49 years | Polycystic ovarian syndrome | Rate | 1992 | 27.84335867 | 41.09438189 | 19.02210193 |
| Incidence | Tropical Latin America | Female | 15-49 years | Polycystic ovarian syndrome | Rate | 1993 | 28.22053107 | 41.64500069 | 19.21327614 |
| Incidence | Tropical Latin America | Female | 15-49 years | Polycystic ovarian syndrome | Rate | 1994 | 28.45459541 | 41.99561884 | 19.35891143 |
| Incidence | Tropical Latin America | Female | 15-49 years | Polycystic ovarian syndrome | Rate | 1995 | 28.50073501 | 42.2162979 | 19.4042111 |
| Incidence | Tropical Latin America | Female | 15-49 years | Polycystic ovarian syndrome | Rate | 1996 | 28.37713092 | 42.00722153 | 19.27456334 |
| Incidence | Tropical Latin America | Female | 15-49 years | Polycystic ovarian syndrome | Rate | 1997 | 28.15413278 | 41.61784095 | 19.15636279 |
| Incidence | Tropical Latin America | Female | 15-49 years | Polycystic ovarian syndrome | Rate | 1998 | 27.84762192 | 40.96808222 | 18.97015641 |
| Incidence | Tropical Latin America | Female | 15-49 years | Polycystic ovarian syndrome | Rate | 1999 | 27.48337675 | 40.23163106 | 18.74418365 |
| Incidence | Tropical Latin America | Female | 15-49 years | Polycystic ovarian syndrome | Rate | 2000 | 27.06643324 | 39.43218098 | 18.48048303 |
| Incidence | Tropical Latin America | Female | 15-49 years | Polycystic ovarian syndrome | Rate | 2001 | 26.60521564 | 38.5476841 | 18.18134745 |
| Incidence | Tropical Latin America | Female | 15-49 years | Polycystic ovarian syndrome | Rate | 2002 | 26.03887585 | 37.53753377 | 17.80314336 |
| Incidence | Tropical Latin America | Female | 15-49 years | Polycystic ovarian syndrome | Rate | 2003 | 25.39797672 | 36.44132369 | 17.35173908 |
| Incidence | Tropical Latin America | Female | 15-49 years | Polycystic ovarian syndrome | Rate | 2004 | 24.71119133 | 35.44382929 | 16.86014508 |
| Incidence | Tropical Latin America | Female | 15-49 years | Polycystic ovarian syndrome | Rate | 2005 | 24.02041293 | 34.33945645 | 16.36425243 |
| Incidence | Tropical Latin America | Female | 15-49 years | Polycystic ovarian syndrome | Rate | 2006 | 23.16590993 | 32.9940077 | 15.85226699 |
| Incidence | Tropical Latin America | Female | 15-49 years | Polycystic ovarian syndrome | Rate | 2007 | 22.11639903 | 31.23965279 | 15.19907415 |
| Incidence | Tropical Latin America | Female | 15-49 years | Polycystic ovarian syndrome | Rate | 2008 | 21.1010881 | 29.76663457 | 14.66704485 |
| Incidence | Tropical Latin America | Female | 15-49 years | Polycystic ovarian syndrome | Rate | 2009 | 20.32237006 | 28.66609152 | 14.25962343 |
| Incidence | Tropical Latin America | Female | 15-49 years | Polycystic ovarian syndrome | Rate | 2010 | 19.96187362 | 28.19581453 | 14.04711526 |
| Incidence | Tropical Latin America | Female | 15-49 years | Polycystic ovarian syndrome | Rate | 2011 | 19.95073328 | 28.22852826 | 14.0256408 |
| Incidence | Tropical Latin America | Female | 15-49 years | Polycystic ovarian syndrome | Rate | 2012 | 20.0576491 | 28.41221076 | 14.0871466 |
| Incidence | Tropical Latin America | Female | 15-49 years | Polycystic ovarian syndrome | Rate | 2013 | 20.21735559 | 28.6820261 | 14.18715174 |
| Incidence | Tropical Latin America | Female | 15-49 years | Polycystic ovarian syndrome | Rate | 2014 | 20.37118045 | 28.94634696 | 14.30057654 |
| Incidence | Tropical Latin America | Female | 15-49 years | Polycystic ovarian syndrome | Rate | 2015 | 20.46252842 | 29.11380896 | 14.39483644 |
| Incidence | Tropical Latin America | Female | 15-49 years | Polycystic ovarian syndrome | Rate | 2016 | 20.60019874 | 29.40324324 | 14.43772462 |
| Incidence | Tropical Latin America | Female | 15-49 years | Polycystic ovarian syndrome | Rate | 2017 | 20.81722304 | 29.842685 | 14.52953253 |
| Incidence | Tropical Latin America | Female | 15-49 years | Polycystic ovarian syndrome | Rate | 2018 | 20.95180547 | 30.04442497 | 14.56623332 |
| Incidence | Tropical Latin America | Female | 15-49 years | Polycystic ovarian syndrome | Rate | 2019 | 20.86429816 | 29.8575683 | 14.51852809 |
| Incidence | Tropical Latin America | Female | 15-49 years | Polycystic ovarian syndrome | Rate | 2020 | 20.51674784 | 29.08070946 | 14.31004572 |
| Incidence | Tropical Latin America | Female | 15-49 years | Polycystic ovarian syndrome | Rate | 2021 | 20.18022959 | 28.85338366 | 14.05818571 |
| Incidence | South Asia | Female | 15-49 years | Polycystic ovarian syndrome | Rate | 1990 | 33.56987332 | 47.28097149 | 23.23298491 |
| Incidence | South Asia | Female | 15-49 years | Polycystic ovarian syndrome | Rate | 1991 | 33.58151692 | 47.36044161 | 23.23756115 |
| Incidence | South Asia | Female | 15-49 years | Polycystic ovarian syndrome | Rate | 1992 | 33.65264632 | 47.37459936 | 23.28114336 |
| Incidence | South Asia | Female | 15-49 years | Polycystic ovarian syndrome | Rate | 1993 | 33.7895979 | 47.48864843 | 23.3675938 |
| Incidence | South Asia | Female | 15-49 years | Polycystic ovarian syndrome | Rate | 1994 | 33.98412426 | 47.70835212 | 23.48913162 |
| Incidence | South Asia | Female | 15-49 years | Polycystic ovarian syndrome | Rate | 1995 | 34.22253447 | 48.08797962 | 23.62146317 |
| Incidence | South Asia | Female | 15-49 years | Polycystic ovarian syndrome | Rate | 1996 | 34.5267348 | 48.54756218 | 23.79572438 |
| Incidence | South Asia | Female | 15-49 years | Polycystic ovarian syndrome | Rate | 1997 | 34.92303462 | 49.20315696 | 23.97989222 |
| Incidence | South Asia | Female | 15-49 years | Polycystic ovarian syndrome | Rate | 1998 | 35.35179471 | 50.08395608 | 24.1324129 |
| Incidence | South Asia | Female | 15-49 years | Polycystic ovarian syndrome | Rate | 1999 | 35.76890836 | 50.64855534 | 24.27632116 |
| Incidence | South Asia | Female | 15-49 years | Polycystic ovarian syndrome | Rate | 2000 | 36.1120623 | 51.20981865 | 24.3870511 |
| Incidence | South Asia | Female | 15-49 years | Polycystic ovarian syndrome | Rate | 2001 | 36.47551678 | 51.60268019 | 24.65930631 |
| Incidence | South Asia | Female | 15-49 years | Polycystic ovarian syndrome | Rate | 2002 | 36.84777938 | 52.14633329 | 24.9571763 |
| Incidence | South Asia | Female | 15-49 years | Polycystic ovarian syndrome | Rate | 2003 | 37.20719374 | 52.58252988 | 25.31751807 |
| Incidence | South Asia | Female | 15-49 years | Polycystic ovarian syndrome | Rate | 2004 | 37.55145063 | 53.0237516 | 25.62847404 |
| Incidence | South Asia | Female | 15-49 years | Polycystic ovarian syndrome | Rate | 2005 | 37.88772224 | 53.36813712 | 25.80072251 |
| Incidence | South Asia | Female | 15-49 years | Polycystic ovarian syndrome | Rate | 2006 | 38.54997217 | 54.37586196 | 26.20907183 |
| Incidence | South Asia | Female | 15-49 years | Polycystic ovarian syndrome | Rate | 2007 | 39.66962973 | 56.07288773 | 26.88709906 |
| Incidence | South Asia | Female | 15-49 years | Polycystic ovarian syndrome | Rate | 2008 | 40.98629982 | 58.08088833 | 27.68275243 |
| Incidence | South Asia | Female | 15-49 years | Polycystic ovarian syndrome | Rate | 2009 | 42.23210928 | 60.03657754 | 28.40006754 |
| Incidence | South Asia | Female | 15-49 years | Polycystic ovarian syndrome | Rate | 2010 | 43.13610869 | 61.48554663 | 28.92997805 |
| Incidence | South Asia | Female | 15-49 years | Polycystic ovarian syndrome | Rate | 2011 | 43.72865885 | 62.40682693 | 29.33775044 |
| Incidence | South Asia | Female | 15-49 years | Polycystic ovarian syndrome | Rate | 2012 | 44.21769906 | 63.28341479 | 29.68969162 |
| Incidence | South Asia | Female | 15-49 years | Polycystic ovarian syndrome | Rate | 2013 | 44.58276352 | 63.84573645 | 29.95587561 |
| Incidence | South Asia | Female | 15-49 years | Polycystic ovarian syndrome | Rate | 2014 | 44.82349888 | 64.15132934 | 30.10621145 |
| Incidence | South Asia | Female | 15-49 years | Polycystic ovarian syndrome | Rate | 2015 | 44.95632622 | 64.30687342 | 30.26830666 |
| Incidence | South Asia | Female | 15-49 years | Polycystic ovarian syndrome | Rate | 2016 | 45.02470535 | 64.56944824 | 30.25568737 |
| Incidence | South Asia | Female | 15-49 years | Polycystic ovarian syndrome | Rate | 2017 | 45.04370568 | 64.57952094 | 30.19699287 |
| Incidence | South Asia | Female | 15-49 years | Polycystic ovarian syndrome | Rate | 2018 | 45.01281325 | 64.52732173 | 30.14306687 |
| Incidence | South Asia | Female | 15-49 years | Polycystic ovarian syndrome | Rate | 2019 | 44.91286309 | 64.32426893 | 30.04569645 |
| Incidence | South Asia | Female | 15-49 years | Polycystic ovarian syndrome | Rate | 2020 | 44.68631464 | 63.74535964 | 29.88140097 |
| Incidence | South Asia | Female | 15-49 years | Polycystic ovarian syndrome | Rate | 2021 | 44.64127809 | 63.61508318 | 29.90687251 |
| Incidence | Southern Sub-Saharan Africa | Female | 15-49 years | Polycystic ovarian syndrome | Rate | 1990 | 47.32320981 | 69.97677787 | 31.01340558 |
| Incidence | Southern Sub-Saharan Africa | Female | 15-49 years | Polycystic ovarian syndrome | Rate | 1991 | 47.34581088 | 69.90435804 | 31.03402908 |
| Incidence | Southern Sub-Saharan Africa | Female | 15-49 years | Polycystic ovarian syndrome | Rate | 1992 | 47.33390742 | 69.92538357 | 31.04732252 |
| Incidence | Southern Sub-Saharan Africa | Female | 15-49 years | Polycystic ovarian syndrome | Rate | 1993 | 47.30725088 | 70.00229002 | 31.09643296 |
| Incidence | Southern Sub-Saharan Africa | Female | 15-49 years | Polycystic ovarian syndrome | Rate | 1994 | 47.29331889 | 70.08967574 | 31.08157925 |
| Incidence | Southern Sub-Saharan Africa | Female | 15-49 years | Polycystic ovarian syndrome | Rate | 1995 | 47.32868928 | 70.41480088 | 31.033767 |
| Incidence | Southern Sub-Saharan Africa | Female | 15-49 years | Polycystic ovarian syndrome | Rate | 1996 | 47.6393523 | 70.78208008 | 31.19524462 |
| Incidence | Southern Sub-Saharan Africa | Female | 15-49 years | Polycystic ovarian syndrome | Rate | 1997 | 48.25454944 | 71.78648202 | 31.45032926 |
| Incidence | Southern Sub-Saharan Africa | Female | 15-49 years | Polycystic ovarian syndrome | Rate | 1998 | 49.01443168 | 72.79590595 | 31.793371 |
| Incidence | Southern Sub-Saharan Africa | Female | 15-49 years | Polycystic ovarian syndrome | Rate | 1999 | 49.73273208 | 73.73001214 | 32.18473145 |
| Incidence | Southern Sub-Saharan Africa | Female | 15-49 years | Polycystic ovarian syndrome | Rate | 2000 | 50.19937326 | 74.38665716 | 32.42942909 |
| Incidence | Southern Sub-Saharan Africa | Female | 15-49 years | Polycystic ovarian syndrome | Rate | 2001 | 50.52323431 | 74.60765493 | 32.57044264 |
| Incidence | Southern Sub-Saharan Africa | Female | 15-49 years | Polycystic ovarian syndrome | Rate | 2002 | 50.68770851 | 74.68991832 | 32.80476764 |
| Incidence | Southern Sub-Saharan Africa | Female | 15-49 years | Polycystic ovarian syndrome | Rate | 2003 | 50.71112419 | 74.60391021 | 33.04119594 |
| Incidence | Southern Sub-Saharan Africa | Female | 15-49 years | Polycystic ovarian syndrome | Rate | 2004 | 50.61370327 | 74.48748122 | 33.05551061 |
| Incidence | Southern Sub-Saharan Africa | Female | 15-49 years | Polycystic ovarian syndrome | Rate | 2005 | 50.42224024 | 74.4921602 | 32.78745011 |
| Incidence | Southern Sub-Saharan Africa | Female | 15-49 years | Polycystic ovarian syndrome | Rate | 2006 | 50.21764287 | 74.14394851 | 32.71606204 |
| Incidence | Southern Sub-Saharan Africa | Female | 15-49 years | Polycystic ovarian syndrome | Rate | 2007 | 50.00111989 | 73.99080741 | 32.57673017 |
| Incidence | Southern Sub-Saharan Africa | Female | 15-49 years | Polycystic ovarian syndrome | Rate | 2008 | 49.6884398 | 73.87183062 | 32.34209569 |
| Incidence | Southern Sub-Saharan Africa | Female | 15-49 years | Polycystic ovarian syndrome | Rate | 2009 | 49.21644892 | 73.22059079 | 32.01952268 |
| Incidence | Southern Sub-Saharan Africa | Female | 15-49 years | Polycystic ovarian syndrome | Rate | 2010 | 48.51513097 | 72.10173029 | 31.48600358 |
| Incidence | Southern Sub-Saharan Africa | Female | 15-49 years | Polycystic ovarian syndrome | Rate | 2011 | 47.60957136 | 70.57579294 | 30.98789479 |
| Incidence | Southern Sub-Saharan Africa | Female | 15-49 years | Polycystic ovarian syndrome | Rate | 2012 | 46.61256151 | 68.89135048 | 30.46631445 |
| Incidence | Southern Sub-Saharan Africa | Female | 15-49 years | Polycystic ovarian syndrome | Rate | 2013 | 45.60955177 | 67.16785989 | 29.88851072 |
| Incidence | Southern Sub-Saharan Africa | Female | 15-49 years | Polycystic ovarian syndrome | Rate | 2014 | 44.67268034 | 65.60503047 | 29.29350556 |
| Incidence | Southern Sub-Saharan Africa | Female | 15-49 years | Polycystic ovarian syndrome | Rate | 2015 | 43.86969118 | 64.18874192 | 28.83683636 |
| Incidence | Southern Sub-Saharan Africa | Female | 15-49 years | Polycystic ovarian syndrome | Rate | 2016 | 43.29101074 | 63.38680839 | 28.4510037 |
| Incidence | Southern Sub-Saharan Africa | Female | 15-49 years | Polycystic ovarian syndrome | Rate | 2017 | 42.99367292 | 63.02655605 | 28.31345597 |
| Incidence | Southern Sub-Saharan Africa | Female | 15-49 years | Polycystic ovarian syndrome | Rate | 2018 | 42.94826196 | 62.9622494 | 28.23204861 |
| Incidence | Southern Sub-Saharan Africa | Female | 15-49 years | Polycystic ovarian syndrome | Rate | 2019 | 43.11843084 | 63.3408716 | 28.26224056 |
| Incidence | Southern Sub-Saharan Africa | Female | 15-49 years | Polycystic ovarian syndrome | Rate | 2020 | 43.8554128 | 65.32093951 | 28.55281628 |
| Incidence | Southern Sub-Saharan Africa | Female | 15-49 years | Polycystic ovarian syndrome | Rate | 2021 | 43.75220732 | 64.65071284 | 28.59074414 |
| Incidence | Western Sub-Saharan Africa | Female | 15-49 years | Polycystic ovarian syndrome | Rate | 1990 | 31.99663539 | 46.90161849 | 21.22964527 |
| Incidence | Western Sub-Saharan Africa | Female | 15-49 years | Polycystic ovarian syndrome | Rate | 1991 | 33.05912932 | 48.55607267 | 21.90985384 |
| Incidence | Western Sub-Saharan Africa | Female | 15-49 years | Polycystic ovarian syndrome | Rate | 1992 | 34.07316481 | 50.15022897 | 22.55494157 |
| Incidence | Western Sub-Saharan Africa | Female | 15-49 years | Polycystic ovarian syndrome | Rate | 1993 | 34.99039582 | 51.58887073 | 23.13223589 |
| Incidence | Western Sub-Saharan Africa | Female | 15-49 years | Polycystic ovarian syndrome | Rate | 1994 | 35.80956578 | 52.80661213 | 23.6687696 |
| Incidence | Western Sub-Saharan Africa | Female | 15-49 years | Polycystic ovarian syndrome | Rate | 1995 | 36.43467414 | 53.793298 | 24.05560925 |
| Incidence | Western Sub-Saharan Africa | Female | 15-49 years | Polycystic ovarian syndrome | Rate | 1996 | 36.95914285 | 54.59313014 | 24.40386361 |
| Incidence | Western Sub-Saharan Africa | Female | 15-49 years | Polycystic ovarian syndrome | Rate | 1997 | 37.42686756 | 55.32622176 | 24.69879643 |
| Incidence | Western Sub-Saharan Africa | Female | 15-49 years | Polycystic ovarian syndrome | Rate | 1998 | 37.80498865 | 55.92914582 | 24.95541881 |
| Incidence | Western Sub-Saharan Africa | Female | 15-49 years | Polycystic ovarian syndrome | Rate | 1999 | 38.09662469 | 56.39469516 | 25.14597703 |
| Incidence | Western Sub-Saharan Africa | Female | 15-49 years | Polycystic ovarian syndrome | Rate | 2000 | 38.3775264 | 56.77558677 | 25.33423928 |
| Incidence | Western Sub-Saharan Africa | Female | 15-49 years | Polycystic ovarian syndrome | Rate | 2001 | 38.5704715 | 57.1120839 | 25.45831385 |
| Incidence | Western Sub-Saharan Africa | Female | 15-49 years | Polycystic ovarian syndrome | Rate | 2002 | 38.75769055 | 57.35341495 | 25.55558732 |
| Incidence | Western Sub-Saharan Africa | Female | 15-49 years | Polycystic ovarian syndrome | Rate | 2003 | 38.90622648 | 57.57614551 | 25.65579056 |
| Incidence | Western Sub-Saharan Africa | Female | 15-49 years | Polycystic ovarian syndrome | Rate | 2004 | 38.9906848 | 57.84505557 | 25.68929025 |
| Incidence | Western Sub-Saharan Africa | Female | 15-49 years | Polycystic ovarian syndrome | Rate | 2005 | 38.98265279 | 57.98015413 | 25.63876064 |
| Incidence | Western Sub-Saharan Africa | Female | 15-49 years | Polycystic ovarian syndrome | Rate | 2006 | 38.93978198 | 57.88518646 | 25.61182393 |
| Incidence | Western Sub-Saharan Africa | Female | 15-49 years | Polycystic ovarian syndrome | Rate | 2007 | 38.94780133 | 57.91968674 | 25.60500158 |
| Incidence | Western Sub-Saharan Africa | Female | 15-49 years | Polycystic ovarian syndrome | Rate | 2008 | 38.99044532 | 58.17255 | 25.61209998 |
| Incidence | Western Sub-Saharan Africa | Female | 15-49 years | Polycystic ovarian syndrome | Rate | 2009 | 39.04406482 | 58.29877322 | 25.66565752 |
| Incidence | Western Sub-Saharan Africa | Female | 15-49 years | Polycystic ovarian syndrome | Rate | 2010 | 39.0926326 | 58.3757231 | 25.70872734 |
| Incidence | Western Sub-Saharan Africa | Female | 15-49 years | Polycystic ovarian syndrome | Rate | 2011 | 39.13546326 | 58.39039972 | 25.72368287 |
| Incidence | Western Sub-Saharan Africa | Female | 15-49 years | Polycystic ovarian syndrome | Rate | 2012 | 39.17501397 | 58.3721889 | 25.74265579 |
| Incidence | Western Sub-Saharan Africa | Female | 15-49 years | Polycystic ovarian syndrome | Rate | 2013 | 39.22285169 | 58.25634652 | 25.79133781 |
| Incidence | Western Sub-Saharan Africa | Female | 15-49 years | Polycystic ovarian syndrome | Rate | 2014 | 39.28850776 | 58.32276383 | 25.85487115 |
| Incidence | Western Sub-Saharan Africa | Female | 15-49 years | Polycystic ovarian syndrome | Rate | 2015 | 39.37605012 | 58.42000844 | 25.90673508 |
| Incidence | Western Sub-Saharan Africa | Female | 15-49 years | Polycystic ovarian syndrome | Rate | 2016 | 39.53796074 | 58.65107716 | 25.93747831 |
| Incidence | Western Sub-Saharan Africa | Female | 15-49 years | Polycystic ovarian syndrome | Rate | 2017 | 39.80793967 | 59.01366002 | 26.05180666 |
| Incidence | Western Sub-Saharan Africa | Female | 15-49 years | Polycystic ovarian syndrome | Rate | 2018 | 40.15494416 | 59.44070702 | 26.26009758 |
| Incidence | Western Sub-Saharan Africa | Female | 15-49 years | Polycystic ovarian syndrome | Rate | 2019 | 40.54830336 | 59.90016604 | 26.54727319 |
| Incidence | Western Sub-Saharan Africa | Female | 15-49 years | Polycystic ovarian syndrome | Rate | 2020 | 41.45458558 | 61.3353722 | 27.1271706 |
| Incidence | Western Sub-Saharan Africa | Female | 15-49 years | Polycystic ovarian syndrome | Rate | 2021 | 41.10066873 | 60.52202544 | 26.91538177 |
| Incidence | Central Sub-Saharan Africa | Female | 15-49 years | Polycystic ovarian syndrome | Rate | 1990 | 27.38395151 | 40.48110902 | 17.76460278 |
| Incidence | Central Sub-Saharan Africa | Female | 15-49 years | Polycystic ovarian syndrome | Rate | 1991 | 27.47934029 | 40.68421654 | 17.81345369 |
| Incidence | Central Sub-Saharan Africa | Female | 15-49 years | Polycystic ovarian syndrome | Rate | 1992 | 27.64267879 | 40.58489421 | 18.06325944 |
| Incidence | Central Sub-Saharan Africa | Female | 15-49 years | Polycystic ovarian syndrome | Rate | 1993 | 27.86175713 | 41.06137446 | 18.22105613 |
| Incidence | Central Sub-Saharan Africa | Female | 15-49 years | Polycystic ovarian syndrome | Rate | 1994 | 28.12585477 | 41.34443455 | 18.22203874 |
| Incidence | Central Sub-Saharan Africa | Female | 15-49 years | Polycystic ovarian syndrome | Rate | 1995 | 28.41983565 | 41.5580402 | 18.30825025 |
| Incidence | Central Sub-Saharan Africa | Female | 15-49 years | Polycystic ovarian syndrome | Rate | 1996 | 29.10884565 | 42.90841043 | 18.80881503 |
| Incidence | Central Sub-Saharan Africa | Female | 15-49 years | Polycystic ovarian syndrome | Rate | 1997 | 30.31229213 | 44.90814572 | 19.68894368 |
| Incidence | Central Sub-Saharan Africa | Female | 15-49 years | Polycystic ovarian syndrome | Rate | 1998 | 31.66245184 | 46.54845979 | 20.62709085 |
| Incidence | Central Sub-Saharan Africa | Female | 15-49 years | Polycystic ovarian syndrome | Rate | 1999 | 32.79121552 | 48.40716791 | 21.31053085 |
| Incidence | Central Sub-Saharan Africa | Female | 15-49 years | Polycystic ovarian syndrome | Rate | 2000 | 33.29981299 | 49.22590127 | 21.57141059 |
| Incidence | Central Sub-Saharan Africa | Female | 15-49 years | Polycystic ovarian syndrome | Rate | 2001 | 33.44992553 | 49.34600457 | 21.75595933 |
| Incidence | Central Sub-Saharan Africa | Female | 15-49 years | Polycystic ovarian syndrome | Rate | 2002 | 33.56218143 | 49.89854825 | 21.86498528 |
| Incidence | Central Sub-Saharan Africa | Female | 15-49 years | Polycystic ovarian syndrome | Rate | 2003 | 33.63556819 | 49.64127265 | 21.97459937 |
| Incidence | Central Sub-Saharan Africa | Female | 15-49 years | Polycystic ovarian syndrome | Rate | 2004 | 33.67023936 | 49.46421656 | 22.03905571 |
| Incidence | Central Sub-Saharan Africa | Female | 15-49 years | Polycystic ovarian syndrome | Rate | 2005 | 33.66666216 | 49.75133123 | 22.00006241 |
| Incidence | Central Sub-Saharan Africa | Female | 15-49 years | Polycystic ovarian syndrome | Rate | 2006 | 33.6484187 | 49.40235623 | 22.03797101 |
| Incidence | Central Sub-Saharan Africa | Female | 15-49 years | Polycystic ovarian syndrome | Rate | 2007 | 33.63172914 | 49.49703324 | 21.97070889 |
| Incidence | Central Sub-Saharan Africa | Female | 15-49 years | Polycystic ovarian syndrome | Rate | 2008 | 33.62361699 | 49.52840171 | 21.90923352 |
| Incidence | Central Sub-Saharan Africa | Female | 15-49 years | Polycystic ovarian syndrome | Rate | 2009 | 33.63220774 | 49.68909964 | 21.90663716 |
| Incidence | Central Sub-Saharan Africa | Female | 15-49 years | Polycystic ovarian syndrome | Rate | 2010 | 33.67058702 | 49.72261969 | 21.84321653 |
| Incidence | Central Sub-Saharan Africa | Female | 15-49 years | Polycystic ovarian syndrome | Rate | 2011 | 33.8164226 | 49.4688455 | 21.97706817 |
| Incidence | Central Sub-Saharan Africa | Female | 15-49 years | Polycystic ovarian syndrome | Rate | 2012 | 34.10388917 | 50.22747351 | 22.19818803 |
| Incidence | Central Sub-Saharan Africa | Female | 15-49 years | Polycystic ovarian syndrome | Rate | 2013 | 34.47207281 | 51.08787707 | 22.44192368 |
| Incidence | Central Sub-Saharan Africa | Female | 15-49 years | Polycystic ovarian syndrome | Rate | 2014 | 34.86448561 | 51.41669978 | 22.65298179 |
| Incidence | Central Sub-Saharan Africa | Female | 15-49 years | Polycystic ovarian syndrome | Rate | 2015 | 35.21566821 | 52.01475895 | 22.81109571 |
| Incidence | Central Sub-Saharan Africa | Female | 15-49 years | Polycystic ovarian syndrome | Rate | 2016 | 35.50262019 | 52.0830811 | 23.02994233 |
| Incidence | Central Sub-Saharan Africa | Female | 15-49 years | Polycystic ovarian syndrome | Rate | 2017 | 35.78698783 | 52.94518807 | 23.11905566 |
| Incidence | Central Sub-Saharan Africa | Female | 15-49 years | Polycystic ovarian syndrome | Rate | 2018 | 36.13620308 | 53.48404801 | 23.2616269 |
| Incidence | Central Sub-Saharan Africa | Female | 15-49 years | Polycystic ovarian syndrome | Rate | 2019 | 36.6099928 | 54.66536435 | 23.6219391 |
| Incidence | Central Sub-Saharan Africa | Female | 15-49 years | Polycystic ovarian syndrome | Rate | 2020 | 37.46589477 | 55.84342843 | 24.38666748 |
| Incidence | Central Sub-Saharan Africa | Female | 15-49 years | Polycystic ovarian syndrome | Rate | 2021 | 37.43269335 | 56.29631102 | 24.26879909 |
| Incidence | Eastern Sub-Saharan Africa | Female | 15-49 years | Polycystic ovarian syndrome | Rate | 1990 | 33.21274123 | 49.31622746 | 21.85466491 |
| Incidence | Eastern Sub-Saharan Africa | Female | 15-49 years | Polycystic ovarian syndrome | Rate | 1991 | 33.33693885 | 49.66807032 | 21.9670271 |
| Incidence | Eastern Sub-Saharan Africa | Female | 15-49 years | Polycystic ovarian syndrome | Rate | 1992 | 33.49275181 | 49.9596464 | 22.09765762 |
| Incidence | Eastern Sub-Saharan Africa | Female | 15-49 years | Polycystic ovarian syndrome | Rate | 1993 | 33.67704147 | 50.12783085 | 22.23664716 |
| Incidence | Eastern Sub-Saharan Africa | Female | 15-49 years | Polycystic ovarian syndrome | Rate | 1994 | 33.8635028 | 50.33663426 | 22.37732592 |
| Incidence | Eastern Sub-Saharan Africa | Female | 15-49 years | Polycystic ovarian syndrome | Rate | 1995 | 34.04344848 | 50.63573717 | 22.51648327 |
| Incidence | Eastern Sub-Saharan Africa | Female | 15-49 years | Polycystic ovarian syndrome | Rate | 1996 | 34.3217151 | 51.01530279 | 22.67430099 |
| Incidence | Eastern Sub-Saharan Africa | Female | 15-49 years | Polycystic ovarian syndrome | Rate | 1997 | 34.73558873 | 51.77672697 | 22.9011445 |
| Incidence | Eastern Sub-Saharan Africa | Female | 15-49 years | Polycystic ovarian syndrome | Rate | 1998 | 35.17138733 | 52.46620194 | 23.16337297 |
| Incidence | Eastern Sub-Saharan Africa | Female | 15-49 years | Polycystic ovarian syndrome | Rate | 1999 | 35.51310594 | 52.92370137 | 23.38839718 |
| Incidence | Eastern Sub-Saharan Africa | Female | 15-49 years | Polycystic ovarian syndrome | Rate | 2000 | 35.65764762 | 53.04579516 | 23.46100698 |
| Incidence | Eastern Sub-Saharan Africa | Female | 15-49 years | Polycystic ovarian syndrome | Rate | 2001 | 35.77594784 | 53.10701245 | 23.55330637 |
| Incidence | Eastern Sub-Saharan Africa | Female | 15-49 years | Polycystic ovarian syndrome | Rate | 2002 | 35.86370858 | 53.11773103 | 23.66120065 |
| Incidence | Eastern Sub-Saharan Africa | Female | 15-49 years | Polycystic ovarian syndrome | Rate | 2003 | 35.94050558 | 53.09414272 | 23.77022498 |
| Incidence | Eastern Sub-Saharan Africa | Female | 15-49 years | Polycystic ovarian syndrome | Rate | 2004 | 36.00814945 | 53.12052811 | 23.84459011 |
| Incidence | Eastern Sub-Saharan Africa | Female | 15-49 years | Polycystic ovarian syndrome | Rate | 2005 | 36.05994569 | 53.17118506 | 23.8592309 |
| Incidence | Eastern Sub-Saharan Africa | Female | 15-49 years | Polycystic ovarian syndrome | Rate | 2006 | 36.12448097 | 53.34049817 | 23.88193944 |
| Incidence | Eastern Sub-Saharan Africa | Female | 15-49 years | Polycystic ovarian syndrome | Rate | 2007 | 36.2289056 | 53.66023349 | 23.92009119 |
| Incidence | Eastern Sub-Saharan Africa | Female | 15-49 years | Polycystic ovarian syndrome | Rate | 2008 | 36.36558268 | 54.0488834 | 23.97641541 |
| Incidence | Eastern Sub-Saharan Africa | Female | 15-49 years | Polycystic ovarian syndrome | Rate | 2009 | 36.5173736 | 54.39778908 | 24.08020692 |
| Incidence | Eastern Sub-Saharan Africa | Female | 15-49 years | Polycystic ovarian syndrome | Rate | 2010 | 36.6741184 | 54.72035811 | 24.14911633 |
| Incidence | Eastern Sub-Saharan Africa | Female | 15-49 years | Polycystic ovarian syndrome | Rate | 2011 | 36.87236378 | 54.98435852 | 24.26490494 |
| Incidence | Eastern Sub-Saharan Africa | Female | 15-49 years | Polycystic ovarian syndrome | Rate | 2012 | 37.11799898 | 55.17323797 | 24.42531027 |
| Incidence | Eastern Sub-Saharan Africa | Female | 15-49 years | Polycystic ovarian syndrome | Rate | 2013 | 37.37067026 | 55.58459341 | 24.56759306 |
| Incidence | Eastern Sub-Saharan Africa | Female | 15-49 years | Polycystic ovarian syndrome | Rate | 2014 | 37.62058156 | 56.17348049 | 24.73009079 |
| Incidence | Eastern Sub-Saharan Africa | Female | 15-49 years | Polycystic ovarian syndrome | Rate | 2015 | 37.85900383 | 56.63329036 | 24.90450483 |
| Incidence | Eastern Sub-Saharan Africa | Female | 15-49 years | Polycystic ovarian syndrome | Rate | 2016 | 38.07418607 | 56.7129052 | 25.0103799 |
| Incidence | Eastern Sub-Saharan Africa | Female | 15-49 years | Polycystic ovarian syndrome | Rate | 2017 | 38.29713284 | 56.78842138 | 25.11346483 |
| Incidence | Eastern Sub-Saharan Africa | Female | 15-49 years | Polycystic ovarian syndrome | Rate | 2018 | 38.53956462 | 57.14569793 | 25.23423929 |
| Incidence | Eastern Sub-Saharan Africa | Female | 15-49 years | Polycystic ovarian syndrome | Rate | 2019 | 38.79814103 | 57.62899278 | 25.38099992 |
| Incidence | Eastern Sub-Saharan Africa | Female | 15-49 years | Polycystic ovarian syndrome | Rate | 2020 | 39.30355425 | 58.03235758 | 25.64529126 |
| Incidence | Eastern Sub-Saharan Africa | Female | 15-49 years | Polycystic ovarian syndrome | Rate | 2021 | 38.92834962 | 58.04716059 | 25.40402561 |
