# Supplementary material for: Global and Chinese epidemiologic study of polycystic ovary syndrome in women of childbearing age, 1990–2021, and projections to 2035: Based on the Global Burden of Disease 2021 study
Source: PLoS One. 2025 Aug 19;20(8):e0329090. doi: 10.1371/journal.pone.0329090 (PMC12364318; doi:10.1371/journal.pone.0329090)
Supplement: S2 Table — (DOCX) [file pone.0329090.s002.docx]

| **Supplementary Table 2** Global and Regional Prevalence of Polycystic Ovarian Syndrome Among Women Aged 15-49 Years: A Comprehensive Analysis from 1990 to 2021 | | | | | | | | | |
| --- | --- | --- | --- | --- | --- | --- | --- | --- | --- |
| **Measure** | **Location** | **Sex** | **Age** | **Cause** | **Metric** | **Year** | **Value** | **Upper** | **Lower** |
| Prevalence | Global | Female | 15-49 years | Polycystic ovarian syndrome | Rate | 1990 | 2602.624529 | 3583.124704 | 1864.268585 |
| Prevalence | Global | Female | 15-49 years | Polycystic ovarian syndrome | Rate | 1991 | 2627.369145 | 3613.316279 | 1881.907758 |
| Prevalence | Global | Female | 15-49 years | Polycystic ovarian syndrome | Rate | 1992 | 2653.007084 | 3645.377271 | 1899.76936 |
| Prevalence | Global | Female | 15-49 years | Polycystic ovarian syndrome | Rate | 1993 | 2677.812628 | 3677.808505 | 1919.214206 |
| Prevalence | Global | Female | 15-49 years | Polycystic ovarian syndrome | Rate | 1994 | 2701.174721 | 3708.198489 | 1937.430765 |
| Prevalence | Global | Female | 15-49 years | Polycystic ovarian syndrome | Rate | 1995 | 2723.843062 | 3737.513642 | 1954.509167 |
| Prevalence | Global | Female | 15-49 years | Polycystic ovarian syndrome | Rate | 1996 | 2751.279909 | 3764.239951 | 1976.686793 |
| Prevalence | Global | Female | 15-49 years | Polycystic ovarian syndrome | Rate | 1997 | 2783.571555 | 3797.601808 | 2002.921464 |
| Prevalence | Global | Female | 15-49 years | Polycystic ovarian syndrome | Rate | 1998 | 2817.01057 | 3833.2886 | 2026.360344 |
| Prevalence | Global | Female | 15-49 years | Polycystic ovarian syndrome | Rate | 1999 | 2847.768863 | 3865.822879 | 2052.784161 |
| Prevalence | Global | Female | 15-49 years | Polycystic ovarian syndrome | Rate | 2000 | 2869.698474 | 3893.610986 | 2072.701019 |
| Prevalence | Global | Female | 15-49 years | Polycystic ovarian syndrome | Rate | 2001 | 2888.248628 | 3917.169515 | 2093.620147 |
| Prevalence | Global | Female | 15-49 years | Polycystic ovarian syndrome | Rate | 2002 | 2907.518356 | 3941.828945 | 2114.490087 |
| Prevalence | Global | Female | 15-49 years | Polycystic ovarian syndrome | Rate | 2003 | 2926.465758 | 3966.538746 | 2134.22108 |
| Prevalence | Global | Female | 15-49 years | Polycystic ovarian syndrome | Rate | 2004 | 2944.123607 | 3990.23632 | 2149.290541 |
| Prevalence | Global | Female | 15-49 years | Polycystic ovarian syndrome | Rate | 2005 | 2958.511839 | 4013.529997 | 2155.565851 |
| Prevalence | Global | Female | 15-49 years | Polycystic ovarian syndrome | Rate | 2006 | 2968.224051 | 4021.542557 | 2169.203555 |
| Prevalence | Global | Female | 15-49 years | Polycystic ovarian syndrome | Rate | 2007 | 2975.033348 | 4026.222028 | 2180.837183 |
| Prevalence | Global | Female | 15-49 years | Polycystic ovarian syndrome | Rate | 2008 | 2982.743803 | 4036.338967 | 2191.365452 |
| Prevalence | Global | Female | 15-49 years | Polycystic ovarian syndrome | Rate | 2009 | 2994.993788 | 4056.153959 | 2201.913567 |
| Prevalence | Global | Female | 15-49 years | Polycystic ovarian syndrome | Rate | 2010 | 3013.924553 | 4086.300012 | 2216.035313 |
| Prevalence | Global | Female | 15-49 years | Polycystic ovarian syndrome | Rate | 2011 | 3040.426606 | 4125.321047 | 2230.539375 |
| Prevalence | Global | Female | 15-49 years | Polycystic ovarian syndrome | Rate | 2012 | 3071.541878 | 4171.934966 | 2248.217528 |
| Prevalence | Global | Female | 15-49 years | Polycystic ovarian syndrome | Rate | 2013 | 3104.470258 | 4221.939112 | 2267.203726 |
| Prevalence | Global | Female | 15-49 years | Polycystic ovarian syndrome | Rate | 2014 | 3135.770647 | 4271.824988 | 2285.11398 |
| Prevalence | Global | Female | 15-49 years | Polycystic ovarian syndrome | Rate | 2015 | 3162.108396 | 4316.013076 | 2299.51069 |
| Prevalence | Global | Female | 15-49 years | Polycystic ovarian syndrome | Rate | 2016 | 3192.57049 | 4360.431234 | 2320.136126 |
| Prevalence | Global | Female | 15-49 years | Polycystic ovarian syndrome | Rate | 2017 | 3231.34732 | 4414.665518 | 2345.495428 |
| Prevalence | Global | Female | 15-49 years | Polycystic ovarian syndrome | Rate | 2018 | 3272.345129 | 4474.687476 | 2369.367482 |
| Prevalence | Global | Female | 15-49 years | Polycystic ovarian syndrome | Rate | 2019 | 3309.061856 | 4534.083517 | 2391.827651 |
| Prevalence | Global | Female | 15-49 years | Polycystic ovarian syndrome | Rate | 2020 | 3355.027665 | 4601.581705 | 2410.961793 |
| Prevalence | Global | Female | 15-49 years | Polycystic ovarian syndrome | Rate | 2021 | 3374.676019 | 4649.681056 | 2394.973206 |
| Prevalence | Central Europe | Female | 15-49 years | Polycystic ovarian syndrome | Rate | 1990 | 355.0326141 | 530.7226664 | 235.4152348 |
| Prevalence | Central Europe | Female | 15-49 years | Polycystic ovarian syndrome | Rate | 1991 | 357.8754831 | 536.1839949 | 236.4793317 |
| Prevalence | Central Europe | Female | 15-49 years | Polycystic ovarian syndrome | Rate | 1992 | 360.6169003 | 540.4372628 | 237.695935 |
| Prevalence | Central Europe | Female | 15-49 years | Polycystic ovarian syndrome | Rate | 1993 | 363.4010622 | 543.692077 | 239.7272384 |
| Prevalence | Central Europe | Female | 15-49 years | Polycystic ovarian syndrome | Rate | 1994 | 366.3960734 | 547.2588447 | 241.4568265 |
| Prevalence | Central Europe | Female | 15-49 years | Polycystic ovarian syndrome | Rate | 1995 | 369.376076 | 551.0589058 | 242.8922539 |
| Prevalence | Central Europe | Female | 15-49 years | Polycystic ovarian syndrome | Rate | 1996 | 372.6065582 | 556.6031175 | 244.2426612 |
| Prevalence | Central Europe | Female | 15-49 years | Polycystic ovarian syndrome | Rate | 1997 | 376.5752366 | 563.790246 | 246.6435375 |
| Prevalence | Central Europe | Female | 15-49 years | Polycystic ovarian syndrome | Rate | 1998 | 380.6321266 | 571.2099745 | 249.2167191 |
| Prevalence | Central Europe | Female | 15-49 years | Polycystic ovarian syndrome | Rate | 1999 | 384.4705697 | 577.8595215 | 252.332961 |
| Prevalence | Central Europe | Female | 15-49 years | Polycystic ovarian syndrome | Rate | 2000 | 387.7600702 | 582.9324768 | 255.0675531 |
| Prevalence | Central Europe | Female | 15-49 years | Polycystic ovarian syndrome | Rate | 2001 | 390.6862015 | 587.0577754 | 256.5304108 |
| Prevalence | Central Europe | Female | 15-49 years | Polycystic ovarian syndrome | Rate | 2002 | 393.6905561 | 591.6002817 | 258.106675 |
| Prevalence | Central Europe | Female | 15-49 years | Polycystic ovarian syndrome | Rate | 2003 | 396.8212017 | 596.5013663 | 259.8546055 |
| Prevalence | Central Europe | Female | 15-49 years | Polycystic ovarian syndrome | Rate | 2004 | 399.9290567 | 601.2306681 | 262.1280175 |
| Prevalence | Central Europe | Female | 15-49 years | Polycystic ovarian syndrome | Rate | 2005 | 403.0957371 | 604.7784002 | 264.4901899 |
| Prevalence | Central Europe | Female | 15-49 years | Polycystic ovarian syndrome | Rate | 2006 | 406.465146 | 608.8564906 | 267.4802 |
| Prevalence | Central Europe | Female | 15-49 years | Polycystic ovarian syndrome | Rate | 2007 | 409.9316294 | 611.2359825 | 270.9341844 |
| Prevalence | Central Europe | Female | 15-49 years | Polycystic ovarian syndrome | Rate | 2008 | 413.1309625 | 608.7079085 | 274.3029327 |
| Prevalence | Central Europe | Female | 15-49 years | Polycystic ovarian syndrome | Rate | 2009 | 415.8419701 | 607.6413276 | 278.2655061 |
| Prevalence | Central Europe | Female | 15-49 years | Polycystic ovarian syndrome | Rate | 2010 | 417.8627354 | 609.6788086 | 281.7533043 |
| Prevalence | Central Europe | Female | 15-49 years | Polycystic ovarian syndrome | Rate | 2011 | 418.9113759 | 605.6177948 | 283.4525621 |
| Prevalence | Central Europe | Female | 15-49 years | Polycystic ovarian syndrome | Rate | 2012 | 419.1053368 | 598.5433508 | 285.436717 |
| Prevalence | Central Europe | Female | 15-49 years | Polycystic ovarian syndrome | Rate | 2013 | 418.787481 | 590.7173973 | 287.6732752 |
| Prevalence | Central Europe | Female | 15-49 years | Polycystic ovarian syndrome | Rate | 2014 | 418.7508464 | 587.3969286 | 290.0093937 |
| Prevalence | Central Europe | Female | 15-49 years | Polycystic ovarian syndrome | Rate | 2015 | 419.5165523 | 586.1389475 | 292.2158579 |
| Prevalence | Central Europe | Female | 15-49 years | Polycystic ovarian syndrome | Rate | 2016 | 421.2972629 | 587.1497936 | 293.7140389 |
| Prevalence | Central Europe | Female | 15-49 years | Polycystic ovarian syndrome | Rate | 2017 | 423.7587552 | 588.9825948 | 295.1552943 |
| Prevalence | Central Europe | Female | 15-49 years | Polycystic ovarian syndrome | Rate | 2018 | 426.6111764 | 592.0033557 | 297.5841806 |
| Prevalence | Central Europe | Female | 15-49 years | Polycystic ovarian syndrome | Rate | 2019 | 429.0817707 | 595.1351014 | 299.0659926 |
| Prevalence | Central Europe | Female | 15-49 years | Polycystic ovarian syndrome | Rate | 2020 | 432.5172246 | 597.9292527 | 302.5664989 |
| Prevalence | Central Europe | Female | 15-49 years | Polycystic ovarian syndrome | Rate | 2021 | 436.2517159 | 615.300946 | 297.9052519 |
| Prevalence | Eastern Europe | Female | 15-49 years | Polycystic ovarian syndrome | Rate | 1990 | 401.469133 | 580.7255882 | 272.3678289 |
| Prevalence | Eastern Europe | Female | 15-49 years | Polycystic ovarian syndrome | Rate | 1991 | 403.5339068 | 582.1020543 | 273.3947325 |
| Prevalence | Eastern Europe | Female | 15-49 years | Polycystic ovarian syndrome | Rate | 1992 | 405.1481954 | 584.4224975 | 274.1902839 |
| Prevalence | Eastern Europe | Female | 15-49 years | Polycystic ovarian syndrome | Rate | 1993 | 406.6107466 | 586.2473307 | 274.9667877 |
| Prevalence | Eastern Europe | Female | 15-49 years | Polycystic ovarian syndrome | Rate | 1994 | 407.8209437 | 587.4552612 | 275.9745454 |
| Prevalence | Eastern Europe | Female | 15-49 years | Polycystic ovarian syndrome | Rate | 1995 | 408.8700231 | 588.1015793 | 277.250315 |
| Prevalence | Eastern Europe | Female | 15-49 years | Polycystic ovarian syndrome | Rate | 1996 | 410.4330601 | 589.8669028 | 278.7737199 |
| Prevalence | Eastern Europe | Female | 15-49 years | Polycystic ovarian syndrome | Rate | 1997 | 412.5897313 | 592.5569993 | 280.6488824 |
| Prevalence | Eastern Europe | Female | 15-49 years | Polycystic ovarian syndrome | Rate | 1998 | 414.9701104 | 595.5990564 | 283.343688 |
| Prevalence | Eastern Europe | Female | 15-49 years | Polycystic ovarian syndrome | Rate | 1999 | 417.4880407 | 598.8901698 | 286.0107865 |
| Prevalence | Eastern Europe | Female | 15-49 years | Polycystic ovarian syndrome | Rate | 2000 | 419.9089235 | 602.0363665 | 287.7891129 |
| Prevalence | Eastern Europe | Female | 15-49 years | Polycystic ovarian syndrome | Rate | 2001 | 422.5877053 | 605.5817449 | 289.1394023 |
| Prevalence | Eastern Europe | Female | 15-49 years | Polycystic ovarian syndrome | Rate | 2002 | 426.0695048 | 610.6032109 | 291.0156537 |
| Prevalence | Eastern Europe | Female | 15-49 years | Polycystic ovarian syndrome | Rate | 2003 | 430.2537832 | 616.7816262 | 293.9584815 |
| Prevalence | Eastern Europe | Female | 15-49 years | Polycystic ovarian syndrome | Rate | 2004 | 434.9788242 | 623.7467626 | 297.0540229 |
| Prevalence | Eastern Europe | Female | 15-49 years | Polycystic ovarian syndrome | Rate | 2005 | 440.0648771 | 631.6380037 | 300.1474304 |
| Prevalence | Eastern Europe | Female | 15-49 years | Polycystic ovarian syndrome | Rate | 2006 | 446.2734771 | 639.4178285 | 305.7581118 |
| Prevalence | Eastern Europe | Female | 15-49 years | Polycystic ovarian syndrome | Rate | 2007 | 453.9861036 | 650.776539 | 311.8082682 |
| Prevalence | Eastern Europe | Female | 15-49 years | Polycystic ovarian syndrome | Rate | 2008 | 462.4955362 | 663.3024554 | 318.4373113 |
| Prevalence | Eastern Europe | Female | 15-49 years | Polycystic ovarian syndrome | Rate | 2009 | 470.9779959 | 675.9060849 | 324.9858014 |
| Prevalence | Eastern Europe | Female | 15-49 years | Polycystic ovarian syndrome | Rate | 2010 | 478.6693177 | 687.1634337 | 330.4524153 |
| Prevalence | Eastern Europe | Female | 15-49 years | Polycystic ovarian syndrome | Rate | 2011 | 485.4863534 | 697.0732914 | 335.4455986 |
| Prevalence | Eastern Europe | Female | 15-49 years | Polycystic ovarian syndrome | Rate | 2012 | 491.8954625 | 706.4342859 | 340.515516 |
| Prevalence | Eastern Europe | Female | 15-49 years | Polycystic ovarian syndrome | Rate | 2013 | 497.8998873 | 717.0702735 | 344.7311998 |
| Prevalence | Eastern Europe | Female | 15-49 years | Polycystic ovarian syndrome | Rate | 2014 | 503.5511766 | 726.978256 | 348.6777751 |
| Prevalence | Eastern Europe | Female | 15-49 years | Polycystic ovarian syndrome | Rate | 2015 | 508.8554599 | 735.6043217 | 352.1481538 |
| Prevalence | Eastern Europe | Female | 15-49 years | Polycystic ovarian syndrome | Rate | 2016 | 514.6590014 | 742.6250465 | 357.7398422 |
| Prevalence | Eastern Europe | Female | 15-49 years | Polycystic ovarian syndrome | Rate | 2017 | 520.851284 | 750.2475722 | 363.5231766 |
| Prevalence | Eastern Europe | Female | 15-49 years | Polycystic ovarian syndrome | Rate | 2018 | 526.1793349 | 757.2501089 | 368.6636462 |
| Prevalence | Eastern Europe | Female | 15-49 years | Polycystic ovarian syndrome | Rate | 2019 | 529.3164672 | 762.1063531 | 371.8277574 |
| Prevalence | Eastern Europe | Female | 15-49 years | Polycystic ovarian syndrome | Rate | 2020 | 529.391965 | 761.3002534 | 371.2347845 |
| Prevalence | Eastern Europe | Female | 15-49 years | Polycystic ovarian syndrome | Rate | 2021 | 520.3073032 | 752.896939 | 363.7259279 |
| Prevalence | East Asia | Female | 15-49 years | Polycystic ovarian syndrome | Rate | 1990 | 1615.559458 | 2276.319317 | 1131.483187 |
| Prevalence | East Asia | Female | 15-49 years | Polycystic ovarian syndrome | Rate | 1991 | 1631.640461 | 2298.254961 | 1144.645 |
| Prevalence | East Asia | Female | 15-49 years | Polycystic ovarian syndrome | Rate | 1992 | 1658.260775 | 2334.683026 | 1162.564739 |
| Prevalence | East Asia | Female | 15-49 years | Polycystic ovarian syndrome | Rate | 1993 | 1692.764202 | 2382.870367 | 1189.642503 |
| Prevalence | East Asia | Female | 15-49 years | Polycystic ovarian syndrome | Rate | 1994 | 1731.299736 | 2436.069885 | 1215.759483 |
| Prevalence | East Asia | Female | 15-49 years | Polycystic ovarian syndrome | Rate | 1995 | 1772.760419 | 2494.915673 | 1245.043164 |
| Prevalence | East Asia | Female | 15-49 years | Polycystic ovarian syndrome | Rate | 1996 | 1830.184593 | 2571.079347 | 1286.667299 |
| Prevalence | East Asia | Female | 15-49 years | Polycystic ovarian syndrome | Rate | 1997 | 1910.526903 | 2685.036039 | 1340.716888 |
| Prevalence | East Asia | Female | 15-49 years | Polycystic ovarian syndrome | Rate | 1998 | 1999.640983 | 2814.284611 | 1401.451261 |
| Prevalence | East Asia | Female | 15-49 years | Polycystic ovarian syndrome | Rate | 1999 | 2084.858983 | 2935.689582 | 1460.860075 |
| Prevalence | East Asia | Female | 15-49 years | Polycystic ovarian syndrome | Rate | 2000 | 2152.398722 | 3031.205059 | 1507.876006 |
| Prevalence | East Asia | Female | 15-49 years | Polycystic ovarian syndrome | Rate | 2001 | 2205.653952 | 3105.564502 | 1541.715592 |
| Prevalence | East Asia | Female | 15-49 years | Polycystic ovarian syndrome | Rate | 2002 | 2255.92357 | 3175.836075 | 1580.330333 |
| Prevalence | East Asia | Female | 15-49 years | Polycystic ovarian syndrome | Rate | 2003 | 2303.52984 | 3242.796458 | 1616.764359 |
| Prevalence | East Asia | Female | 15-49 years | Polycystic ovarian syndrome | Rate | 2004 | 2347.939736 | 3305.674986 | 1653.091344 |
| Prevalence | East Asia | Female | 15-49 years | Polycystic ovarian syndrome | Rate | 2005 | 2388.022603 | 3362.99998 | 1689.312173 |
| Prevalence | East Asia | Female | 15-49 years | Polycystic ovarian syndrome | Rate | 2006 | 2422.469693 | 3407.487007 | 1712.147855 |
| Prevalence | East Asia | Female | 15-49 years | Polycystic ovarian syndrome | Rate | 2007 | 2451.858884 | 3446.63002 | 1731.39972 |
| Prevalence | East Asia | Female | 15-49 years | Polycystic ovarian syndrome | Rate | 2008 | 2478.450613 | 3480.042859 | 1748.774672 |
| Prevalence | East Asia | Female | 15-49 years | Polycystic ovarian syndrome | Rate | 2009 | 2505.606291 | 3521.23218 | 1773.086904 |
| Prevalence | East Asia | Female | 15-49 years | Polycystic ovarian syndrome | Rate | 2010 | 2536.425386 | 3568.496798 | 1802.454862 |
| Prevalence | East Asia | Female | 15-49 years | Polycystic ovarian syndrome | Rate | 2011 | 2572.594845 | 3622.325471 | 1827.300384 |
| Prevalence | East Asia | Female | 15-49 years | Polycystic ovarian syndrome | Rate | 2012 | 2613.059397 | 3682.824354 | 1854.252644 |
| Prevalence | East Asia | Female | 15-49 years | Polycystic ovarian syndrome | Rate | 2013 | 2656.43456 | 3742.650459 | 1882.189348 |
| Prevalence | East Asia | Female | 15-49 years | Polycystic ovarian syndrome | Rate | 2014 | 2700.835394 | 3806.71356 | 1907.629714 |
| Prevalence | East Asia | Female | 15-49 years | Polycystic ovarian syndrome | Rate | 2015 | 2744.195783 | 3871.223266 | 1931.170522 |
| Prevalence | East Asia | Female | 15-49 years | Polycystic ovarian syndrome | Rate | 2016 | 2791.533349 | 3939.721307 | 1965.906212 |
| Prevalence | East Asia | Female | 15-49 years | Polycystic ovarian syndrome | Rate | 2017 | 2843.546858 | 4013.651337 | 2001.143207 |
| Prevalence | East Asia | Female | 15-49 years | Polycystic ovarian syndrome | Rate | 2018 | 2891.937976 | 4077.862556 | 2035.510192 |
| Prevalence | East Asia | Female | 15-49 years | Polycystic ovarian syndrome | Rate | 2019 | 2928.829026 | 4125.270969 | 2063.151686 |
| Prevalence | East Asia | Female | 15-49 years | Polycystic ovarian syndrome | Rate | 2020 | 2948.102428 | 4132.944782 | 2065.356252 |
| Prevalence | East Asia | Female | 15-49 years | Polycystic ovarian syndrome | Rate | 2021 | 2983.917535 | 4236.169024 | 2103.545364 |
| Prevalence | Oceania | Female | 15-49 years | Polycystic ovarian syndrome | Rate | 1990 | 2443.515139 | 3447.430894 | 1681.986536 |
| Prevalence | Oceania | Female | 15-49 years | Polycystic ovarian syndrome | Rate | 1991 | 2512.4315 | 3547.754094 | 1727.475566 |
| Prevalence | Oceania | Female | 15-49 years | Polycystic ovarian syndrome | Rate | 1992 | 2578.943988 | 3652.650701 | 1775.136312 |
| Prevalence | Oceania | Female | 15-49 years | Polycystic ovarian syndrome | Rate | 1993 | 2644.414098 | 3754.959409 | 1821.490629 |
| Prevalence | Oceania | Female | 15-49 years | Polycystic ovarian syndrome | Rate | 1994 | 2709.809073 | 3855.908756 | 1871.739545 |
| Prevalence | Oceania | Female | 15-49 years | Polycystic ovarian syndrome | Rate | 1995 | 2775.465662 | 3956.59085 | 1923.276625 |
| Prevalence | Oceania | Female | 15-49 years | Polycystic ovarian syndrome | Rate | 1996 | 2841.349516 | 4060.423973 | 1974.259155 |
| Prevalence | Oceania | Female | 15-49 years | Polycystic ovarian syndrome | Rate | 1997 | 2911.713655 | 4168.477901 | 2013.62156 |
| Prevalence | Oceania | Female | 15-49 years | Polycystic ovarian syndrome | Rate | 1998 | 2979.623175 | 4271.777302 | 2069.955726 |
| Prevalence | Oceania | Female | 15-49 years | Polycystic ovarian syndrome | Rate | 1999 | 3039.462572 | 4363.464619 | 2119.693356 |
| Prevalence | Oceania | Female | 15-49 years | Polycystic ovarian syndrome | Rate | 2000 | 3085.408475 | 4435.393198 | 2153.780588 |
| Prevalence | Oceania | Female | 15-49 years | Polycystic ovarian syndrome | Rate | 2001 | 3125.374441 | 4469.064002 | 2181.616932 |
| Prevalence | Oceania | Female | 15-49 years | Polycystic ovarian syndrome | Rate | 2002 | 3162.229155 | 4501.193758 | 2202.011537 |
| Prevalence | Oceania | Female | 15-49 years | Polycystic ovarian syndrome | Rate | 2003 | 3193.639681 | 4537.732159 | 2222.942241 |
| Prevalence | Oceania | Female | 15-49 years | Polycystic ovarian syndrome | Rate | 2004 | 3215.913575 | 4553.378665 | 2230.001156 |
| Prevalence | Oceania | Female | 15-49 years | Polycystic ovarian syndrome | Rate | 2005 | 3223.121796 | 4570.457733 | 2227.117916 |
| Prevalence | Oceania | Female | 15-49 years | Polycystic ovarian syndrome | Rate | 2006 | 3217.091044 | 4542.563831 | 2227.991094 |
| Prevalence | Oceania | Female | 15-49 years | Polycystic ovarian syndrome | Rate | 2007 | 3205.585832 | 4506.515599 | 2236.760981 |
| Prevalence | Oceania | Female | 15-49 years | Polycystic ovarian syndrome | Rate | 2008 | 3191.67883 | 4493.54198 | 2225.495553 |
| Prevalence | Oceania | Female | 15-49 years | Polycystic ovarian syndrome | Rate | 2009 | 3178.79873 | 4505.119 | 2217.67618 |
| Prevalence | Oceania | Female | 15-49 years | Polycystic ovarian syndrome | Rate | 2010 | 3170.570053 | 4490.535851 | 2220.720744 |
| Prevalence | Oceania | Female | 15-49 years | Polycystic ovarian syndrome | Rate | 2011 | 3171.231921 | 4493.975216 | 2215.798433 |
| Prevalence | Oceania | Female | 15-49 years | Polycystic ovarian syndrome | Rate | 2012 | 3180.688305 | 4510.627128 | 2220.571535 |
| Prevalence | Oceania | Female | 15-49 years | Polycystic ovarian syndrome | Rate | 2013 | 3195.92519 | 4535.220562 | 2235.686031 |
| Prevalence | Oceania | Female | 15-49 years | Polycystic ovarian syndrome | Rate | 2014 | 3213.788073 | 4563.563909 | 2244.636532 |
| Prevalence | Oceania | Female | 15-49 years | Polycystic ovarian syndrome | Rate | 2015 | 3231.167149 | 4592.152846 | 2239.322935 |
| Prevalence | Oceania | Female | 15-49 years | Polycystic ovarian syndrome | Rate | 2016 | 3253.66901 | 4633.131978 | 2251.436879 |
| Prevalence | Oceania | Female | 15-49 years | Polycystic ovarian syndrome | Rate | 2017 | 3283.119829 | 4660.630886 | 2261.716844 |
| Prevalence | Oceania | Female | 15-49 years | Polycystic ovarian syndrome | Rate | 2018 | 3310.45147 | 4673.662398 | 2291.296875 |
| Prevalence | Oceania | Female | 15-49 years | Polycystic ovarian syndrome | Rate | 2019 | 3326.293715 | 4680.745633 | 2317.614118 |
| Prevalence | Oceania | Female | 15-49 years | Polycystic ovarian syndrome | Rate | 2020 | 3324.156153 | 4771.262145 | 2286.754983 |
| Prevalence | Oceania | Female | 15-49 years | Polycystic ovarian syndrome | Rate | 2021 | 3393.429831 | 4846.834684 | 2360.221551 |
| Prevalence | Southeast Asia | Female | 15-49 years | Polycystic ovarian syndrome | Rate | 1990 | 2917.33419 | 4111.875571 | 2061.923924 |
| Prevalence | Southeast Asia | Female | 15-49 years | Polycystic ovarian syndrome | Rate | 1991 | 2948.938192 | 4165.619784 | 2082.632503 |
| Prevalence | Southeast Asia | Female | 15-49 years | Polycystic ovarian syndrome | Rate | 1992 | 2988.390701 | 4227.102766 | 2110.920642 |
| Prevalence | Southeast Asia | Female | 15-49 years | Polycystic ovarian syndrome | Rate | 1993 | 3034.197258 | 4292.465473 | 2146.412248 |
| Prevalence | Southeast Asia | Female | 15-49 years | Polycystic ovarian syndrome | Rate | 1994 | 3085.415075 | 4362.322475 | 2187.392781 |
| Prevalence | Southeast Asia | Female | 15-49 years | Polycystic ovarian syndrome | Rate | 1995 | 3141.400362 | 4430.361247 | 2228.347434 |
| Prevalence | Southeast Asia | Female | 15-49 years | Polycystic ovarian syndrome | Rate | 1996 | 3207.640498 | 4476.081913 | 2296.898272 |
| Prevalence | Southeast Asia | Female | 15-49 years | Polycystic ovarian syndrome | Rate | 1997 | 3288.656582 | 4531.456338 | 2377.754619 |
| Prevalence | Southeast Asia | Female | 15-49 years | Polycystic ovarian syndrome | Rate | 1998 | 3378.990353 | 4601.350658 | 2466.821989 |
| Prevalence | Southeast Asia | Female | 15-49 years | Polycystic ovarian syndrome | Rate | 1999 | 3473.088211 | 4682.875583 | 2560.547208 |
| Prevalence | Southeast Asia | Female | 15-49 years | Polycystic ovarian syndrome | Rate | 2000 | 3564.874717 | 4775.671616 | 2637.47104 |
| Prevalence | Southeast Asia | Female | 15-49 years | Polycystic ovarian syndrome | Rate | 2001 | 3666.182942 | 4915.182986 | 2709.043116 |
| Prevalence | Southeast Asia | Female | 15-49 years | Polycystic ovarian syndrome | Rate | 2002 | 3783.067977 | 5083.636899 | 2792.149338 |
| Prevalence | Southeast Asia | Female | 15-49 years | Polycystic ovarian syndrome | Rate | 2003 | 3906.773243 | 5265.95312 | 2876.154916 |
| Prevalence | Southeast Asia | Female | 15-49 years | Polycystic ovarian syndrome | Rate | 2004 | 4028.572822 | 5450.353034 | 2951.366885 |
| Prevalence | Southeast Asia | Female | 15-49 years | Polycystic ovarian syndrome | Rate | 2005 | 4139.867143 | 5624.562481 | 3012.939763 |
| Prevalence | Southeast Asia | Female | 15-49 years | Polycystic ovarian syndrome | Rate | 2006 | 4246.688877 | 5812.56228 | 3089.404291 |
| Prevalence | Southeast Asia | Female | 15-49 years | Polycystic ovarian syndrome | Rate | 2007 | 4358.060227 | 6013.998187 | 3156.149374 |
| Prevalence | Southeast Asia | Female | 15-49 years | Polycystic ovarian syndrome | Rate | 2008 | 4472.408322 | 6223.86866 | 3229.334681 |
| Prevalence | Southeast Asia | Female | 15-49 years | Polycystic ovarian syndrome | Rate | 2009 | 4585.969012 | 6435.768716 | 3303.857261 |
| Prevalence | Southeast Asia | Female | 15-49 years | Polycystic ovarian syndrome | Rate | 2010 | 4688.902801 | 6631.553968 | 3357.935589 |
| Prevalence | Southeast Asia | Female | 15-49 years | Polycystic ovarian syndrome | Rate | 2011 | 4787.456695 | 6784.846272 | 3424.485936 |
| Prevalence | Southeast Asia | Female | 15-49 years | Polycystic ovarian syndrome | Rate | 2012 | 4891.678218 | 6940.91206 | 3490.071842 |
| Prevalence | Southeast Asia | Female | 15-49 years | Polycystic ovarian syndrome | Rate | 2013 | 4994.335133 | 7092.593274 | 3552.935953 |
| Prevalence | Southeast Asia | Female | 15-49 years | Polycystic ovarian syndrome | Rate | 2014 | 5086.540116 | 7244.611783 | 3606.569797 |
| Prevalence | Southeast Asia | Female | 15-49 years | Polycystic ovarian syndrome | Rate | 2015 | 5161.04603 | 7372.840617 | 3647.4675 |
| Prevalence | Southeast Asia | Female | 15-49 years | Polycystic ovarian syndrome | Rate | 2016 | 5221.535846 | 7445.779877 | 3700.500728 |
| Prevalence | Southeast Asia | Female | 15-49 years | Polycystic ovarian syndrome | Rate | 2017 | 5277.86316 | 7509.280797 | 3740.560971 |
| Prevalence | Southeast Asia | Female | 15-49 years | Polycystic ovarian syndrome | Rate | 2018 | 5332.158824 | 7568.610193 | 3777.476821 |
| Prevalence | Southeast Asia | Female | 15-49 years | Polycystic ovarian syndrome | Rate | 2019 | 5386.431427 | 7621.55204 | 3809.783773 |
| Prevalence | Southeast Asia | Female | 15-49 years | Polycystic ovarian syndrome | Rate | 2020 | 5463.759744 | 7702.078305 | 3858.020708 |
| Prevalence | Southeast Asia | Female | 15-49 years | Polycystic ovarian syndrome | Rate | 2021 | 5457.54041 | 7695.675503 | 3851.149986 |
| Prevalence | Central Asia | Female | 15-49 years | Polycystic ovarian syndrome | Rate | 1990 | 667.5876036 | 973.1478064 | 448.7398851 |
| Prevalence | Central Asia | Female | 15-49 years | Polycystic ovarian syndrome | Rate | 1991 | 677.9633522 | 989.147392 | 455.9310847 |
| Prevalence | Central Asia | Female | 15-49 years | Polycystic ovarian syndrome | Rate | 1992 | 687.3017701 | 1004.075512 | 463.5278705 |
| Prevalence | Central Asia | Female | 15-49 years | Polycystic ovarian syndrome | Rate | 1993 | 695.4560672 | 1017.757318 | 469.3230034 |
| Prevalence | Central Asia | Female | 15-49 years | Polycystic ovarian syndrome | Rate | 1994 | 701.9414468 | 1029.568965 | 474.1761522 |
| Prevalence | Central Asia | Female | 15-49 years | Polycystic ovarian syndrome | Rate | 1995 | 706.5415757 | 1039.292411 | 475.3463966 |
| Prevalence | Central Asia | Female | 15-49 years | Polycystic ovarian syndrome | Rate | 1996 | 709.5001212 | 1037.156148 | 477.8076682 |
| Prevalence | Central Asia | Female | 15-49 years | Polycystic ovarian syndrome | Rate | 1997 | 712.391493 | 1035.513236 | 479.352474 |
| Prevalence | Central Asia | Female | 15-49 years | Polycystic ovarian syndrome | Rate | 1998 | 715.1571207 | 1035.962625 | 481.0320466 |
| Prevalence | Central Asia | Female | 15-49 years | Polycystic ovarian syndrome | Rate | 1999 | 717.9748434 | 1039.507961 | 483.5889366 |
| Prevalence | Central Asia | Female | 15-49 years | Polycystic ovarian syndrome | Rate | 2000 | 720.9806624 | 1043.447824 | 486.3093487 |
| Prevalence | Central Asia | Female | 15-49 years | Polycystic ovarian syndrome | Rate | 2001 | 725.4498137 | 1050.870086 | 488.891847 |
| Prevalence | Central Asia | Female | 15-49 years | Polycystic ovarian syndrome | Rate | 2002 | 731.4755365 | 1061.695812 | 491.4159003 |
| Prevalence | Central Asia | Female | 15-49 years | Polycystic ovarian syndrome | Rate | 2003 | 738.7218072 | 1072.587159 | 494.6323797 |
| Prevalence | Central Asia | Female | 15-49 years | Polycystic ovarian syndrome | Rate | 2004 | 746.719349 | 1078.364158 | 501.7966015 |
| Prevalence | Central Asia | Female | 15-49 years | Polycystic ovarian syndrome | Rate | 2005 | 755.1290467 | 1081.239145 | 508.9330852 |
| Prevalence | Central Asia | Female | 15-49 years | Polycystic ovarian syndrome | Rate | 2006 | 764.7349864 | 1100.711943 | 515.1048658 |
| Prevalence | Central Asia | Female | 15-49 years | Polycystic ovarian syndrome | Rate | 2007 | 776.4375298 | 1120.083634 | 521.8643063 |
| Prevalence | Central Asia | Female | 15-49 years | Polycystic ovarian syndrome | Rate | 2008 | 789.555038 | 1134.364834 | 530.2762272 |
| Prevalence | Central Asia | Female | 15-49 years | Polycystic ovarian syndrome | Rate | 2009 | 803.4532231 | 1150.702456 | 537.7004263 |
| Prevalence | Central Asia | Female | 15-49 years | Polycystic ovarian syndrome | Rate | 2010 | 817.5541228 | 1176.771809 | 546.9222676 |
| Prevalence | Central Asia | Female | 15-49 years | Polycystic ovarian syndrome | Rate | 2011 | 833.1417769 | 1194.210914 | 560.6438247 |
| Prevalence | Central Asia | Female | 15-49 years | Polycystic ovarian syndrome | Rate | 2012 | 850.6709982 | 1223.048188 | 576.9276638 |
| Prevalence | Central Asia | Female | 15-49 years | Polycystic ovarian syndrome | Rate | 2013 | 868.4699529 | 1253.598901 | 591.7799655 |
| Prevalence | Central Asia | Female | 15-49 years | Polycystic ovarian syndrome | Rate | 2014 | 884.9129041 | 1267.777071 | 604.0450668 |
| Prevalence | Central Asia | Female | 15-49 years | Polycystic ovarian syndrome | Rate | 2015 | 898.527536 | 1281.977405 | 613.8987885 |
| Prevalence | Central Asia | Female | 15-49 years | Polycystic ovarian syndrome | Rate | 2016 | 910.086162 | 1291.896758 | 618.0125712 |
| Prevalence | Central Asia | Female | 15-49 years | Polycystic ovarian syndrome | Rate | 2017 | 920.9521389 | 1311.757259 | 622.3292356 |
| Prevalence | Central Asia | Female | 15-49 years | Polycystic ovarian syndrome | Rate | 2018 | 930.433007 | 1325.241528 | 627.2979136 |
| Prevalence | Central Asia | Female | 15-49 years | Polycystic ovarian syndrome | Rate | 2019 | 937.9949029 | 1337.507331 | 630.8919127 |
| Prevalence | Central Asia | Female | 15-49 years | Polycystic ovarian syndrome | Rate | 2020 | 944.3110669 | 1338.453372 | 636.0853823 |
| Prevalence | Central Asia | Female | 15-49 years | Polycystic ovarian syndrome | Rate | 2021 | 929.2615275 | 1304.198314 | 639.6360995 |
| Prevalence | High-income Asia Pacific | Female | 15-49 years | Polycystic ovarian syndrome | Rate | 1990 | 9185.527576 | 12792.03475 | 6624.984825 |
| Prevalence | High-income Asia Pacific | Female | 15-49 years | Polycystic ovarian syndrome | Rate | 1991 | 9254.822108 | 12849.86786 | 6674.913398 |
| Prevalence | High-income Asia Pacific | Female | 15-49 years | Polycystic ovarian syndrome | Rate | 1992 | 9312.500223 | 12903.99211 | 6716.371952 |
| Prevalence | High-income Asia Pacific | Female | 15-49 years | Polycystic ovarian syndrome | Rate | 1993 | 9369.773171 | 12986.70622 | 6757.643009 |
| Prevalence | High-income Asia Pacific | Female | 15-49 years | Polycystic ovarian syndrome | Rate | 1994 | 9419.205741 | 13060.65142 | 6792.935747 |
| Prevalence | High-income Asia Pacific | Female | 15-49 years | Polycystic ovarian syndrome | Rate | 1995 | 9464.501742 | 13127.53237 | 6822.823507 |
| Prevalence | High-income Asia Pacific | Female | 15-49 years | Polycystic ovarian syndrome | Rate | 1996 | 9519.188839 | 13188.94359 | 6848.845234 |
| Prevalence | High-income Asia Pacific | Female | 15-49 years | Polycystic ovarian syndrome | Rate | 1997 | 9581.413453 | 13271.45982 | 6878.701868 |
| Prevalence | High-income Asia Pacific | Female | 15-49 years | Polycystic ovarian syndrome | Rate | 1998 | 9646.016959 | 13359.79998 | 6909.458353 |
| Prevalence | High-income Asia Pacific | Female | 15-49 years | Polycystic ovarian syndrome | Rate | 1999 | 9703.060125 | 13439.1948 | 6941.650289 |
| Prevalence | High-income Asia Pacific | Female | 15-49 years | Polycystic ovarian syndrome | Rate | 2000 | 9738.545263 | 13487.0351 | 6965.243463 |
| Prevalence | High-income Asia Pacific | Female | 15-49 years | Polycystic ovarian syndrome | Rate | 2001 | 9763.132026 | 13358.27068 | 7060.40649 |
| Prevalence | High-income Asia Pacific | Female | 15-49 years | Polycystic ovarian syndrome | Rate | 2002 | 9805.171452 | 13305.53804 | 7165.921174 |
| Prevalence | High-income Asia Pacific | Female | 15-49 years | Polycystic ovarian syndrome | Rate | 2003 | 9840.094376 | 13271.59255 | 7256.413235 |
| Prevalence | High-income Asia Pacific | Female | 15-49 years | Polycystic ovarian syndrome | Rate | 2004 | 9863.509401 | 13250.78697 | 7316.454212 |
| Prevalence | High-income Asia Pacific | Female | 15-49 years | Polycystic ovarian syndrome | Rate | 2005 | 9869.444323 | 13219.89811 | 7321.505191 |
| Prevalence | High-income Asia Pacific | Female | 15-49 years | Polycystic ovarian syndrome | Rate | 2006 | 9844.289362 | 13202.91234 | 7305.415297 |
| Prevalence | High-income Asia Pacific | Female | 15-49 years | Polycystic ovarian syndrome | Rate | 2007 | 9797.359188 | 13154.05133 | 7274.381529 |
| Prevalence | High-income Asia Pacific | Female | 15-49 years | Polycystic ovarian syndrome | Rate | 2008 | 9741.489952 | 13088.06711 | 7237.815703 |
| Prevalence | High-income Asia Pacific | Female | 15-49 years | Polycystic ovarian syndrome | Rate | 2009 | 9691.245813 | 13012.52948 | 7205.63799 |
| Prevalence | High-income Asia Pacific | Female | 15-49 years | Polycystic ovarian syndrome | Rate | 2010 | 9656.091978 | 12979.28833 | 7182.934756 |
| Prevalence | High-income Asia Pacific | Female | 15-49 years | Polycystic ovarian syndrome | Rate | 2011 | 9687.871494 | 13056.36026 | 7178.321849 |
| Prevalence | High-income Asia Pacific | Female | 15-49 years | Polycystic ovarian syndrome | Rate | 2012 | 9773.363727 | 13242.23066 | 7180.146837 |
| Prevalence | High-income Asia Pacific | Female | 15-49 years | Polycystic ovarian syndrome | Rate | 2013 | 9886.205439 | 13488.03648 | 7191.308242 |
| Prevalence | High-income Asia Pacific | Female | 15-49 years | Polycystic ovarian syndrome | Rate | 2014 | 9984.461621 | 13740.0596 | 7186.875813 |
| Prevalence | High-income Asia Pacific | Female | 15-49 years | Polycystic ovarian syndrome | Rate | 2015 | 10030.09358 | 13925.32223 | 7157.658133 |
| Prevalence | High-income Asia Pacific | Female | 15-49 years | Polycystic ovarian syndrome | Rate | 2016 | 10058.12876 | 14015.51175 | 7197.372024 |
| Prevalence | High-income Asia Pacific | Female | 15-49 years | Polycystic ovarian syndrome | Rate | 2017 | 10112.79385 | 14103.45983 | 7214.912426 |
| Prevalence | High-income Asia Pacific | Female | 15-49 years | Polycystic ovarian syndrome | Rate | 2018 | 10175.39112 | 14140.8738 | 7258.20867 |
| Prevalence | High-income Asia Pacific | Female | 15-49 years | Polycystic ovarian syndrome | Rate | 2019 | 10224.17772 | 14112.33234 | 7296.607926 |
| Prevalence | High-income Asia Pacific | Female | 15-49 years | Polycystic ovarian syndrome | Rate | 2020 | 10265.64783 | 14277.58853 | 7301.58083 |
| Prevalence | High-income Asia Pacific | Female | 15-49 years | Polycystic ovarian syndrome | Rate | 2021 | 10239.02236 | 14296.03177 | 7234.27661 |
| Prevalence | Western Europe | Female | 15-49 years | Polycystic ovarian syndrome | Rate | 1990 | 6758.258756 | 9390.762835 | 4758.235332 |
| Prevalence | Western Europe | Female | 15-49 years | Polycystic ovarian syndrome | Rate | 1991 | 6849.15767 | 9493.893152 | 4851.144 |
| Prevalence | Western Europe | Female | 15-49 years | Polycystic ovarian syndrome | Rate | 1992 | 6928.084197 | 9583.259083 | 4933.240296 |
| Prevalence | Western Europe | Female | 15-49 years | Polycystic ovarian syndrome | Rate | 1993 | 7000.913426 | 9665.770568 | 5004.103968 |
| Prevalence | Western Europe | Female | 15-49 years | Polycystic ovarian syndrome | Rate | 1994 | 7066.129115 | 9726.209915 | 5063.841454 |
| Prevalence | Western Europe | Female | 15-49 years | Polycystic ovarian syndrome | Rate | 1995 | 7120.581903 | 9744.17367 | 5112.77889 |
| Prevalence | Western Europe | Female | 15-49 years | Polycystic ovarian syndrome | Rate | 1996 | 7179.601718 | 9791.275736 | 5161.776402 |
| Prevalence | Western Europe | Female | 15-49 years | Polycystic ovarian syndrome | Rate | 1997 | 7248.02545 | 9866.391718 | 5228.096662 |
| Prevalence | Western Europe | Female | 15-49 years | Polycystic ovarian syndrome | Rate | 1998 | 7316.541681 | 9909.052568 | 5299.10836 |
| Prevalence | Western Europe | Female | 15-49 years | Polycystic ovarian syndrome | Rate | 1999 | 7374.828114 | 9967.920827 | 5346.248581 |
| Prevalence | Western Europe | Female | 15-49 years | Polycystic ovarian syndrome | Rate | 2000 | 7411.541405 | 10036.9398 | 5367.895905 |
| Prevalence | Western Europe | Female | 15-49 years | Polycystic ovarian syndrome | Rate | 2001 | 7422.528942 | 10046.93025 | 5365.082755 |
| Prevalence | Western Europe | Female | 15-49 years | Polycystic ovarian syndrome | Rate | 2002 | 7418.145817 | 10080.53036 | 5351.003986 |
| Prevalence | Western Europe | Female | 15-49 years | Polycystic ovarian syndrome | Rate | 2003 | 7403.322371 | 10118.57846 | 5324.692486 |
| Prevalence | Western Europe | Female | 15-49 years | Polycystic ovarian syndrome | Rate | 2004 | 7383.807205 | 10155.95567 | 5301.969459 |
| Prevalence | Western Europe | Female | 15-49 years | Polycystic ovarian syndrome | Rate | 2005 | 7360.348322 | 10184.7608 | 5260.044954 |
| Prevalence | Western Europe | Female | 15-49 years | Polycystic ovarian syndrome | Rate | 2006 | 7333.497165 | 10128.9122 | 5250.634611 |
| Prevalence | Western Europe | Female | 15-49 years | Polycystic ovarian syndrome | Rate | 2007 | 7302.513101 | 10065.48789 | 5229.594852 |
| Prevalence | Western Europe | Female | 15-49 years | Polycystic ovarian syndrome | Rate | 2008 | 7275.88392 | 10010.13419 | 5211.70074 |
| Prevalence | Western Europe | Female | 15-49 years | Polycystic ovarian syndrome | Rate | 2009 | 7259.639071 | 9971.882426 | 5196.208646 |
| Prevalence | Western Europe | Female | 15-49 years | Polycystic ovarian syndrome | Rate | 2010 | 7258.608269 | 9958.42168 | 5182.146477 |
| Prevalence | Western Europe | Female | 15-49 years | Polycystic ovarian syndrome | Rate | 2011 | 7272.962282 | 10020.33797 | 5201.115028 |
| Prevalence | Western Europe | Female | 15-49 years | Polycystic ovarian syndrome | Rate | 2012 | 7293.328808 | 10098.32009 | 5216.350212 |
| Prevalence | Western Europe | Female | 15-49 years | Polycystic ovarian syndrome | Rate | 2013 | 7316.653846 | 10181.01839 | 5218.451195 |
| Prevalence | Western Europe | Female | 15-49 years | Polycystic ovarian syndrome | Rate | 2014 | 7337.885599 | 10225.57227 | 5213.832012 |
| Prevalence | Western Europe | Female | 15-49 years | Polycystic ovarian syndrome | Rate | 2015 | 7350.622847 | 10245.0944 | 5215.853347 |
| Prevalence | Western Europe | Female | 15-49 years | Polycystic ovarian syndrome | Rate | 2016 | 7369.920803 | 10296.42025 | 5235.475853 |
| Prevalence | Western Europe | Female | 15-49 years | Polycystic ovarian syndrome | Rate | 2017 | 7401.467854 | 10360.87662 | 5259.019732 |
| Prevalence | Western Europe | Female | 15-49 years | Polycystic ovarian syndrome | Rate | 2018 | 7433.438897 | 10427.38069 | 5254.107111 |
| Prevalence | Western Europe | Female | 15-49 years | Polycystic ovarian syndrome | Rate | 2019 | 7452.124038 | 10459.91396 | 5245.061653 |
| Prevalence | Western Europe | Female | 15-49 years | Polycystic ovarian syndrome | Rate | 2020 | 7487.945698 | 10404.47055 | 5268.599988 |
| Prevalence | Western Europe | Female | 15-49 years | Polycystic ovarian syndrome | Rate | 2021 | 7518.708157 | 10491.11599 | 5296.660667 |
| Prevalence | Australasia | Female | 15-49 years | Polycystic ovarian syndrome | Rate | 1990 | 7918.642708 | 10416.61487 | 5832.169803 |
| Prevalence | Australasia | Female | 15-49 years | Polycystic ovarian syndrome | Rate | 1991 | 8071.564168 | 10666.63172 | 5938.43761 |
| Prevalence | Australasia | Female | 15-49 years | Polycystic ovarian syndrome | Rate | 1992 | 8211.150819 | 10841.47456 | 6035.009042 |
| Prevalence | Australasia | Female | 15-49 years | Polycystic ovarian syndrome | Rate | 1993 | 8339.673009 | 11003.40958 | 6117.269005 |
| Prevalence | Australasia | Female | 15-49 years | Polycystic ovarian syndrome | Rate | 1994 | 8457.0788 | 11195.97902 | 6202.86863 |
| Prevalence | Australasia | Female | 15-49 years | Polycystic ovarian syndrome | Rate | 1995 | 8558.565305 | 11279.91224 | 6273.312155 |
| Prevalence | Australasia | Female | 15-49 years | Polycystic ovarian syndrome | Rate | 1996 | 8649.746093 | 11398.14179 | 6416.488072 |
| Prevalence | Australasia | Female | 15-49 years | Polycystic ovarian syndrome | Rate | 1997 | 8741.322915 | 11397.56226 | 6561.529755 |
| Prevalence | Australasia | Female | 15-49 years | Polycystic ovarian syndrome | Rate | 1998 | 8817.521761 | 11360.79084 | 6675.349363 |
| Prevalence | Australasia | Female | 15-49 years | Polycystic ovarian syndrome | Rate | 1999 | 8876.840768 | 11297.65288 | 6738.392505 |
| Prevalence | Australasia | Female | 15-49 years | Polycystic ovarian syndrome | Rate | 2000 | 8915.505916 | 11347.47177 | 6820.643475 |
| Prevalence | Australasia | Female | 15-49 years | Polycystic ovarian syndrome | Rate | 2001 | 8934.801695 | 11089.31538 | 7017.321174 |
| Prevalence | Australasia | Female | 15-49 years | Polycystic ovarian syndrome | Rate | 2002 | 8943.560645 | 10823.5694 | 7274.44917 |
| Prevalence | Australasia | Female | 15-49 years | Polycystic ovarian syndrome | Rate | 2003 | 8945.325977 | 10639.09795 | 7464.035823 |
| Prevalence | Australasia | Female | 15-49 years | Polycystic ovarian syndrome | Rate | 2004 | 8937.033343 | 10477.7961 | 7578.977819 |
| Prevalence | Australasia | Female | 15-49 years | Polycystic ovarian syndrome | Rate | 2005 | 8922.696592 | 10355.52745 | 7681.592111 |
| Prevalence | Australasia | Female | 15-49 years | Polycystic ovarian syndrome | Rate | 2006 | 8903.053584 | 10322.06548 | 7661.48795 |
| Prevalence | Australasia | Female | 15-49 years | Polycystic ovarian syndrome | Rate | 2007 | 8879.61432 | 10279.71178 | 7612.520972 |
| Prevalence | Australasia | Female | 15-49 years | Polycystic ovarian syndrome | Rate | 2008 | 8857.429737 | 10276.17419 | 7544.22911 |
| Prevalence | Australasia | Female | 15-49 years | Polycystic ovarian syndrome | Rate | 2009 | 8840.513722 | 10300.09528 | 7409.279695 |
| Prevalence | Australasia | Female | 15-49 years | Polycystic ovarian syndrome | Rate | 2010 | 8833.03751 | 10341.08416 | 7350.158653 |
| Prevalence | Australasia | Female | 15-49 years | Polycystic ovarian syndrome | Rate | 2011 | 8827.241741 | 10497.33686 | 7209.2268 |
| Prevalence | Australasia | Female | 15-49 years | Polycystic ovarian syndrome | Rate | 2012 | 8816.09315 | 10771.43754 | 7133.248947 |
| Prevalence | Australasia | Female | 15-49 years | Polycystic ovarian syndrome | Rate | 2013 | 8807.267968 | 11241.17919 | 6865.512784 |
| Prevalence | Australasia | Female | 15-49 years | Polycystic ovarian syndrome | Rate | 2014 | 8806.506208 | 11637.55175 | 6622.76138 |
| Prevalence | Australasia | Female | 15-49 years | Polycystic ovarian syndrome | Rate | 2015 | 8821.026069 | 12118.50775 | 6390.626633 |
| Prevalence | Australasia | Female | 15-49 years | Polycystic ovarian syndrome | Rate | 2016 | 8860.506931 | 12143.80209 | 6389.441912 |
| Prevalence | Australasia | Female | 15-49 years | Polycystic ovarian syndrome | Rate | 2017 | 8924.356538 | 12242.41373 | 6402.315891 |
| Prevalence | Australasia | Female | 15-49 years | Polycystic ovarian syndrome | Rate | 2018 | 8993.597527 | 12410.39926 | 6425.773877 |
| Prevalence | Australasia | Female | 15-49 years | Polycystic ovarian syndrome | Rate | 2019 | 9050.39159 | 12627.56935 | 6431.185986 |
| Prevalence | Australasia | Female | 15-49 years | Polycystic ovarian syndrome | Rate | 2020 | 9161.929689 | 12794.52201 | 6612.503455 |
| Prevalence | Australasia | Female | 15-49 years | Polycystic ovarian syndrome | Rate | 2021 | 9213.662096 | 12814.35244 | 6561.161207 |
| Prevalence | Caribbean | Female | 15-49 years | Polycystic ovarian syndrome | Rate | 1990 | 2257.795168 | 3247.57356 | 1519.06632 |
| Prevalence | Caribbean | Female | 15-49 years | Polycystic ovarian syndrome | Rate | 1991 | 2286.330139 | 3276.942133 | 1547.38187 |
| Prevalence | Caribbean | Female | 15-49 years | Polycystic ovarian syndrome | Rate | 1992 | 2313.916086 | 3305.830194 | 1569.591941 |
| Prevalence | Caribbean | Female | 15-49 years | Polycystic ovarian syndrome | Rate | 1993 | 2339.359367 | 3335.828367 | 1587.522854 |
| Prevalence | Caribbean | Female | 15-49 years | Polycystic ovarian syndrome | Rate | 1994 | 2362.356023 | 3364.040123 | 1611.806419 |
| Prevalence | Caribbean | Female | 15-49 years | Polycystic ovarian syndrome | Rate | 1995 | 2382.508304 | 3389.062326 | 1631.375818 |
| Prevalence | Caribbean | Female | 15-49 years | Polycystic ovarian syndrome | Rate | 1996 | 2400.286225 | 3419.354576 | 1650.507454 |
| Prevalence | Caribbean | Female | 15-49 years | Polycystic ovarian syndrome | Rate | 1997 | 2417.459223 | 3450.523614 | 1654.226018 |
| Prevalence | Caribbean | Female | 15-49 years | Polycystic ovarian syndrome | Rate | 1998 | 2434.212993 | 3493.281171 | 1661.852646 |
| Prevalence | Caribbean | Female | 15-49 years | Polycystic ovarian syndrome | Rate | 1999 | 2451.061273 | 3529.884131 | 1671.796858 |
| Prevalence | Caribbean | Female | 15-49 years | Polycystic ovarian syndrome | Rate | 2000 | 2468.691782 | 3567.149078 | 1691.536059 |
| Prevalence | Caribbean | Female | 15-49 years | Polycystic ovarian syndrome | Rate | 2001 | 2493.601156 | 3607.77859 | 1702.562599 |
| Prevalence | Caribbean | Female | 15-49 years | Polycystic ovarian syndrome | Rate | 2002 | 2527.120027 | 3659.81002 | 1720.005705 |
| Prevalence | Caribbean | Female | 15-49 years | Polycystic ovarian syndrome | Rate | 2003 | 2564.124334 | 3717.411965 | 1745.921458 |
| Prevalence | Caribbean | Female | 15-49 years | Polycystic ovarian syndrome | Rate | 2004 | 2599.424184 | 3764.17977 | 1773.866871 |
| Prevalence | Caribbean | Female | 15-49 years | Polycystic ovarian syndrome | Rate | 2005 | 2627.781492 | 3800.881345 | 1800.898206 |
| Prevalence | Caribbean | Female | 15-49 years | Polycystic ovarian syndrome | Rate | 2006 | 2651.799988 | 3847.613599 | 1814.473529 |
| Prevalence | Caribbean | Female | 15-49 years | Polycystic ovarian syndrome | Rate | 2007 | 2676.027239 | 3873.594831 | 1829.079245 |
| Prevalence | Caribbean | Female | 15-49 years | Polycystic ovarian syndrome | Rate | 2008 | 2698.298983 | 3886.864557 | 1845.820098 |
| Prevalence | Caribbean | Female | 15-49 years | Polycystic ovarian syndrome | Rate | 2009 | 2716.453467 | 3901.913954 | 1857.116105 |
| Prevalence | Caribbean | Female | 15-49 years | Polycystic ovarian syndrome | Rate | 2010 | 2730.566931 | 3915.22451 | 1870.532771 |
| Prevalence | Caribbean | Female | 15-49 years | Polycystic ovarian syndrome | Rate | 2011 | 2740.194034 | 3935.508943 | 1871.354852 |
| Prevalence | Caribbean | Female | 15-49 years | Polycystic ovarian syndrome | Rate | 2012 | 2746.589699 | 3953.363481 | 1879.526697 |
| Prevalence | Caribbean | Female | 15-49 years | Polycystic ovarian syndrome | Rate | 2013 | 2752.428691 | 3967.205559 | 1894.779452 |
| Prevalence | Caribbean | Female | 15-49 years | Polycystic ovarian syndrome | Rate | 2014 | 2758.148167 | 3978.82518 | 1903.65544 |
| Prevalence | Caribbean | Female | 15-49 years | Polycystic ovarian syndrome | Rate | 2015 | 2764.24109 | 3982.228742 | 1906.128039 |
| Prevalence | Caribbean | Female | 15-49 years | Polycystic ovarian syndrome | Rate | 2016 | 2772.427183 | 3974.067598 | 1913.849596 |
| Prevalence | Caribbean | Female | 15-49 years | Polycystic ovarian syndrome | Rate | 2017 | 2781.651296 | 3976.702916 | 1914.317053 |
| Prevalence | Caribbean | Female | 15-49 years | Polycystic ovarian syndrome | Rate | 2018 | 2793.456424 | 3990.302001 | 1924.077304 |
| Prevalence | Caribbean | Female | 15-49 years | Polycystic ovarian syndrome | Rate | 2019 | 2808.910744 | 3985.664011 | 1929.359073 |
| Prevalence | Caribbean | Female | 15-49 years | Polycystic ovarian syndrome | Rate | 2020 | 2838.753884 | 4096.79864 | 1937.39006 |
| Prevalence | Caribbean | Female | 15-49 years | Polycystic ovarian syndrome | Rate | 2021 | 2825.756836 | 4075.491197 | 1918.969034 |
| Prevalence | Andean Latin America | Female | 15-49 years | Polycystic ovarian syndrome | Rate | 1990 | 4568.865007 | 6358.3963 | 3146.350323 |
| Prevalence | Andean Latin America | Female | 15-49 years | Polycystic ovarian syndrome | Rate | 1991 | 4589.053738 | 6436.778663 | 3166.186327 |
| Prevalence | Andean Latin America | Female | 15-49 years | Polycystic ovarian syndrome | Rate | 1992 | 4621.054595 | 6528.869517 | 3189.273899 |
| Prevalence | Andean Latin America | Female | 15-49 years | Polycystic ovarian syndrome | Rate | 1993 | 4665.75141 | 6632.284079 | 3233.723083 |
| Prevalence | Andean Latin America | Female | 15-49 years | Polycystic ovarian syndrome | Rate | 1994 | 4722.798639 | 6731.6655 | 3262.489591 |
| Prevalence | Andean Latin America | Female | 15-49 years | Polycystic ovarian syndrome | Rate | 1995 | 4792.443894 | 6830.79016 | 3291.237149 |
| Prevalence | Andean Latin America | Female | 15-49 years | Polycystic ovarian syndrome | Rate | 1996 | 4891.662715 | 6950.212435 | 3398.46937 |
| Prevalence | Andean Latin America | Female | 15-49 years | Polycystic ovarian syndrome | Rate | 1997 | 5021.376755 | 7078.46659 | 3500.419259 |
| Prevalence | Andean Latin America | Female | 15-49 years | Polycystic ovarian syndrome | Rate | 1998 | 5155.45876 | 7204.94322 | 3602.641588 |
| Prevalence | Andean Latin America | Female | 15-49 years | Polycystic ovarian syndrome | Rate | 1999 | 5266.26689 | 7332.705801 | 3694.2472 |
| Prevalence | Andean Latin America | Female | 15-49 years | Polycystic ovarian syndrome | Rate | 2000 | 5327.288953 | 7406.060746 | 3747.096926 |
| Prevalence | Andean Latin America | Female | 15-49 years | Polycystic ovarian syndrome | Rate | 2001 | 5355.66384 | 7447.032768 | 3768.475687 |
| Prevalence | Andean Latin America | Female | 15-49 years | Polycystic ovarian syndrome | Rate | 2002 | 5386.074541 | 7471.271543 | 3796.925099 |
| Prevalence | Andean Latin America | Female | 15-49 years | Polycystic ovarian syndrome | Rate | 2003 | 5416.488367 | 7495.608689 | 3817.9628 |
| Prevalence | Andean Latin America | Female | 15-49 years | Polycystic ovarian syndrome | Rate | 2004 | 5445.179521 | 7506.035924 | 3837.977413 |
| Prevalence | Andean Latin America | Female | 15-49 years | Polycystic ovarian syndrome | Rate | 2005 | 5470.439019 | 7586.84025 | 3874.450246 |
| Prevalence | Andean Latin America | Female | 15-49 years | Polycystic ovarian syndrome | Rate | 2006 | 5524.007669 | 7621.927572 | 3929.333722 |
| Prevalence | Andean Latin America | Female | 15-49 years | Polycystic ovarian syndrome | Rate | 2007 | 5621.30677 | 7690.442114 | 3986.733067 |
| Prevalence | Andean Latin America | Female | 15-49 years | Polycystic ovarian syndrome | Rate | 2008 | 5736.268017 | 7818.98141 | 4034.343443 |
| Prevalence | Andean Latin America | Female | 15-49 years | Polycystic ovarian syndrome | Rate | 2009 | 5842.659797 | 7893.185337 | 4125.912792 |
| Prevalence | Andean Latin America | Female | 15-49 years | Polycystic ovarian syndrome | Rate | 2010 | 5914.01094 | 7926.529794 | 4200.866608 |
| Prevalence | Andean Latin America | Female | 15-49 years | Polycystic ovarian syndrome | Rate | 2011 | 5953.956579 | 8086.822552 | 4214.289677 |
| Prevalence | Andean Latin America | Female | 15-49 years | Polycystic ovarian syndrome | Rate | 2012 | 5985.877366 | 8170.233544 | 4201.518479 |
| Prevalence | Andean Latin America | Female | 15-49 years | Polycystic ovarian syndrome | Rate | 2013 | 6012.461103 | 8239.622758 | 4193.857828 |
| Prevalence | Andean Latin America | Female | 15-49 years | Polycystic ovarian syndrome | Rate | 2014 | 6037.615232 | 8325.973392 | 4202.0965 |
| Prevalence | Andean Latin America | Female | 15-49 years | Polycystic ovarian syndrome | Rate | 2015 | 6064.223912 | 8425.192178 | 4196.987898 |
| Prevalence | Andean Latin America | Female | 15-49 years | Polycystic ovarian syndrome | Rate | 2016 | 6093.979152 | 8467.88175 | 4236.342459 |
| Prevalence | Andean Latin America | Female | 15-49 years | Polycystic ovarian syndrome | Rate | 2017 | 6126.237471 | 8572.820235 | 4287.138246 |
| Prevalence | Andean Latin America | Female | 15-49 years | Polycystic ovarian syndrome | Rate | 2018 | 6163.426413 | 8696.59985 | 4317.986525 |
| Prevalence | Andean Latin America | Female | 15-49 years | Polycystic ovarian syndrome | Rate | 2019 | 6206.759811 | 8814.255973 | 4353.602962 |
| Prevalence | Andean Latin America | Female | 15-49 years | Polycystic ovarian syndrome | Rate | 2020 | 6338.065263 | 8990.435262 | 4466.13962 |
| Prevalence | Andean Latin America | Female | 15-49 years | Polycystic ovarian syndrome | Rate | 2021 | 6332.514086 | 8867.701656 | 4336.269668 |
| Prevalence | High-income North America | Female | 15-49 years | Polycystic ovarian syndrome | Rate | 1990 | 5765.462369 | 8071.026002 | 4067.270917 |
| Prevalence | High-income North America | Female | 15-49 years | Polycystic ovarian syndrome | Rate | 1991 | 5815.485362 | 8144.856414 | 4098.119026 |
| Prevalence | High-income North America | Female | 15-49 years | Polycystic ovarian syndrome | Rate | 1992 | 5853.584639 | 8208.327718 | 4147.524012 |
| Prevalence | High-income North America | Female | 15-49 years | Polycystic ovarian syndrome | Rate | 1993 | 5887.12436 | 8275.956047 | 4177.12309 |
| Prevalence | High-income North America | Female | 15-49 years | Polycystic ovarian syndrome | Rate | 1994 | 5916.27285 | 8320.642966 | 4209.315221 |
| Prevalence | High-income North America | Female | 15-49 years | Polycystic ovarian syndrome | Rate | 1995 | 5938.997861 | 8331.628061 | 4242.286681 |
| Prevalence | High-income North America | Female | 15-49 years | Polycystic ovarian syndrome | Rate | 1996 | 5968.966932 | 8320.189172 | 4258.632999 |
| Prevalence | High-income North America | Female | 15-49 years | Polycystic ovarian syndrome | Rate | 1997 | 6014.116438 | 8328.906523 | 4295.357087 |
| Prevalence | High-income North America | Female | 15-49 years | Polycystic ovarian syndrome | Rate | 1998 | 6056.345576 | 8377.465673 | 4347.943985 |
| Prevalence | High-income North America | Female | 15-49 years | Polycystic ovarian syndrome | Rate | 1999 | 6082.624935 | 8383.410458 | 4398.072947 |
| Prevalence | High-income North America | Female | 15-49 years | Polycystic ovarian syndrome | Rate | 2000 | 6081.181289 | 8374.450013 | 4332.780607 |
| Prevalence | High-income North America | Female | 15-49 years | Polycystic ovarian syndrome | Rate | 2001 | 6046.675575 | 8293.628976 | 4356.481435 |
| Prevalence | High-income North America | Female | 15-49 years | Polycystic ovarian syndrome | Rate | 2002 | 5986.204828 | 8184.357959 | 4299.307569 |
| Prevalence | High-income North America | Female | 15-49 years | Polycystic ovarian syndrome | Rate | 2003 | 5906.005805 | 8052.2209 | 4264.369585 |
| Prevalence | High-income North America | Female | 15-49 years | Polycystic ovarian syndrome | Rate | 2004 | 5811.895064 | 7892.942193 | 4215.215553 |
| Prevalence | High-income North America | Female | 15-49 years | Polycystic ovarian syndrome | Rate | 2005 | 5709.196206 | 7775.64875 | 4180.231194 |
| Prevalence | High-income North America | Female | 15-49 years | Polycystic ovarian syndrome | Rate | 2006 | 5494.742281 | 7274.791823 | 4129.02828 |
| Prevalence | High-income North America | Female | 15-49 years | Polycystic ovarian syndrome | Rate | 2007 | 5134.494999 | 6600.311797 | 4005.338982 |
| Prevalence | High-income North America | Female | 15-49 years | Polycystic ovarian syndrome | Rate | 2008 | 4740.211736 | 5904.514182 | 3839.105277 |
| Prevalence | High-income North America | Female | 15-49 years | Polycystic ovarian syndrome | Rate | 2009 | 4422.874905 | 5343.548591 | 3634.612075 |
| Prevalence | High-income North America | Female | 15-49 years | Polycystic ovarian syndrome | Rate | 2010 | 4295.13258 | 5120.639568 | 3543.856064 |
| Prevalence | High-income North America | Female | 15-49 years | Polycystic ovarian syndrome | Rate | 2011 | 4312.514724 | 5140.123886 | 3574.113046 |
| Prevalence | High-income North America | Female | 15-49 years | Polycystic ovarian syndrome | Rate | 2012 | 4352.668217 | 5168.653715 | 3610.614402 |
| Prevalence | High-income North America | Female | 15-49 years | Polycystic ovarian syndrome | Rate | 2013 | 4410.658405 | 5217.926017 | 3665.340292 |
| Prevalence | High-income North America | Female | 15-49 years | Polycystic ovarian syndrome | Rate | 2014 | 4481.021351 | 5286.207016 | 3719.989909 |
| Prevalence | High-income North America | Female | 15-49 years | Polycystic ovarian syndrome | Rate | 2015 | 4557.710437 | 5367.087917 | 3788.422187 |
| Prevalence | High-income North America | Female | 15-49 years | Polycystic ovarian syndrome | Rate | 2016 | 4739.539322 | 5608.817966 | 3949.71295 |
| Prevalence | High-income North America | Female | 15-49 years | Polycystic ovarian syndrome | Rate | 2017 | 5081.630637 | 6098.623587 | 4221.836181 |
| Prevalence | High-income North America | Female | 15-49 years | Polycystic ovarian syndrome | Rate | 2018 | 5510.681333 | 6705.472411 | 4546.8225 |
| Prevalence | High-income North America | Female | 15-49 years | Polycystic ovarian syndrome | Rate | 2019 | 5953.701581 | 7400.937485 | 4833.636518 |
| Prevalence | High-income North America | Female | 15-49 years | Polycystic ovarian syndrome | Rate | 2020 | 6465.106125 | 8169.902322 | 5028.368238 |
| Prevalence | High-income North America | Female | 15-49 years | Polycystic ovarian syndrome | Rate | 2021 | 7225.929795 | 9514.859367 | 5394.792823 |
| Prevalence | North Africa and Middle East | Female | 15-49 years | Polycystic ovarian syndrome | Rate | 1990 | 2963.069211 | 4208.839181 | 2055.04002 |
| Prevalence | North Africa and Middle East | Female | 15-49 years | Polycystic ovarian syndrome | Rate | 1991 | 2989.828987 | 4226.678954 | 2081.440203 |
| Prevalence | North Africa and Middle East | Female | 15-49 years | Polycystic ovarian syndrome | Rate | 1992 | 3016.899599 | 4254.162307 | 2104.486083 |
| Prevalence | North Africa and Middle East | Female | 15-49 years | Polycystic ovarian syndrome | Rate | 1993 | 3042.172177 | 4285.768944 | 2119.887595 |
| Prevalence | North Africa and Middle East | Female | 15-49 years | Polycystic ovarian syndrome | Rate | 1994 | 3067.092142 | 4316.845468 | 2135.131403 |
| Prevalence | North Africa and Middle East | Female | 15-49 years | Polycystic ovarian syndrome | Rate | 1995 | 3093.679835 | 4348.861919 | 2151.157508 |
| Prevalence | North Africa and Middle East | Female | 15-49 years | Polycystic ovarian syndrome | Rate | 1996 | 3124.177664 | 4393.707278 | 2170.489707 |
| Prevalence | North Africa and Middle East | Female | 15-49 years | Polycystic ovarian syndrome | Rate | 1997 | 3159.670834 | 4447.38127 | 2193.105625 |
| Prevalence | North Africa and Middle East | Female | 15-49 years | Polycystic ovarian syndrome | Rate | 1998 | 3198.264837 | 4504.797157 | 2224.12496 |
| Prevalence | North Africa and Middle East | Female | 15-49 years | Polycystic ovarian syndrome | Rate | 1999 | 3238.915604 | 4570.720334 | 2255.75456 |
| Prevalence | North Africa and Middle East | Female | 15-49 years | Polycystic ovarian syndrome | Rate | 2000 | 3280.08113 | 4641.793203 | 2292.328986 |
| Prevalence | North Africa and Middle East | Female | 15-49 years | Polycystic ovarian syndrome | Rate | 2001 | 3335.242201 | 4708.709123 | 2324.502525 |
| Prevalence | North Africa and Middle East | Female | 15-49 years | Polycystic ovarian syndrome | Rate | 2002 | 3405.634267 | 4806.289101 | 2367.222248 |
| Prevalence | North Africa and Middle East | Female | 15-49 years | Polycystic ovarian syndrome | Rate | 2003 | 3478.400561 | 4911.608385 | 2415.503289 |
| Prevalence | North Africa and Middle East | Female | 15-49 years | Polycystic ovarian syndrome | Rate | 2004 | 3544.779479 | 5006.56288 | 2463.986863 |
| Prevalence | North Africa and Middle East | Female | 15-49 years | Polycystic ovarian syndrome | Rate | 2005 | 3593.922636 | 5076.826224 | 2500.668551 |
| Prevalence | North Africa and Middle East | Female | 15-49 years | Polycystic ovarian syndrome | Rate | 2006 | 3630.159316 | 5126.867217 | 2527.433114 |
| Prevalence | North Africa and Middle East | Female | 15-49 years | Polycystic ovarian syndrome | Rate | 2007 | 3667.337158 | 5172.452216 | 2555.782143 |
| Prevalence | North Africa and Middle East | Female | 15-49 years | Polycystic ovarian syndrome | Rate | 2008 | 3704.413259 | 5218.365315 | 2587.748148 |
| Prevalence | North Africa and Middle East | Female | 15-49 years | Polycystic ovarian syndrome | Rate | 2009 | 3738.963607 | 5277.253542 | 2617.947516 |
| Prevalence | North Africa and Middle East | Female | 15-49 years | Polycystic ovarian syndrome | Rate | 2010 | 3771.145493 | 5318.782849 | 2638.95127 |
| Prevalence | North Africa and Middle East | Female | 15-49 years | Polycystic ovarian syndrome | Rate | 2011 | 3806.791687 | 5360.861749 | 2665.828533 |
| Prevalence | North Africa and Middle East | Female | 15-49 years | Polycystic ovarian syndrome | Rate | 2012 | 3847.415224 | 5425.133686 | 2695.085946 |
| Prevalence | North Africa and Middle East | Female | 15-49 years | Polycystic ovarian syndrome | Rate | 2013 | 3889.330674 | 5492.957625 | 2722.089724 |
| Prevalence | North Africa and Middle East | Female | 15-49 years | Polycystic ovarian syndrome | Rate | 2014 | 3928.38875 | 5553.098695 | 2746.513326 |
| Prevalence | North Africa and Middle East | Female | 15-49 years | Polycystic ovarian syndrome | Rate | 2015 | 3959.724796 | 5602.722015 | 2764.875738 |
| Prevalence | North Africa and Middle East | Female | 15-49 years | Polycystic ovarian syndrome | Rate | 2016 | 3968.406912 | 5615.511236 | 2773.84338 |
| Prevalence | North Africa and Middle East | Female | 15-49 years | Polycystic ovarian syndrome | Rate | 2017 | 3957.382199 | 5601.473494 | 2765.837977 |
| Prevalence | North Africa and Middle East | Female | 15-49 years | Polycystic ovarian syndrome | Rate | 2018 | 3948.509436 | 5588.29718 | 2754.287001 |
| Prevalence | North Africa and Middle East | Female | 15-49 years | Polycystic ovarian syndrome | Rate | 2019 | 3962.727374 | 5606.473276 | 2775.894286 |
| Prevalence | North Africa and Middle East | Female | 15-49 years | Polycystic ovarian syndrome | Rate | 2020 | 4039.470966 | 5717.401787 | 2804.772375 |
| Prevalence | North Africa and Middle East | Female | 15-49 years | Polycystic ovarian syndrome | Rate | 2021 | 3975.801762 | 5616.815343 | 2791.126615 |
| Prevalence | Southern Latin America | Female | 15-49 years | Polycystic ovarian syndrome | Rate | 1990 | 2276.465813 | 3306.583867 | 1573.891039 |
| Prevalence | Southern Latin America | Female | 15-49 years | Polycystic ovarian syndrome | Rate | 1991 | 2340.321977 | 3337.164 | 1638.348397 |
| Prevalence | Southern Latin America | Female | 15-49 years | Polycystic ovarian syndrome | Rate | 1992 | 2408.268776 | 3433.554449 | 1687.141686 |
| Prevalence | Southern Latin America | Female | 15-49 years | Polycystic ovarian syndrome | Rate | 1993 | 2479.403386 | 3523.362347 | 1738.018147 |
| Prevalence | Southern Latin America | Female | 15-49 years | Polycystic ovarian syndrome | Rate | 1994 | 2553.339997 | 3624.964725 | 1793.308592 |
| Prevalence | Southern Latin America | Female | 15-49 years | Polycystic ovarian syndrome | Rate | 1995 | 2630.114871 | 3746.09741 | 1838.66731 |
| Prevalence | Southern Latin America | Female | 15-49 years | Polycystic ovarian syndrome | Rate | 1996 | 2715.309522 | 3859.814675 | 1926.032615 |
| Prevalence | Southern Latin America | Female | 15-49 years | Polycystic ovarian syndrome | Rate | 1997 | 2809.247213 | 3973.941516 | 2012.943011 |
| Prevalence | Southern Latin America | Female | 15-49 years | Polycystic ovarian syndrome | Rate | 1998 | 2905.085021 | 4090.3512 | 2092.110969 |
| Prevalence | Southern Latin America | Female | 15-49 years | Polycystic ovarian syndrome | Rate | 1999 | 2995.802198 | 4201.26622 | 2149.747453 |
| Prevalence | Southern Latin America | Female | 15-49 years | Polycystic ovarian syndrome | Rate | 2000 | 3073.684652 | 4297.298979 | 2206.23856 |
| Prevalence | Southern Latin America | Female | 15-49 years | Polycystic ovarian syndrome | Rate | 2001 | 3142.109443 | 4394.927993 | 2255.07279 |
| Prevalence | Southern Latin America | Female | 15-49 years | Polycystic ovarian syndrome | Rate | 2002 | 3208.073173 | 4475.239769 | 2298.609526 |
| Prevalence | Southern Latin America | Female | 15-49 years | Polycystic ovarian syndrome | Rate | 2003 | 3268.22631 | 4565.91814 | 2354.404199 |
| Prevalence | Southern Latin America | Female | 15-49 years | Polycystic ovarian syndrome | Rate | 2004 | 3319.686339 | 4652.196867 | 2392.762784 |
| Prevalence | Southern Latin America | Female | 15-49 years | Polycystic ovarian syndrome | Rate | 2005 | 3359.88539 | 4728.288342 | 2429.637951 |
| Prevalence | Southern Latin America | Female | 15-49 years | Polycystic ovarian syndrome | Rate | 2006 | 3393.658627 | 4770.040968 | 2449.106742 |
| Prevalence | Southern Latin America | Female | 15-49 years | Polycystic ovarian syndrome | Rate | 2007 | 3426.700787 | 4835.704837 | 2452.741983 |
| Prevalence | Southern Latin America | Female | 15-49 years | Polycystic ovarian syndrome | Rate | 2008 | 3457.446731 | 4894.837073 | 2470.579835 |
| Prevalence | Southern Latin America | Female | 15-49 years | Polycystic ovarian syndrome | Rate | 2009 | 3484.307155 | 4954.33857 | 2477.607801 |
| Prevalence | Southern Latin America | Female | 15-49 years | Polycystic ovarian syndrome | Rate | 2010 | 3505.700253 | 5017.06104 | 2489.066708 |
| Prevalence | Southern Latin America | Female | 15-49 years | Polycystic ovarian syndrome | Rate | 2011 | 3522.611271 | 5043.334002 | 2511.698954 |
| Prevalence | Southern Latin America | Female | 15-49 years | Polycystic ovarian syndrome | Rate | 2012 | 3537.391641 | 5057.090395 | 2522.397847 |
| Prevalence | Southern Latin America | Female | 15-49 years | Polycystic ovarian syndrome | Rate | 2013 | 3550.320728 | 5049.504073 | 2518.313842 |
| Prevalence | Southern Latin America | Female | 15-49 years | Polycystic ovarian syndrome | Rate | 2014 | 3561.753431 | 5092.857878 | 2505.414454 |
| Prevalence | Southern Latin America | Female | 15-49 years | Polycystic ovarian syndrome | Rate | 2015 | 3572.583876 | 5106.241987 | 2495.079359 |
| Prevalence | Southern Latin America | Female | 15-49 years | Polycystic ovarian syndrome | Rate | 2016 | 3584.267213 | 5081.077712 | 2499.947875 |
| Prevalence | Southern Latin America | Female | 15-49 years | Polycystic ovarian syndrome | Rate | 2017 | 3598.500578 | 5100.709881 | 2509.086412 |
| Prevalence | Southern Latin America | Female | 15-49 years | Polycystic ovarian syndrome | Rate | 2018 | 3616.632227 | 5110.345256 | 2526.908212 |
| Prevalence | Southern Latin America | Female | 15-49 years | Polycystic ovarian syndrome | Rate | 2019 | 3639.065925 | 5131.422195 | 2546.876398 |
| Prevalence | Southern Latin America | Female | 15-49 years | Polycystic ovarian syndrome | Rate | 2020 | 3679.60512 | 5358.495581 | 2576.259992 |
| Prevalence | Southern Latin America | Female | 15-49 years | Polycystic ovarian syndrome | Rate | 2021 | 3658.164865 | 5251.351076 | 2573.534517 |
| Prevalence | Tropical Latin America | Female | 15-49 years | Polycystic ovarian syndrome | Rate | 1990 | 1049.339208 | 1515.185782 | 710.4638743 |
| Prevalence | Tropical Latin America | Female | 15-49 years | Polycystic ovarian syndrome | Rate | 1991 | 1087.577374 | 1576.178544 | 733.1176839 |
| Prevalence | Tropical Latin America | Female | 15-49 years | Polycystic ovarian syndrome | Rate | 1992 | 1120.015609 | 1625.528975 | 752.2657026 |
| Prevalence | Tropical Latin America | Female | 15-49 years | Polycystic ovarian syndrome | Rate | 1993 | 1145.307009 | 1665.315612 | 770.2975347 |
| Prevalence | Tropical Latin America | Female | 15-49 years | Polycystic ovarian syndrome | Rate | 1994 | 1162.189706 | 1691.670295 | 783.0782328 |
| Prevalence | Tropical Latin America | Female | 15-49 years | Polycystic ovarian syndrome | Rate | 1995 | 1169.539047 | 1704.207792 | 792.1721109 |
| Prevalence | Tropical Latin America | Female | 15-49 years | Polycystic ovarian syndrome | Rate | 1996 | 1167.321626 | 1691.24803 | 792.0722459 |
| Prevalence | Tropical Latin America | Female | 15-49 years | Polycystic ovarian syndrome | Rate | 1997 | 1158.550003 | 1674.832081 | 786.2874307 |
| Prevalence | Tropical Latin America | Female | 15-49 years | Polycystic ovarian syndrome | Rate | 1998 | 1146.629012 | 1647.755131 | 778.6236553 |
| Prevalence | Tropical Latin America | Female | 15-49 years | Polycystic ovarian syndrome | Rate | 1999 | 1134.977882 | 1626.497686 | 771.0996396 |
| Prevalence | Tropical Latin America | Female | 15-49 years | Polycystic ovarian syndrome | Rate | 2000 | 1127.04886 | 1610.398432 | 766.5567987 |
| Prevalence | Tropical Latin America | Female | 15-49 years | Polycystic ovarian syndrome | Rate | 2001 | 1123.142339 | 1607.321659 | 764.1524612 |
| Prevalence | Tropical Latin America | Female | 15-49 years | Polycystic ovarian syndrome | Rate | 2002 | 1120.102498 | 1604.566915 | 762.5015141 |
| Prevalence | Tropical Latin America | Female | 15-49 years | Polycystic ovarian syndrome | Rate | 2003 | 1117.105407 | 1600.974935 | 761.4357306 |
| Prevalence | Tropical Latin America | Female | 15-49 years | Polycystic ovarian syndrome | Rate | 2004 | 1113.363345 | 1595.735358 | 763.2332026 |
| Prevalence | Tropical Latin America | Female | 15-49 years | Polycystic ovarian syndrome | Rate | 2005 | 1108.04267 | 1588.291503 | 761.5141407 |
| Prevalence | Tropical Latin America | Female | 15-49 years | Polycystic ovarian syndrome | Rate | 2006 | 1092.497602 | 1552.787903 | 751.050016 |
| Prevalence | Tropical Latin America | Female | 15-49 years | Polycystic ovarian syndrome | Rate | 2007 | 1064.836212 | 1504.543836 | 729.2901178 |
| Prevalence | Tropical Latin America | Female | 15-49 years | Polycystic ovarian syndrome | Rate | 2008 | 1034.393447 | 1455.179089 | 706.0483872 |
| Prevalence | Tropical Latin America | Female | 15-49 years | Polycystic ovarian syndrome | Rate | 2009 | 1010.639938 | 1409.633134 | 691.0042186 |
| Prevalence | Tropical Latin America | Female | 15-49 years | Polycystic ovarian syndrome | Rate | 2010 | 1003.17113 | 1399.163813 | 684.4959762 |
| Prevalence | Tropical Latin America | Female | 15-49 years | Polycystic ovarian syndrome | Rate | 2011 | 1009.726084 | 1410.626294 | 689.3251635 |
| Prevalence | Tropical Latin America | Female | 15-49 years | Polycystic ovarian syndrome | Rate | 2012 | 1020.643287 | 1428.269274 | 697.4481984 |
| Prevalence | Tropical Latin America | Female | 15-49 years | Polycystic ovarian syndrome | Rate | 2013 | 1034.362838 | 1450.285223 | 707.8370955 |
| Prevalence | Tropical Latin America | Female | 15-49 years | Polycystic ovarian syndrome | Rate | 2014 | 1049.276733 | 1475.33754 | 719.0736238 |
| Prevalence | Tropical Latin America | Female | 15-49 years | Polycystic ovarian syndrome | Rate | 2015 | 1063.696561 | 1499.798273 | 729.646024 |
| Prevalence | Tropical Latin America | Female | 15-49 years | Polycystic ovarian syndrome | Rate | 2016 | 1081.650805 | 1526.183476 | 740.5980886 |
| Prevalence | Tropical Latin America | Female | 15-49 years | Polycystic ovarian syndrome | Rate | 2017 | 1104.219647 | 1559.100591 | 753.1935891 |
| Prevalence | Tropical Latin America | Female | 15-49 years | Polycystic ovarian syndrome | Rate | 2018 | 1125.40505 | 1593.881181 | 766.3082564 |
| Prevalence | Tropical Latin America | Female | 15-49 years | Polycystic ovarian syndrome | Rate | 2019 | 1139.142437 | 1618.450506 | 775.3590339 |
| Prevalence | Tropical Latin America | Female | 15-49 years | Polycystic ovarian syndrome | Rate | 2020 | 1143.702885 | 1636.717074 | 784.6420506 |
| Prevalence | Tropical Latin America | Female | 15-49 years | Polycystic ovarian syndrome | Rate | 2021 | 1145.465122 | 1624.711388 | 777.9392585 |
| Prevalence | Central Sub-Saharan Africa | Female | 15-49 years | Polycystic ovarian syndrome | Rate | 1990 | 843.9649082 | 1228.214695 | 583.9045482 |
| Prevalence | Central Sub-Saharan Africa | Female | 15-49 years | Polycystic ovarian syndrome | Rate | 1991 | 849.1189819 | 1231.936037 | 586.0157122 |
| Prevalence | Central Sub-Saharan Africa | Female | 15-49 years | Polycystic ovarian syndrome | Rate | 1992 | 855.9604595 | 1237.878809 | 597.1449824 |
| Prevalence | Central Sub-Saharan Africa | Female | 15-49 years | Polycystic ovarian syndrome | Rate | 1993 | 864.2765352 | 1240.707273 | 604.9976233 |
| Prevalence | Central Sub-Saharan Africa | Female | 15-49 years | Polycystic ovarian syndrome | Rate | 1994 | 873.83009 | 1249.96752 | 610.4965943 |
| Prevalence | Central Sub-Saharan Africa | Female | 15-49 years | Polycystic ovarian syndrome | Rate | 1995 | 884.5349367 | 1260.860584 | 613.294713 |
| Prevalence | Central Sub-Saharan Africa | Female | 15-49 years | Polycystic ovarian syndrome | Rate | 1996 | 910.2527741 | 1303.293433 | 637.9082853 |
| Prevalence | Central Sub-Saharan Africa | Female | 15-49 years | Polycystic ovarian syndrome | Rate | 1997 | 956.3899207 | 1372.445852 | 666.8525555 |
| Prevalence | Central Sub-Saharan Africa | Female | 15-49 years | Polycystic ovarian syndrome | Rate | 1998 | 1008.441458 | 1454.614283 | 696.9082467 |
| Prevalence | Central Sub-Saharan Africa | Female | 15-49 years | Polycystic ovarian syndrome | Rate | 1999 | 1052.023001 | 1522.27154 | 722.7554448 |
| Prevalence | Central Sub-Saharan Africa | Female | 15-49 years | Polycystic ovarian syndrome | Rate | 2000 | 1072.628506 | 1552.949938 | 735.8495516 |
| Prevalence | Central Sub-Saharan Africa | Female | 15-49 years | Polycystic ovarian syndrome | Rate | 2001 | 1076.366932 | 1553.527557 | 740.5728845 |
| Prevalence | Central Sub-Saharan Africa | Female | 15-49 years | Polycystic ovarian syndrome | Rate | 2002 | 1078.692005 | 1553.864141 | 743.1958516 |
| Prevalence | Central Sub-Saharan Africa | Female | 15-49 years | Polycystic ovarian syndrome | Rate | 2003 | 1080.337011 | 1550.63608 | 745.4469761 |
| Prevalence | Central Sub-Saharan Africa | Female | 15-49 years | Polycystic ovarian syndrome | Rate | 2004 | 1082.013156 | 1559.392684 | 752.015507 |
| Prevalence | Central Sub-Saharan Africa | Female | 15-49 years | Polycystic ovarian syndrome | Rate | 2005 | 1084.438778 | 1566.709077 | 755.8763273 |
| Prevalence | Central Sub-Saharan Africa | Female | 15-49 years | Polycystic ovarian syndrome | Rate | 2006 | 1087.781614 | 1561.724753 | 754.0641686 |
| Prevalence | Central Sub-Saharan Africa | Female | 15-49 years | Polycystic ovarian syndrome | Rate | 2007 | 1091.778564 | 1566.727935 | 757.1607213 |
| Prevalence | Central Sub-Saharan Africa | Female | 15-49 years | Polycystic ovarian syndrome | Rate | 2008 | 1096.487747 | 1573.122363 | 761.7949558 |
| Prevalence | Central Sub-Saharan Africa | Female | 15-49 years | Polycystic ovarian syndrome | Rate | 2009 | 1101.961385 | 1580.234147 | 759.206669 |
| Prevalence | Central Sub-Saharan Africa | Female | 15-49 years | Polycystic ovarian syndrome | Rate | 2010 | 1108.236216 | 1587.567879 | 757.9949951 |
| Prevalence | Central Sub-Saharan Africa | Female | 15-49 years | Polycystic ovarian syndrome | Rate | 2011 | 1117.495087 | 1605.678684 | 764.7719568 |
| Prevalence | Central Sub-Saharan Africa | Female | 15-49 years | Polycystic ovarian syndrome | Rate | 2012 | 1130.848644 | 1625.590753 | 771.6939703 |
| Prevalence | Central Sub-Saharan Africa | Female | 15-49 years | Polycystic ovarian syndrome | Rate | 2013 | 1146.739269 | 1645.344906 | 784.4908711 |
| Prevalence | Central Sub-Saharan Africa | Female | 15-49 years | Polycystic ovarian syndrome | Rate | 2014 | 1163.644541 | 1667.113764 | 798.0814106 |
| Prevalence | Central Sub-Saharan Africa | Female | 15-49 years | Polycystic ovarian syndrome | Rate | 2015 | 1179.994487 | 1694.021116 | 810.7380894 |
| Prevalence | Central Sub-Saharan Africa | Female | 15-49 years | Polycystic ovarian syndrome | Rate | 2016 | 1195.834074 | 1725.086694 | 825.753407 |
| Prevalence | Central Sub-Saharan Africa | Female | 15-49 years | Polycystic ovarian syndrome | Rate | 2017 | 1212.779261 | 1752.356567 | 835.5416527 |
| Prevalence | Central Sub-Saharan Africa | Female | 15-49 years | Polycystic ovarian syndrome | Rate | 2018 | 1231.549193 | 1769.721706 | 849.212514 |
| Prevalence | Central Sub-Saharan Africa | Female | 15-49 years | Polycystic ovarian syndrome | Rate | 2019 | 1252.817367 | 1782.54699 | 861.4455848 |
| Prevalence | Central Sub-Saharan Africa | Female | 15-49 years | Polycystic ovarian syndrome | Rate | 2020 | 1290.832033 | 1826.283556 | 898.0804668 |
| Prevalence | Central Sub-Saharan Africa | Female | 15-49 years | Polycystic ovarian syndrome | Rate | 2021 | 1281.701839 | 1849.839093 | 886.2606343 |
| Prevalence | Central Latin America | Female | 15-49 years | Polycystic ovarian syndrome | Rate | 1990 | 5077.662123 | 7054.774252 | 3485.100182 |
| Prevalence | Central Latin America | Female | 15-49 years | Polycystic ovarian syndrome | Rate | 1991 | 5280.026193 | 7322.110817 | 3627.228308 |
| Prevalence | Central Latin America | Female | 15-49 years | Polycystic ovarian syndrome | Rate | 1992 | 5447.391157 | 7542.362402 | 3744.094742 |
| Prevalence | Central Latin America | Female | 15-49 years | Polycystic ovarian syndrome | Rate | 1993 | 5575.931776 | 7710.56214 | 3833.566502 |
| Prevalence | Central Latin America | Female | 15-49 years | Polycystic ovarian syndrome | Rate | 1994 | 5662.15563 | 7842.285477 | 3892.229679 |
| Prevalence | Central Latin America | Female | 15-49 years | Polycystic ovarian syndrome | Rate | 1995 | 5703.450649 | 7921.328427 | 3917.332088 |
| Prevalence | Central Latin America | Female | 15-49 years | Polycystic ovarian syndrome | Rate | 1996 | 5693.544583 | 7906.068079 | 3928.33818 |
| Prevalence | Central Latin America | Female | 15-49 years | Polycystic ovarian syndrome | Rate | 1997 | 5642.394937 | 7796.80372 | 3915.733658 |
| Prevalence | Central Latin America | Female | 15-49 years | Polycystic ovarian syndrome | Rate | 1998 | 5567.507174 | 7678.605279 | 3887.535963 |
| Prevalence | Central Latin America | Female | 15-49 years | Polycystic ovarian syndrome | Rate | 1999 | 5487.682013 | 7583.666487 | 3837.692968 |
| Prevalence | Central Latin America | Female | 15-49 years | Polycystic ovarian syndrome | Rate | 2000 | 5419.612995 | 7489.695112 | 3796.338991 |
| Prevalence | Central Latin America | Female | 15-49 years | Polycystic ovarian syndrome | Rate | 2001 | 5361.902038 | 7382.5544 | 3765.004562 |
| Prevalence | Central Latin America | Female | 15-49 years | Polycystic ovarian syndrome | Rate | 2002 | 5302.106726 | 7269.275174 | 3734.714995 |
| Prevalence | Central Latin America | Female | 15-49 years | Polycystic ovarian syndrome | Rate | 2003 | 5244.223187 | 7167.437601 | 3713.491638 |
| Prevalence | Central Latin America | Female | 15-49 years | Polycystic ovarian syndrome | Rate | 2004 | 5192.290582 | 7105.394553 | 3697.047611 |
| Prevalence | Central Latin America | Female | 15-49 years | Polycystic ovarian syndrome | Rate | 2005 | 5150.340732 | 7018.660108 | 3682.415724 |
| Prevalence | Central Latin America | Female | 15-49 years | Polycystic ovarian syndrome | Rate | 2006 | 5110.125456 | 6949.697548 | 3659.411957 |
| Prevalence | Central Latin America | Female | 15-49 years | Polycystic ovarian syndrome | Rate | 2007 | 5066.495171 | 6876.138504 | 3631.113795 |
| Prevalence | Central Latin America | Female | 15-49 years | Polycystic ovarian syndrome | Rate | 2008 | 5028.204664 | 6836.423752 | 3599.644985 |
| Prevalence | Central Latin America | Female | 15-49 years | Polycystic ovarian syndrome | Rate | 2009 | 5003.831119 | 6810.229508 | 3578.249893 |
| Prevalence | Central Latin America | Female | 15-49 years | Polycystic ovarian syndrome | Rate | 2010 | 5001.783219 | 6818.976368 | 3576.322798 |
| Prevalence | Central Latin America | Female | 15-49 years | Polycystic ovarian syndrome | Rate | 2011 | 5021.355697 | 6862.25617 | 3596.884873 |
| Prevalence | Central Latin America | Female | 15-49 years | Polycystic ovarian syndrome | Rate | 2012 | 5053.719687 | 6927.480968 | 3605.673681 |
| Prevalence | Central Latin America | Female | 15-49 years | Polycystic ovarian syndrome | Rate | 2013 | 5095.056988 | 6954.732936 | 3621.727186 |
| Prevalence | Central Latin America | Female | 15-49 years | Polycystic ovarian syndrome | Rate | 2014 | 5141.361667 | 7018.234348 | 3646.930136 |
| Prevalence | Central Latin America | Female | 15-49 years | Polycystic ovarian syndrome | Rate | 2015 | 5188.651304 | 7141.347263 | 3687.45092 |
| Prevalence | Central Latin America | Female | 15-49 years | Polycystic ovarian syndrome | Rate | 2016 | 5268.765019 | 7256.196036 | 3741.222244 |
| Prevalence | Central Latin America | Female | 15-49 years | Polycystic ovarian syndrome | Rate | 2017 | 5388.116551 | 7435.536597 | 3820.28539 |
| Prevalence | Central Latin America | Female | 15-49 years | Polycystic ovarian syndrome | Rate | 2018 | 5504.813859 | 7606.661434 | 3893.857064 |
| Prevalence | Central Latin America | Female | 15-49 years | Polycystic ovarian syndrome | Rate | 2019 | 5577.591451 | 7706.995472 | 3938.210117 |
| Prevalence | Central Latin America | Female | 15-49 years | Polycystic ovarian syndrome | Rate | 2020 | 5600.223642 | 7738.819198 | 3901.253822 |
| Prevalence | Central Latin America | Female | 15-49 years | Polycystic ovarian syndrome | Rate | 2021 | 5582.444987 | 7803.8388 | 3878.649748 |
| Prevalence | Western Sub-Saharan Africa | Female | 15-49 years | Polycystic ovarian syndrome | Rate | 1990 | 951.1332592 | 1373.003273 | 664.3030223 |
| Prevalence | Western Sub-Saharan Africa | Female | 15-49 years | Polycystic ovarian syndrome | Rate | 1991 | 992.2939964 | 1430.0057 | 694.3007015 |
| Prevalence | Western Sub-Saharan Africa | Female | 15-49 years | Polycystic ovarian syndrome | Rate | 1992 | 1031.597998 | 1484.835266 | 722.1198231 |
| Prevalence | Western Sub-Saharan Africa | Female | 15-49 years | Polycystic ovarian syndrome | Rate | 1993 | 1068.229905 | 1538.916338 | 746.927284 |
| Prevalence | Western Sub-Saharan Africa | Female | 15-49 years | Polycystic ovarian syndrome | Rate | 1994 | 1101.609258 | 1590.567868 | 769.2326221 |
| Prevalence | Western Sub-Saharan Africa | Female | 15-49 years | Polycystic ovarian syndrome | Rate | 1995 | 1130.339576 | 1630.587734 | 788.3570475 |
| Prevalence | Western Sub-Saharan Africa | Female | 15-49 years | Polycystic ovarian syndrome | Rate | 1996 | 1157.238167 | 1671.210695 | 806.3276706 |
| Prevalence | Western Sub-Saharan Africa | Female | 15-49 years | Polycystic ovarian syndrome | Rate | 1997 | 1183.590076 | 1706.320032 | 824.1817752 |
| Prevalence | Western Sub-Saharan Africa | Female | 15-49 years | Polycystic ovarian syndrome | Rate | 1998 | 1207.877502 | 1737.707315 | 841.5480985 |
| Prevalence | Western Sub-Saharan Africa | Female | 15-49 years | Polycystic ovarian syndrome | Rate | 1999 | 1228.865747 | 1762.430161 | 856.4855976 |
| Prevalence | Western Sub-Saharan Africa | Female | 15-49 years | Polycystic ovarian syndrome | Rate | 2000 | 1245.863089 | 1780.093107 | 867.4070559 |
| Prevalence | Western Sub-Saharan Africa | Female | 15-49 years | Polycystic ovarian syndrome | Rate | 2001 | 1260.004723 | 1799.359145 | 875.6816276 |
| Prevalence | Western Sub-Saharan Africa | Female | 15-49 years | Polycystic ovarian syndrome | Rate | 2002 | 1274.152241 | 1820.321497 | 882.8411466 |
| Prevalence | Western Sub-Saharan Africa | Female | 15-49 years | Polycystic ovarian syndrome | Rate | 2003 | 1287.092475 | 1840.127635 | 890.5644987 |
| Prevalence | Western Sub-Saharan Africa | Female | 15-49 years | Polycystic ovarian syndrome | Rate | 2004 | 1297.670683 | 1856.548423 | 897.5423158 |
| Prevalence | Western Sub-Saharan Africa | Female | 15-49 years | Polycystic ovarian syndrome | Rate | 2005 | 1304.751995 | 1869.838954 | 902.2784022 |
| Prevalence | Western Sub-Saharan Africa | Female | 15-49 years | Polycystic ovarian syndrome | Rate | 2006 | 1310.14296 | 1876.552073 | 906.0333427 |
| Prevalence | Western Sub-Saharan Africa | Female | 15-49 years | Polycystic ovarian syndrome | Rate | 2007 | 1315.810575 | 1883.00566 | 910.2595417 |
| Prevalence | Western Sub-Saharan Africa | Female | 15-49 years | Polycystic ovarian syndrome | Rate | 2008 | 1320.968279 | 1888.399853 | 914.3576052 |
| Prevalence | Western Sub-Saharan Africa | Female | 15-49 years | Polycystic ovarian syndrome | Rate | 2009 | 1324.883286 | 1892.023713 | 918.0260878 |
| Prevalence | Western Sub-Saharan Africa | Female | 15-49 years | Polycystic ovarian syndrome | Rate | 2010 | 1326.808618 | 1892.622678 | 919.9647722 |
| Prevalence | Western Sub-Saharan Africa | Female | 15-49 years | Polycystic ovarian syndrome | Rate | 2011 | 1326.864906 | 1892.484479 | 919.7194469 |
| Prevalence | Western Sub-Saharan Africa | Female | 15-49 years | Polycystic ovarian syndrome | Rate | 2012 | 1326.163275 | 1891.262552 | 919.5914824 |
| Prevalence | Western Sub-Saharan Africa | Female | 15-49 years | Polycystic ovarian syndrome | Rate | 2013 | 1325.35252 | 1889.992784 | 919.3115034 |
| Prevalence | Western Sub-Saharan Africa | Female | 15-49 years | Polycystic ovarian syndrome | Rate | 2014 | 1324.975119 | 1885.534417 | 920.1191776 |
| Prevalence | Western Sub-Saharan Africa | Female | 15-49 years | Polycystic ovarian syndrome | Rate | 2015 | 1325.630163 | 1881.837334 | 922.7230899 |
| Prevalence | Western Sub-Saharan Africa | Female | 15-49 years | Polycystic ovarian syndrome | Rate | 2016 | 1328.437375 | 1891.498298 | 927.1922814 |
| Prevalence | Western Sub-Saharan Africa | Female | 15-49 years | Polycystic ovarian syndrome | Rate | 2017 | 1333.931674 | 1898.829319 | 933.1520348 |
| Prevalence | Western Sub-Saharan Africa | Female | 15-49 years | Polycystic ovarian syndrome | Rate | 2018 | 1341.867333 | 1910.393512 | 941.1270867 |
| Prevalence | Western Sub-Saharan Africa | Female | 15-49 years | Polycystic ovarian syndrome | Rate | 2019 | 1352.070294 | 1924.085676 | 946.6001639 |
| Prevalence | Western Sub-Saharan Africa | Female | 15-49 years | Polycystic ovarian syndrome | Rate | 2020 | 1386.945672 | 1971.370472 | 969.0952034 |
| Prevalence | Western Sub-Saharan Africa | Female | 15-49 years | Polycystic ovarian syndrome | Rate | 2021 | 1371.936675 | 1957.592056 | 959.8862587 |
| Prevalence | Eastern Sub-Saharan Africa | Female | 15-49 years | Polycystic ovarian syndrome | Rate | 1990 | 979.7245577 | 1412.066948 | 684.5666319 |
| Prevalence | Eastern Sub-Saharan Africa | Female | 15-49 years | Polycystic ovarian syndrome | Rate | 1991 | 980.1427119 | 1411.318354 | 684.9753091 |
| Prevalence | Eastern Sub-Saharan Africa | Female | 15-49 years | Polycystic ovarian syndrome | Rate | 1992 | 981.9224728 | 1412.569854 | 685.3073883 |
| Prevalence | Eastern Sub-Saharan Africa | Female | 15-49 years | Polycystic ovarian syndrome | Rate | 1993 | 984.8422173 | 1416.325557 | 688.3145597 |
| Prevalence | Eastern Sub-Saharan Africa | Female | 15-49 years | Polycystic ovarian syndrome | Rate | 1994 | 987.8638653 | 1420.219773 | 690.7854397 |
| Prevalence | Eastern Sub-Saharan Africa | Female | 15-49 years | Polycystic ovarian syndrome | Rate | 1995 | 991.2744383 | 1424.680415 | 692.2675794 |
| Prevalence | Eastern Sub-Saharan Africa | Female | 15-49 years | Polycystic ovarian syndrome | Rate | 1996 | 998.6365185 | 1434.117407 | 698.4094149 |
| Prevalence | Eastern Sub-Saharan Africa | Female | 15-49 years | Polycystic ovarian syndrome | Rate | 1997 | 1011.874685 | 1452.332452 | 706.9561388 |
| Prevalence | Eastern Sub-Saharan Africa | Female | 15-49 years | Polycystic ovarian syndrome | Rate | 1998 | 1027.714627 | 1474.381933 | 715.996288 |
| Prevalence | Eastern Sub-Saharan Africa | Female | 15-49 years | Polycystic ovarian syndrome | Rate | 1999 | 1042.893351 | 1495.461755 | 724.6287982 |
| Prevalence | Eastern Sub-Saharan Africa | Female | 15-49 years | Polycystic ovarian syndrome | Rate | 2000 | 1054.252429 | 1511.386021 | 730.8709504 |
| Prevalence | Eastern Sub-Saharan Africa | Female | 15-49 years | Polycystic ovarian syndrome | Rate | 2001 | 1062.915824 | 1526.585072 | 737.4229865 |
| Prevalence | Eastern Sub-Saharan Africa | Female | 15-49 years | Polycystic ovarian syndrome | Rate | 2002 | 1070.802956 | 1539.100658 | 742.5214585 |
| Prevalence | Eastern Sub-Saharan Africa | Female | 15-49 years | Polycystic ovarian syndrome | Rate | 2003 | 1078.03712 | 1546.762386 | 746.9157978 |
| Prevalence | Eastern Sub-Saharan Africa | Female | 15-49 years | Polycystic ovarian syndrome | Rate | 2004 | 1084.880983 | 1553.526131 | 750.9820782 |
| Prevalence | Eastern Sub-Saharan Africa | Female | 15-49 years | Polycystic ovarian syndrome | Rate | 2005 | 1091.680918 | 1560.730346 | 755.7789329 |
| Prevalence | Eastern Sub-Saharan Africa | Female | 15-49 years | Polycystic ovarian syndrome | Rate | 2006 | 1099.441837 | 1572.891872 | 760.8771407 |
| Prevalence | Eastern Sub-Saharan Africa | Female | 15-49 years | Polycystic ovarian syndrome | Rate | 2007 | 1108.516527 | 1586.551394 | 767.4970585 |
| Prevalence | Eastern Sub-Saharan Africa | Female | 15-49 years | Polycystic ovarian syndrome | Rate | 2008 | 1118.261415 | 1600.12809 | 774.9168071 |
| Prevalence | Eastern Sub-Saharan Africa | Female | 15-49 years | Polycystic ovarian syndrome | Rate | 2009 | 1128.058387 | 1614.106744 | 782.9902265 |
| Prevalence | Eastern Sub-Saharan Africa | Female | 15-49 years | Polycystic ovarian syndrome | Rate | 2010 | 1137.301532 | 1628.096218 | 790.7180017 |
| Prevalence | Eastern Sub-Saharan Africa | Female | 15-49 years | Polycystic ovarian syndrome | Rate | 2011 | 1145.977024 | 1641.68425 | 794.7832264 |
| Prevalence | Eastern Sub-Saharan Africa | Female | 15-49 years | Polycystic ovarian syndrome | Rate | 2012 | 1154.660133 | 1655.948074 | 799.5518721 |
| Prevalence | Eastern Sub-Saharan Africa | Female | 15-49 years | Polycystic ovarian syndrome | Rate | 2013 | 1163.63413 | 1670.314941 | 808.3705508 |
| Prevalence | Eastern Sub-Saharan Africa | Female | 15-49 years | Polycystic ovarian syndrome | Rate | 2014 | 1173.258297 | 1684.408305 | 817.5350921 |
| Prevalence | Eastern Sub-Saharan Africa | Female | 15-49 years | Polycystic ovarian syndrome | Rate | 2015 | 1184.020254 | 1703.985787 | 825.3103359 |
| Prevalence | Eastern Sub-Saharan Africa | Female | 15-49 years | Polycystic ovarian syndrome | Rate | 2016 | 1197.653644 | 1722.366695 | 834.6867497 |
| Prevalence | Eastern Sub-Saharan Africa | Female | 15-49 years | Polycystic ovarian syndrome | Rate | 2017 | 1214.157119 | 1744.899233 | 846.8504041 |
| Prevalence | Eastern Sub-Saharan Africa | Female | 15-49 years | Polycystic ovarian syndrome | Rate | 2018 | 1231.265571 | 1762.930305 | 863.3866686 |
| Prevalence | Eastern Sub-Saharan Africa | Female | 15-49 years | Polycystic ovarian syndrome | Rate | 2019 | 1246.843362 | 1785.684695 | 874.1904174 |
| Prevalence | Eastern Sub-Saharan Africa | Female | 15-49 years | Polycystic ovarian syndrome | Rate | 2020 | 1271.423751 | 1811.496413 | 880.2602796 |
| Prevalence | Eastern Sub-Saharan Africa | Female | 15-49 years | Polycystic ovarian syndrome | Rate | 2021 | 1272.440415 | 1817.390259 | 894.0412936 |
| Prevalence | South Asia | Female | 15-49 years | Polycystic ovarian syndrome | Rate | 1990 | 1220.900977 | 1698.946423 | 869.5449389 |
| Prevalence | South Asia | Female | 15-49 years | Polycystic ovarian syndrome | Rate | 1991 | 1232.852362 | 1717.200662 | 879.6679556 |
| Prevalence | South Asia | Female | 15-49 years | Polycystic ovarian syndrome | Rate | 1992 | 1246.109968 | 1737.048028 | 893.8923888 |
| Prevalence | South Asia | Female | 15-49 years | Polycystic ovarian syndrome | Rate | 1993 | 1260.295497 | 1758.038788 | 908.5064064 |
| Prevalence | South Asia | Female | 15-49 years | Polycystic ovarian syndrome | Rate | 1994 | 1274.99048 | 1779.642966 | 920.8540639 |
| Prevalence | South Asia | Female | 15-49 years | Polycystic ovarian syndrome | Rate | 1995 | 1289.943617 | 1805.534194 | 928.9072254 |
| Prevalence | South Asia | Female | 15-49 years | Polycystic ovarian syndrome | Rate | 1996 | 1305.757069 | 1824.188483 | 942.0613805 |
| Prevalence | South Asia | Female | 15-49 years | Polycystic ovarian syndrome | Rate | 1997 | 1323.500848 | 1847.257476 | 956.328513 |
| Prevalence | South Asia | Female | 15-49 years | Polycystic ovarian syndrome | Rate | 1998 | 1342.517149 | 1872.424779 | 969.4737783 |
| Prevalence | South Asia | Female | 15-49 years | Polycystic ovarian syndrome | Rate | 1999 | 1362.081286 | 1901.004576 | 979.576008 |
| Prevalence | South Asia | Female | 15-49 years | Polycystic ovarian syndrome | Rate | 2000 | 1381.610955 | 1925.507932 | 989.5965714 |
| Prevalence | South Asia | Female | 15-49 years | Polycystic ovarian syndrome | Rate | 2001 | 1402.492835 | 1957.117074 | 1004.008971 |
| Prevalence | South Asia | Female | 15-49 years | Polycystic ovarian syndrome | Rate | 2002 | 1425.891833 | 1992.604266 | 1019.012298 |
| Prevalence | South Asia | Female | 15-49 years | Polycystic ovarian syndrome | Rate | 2003 | 1451.736539 | 2030.046409 | 1035.799774 |
| Prevalence | South Asia | Female | 15-49 years | Polycystic ovarian syndrome | Rate | 2004 | 1479.935356 | 2071.799298 | 1054.068104 |
| Prevalence | South Asia | Female | 15-49 years | Polycystic ovarian syndrome | Rate | 2005 | 1510.275064 | 2116.52499 | 1071.067795 |
| Prevalence | South Asia | Female | 15-49 years | Polycystic ovarian syndrome | Rate | 2006 | 1556.350316 | 2171.456648 | 1104.675486 |
| Prevalence | South Asia | Female | 15-49 years | Polycystic ovarian syndrome | Rate | 2007 | 1623.572556 | 2258.451758 | 1150.752136 |
| Prevalence | South Asia | Female | 15-49 years | Polycystic ovarian syndrome | Rate | 2008 | 1699.537515 | 2366.426153 | 1203.442252 |
| Prevalence | South Asia | Female | 15-49 years | Polycystic ovarian syndrome | Rate | 2009 | 1772.083576 | 2469.911695 | 1257.632817 |
| Prevalence | South Asia | Female | 15-49 years | Polycystic ovarian syndrome | Rate | 2010 | 1829.186845 | 2551.624332 | 1294.772 |
| Prevalence | South Asia | Female | 15-49 years | Polycystic ovarian syndrome | Rate | 2011 | 1873.522 | 2601.127803 | 1331.905749 |
| Prevalence | South Asia | Female | 15-49 years | Polycystic ovarian syndrome | Rate | 2012 | 1915.298196 | 2657.497658 | 1366.62477 |
| Prevalence | South Asia | Female | 15-49 years | Polycystic ovarian syndrome | Rate | 2013 | 1954.1657 | 2716.679025 | 1390.170563 |
| Prevalence | South Asia | Female | 15-49 years | Polycystic ovarian syndrome | Rate | 2014 | 1989.831303 | 2772.527141 | 1413.935151 |
| Prevalence | South Asia | Female | 15-49 years | Polycystic ovarian syndrome | Rate | 2015 | 2021.821091 | 2819.375556 | 1438.656216 |
| Prevalence | South Asia | Female | 15-49 years | Polycystic ovarian syndrome | Rate | 2016 | 2053.356386 | 2874.822118 | 1458.193314 |
| Prevalence | South Asia | Female | 15-49 years | Polycystic ovarian syndrome | Rate | 2017 | 2086.43755 | 2928.148957 | 1479.748595 |
| Prevalence | South Asia | Female | 15-49 years | Polycystic ovarian syndrome | Rate | 2018 | 2118.555234 | 2980.230053 | 1496.739603 |
| Prevalence | South Asia | Female | 15-49 years | Polycystic ovarian syndrome | Rate | 2019 | 2147.198469 | 3027.56421 | 1514.55535 |
| Prevalence | South Asia | Female | 15-49 years | Polycystic ovarian syndrome | Rate | 2020 | 2179.021434 | 3059.893619 | 1522.48124 |
| Prevalence | South Asia | Female | 15-49 years | Polycystic ovarian syndrome | Rate | 2021 | 2175.382028 | 3046.402826 | 1537.878737 |
| Prevalence | Southern Sub-Saharan Africa | Female | 15-49 years | Polycystic ovarian syndrome | Rate | 1990 | 1656.995946 | 2384.846225 | 1142.330979 |
| Prevalence | Southern Sub-Saharan Africa | Female | 15-49 years | Polycystic ovarian syndrome | Rate | 1991 | 1661.036935 | 2383.023494 | 1144.452916 |
| Prevalence | Southern Sub-Saharan Africa | Female | 15-49 years | Polycystic ovarian syndrome | Rate | 1992 | 1668.953481 | 2386.346791 | 1151.516315 |
| Prevalence | Southern Sub-Saharan Africa | Female | 15-49 years | Polycystic ovarian syndrome | Rate | 1993 | 1680.67565 | 2396.780139 | 1162.016193 |
| Prevalence | Southern Sub-Saharan Africa | Female | 15-49 years | Polycystic ovarian syndrome | Rate | 1994 | 1696.042877 | 2413.750342 | 1174.78526 |
| Prevalence | Southern Sub-Saharan Africa | Female | 15-49 years | Polycystic ovarian syndrome | Rate | 1995 | 1714.80829 | 2435.543106 | 1187.863916 |
| Prevalence | Southern Sub-Saharan Africa | Female | 15-49 years | Polycystic ovarian syndrome | Rate | 1996 | 1741.622103 | 2465.736704 | 1202.852326 |
| Prevalence | Southern Sub-Saharan Africa | Female | 15-49 years | Polycystic ovarian syndrome | Rate | 1997 | 1778.077311 | 2516.685257 | 1228.263825 |
| Prevalence | Southern Sub-Saharan Africa | Female | 15-49 years | Polycystic ovarian syndrome | Rate | 1998 | 1816.893833 | 2571.081268 | 1254.781999 |
| Prevalence | Southern Sub-Saharan Africa | Female | 15-49 years | Polycystic ovarian syndrome | Rate | 1999 | 1850.929536 | 2623.172338 | 1275.978715 |
| Prevalence | Southern Sub-Saharan Africa | Female | 15-49 years | Polycystic ovarian syndrome | Rate | 2000 | 1872.935842 | 2659.188353 | 1289.284354 |
| Prevalence | Southern Sub-Saharan Africa | Female | 15-49 years | Polycystic ovarian syndrome | Rate | 2001 | 1887.378246 | 2670.212307 | 1300.064846 |
| Prevalence | Southern Sub-Saharan Africa | Female | 15-49 years | Polycystic ovarian syndrome | Rate | 2002 | 1901.755739 | 2685.388081 | 1307.939328 |
| Prevalence | Southern Sub-Saharan Africa | Female | 15-49 years | Polycystic ovarian syndrome | Rate | 2003 | 1915.874942 | 2709.000402 | 1313.424148 |
| Prevalence | Southern Sub-Saharan Africa | Female | 15-49 years | Polycystic ovarian syndrome | Rate | 2004 | 1929.422844 | 2754.007356 | 1320.410304 |
| Prevalence | Southern Sub-Saharan Africa | Female | 15-49 years | Polycystic ovarian syndrome | Rate | 2005 | 1942.026456 | 2782.553901 | 1329.116524 |
| Prevalence | Southern Sub-Saharan Africa | Female | 15-49 years | Polycystic ovarian syndrome | Rate | 2006 | 1956.545895 | 2799.732812 | 1342.382448 |
| Prevalence | Southern Sub-Saharan Africa | Female | 15-49 years | Polycystic ovarian syndrome | Rate | 2007 | 1974.426353 | 2806.686527 | 1357.392289 |
| Prevalence | Southern Sub-Saharan Africa | Female | 15-49 years | Polycystic ovarian syndrome | Rate | 2008 | 1993.249632 | 2823.382819 | 1372.57944 |
| Prevalence | Southern Sub-Saharan Africa | Female | 15-49 years | Polycystic ovarian syndrome | Rate | 2009 | 2010.611476 | 2851.174512 | 1389.872318 |
| Prevalence | Southern Sub-Saharan Africa | Female | 15-49 years | Polycystic ovarian syndrome | Rate | 2010 | 2024.204778 | 2880.578576 | 1400.617602 |
| Prevalence | Southern Sub-Saharan Africa | Female | 15-49 years | Polycystic ovarian syndrome | Rate | 2011 | 2032.448361 | 2885.184634 | 1406.792754 |
| Prevalence | Southern Sub-Saharan Africa | Female | 15-49 years | Polycystic ovarian syndrome | Rate | 2012 | 2036.936458 | 2884.535907 | 1407.083547 |
| Prevalence | Southern Sub-Saharan Africa | Female | 15-49 years | Polycystic ovarian syndrome | Rate | 2013 | 2039.944725 | 2881.887361 | 1405.646382 |
| Prevalence | Southern Sub-Saharan Africa | Female | 15-49 years | Polycystic ovarian syndrome | Rate | 2014 | 2043.548274 | 2881.068791 | 1409.350496 |
| Prevalence | Southern Sub-Saharan Africa | Female | 15-49 years | Polycystic ovarian syndrome | Rate | 2015 | 2049.60377 | 2887.421407 | 1417.305427 |
| Prevalence | Southern Sub-Saharan Africa | Female | 15-49 years | Polycystic ovarian syndrome | Rate | 2016 | 2060.146753 | 2898.253414 | 1420.774944 |
| Prevalence | Southern Sub-Saharan Africa | Female | 15-49 years | Polycystic ovarian syndrome | Rate | 2017 | 2074.285988 | 2919.723137 | 1429.198757 |
| Prevalence | Southern Sub-Saharan Africa | Female | 15-49 years | Polycystic ovarian syndrome | Rate | 2018 | 2089.552085 | 2939.167847 | 1433.28369 |
| Prevalence | Southern Sub-Saharan Africa | Female | 15-49 years | Polycystic ovarian syndrome | Rate | 2019 | 2103.428156 | 2955.699251 | 1439.902093 |
| Prevalence | Southern Sub-Saharan Africa | Female | 15-49 years | Polycystic ovarian syndrome | Rate | 2020 | 2124.596811 | 2995.42712 | 1463.707343 |
| Prevalence | Southern Sub-Saharan Africa | Female | 15-49 years | Polycystic ovarian syndrome | Rate | 2021 | 2103.877038 | 2969.206143 | 1439.468524 |
