# Supplementary material for: Global and Chinese epidemiologic study of polycystic ovary syndrome in women of childbearing age, 1990–2021, and projections to 2035: Based on the Global Burden of Disease 2021 study
Source: PLoS One. 2025 Aug 19;20(8):e0329090. doi: 10.1371/journal.pone.0329090 (PMC12364318; doi:10.1371/journal.pone.0329090)
Supplement: S3 Table — (DOCX) [file pone.0329090.s003.docx]

| **Supplementary Table 3** Global and Regional DALYs of Polycystic Ovarian Syndrome Among Women Aged 15-49 Years: A Comprehensive Analysis from 1990 to 2021 | | | | | | | | | |
| --- | --- | --- | --- | --- | --- | --- | --- | --- | --- |
| **Measure** | **Location** | **Sex** | **Age** | **Cause** | **Metric** | **Year** | **Value** | **Upper** | **Lower** |
| DALYs | Global | Female | 15-49 years | Polycystic ovarian syndrome | Rate | 1990 | 23.02625289 | 48.02207485 | 10.22829911 |
| DALYs | Global | Female | 15-49 years | Polycystic ovarian syndrome | Rate | 1991 | 23.25213828 | 48.4854387 | 10.33665222 |
| DALYs | Global | Female | 15-49 years | Polycystic ovarian syndrome | Rate | 1992 | 23.47866882 | 48.94533476 | 10.43992413 |
| DALYs | Global | Female | 15-49 years | Polycystic ovarian syndrome | Rate | 1993 | 23.69857324 | 49.34077384 | 10.55453779 |
| DALYs | Global | Female | 15-49 years | Polycystic ovarian syndrome | Rate | 1994 | 23.90259014 | 49.76481689 | 10.64045348 |
| DALYs | Global | Female | 15-49 years | Polycystic ovarian syndrome | Rate | 1995 | 24.09611886 | 50.24145543 | 10.72459687 |
| DALYs | Global | Female | 15-49 years | Polycystic ovarian syndrome | Rate | 1996 | 24.32840463 | 50.76872569 | 10.82469732 |
| DALYs | Global | Female | 15-49 years | Polycystic ovarian syndrome | Rate | 1997 | 24.59666848 | 51.36940523 | 10.93148878 |
| DALYs | Global | Female | 15-49 years | Polycystic ovarian syndrome | Rate | 1998 | 24.8731172 | 52.02863261 | 11.09241536 |
| DALYs | Global | Female | 15-49 years | Polycystic ovarian syndrome | Rate | 1999 | 25.13306934 | 52.60555257 | 11.21906596 |
| DALYs | Global | Female | 15-49 years | Polycystic ovarian syndrome | Rate | 2000 | 25.31700879 | 53.25858334 | 11.29639817 |
| DALYs | Global | Female | 15-49 years | Polycystic ovarian syndrome | Rate | 2001 | 25.47627429 | 53.46553364 | 11.39374288 |
| DALYs | Global | Female | 15-49 years | Polycystic ovarian syndrome | Rate | 2002 | 25.64618384 | 53.9127878 | 11.51423049 |
| DALYs | Global | Female | 15-49 years | Polycystic ovarian syndrome | Rate | 2003 | 25.81583966 | 54.25120831 | 11.58782678 |
| DALYs | Global | Female | 15-49 years | Polycystic ovarian syndrome | Rate | 2004 | 25.96974161 | 54.56842172 | 11.67631476 |
| DALYs | Global | Female | 15-49 years | Polycystic ovarian syndrome | Rate | 2005 | 26.09708875 | 54.75903375 | 11.7326791 |
| DALYs | Global | Female | 15-49 years | Polycystic ovarian syndrome | Rate | 2006 | 26.17493725 | 54.83816591 | 11.80768686 |
| DALYs | Global | Female | 15-49 years | Polycystic ovarian syndrome | Rate | 2007 | 26.22951952 | 54.91667922 | 11.86261534 |
| DALYs | Global | Female | 15-49 years | Polycystic ovarian syndrome | Rate | 2008 | 26.2941761 | 55.07870519 | 11.89087874 |
| DALYs | Global | Female | 15-49 years | Polycystic ovarian syndrome | Rate | 2009 | 26.39251967 | 55.31077488 | 11.94614588 |
| DALYs | Global | Female | 15-49 years | Polycystic ovarian syndrome | Rate | 2010 | 26.54723453 | 55.62890229 | 11.98126511 |
| DALYs | Global | Female | 15-49 years | Polycystic ovarian syndrome | Rate | 2011 | 26.76351475 | 56.00619687 | 12.10142835 |
| DALYs | Global | Female | 15-49 years | Polycystic ovarian syndrome | Rate | 2012 | 27.0136048 | 56.49559917 | 12.20873053 |
| DALYs | Global | Female | 15-49 years | Polycystic ovarian syndrome | Rate | 2013 | 27.27921002 | 57.09222064 | 12.30055856 |
| DALYs | Global | Female | 15-49 years | Polycystic ovarian syndrome | Rate | 2014 | 27.53359689 | 57.64114029 | 12.4202327 |
| DALYs | Global | Female | 15-49 years | Polycystic ovarian syndrome | Rate | 2015 | 27.75096119 | 58.05349706 | 12.50914189 |
| DALYs | Global | Female | 15-49 years | Polycystic ovarian syndrome | Rate | 2016 | 28.01432167 | 58.62258958 | 12.64177133 |
| DALYs | Global | Female | 15-49 years | Polycystic ovarian syndrome | Rate | 2017 | 28.34978131 | 59.16056727 | 12.76653928 |
| DALYs | Global | Female | 15-49 years | Polycystic ovarian syndrome | Rate | 2018 | 28.70920043 | 59.88720523 | 12.87881926 |
| DALYs | Global | Female | 15-49 years | Polycystic ovarian syndrome | Rate | 2019 | 29.02716134 | 60.50918048 | 13.00019129 |
| DALYs | Global | Female | 15-49 years | Polycystic ovarian syndrome | Rate | 2020 | 29.39683859 | 61.39656021 | 13.17116638 |
| DALYs | Global | Female | 15-49 years | Polycystic ovarian syndrome | Rate | 2021 | 29.55812998 | 61.66412841 | 13.24059002 |
| DALYs | Australasia | Female | 15-49 years | Polycystic ovarian syndrome | Rate | 1990 | 69.10699199 | 143.5188101 | 31.45713549 |
| DALYs | Australasia | Female | 15-49 years | Polycystic ovarian syndrome | Rate | 1991 | 70.44210804 | 145.5059262 | 31.77715529 |
| DALYs | Australasia | Female | 15-49 years | Polycystic ovarian syndrome | Rate | 1992 | 71.56117332 | 147.676083 | 32.61273837 |
| DALYs | Australasia | Female | 15-49 years | Polycystic ovarian syndrome | Rate | 1993 | 72.72442618 | 150.7596901 | 33.32818165 |
| DALYs | Australasia | Female | 15-49 years | Polycystic ovarian syndrome | Rate | 1994 | 73.7175894 | 152.8408941 | 33.63131454 |
| DALYs | Australasia | Female | 15-49 years | Polycystic ovarian syndrome | Rate | 1995 | 74.52023316 | 153.9807612 | 34.3464011 |
| DALYs | Australasia | Female | 15-49 years | Polycystic ovarian syndrome | Rate | 1996 | 75.28335384 | 156.4714726 | 34.72701298 |
| DALYs | Australasia | Female | 15-49 years | Polycystic ovarian syndrome | Rate | 1997 | 76.03899538 | 157.6733461 | 35.02094492 |
| DALYs | Australasia | Female | 15-49 years | Polycystic ovarian syndrome | Rate | 1998 | 76.64265792 | 156.85991 | 35.81482232 |
| DALYs | Australasia | Female | 15-49 years | Polycystic ovarian syndrome | Rate | 1999 | 77.06808752 | 158.3606399 | 36.16485939 |
| DALYs | Australasia | Female | 15-49 years | Polycystic ovarian syndrome | Rate | 2000 | 77.37536397 | 157.7288697 | 36.70463588 |
| DALYs | Australasia | Female | 15-49 years | Polycystic ovarian syndrome | Rate | 2001 | 77.59420841 | 158.7766042 | 37.27352499 |
| DALYs | Australasia | Female | 15-49 years | Polycystic ovarian syndrome | Rate | 2002 | 77.68150261 | 154.9577708 | 37.58311383 |
| DALYs | Australasia | Female | 15-49 years | Polycystic ovarian syndrome | Rate | 2003 | 77.72174148 | 153.6440385 | 37.79218174 |
| DALYs | Australasia | Female | 15-49 years | Polycystic ovarian syndrome | Rate | 2004 | 77.69346597 | 151.1824579 | 37.9013289 |
| DALYs | Australasia | Female | 15-49 years | Polycystic ovarian syndrome | Rate | 2005 | 77.61502197 | 151.1329815 | 37.87560586 |
| DALYs | Australasia | Female | 15-49 years | Polycystic ovarian syndrome | Rate | 2006 | 77.42744513 | 149.7754758 | 38.08788043 |
| DALYs | Australasia | Female | 15-49 years | Polycystic ovarian syndrome | Rate | 2007 | 77.28183579 | 151.5748203 | 38.08186687 |
| DALYs | Australasia | Female | 15-49 years | Polycystic ovarian syndrome | Rate | 2008 | 77.08991025 | 151.4033303 | 37.48073311 |
| DALYs | Australasia | Female | 15-49 years | Polycystic ovarian syndrome | Rate | 2009 | 76.96798705 | 152.4132236 | 37.50509979 |
| DALYs | Australasia | Female | 15-49 years | Polycystic ovarian syndrome | Rate | 2010 | 76.95802182 | 152.1898699 | 37.26317793 |
| DALYs | Australasia | Female | 15-49 years | Polycystic ovarian syndrome | Rate | 2011 | 76.92420668 | 154.4871607 | 37.18639057 |
| DALYs | Australasia | Female | 15-49 years | Polycystic ovarian syndrome | Rate | 2012 | 76.83886901 | 157.0461034 | 37.10524219 |
| DALYs | Australasia | Female | 15-49 years | Polycystic ovarian syndrome | Rate | 2013 | 76.75875631 | 157.8402019 | 36.2894385 |
| DALYs | Australasia | Female | 15-49 years | Polycystic ovarian syndrome | Rate | 2014 | 76.75685191 | 158.1325771 | 35.64500065 |
| DALYs | Australasia | Female | 15-49 years | Polycystic ovarian syndrome | Rate | 2015 | 76.86891586 | 159.3300919 | 35.17567588 |
| DALYs | Australasia | Female | 15-49 years | Polycystic ovarian syndrome | Rate | 2016 | 77.19353656 | 160.110152 | 34.72049388 |
| DALYs | Australasia | Female | 15-49 years | Polycystic ovarian syndrome | Rate | 2017 | 77.70103409 | 160.1938061 | 34.66054934 |
| DALYs | Australasia | Female | 15-49 years | Polycystic ovarian syndrome | Rate | 2018 | 78.3491459 | 161.6579046 | 34.48117744 |
| DALYs | Australasia | Female | 15-49 years | Polycystic ovarian syndrome | Rate | 2019 | 78.78427862 | 162.5344016 | 34.25500954 |
| DALYs | Australasia | Female | 15-49 years | Polycystic ovarian syndrome | Rate | 2020 | 79.72372635 | 164.8805165 | 35.50211808 |
| DALYs | Australasia | Female | 15-49 years | Polycystic ovarian syndrome | Rate | 2021 | 80.22868362 | 166.5004864 | 36.03549564 |
| DALYs | Caribbean | Female | 15-49 years | Polycystic ovarian syndrome | Rate | 1990 | 20.13173018 | 41.91795529 | 8.734932535 |
| DALYs | Caribbean | Female | 15-49 years | Polycystic ovarian syndrome | Rate | 1991 | 20.37201659 | 42.90735199 | 8.947128665 |
| DALYs | Caribbean | Female | 15-49 years | Polycystic ovarian syndrome | Rate | 1992 | 20.59852067 | 43.19409902 | 9.058518045 |
| DALYs | Caribbean | Female | 15-49 years | Polycystic ovarian syndrome | Rate | 1993 | 20.82024737 | 43.98636133 | 9.248169027 |
| DALYs | Caribbean | Female | 15-49 years | Polycystic ovarian syndrome | Rate | 1994 | 20.97609904 | 43.88498385 | 9.302919202 |
| DALYs | Caribbean | Female | 15-49 years | Polycystic ovarian syndrome | Rate | 1995 | 21.1459531 | 44.41555184 | 9.487548546 |
| DALYs | Caribbean | Female | 15-49 years | Polycystic ovarian syndrome | Rate | 1996 | 21.28291057 | 45.06379072 | 9.530890375 |
| DALYs | Caribbean | Female | 15-49 years | Polycystic ovarian syndrome | Rate | 1997 | 21.42295352 | 44.83593649 | 9.556221073 |
| DALYs | Caribbean | Female | 15-49 years | Polycystic ovarian syndrome | Rate | 1998 | 21.54068203 | 45.48740661 | 9.555567191 |
| DALYs | Caribbean | Female | 15-49 years | Polycystic ovarian syndrome | Rate | 1999 | 21.68350282 | 45.31734521 | 9.527205094 |
| DALYs | Caribbean | Female | 15-49 years | Polycystic ovarian syndrome | Rate | 2000 | 21.829018 | 45.99415462 | 9.603023897 |
| DALYs | Caribbean | Female | 15-49 years | Polycystic ovarian syndrome | Rate | 2001 | 22.04284168 | 46.53296401 | 9.625013937 |
| DALYs | Caribbean | Female | 15-49 years | Polycystic ovarian syndrome | Rate | 2002 | 22.3403856 | 47.04973917 | 9.854566184 |
| DALYs | Caribbean | Female | 15-49 years | Polycystic ovarian syndrome | Rate | 2003 | 22.67814693 | 48.12852459 | 10.0664805 |
| DALYs | Caribbean | Female | 15-49 years | Polycystic ovarian syndrome | Rate | 2004 | 23.00222614 | 48.98906654 | 10.14734025 |
| DALYs | Caribbean | Female | 15-49 years | Polycystic ovarian syndrome | Rate | 2005 | 23.25557051 | 49.63105737 | 10.24620819 |
| DALYs | Caribbean | Female | 15-49 years | Polycystic ovarian syndrome | Rate | 2006 | 23.46911884 | 50.09598406 | 10.27515583 |
| DALYs | Caribbean | Female | 15-49 years | Polycystic ovarian syndrome | Rate | 2007 | 23.69718171 | 50.30974725 | 10.46300877 |
| DALYs | Caribbean | Female | 15-49 years | Polycystic ovarian syndrome | Rate | 2008 | 23.90742548 | 50.71820302 | 10.50149478 |
| DALYs | Caribbean | Female | 15-49 years | Polycystic ovarian syndrome | Rate | 2009 | 24.07639776 | 50.62399752 | 10.55560112 |
| DALYs | Caribbean | Female | 15-49 years | Polycystic ovarian syndrome | Rate | 2010 | 24.19643263 | 51.01837285 | 10.66708321 |
| DALYs | Caribbean | Female | 15-49 years | Polycystic ovarian syndrome | Rate | 2011 | 24.29247941 | 51.48111716 | 10.57088255 |
| DALYs | Caribbean | Female | 15-49 years | Polycystic ovarian syndrome | Rate | 2012 | 24.3407008 | 51.91566997 | 10.60459341 |
| DALYs | Caribbean | Female | 15-49 years | Polycystic ovarian syndrome | Rate | 2013 | 24.3850745 | 51.70002637 | 10.70594606 |
| DALYs | Caribbean | Female | 15-49 years | Polycystic ovarian syndrome | Rate | 2014 | 24.43192956 | 52.17713878 | 10.67936683 |
| DALYs | Caribbean | Female | 15-49 years | Polycystic ovarian syndrome | Rate | 2015 | 24.46908761 | 52.03569872 | 10.6561725 |
| DALYs | Caribbean | Female | 15-49 years | Polycystic ovarian syndrome | Rate | 2016 | 24.52719069 | 52.40768371 | 10.76076555 |
| DALYs | Caribbean | Female | 15-49 years | Polycystic ovarian syndrome | Rate | 2017 | 24.53909467 | 51.7619406 | 10.89953467 |
| DALYs | Caribbean | Female | 15-49 years | Polycystic ovarian syndrome | Rate | 2018 | 24.59701068 | 51.53170494 | 10.86468458 |
| DALYs | Caribbean | Female | 15-49 years | Polycystic ovarian syndrome | Rate | 2019 | 24.70756633 | 51.85428488 | 10.9585247 |
| DALYs | Caribbean | Female | 15-49 years | Polycystic ovarian syndrome | Rate | 2020 | 24.93866865 | 51.89216913 | 11.08954543 |
| DALYs | Caribbean | Female | 15-49 years | Polycystic ovarian syndrome | Rate | 2021 | 24.78868134 | 52.31853619 | 10.76295382 |
| DALYs | Central Asia | Female | 15-49 years | Polycystic ovarian syndrome | Rate | 1990 | 5.903245089 | 12.64163148 | 2.457664823 |
| DALYs | Central Asia | Female | 15-49 years | Polycystic ovarian syndrome | Rate | 1991 | 5.992240453 | 12.81925394 | 2.530253703 |
| DALYs | Central Asia | Female | 15-49 years | Polycystic ovarian syndrome | Rate | 1992 | 6.083567863 | 13.14089863 | 2.551186678 |
| DALYs | Central Asia | Female | 15-49 years | Polycystic ovarian syndrome | Rate | 1993 | 6.157177924 | 13.19781311 | 2.551089915 |
| DALYs | Central Asia | Female | 15-49 years | Polycystic ovarian syndrome | Rate | 1994 | 6.207420474 | 13.15916899 | 2.612635722 |
| DALYs | Central Asia | Female | 15-49 years | Polycystic ovarian syndrome | Rate | 1995 | 6.244500928 | 13.39692292 | 2.626511593 |
| DALYs | Central Asia | Female | 15-49 years | Polycystic ovarian syndrome | Rate | 1996 | 6.274967084 | 13.53092761 | 2.640493621 |
| DALYs | Central Asia | Female | 15-49 years | Polycystic ovarian syndrome | Rate | 1997 | 6.294456867 | 13.70264849 | 2.622444928 |
| DALYs | Central Asia | Female | 15-49 years | Polycystic ovarian syndrome | Rate | 1998 | 6.314035294 | 13.75445786 | 2.668345042 |
| DALYs | Central Asia | Female | 15-49 years | Polycystic ovarian syndrome | Rate | 1999 | 6.338039775 | 13.8679279 | 2.689180816 |
| DALYs | Central Asia | Female | 15-49 years | Polycystic ovarian syndrome | Rate | 2000 | 6.363599476 | 13.48165411 | 2.721526322 |
| DALYs | Central Asia | Female | 15-49 years | Polycystic ovarian syndrome | Rate | 2001 | 6.398831445 | 13.72987007 | 2.703166571 |
| DALYs | Central Asia | Female | 15-49 years | Polycystic ovarian syndrome | Rate | 2002 | 6.45203392 | 13.88033422 | 2.728192765 |
| DALYs | Central Asia | Female | 15-49 years | Polycystic ovarian syndrome | Rate | 2003 | 6.508269056 | 14.03825675 | 2.7591472 |
| DALYs | Central Asia | Female | 15-49 years | Polycystic ovarian syndrome | Rate | 2004 | 6.585730902 | 14.07936874 | 2.741020592 |
| DALYs | Central Asia | Female | 15-49 years | Polycystic ovarian syndrome | Rate | 2005 | 6.650811113 | 14.27841205 | 2.822637372 |
| DALYs | Central Asia | Female | 15-49 years | Polycystic ovarian syndrome | Rate | 2006 | 6.740976093 | 14.44518982 | 2.823354199 |
| DALYs | Central Asia | Female | 15-49 years | Polycystic ovarian syndrome | Rate | 2007 | 6.839295406 | 14.75246805 | 2.874190221 |
| DALYs | Central Asia | Female | 15-49 years | Polycystic ovarian syndrome | Rate | 2008 | 6.970818534 | 15.01820734 | 2.973002228 |
| DALYs | Central Asia | Female | 15-49 years | Polycystic ovarian syndrome | Rate | 2009 | 7.084365742 | 15.2146232 | 3.017187108 |
| DALYs | Central Asia | Female | 15-49 years | Polycystic ovarian syndrome | Rate | 2010 | 7.206377422 | 15.44672974 | 3.039766263 |
| DALYs | Central Asia | Female | 15-49 years | Polycystic ovarian syndrome | Rate | 2011 | 7.34761007 | 15.44093202 | 3.125743279 |
| DALYs | Central Asia | Female | 15-49 years | Polycystic ovarian syndrome | Rate | 2012 | 7.488148248 | 16.0434239 | 3.193755783 |
| DALYs | Central Asia | Female | 15-49 years | Polycystic ovarian syndrome | Rate | 2013 | 7.631109593 | 16.3138708 | 3.242955203 |
| DALYs | Central Asia | Female | 15-49 years | Polycystic ovarian syndrome | Rate | 2014 | 7.76468464 | 16.69702544 | 3.341393693 |
| DALYs | Central Asia | Female | 15-49 years | Polycystic ovarian syndrome | Rate | 2015 | 7.904193363 | 16.92579594 | 3.34616368 |
| DALYs | Central Asia | Female | 15-49 years | Polycystic ovarian syndrome | Rate | 2016 | 8.007009006 | 17.27024967 | 3.425633074 |
| DALYs | Central Asia | Female | 15-49 years | Polycystic ovarian syndrome | Rate | 2017 | 8.082866817 | 17.43147996 | 3.400140373 |
| DALYs | Central Asia | Female | 15-49 years | Polycystic ovarian syndrome | Rate | 2018 | 8.168073536 | 17.76857518 | 3.467228858 |
| DALYs | Central Asia | Female | 15-49 years | Polycystic ovarian syndrome | Rate | 2019 | 8.224499235 | 17.72628427 | 3.451724853 |
| DALYs | Central Asia | Female | 15-49 years | Polycystic ovarian syndrome | Rate | 2020 | 8.271188647 | 17.73761402 | 3.516423595 |
| DALYs | Central Asia | Female | 15-49 years | Polycystic ovarian syndrome | Rate | 2021 | 8.129074327 | 17.54997891 | 3.429504111 |
| DALYs | Central Europe | Female | 15-49 years | Polycystic ovarian syndrome | Rate | 1990 | 3.105694809 | 6.463993078 | 1.300550856 |
| DALYs | Central Europe | Female | 15-49 years | Polycystic ovarian syndrome | Rate | 1991 | 3.12777206 | 6.496415771 | 1.339647242 |
| DALYs | Central Europe | Female | 15-49 years | Polycystic ovarian syndrome | Rate | 1992 | 3.153824279 | 6.568731155 | 1.32387444 |
| DALYs | Central Europe | Female | 15-49 years | Polycystic ovarian syndrome | Rate | 1993 | 3.180254224 | 6.621164378 | 1.342081602 |
| DALYs | Central Europe | Female | 15-49 years | Polycystic ovarian syndrome | Rate | 1994 | 3.204282628 | 6.705217842 | 1.338151389 |
| DALYs | Central Europe | Female | 15-49 years | Polycystic ovarian syndrome | Rate | 1995 | 3.234269735 | 6.727962259 | 1.368579476 |
| DALYs | Central Europe | Female | 15-49 years | Polycystic ovarian syndrome | Rate | 1996 | 3.261444231 | 6.865636819 | 1.3635738 |
| DALYs | Central Europe | Female | 15-49 years | Polycystic ovarian syndrome | Rate | 1997 | 3.290977416 | 6.802344669 | 1.390187342 |
| DALYs | Central Europe | Female | 15-49 years | Polycystic ovarian syndrome | Rate | 1998 | 3.326921748 | 6.874729689 | 1.395466679 |
| DALYs | Central Europe | Female | 15-49 years | Polycystic ovarian syndrome | Rate | 1999 | 3.357192957 | 7.030450054 | 1.400172271 |
| DALYs | Central Europe | Female | 15-49 years | Polycystic ovarian syndrome | Rate | 2000 | 3.386475884 | 7.095281359 | 1.417199336 |
| DALYs | Central Europe | Female | 15-49 years | Polycystic ovarian syndrome | Rate | 2001 | 3.410213547 | 7.211291629 | 1.429952348 |
| DALYs | Central Europe | Female | 15-49 years | Polycystic ovarian syndrome | Rate | 2002 | 3.435216708 | 7.240986085 | 1.457365629 |
| DALYs | Central Europe | Female | 15-49 years | Polycystic ovarian syndrome | Rate | 2003 | 3.46109377 | 7.200494972 | 1.451655855 |
| DALYs | Central Europe | Female | 15-49 years | Polycystic ovarian syndrome | Rate | 2004 | 3.49065115 | 7.273263947 | 1.477518162 |
| DALYs | Central Europe | Female | 15-49 years | Polycystic ovarian syndrome | Rate | 2005 | 3.512925899 | 7.244968459 | 1.484477202 |
| DALYs | Central Europe | Female | 15-49 years | Polycystic ovarian syndrome | Rate | 2006 | 3.545624585 | 7.324895628 | 1.483877138 |
| DALYs | Central Europe | Female | 15-49 years | Polycystic ovarian syndrome | Rate | 2007 | 3.578534343 | 7.439327783 | 1.506880619 |
| DALYs | Central Europe | Female | 15-49 years | Polycystic ovarian syndrome | Rate | 2008 | 3.606164643 | 7.52051771 | 1.514334438 |
| DALYs | Central Europe | Female | 15-49 years | Polycystic ovarian syndrome | Rate | 2009 | 3.62970031 | 7.587354725 | 1.529478759 |
| DALYs | Central Europe | Female | 15-49 years | Polycystic ovarian syndrome | Rate | 2010 | 3.641654417 | 7.593098284 | 1.533771202 |
| DALYs | Central Europe | Female | 15-49 years | Polycystic ovarian syndrome | Rate | 2011 | 3.649647177 | 7.53229914 | 1.544984815 |
| DALYs | Central Europe | Female | 15-49 years | Polycystic ovarian syndrome | Rate | 2012 | 3.653169235 | 7.532379193 | 1.550301505 |
| DALYs | Central Europe | Female | 15-49 years | Polycystic ovarian syndrome | Rate | 2013 | 3.647702777 | 7.604092295 | 1.556242768 |
| DALYs | Central Europe | Female | 15-49 years | Polycystic ovarian syndrome | Rate | 2014 | 3.64620444 | 7.648534215 | 1.579995238 |
| DALYs | Central Europe | Female | 15-49 years | Polycystic ovarian syndrome | Rate | 2015 | 3.652077416 | 7.575538187 | 1.578646091 |
| DALYs | Central Europe | Female | 15-49 years | Polycystic ovarian syndrome | Rate | 2016 | 3.666740834 | 7.678546015 | 1.559954581 |
| DALYs | Central Europe | Female | 15-49 years | Polycystic ovarian syndrome | Rate | 2017 | 3.688642389 | 7.65332345 | 1.58973601 |
| DALYs | Central Europe | Female | 15-49 years | Polycystic ovarian syndrome | Rate | 2018 | 3.711340839 | 7.846935462 | 1.60334158 |
| DALYs | Central Europe | Female | 15-49 years | Polycystic ovarian syndrome | Rate | 2019 | 3.73210479 | 7.722419709 | 1.612997317 |
| DALYs | Central Europe | Female | 15-49 years | Polycystic ovarian syndrome | Rate | 2020 | 3.75669503 | 7.83089039 | 1.624290464 |
| DALYs | Central Europe | Female | 15-49 years | Polycystic ovarian syndrome | Rate | 2021 | 3.783584853 | 7.95219601 | 1.618494665 |
| DALYs | Central Latin America | Female | 15-49 years | Polycystic ovarian syndrome | Rate | 1990 | 44.6501127 | 93.82158697 | 19.7944713 |
| DALYs | Central Latin America | Female | 15-49 years | Polycystic ovarian syndrome | Rate | 1991 | 46.35980779 | 97.0954986 | 20.4675902 |
| DALYs | Central Latin America | Female | 15-49 years | Polycystic ovarian syndrome | Rate | 1992 | 47.77007243 | 99.79005345 | 21.17069116 |
| DALYs | Central Latin America | Female | 15-49 years | Polycystic ovarian syndrome | Rate | 1993 | 48.84523276 | 102.2361742 | 21.67650009 |
| DALYs | Central Latin America | Female | 15-49 years | Polycystic ovarian syndrome | Rate | 1994 | 49.57111062 | 103.5594657 | 21.85179781 |
| DALYs | Central Latin America | Female | 15-49 years | Polycystic ovarian syndrome | Rate | 1995 | 49.89162544 | 104.2792265 | 21.9549146 |
| DALYs | Central Latin America | Female | 15-49 years | Polycystic ovarian syndrome | Rate | 1996 | 49.77835242 | 104.1188134 | 21.74452706 |
| DALYs | Central Latin America | Female | 15-49 years | Polycystic ovarian syndrome | Rate | 1997 | 49.30245354 | 103.5212437 | 21.78432099 |
| DALYs | Central Latin America | Female | 15-49 years | Polycystic ovarian syndrome | Rate | 1998 | 48.60512367 | 102.1710705 | 21.38386078 |
| DALYs | Central Latin America | Female | 15-49 years | Polycystic ovarian syndrome | Rate | 1999 | 47.87876774 | 101.131641 | 21.08956942 |
| DALYs | Central Latin America | Female | 15-49 years | Polycystic ovarian syndrome | Rate | 2000 | 47.26256974 | 100.1790411 | 20.68708624 |
| DALYs | Central Latin America | Female | 15-49 years | Polycystic ovarian syndrome | Rate | 2001 | 46.74496466 | 99.36928083 | 20.6460775 |
| DALYs | Central Latin America | Female | 15-49 years | Polycystic ovarian syndrome | Rate | 2002 | 46.22022486 | 97.92491752 | 20.46266449 |
| DALYs | Central Latin America | Female | 15-49 years | Polycystic ovarian syndrome | Rate | 2003 | 45.71390594 | 96.9038452 | 20.23437183 |
| DALYs | Central Latin America | Female | 15-49 years | Polycystic ovarian syndrome | Rate | 2004 | 45.25273117 | 95.76941061 | 20.07936763 |
| DALYs | Central Latin America | Female | 15-49 years | Polycystic ovarian syndrome | Rate | 2005 | 44.89168165 | 95.34774652 | 19.99317316 |
| DALYs | Central Latin America | Female | 15-49 years | Polycystic ovarian syndrome | Rate | 2006 | 44.53738672 | 94.38858606 | 19.83802348 |
| DALYs | Central Latin America | Female | 15-49 years | Polycystic ovarian syndrome | Rate | 2007 | 44.15240703 | 93.83231946 | 19.59134761 |
| DALYs | Central Latin America | Female | 15-49 years | Polycystic ovarian syndrome | Rate | 2008 | 43.82966195 | 93.03709737 | 19.33288695 |
| DALYs | Central Latin America | Female | 15-49 years | Polycystic ovarian syndrome | Rate | 2009 | 43.61386354 | 92.51943941 | 19.28635812 |
| DALYs | Central Latin America | Female | 15-49 years | Polycystic ovarian syndrome | Rate | 2010 | 43.58581721 | 92.66469695 | 19.11852934 |
| DALYs | Central Latin America | Female | 15-49 years | Polycystic ovarian syndrome | Rate | 2011 | 43.73892231 | 92.97529301 | 19.25979381 |
| DALYs | Central Latin America | Female | 15-49 years | Polycystic ovarian syndrome | Rate | 2012 | 44.02414597 | 94.11118702 | 19.35178013 |
| DALYs | Central Latin America | Female | 15-49 years | Polycystic ovarian syndrome | Rate | 2013 | 44.37042825 | 94.52841342 | 19.51496189 |
| DALYs | Central Latin America | Female | 15-49 years | Polycystic ovarian syndrome | Rate | 2014 | 44.77819791 | 95.69990062 | 19.71398783 |
| DALYs | Central Latin America | Female | 15-49 years | Polycystic ovarian syndrome | Rate | 2015 | 45.17885585 | 96.56074281 | 19.78230561 |
| DALYs | Central Latin America | Female | 15-49 years | Polycystic ovarian syndrome | Rate | 2016 | 45.85607991 | 97.88560525 | 20.04638608 |
| DALYs | Central Latin America | Female | 15-49 years | Polycystic ovarian syndrome | Rate | 2017 | 46.86828226 | 99.98733774 | 20.45799381 |
| DALYs | Central Latin America | Female | 15-49 years | Polycystic ovarian syndrome | Rate | 2018 | 47.87161007 | 101.7820301 | 20.87423189 |
| DALYs | Central Latin America | Female | 15-49 years | Polycystic ovarian syndrome | Rate | 2019 | 48.4885576 | 102.770703 | 21.21651786 |
| DALYs | Central Latin America | Female | 15-49 years | Polycystic ovarian syndrome | Rate | 2020 | 48.64032742 | 103.0734804 | 21.31502949 |
| DALYs | Central Latin America | Female | 15-49 years | Polycystic ovarian syndrome | Rate | 2021 | 48.43481717 | 101.4404644 | 21.05908928 |
| DALYs | Central Sub-Saharan Africa | Female | 15-49 years | Polycystic ovarian syndrome | Rate | 1990 | 7.324625771 | 15.15363667 | 3.108818604 |
| DALYs | Central Sub-Saharan Africa | Female | 15-49 years | Polycystic ovarian syndrome | Rate | 1991 | 7.364273892 | 15.82112796 | 3.104018656 |
| DALYs | Central Sub-Saharan Africa | Female | 15-49 years | Polycystic ovarian syndrome | Rate | 1992 | 7.411222209 | 15.6379921 | 3.125461787 |
| DALYs | Central Sub-Saharan Africa | Female | 15-49 years | Polycystic ovarian syndrome | Rate | 1993 | 7.489323114 | 16.24598438 | 3.147029724 |
| DALYs | Central Sub-Saharan Africa | Female | 15-49 years | Polycystic ovarian syndrome | Rate | 1994 | 7.564316362 | 16.23508094 | 3.256641749 |
| DALYs | Central Sub-Saharan Africa | Female | 15-49 years | Polycystic ovarian syndrome | Rate | 1995 | 7.668580298 | 16.35094588 | 3.232942358 |
| DALYs | Central Sub-Saharan Africa | Female | 15-49 years | Polycystic ovarian syndrome | Rate | 1996 | 7.905956819 | 16.8599041 | 3.310771412 |
| DALYs | Central Sub-Saharan Africa | Female | 15-49 years | Polycystic ovarian syndrome | Rate | 1997 | 8.327034818 | 17.8667576 | 3.579027249 |
| DALYs | Central Sub-Saharan Africa | Female | 15-49 years | Polycystic ovarian syndrome | Rate | 1998 | 8.803585903 | 18.9865958 | 3.764379459 |
| DALYs | Central Sub-Saharan Africa | Female | 15-49 years | Polycystic ovarian syndrome | Rate | 1999 | 9.211738356 | 19.62052254 | 3.960984947 |
| DALYs | Central Sub-Saharan Africa | Female | 15-49 years | Polycystic ovarian syndrome | Rate | 2000 | 9.39764127 | 20.14963356 | 4.016033004 |
| DALYs | Central Sub-Saharan Africa | Female | 15-49 years | Polycystic ovarian syndrome | Rate | 2001 | 9.424001461 | 20.07423151 | 3.9610752 |
| DALYs | Central Sub-Saharan Africa | Female | 15-49 years | Polycystic ovarian syndrome | Rate | 2002 | 9.43724628 | 19.97638911 | 4.050309913 |
| DALYs | Central Sub-Saharan Africa | Female | 15-49 years | Polycystic ovarian syndrome | Rate | 2003 | 9.421798834 | 20.37404007 | 4.058677993 |
| DALYs | Central Sub-Saharan Africa | Female | 15-49 years | Polycystic ovarian syndrome | Rate | 2004 | 9.43027631 | 20.42283676 | 4.0490376 |
| DALYs | Central Sub-Saharan Africa | Female | 15-49 years | Polycystic ovarian syndrome | Rate | 2005 | 9.428428707 | 20.00765011 | 3.980500483 |
| DALYs | Central Sub-Saharan Africa | Female | 15-49 years | Polycystic ovarian syndrome | Rate | 2006 | 9.450193544 | 19.33449937 | 4.030202589 |
| DALYs | Central Sub-Saharan Africa | Female | 15-49 years | Polycystic ovarian syndrome | Rate | 2007 | 9.47886373 | 19.48709308 | 4.145812108 |
| DALYs | Central Sub-Saharan Africa | Female | 15-49 years | Polycystic ovarian syndrome | Rate | 2008 | 9.49933654 | 19.98830467 | 4.173538606 |
| DALYs | Central Sub-Saharan Africa | Female | 15-49 years | Polycystic ovarian syndrome | Rate | 2009 | 9.545270213 | 20.50846074 | 4.05715264 |
| DALYs | Central Sub-Saharan Africa | Female | 15-49 years | Polycystic ovarian syndrome | Rate | 2010 | 9.590499229 | 19.81223975 | 4.118239829 |
| DALYs | Central Sub-Saharan Africa | Female | 15-49 years | Polycystic ovarian syndrome | Rate | 2011 | 9.683058496 | 20.10185062 | 4.176746444 |
| DALYs | Central Sub-Saharan Africa | Female | 15-49 years | Polycystic ovarian syndrome | Rate | 2012 | 9.78998808 | 20.1995572 | 4.17053139 |
| DALYs | Central Sub-Saharan Africa | Female | 15-49 years | Polycystic ovarian syndrome | Rate | 2013 | 9.930019315 | 20.81234199 | 4.182538224 |
| DALYs | Central Sub-Saharan Africa | Female | 15-49 years | Polycystic ovarian syndrome | Rate | 2014 | 10.09224179 | 21.20723114 | 4.292579866 |
| DALYs | Central Sub-Saharan Africa | Female | 15-49 years | Polycystic ovarian syndrome | Rate | 2015 | 10.24401022 | 21.82310223 | 4.363745019 |
| DALYs | Central Sub-Saharan Africa | Female | 15-49 years | Polycystic ovarian syndrome | Rate | 2016 | 10.35038512 | 22.28147258 | 4.432110381 |
| DALYs | Central Sub-Saharan Africa | Female | 15-49 years | Polycystic ovarian syndrome | Rate | 2017 | 10.48240878 | 22.23078432 | 4.503969722 |
| DALYs | Central Sub-Saharan Africa | Female | 15-49 years | Polycystic ovarian syndrome | Rate | 2018 | 10.6560054 | 22.6121417 | 4.566549568 |
| DALYs | Central Sub-Saharan Africa | Female | 15-49 years | Polycystic ovarian syndrome | Rate | 2019 | 10.83616741 | 22.8406239 | 4.617085018 |
| DALYs | Central Sub-Saharan Africa | Female | 15-49 years | Polycystic ovarian syndrome | Rate | 2020 | 11.2225574 | 23.93333951 | 4.874711464 |
| DALYs | Central Sub-Saharan Africa | Female | 15-49 years | Polycystic ovarian syndrome | Rate | 2021 | 11.15035065 | 22.90451811 | 4.811164299 |
| DALYs | East Asia | Female | 15-49 years | Polycystic ovarian syndrome | Rate | 1990 | 13.89388834 | 28.80039301 | 5.96725893 |
| DALYs | East Asia | Female | 15-49 years | Polycystic ovarian syndrome | Rate | 1991 | 14.05347971 | 29.25985603 | 6.004459433 |
| DALYs | East Asia | Female | 15-49 years | Polycystic ovarian syndrome | Rate | 1992 | 14.29302129 | 29.66782557 | 6.117391339 |
| DALYs | East Asia | Female | 15-49 years | Polycystic ovarian syndrome | Rate | 1993 | 14.60368253 | 30.33392647 | 6.299503389 |
| DALYs | East Asia | Female | 15-49 years | Polycystic ovarian syndrome | Rate | 1994 | 14.94222858 | 31.02466805 | 6.476573159 |
| DALYs | East Asia | Female | 15-49 years | Polycystic ovarian syndrome | Rate | 1995 | 15.30699845 | 31.92911798 | 6.602771703 |
| DALYs | East Asia | Female | 15-49 years | Polycystic ovarian syndrome | Rate | 1996 | 15.79891255 | 33.09908708 | 6.816579348 |
| DALYs | East Asia | Female | 15-49 years | Polycystic ovarian syndrome | Rate | 1997 | 16.48717688 | 34.35671026 | 7.152822202 |
| DALYs | East Asia | Female | 15-49 years | Polycystic ovarian syndrome | Rate | 1998 | 17.2366306 | 35.74598763 | 7.468173042 |
| DALYs | East Asia | Female | 15-49 years | Polycystic ovarian syndrome | Rate | 1999 | 17.96885769 | 37.36870885 | 7.762018694 |
| DALYs | East Asia | Female | 15-49 years | Polycystic ovarian syndrome | Rate | 2000 | 18.5405419 | 38.61578029 | 8.035374629 |
| DALYs | East Asia | Female | 15-49 years | Polycystic ovarian syndrome | Rate | 2001 | 18.9940345 | 39.35912135 | 8.211706883 |
| DALYs | East Asia | Female | 15-49 years | Polycystic ovarian syndrome | Rate | 2002 | 19.43284336 | 40.32904399 | 8.359859412 |
| DALYs | East Asia | Female | 15-49 years | Polycystic ovarian syndrome | Rate | 2003 | 19.84664419 | 41.15409276 | 8.568216587 |
| DALYs | East Asia | Female | 15-49 years | Polycystic ovarian syndrome | Rate | 2004 | 20.2327744 | 42.0461341 | 8.776769938 |
| DALYs | East Asia | Female | 15-49 years | Polycystic ovarian syndrome | Rate | 2005 | 20.58478566 | 42.50839186 | 8.906360942 |
| DALYs | East Asia | Female | 15-49 years | Polycystic ovarian syndrome | Rate | 2006 | 20.88638435 | 43.41138503 | 9.039725521 |
| DALYs | East Asia | Female | 15-49 years | Polycystic ovarian syndrome | Rate | 2007 | 21.13501978 | 44.12821808 | 9.119849778 |
| DALYs | East Asia | Female | 15-49 years | Polycystic ovarian syndrome | Rate | 2008 | 21.37814043 | 44.81647575 | 9.169583494 |
| DALYs | East Asia | Female | 15-49 years | Polycystic ovarian syndrome | Rate | 2009 | 21.61643676 | 45.44079984 | 9.247809272 |
| DALYs | East Asia | Female | 15-49 years | Polycystic ovarian syndrome | Rate | 2010 | 21.86988595 | 46.03956887 | 9.386605074 |
| DALYs | East Asia | Female | 15-49 years | Polycystic ovarian syndrome | Rate | 2011 | 22.18070148 | 46.48094267 | 9.502980716 |
| DALYs | East Asia | Female | 15-49 years | Polycystic ovarian syndrome | Rate | 2012 | 22.51802759 | 47.24402251 | 9.686570925 |
| DALYs | East Asia | Female | 15-49 years | Polycystic ovarian syndrome | Rate | 2013 | 22.88714675 | 48.05062323 | 9.843618924 |
| DALYs | East Asia | Female | 15-49 years | Polycystic ovarian syndrome | Rate | 2014 | 23.25455115 | 48.86761598 | 10.04324888 |
| DALYs | East Asia | Female | 15-49 years | Polycystic ovarian syndrome | Rate | 2015 | 23.62198141 | 49.77089729 | 10.21676857 |
| DALYs | East Asia | Female | 15-49 years | Polycystic ovarian syndrome | Rate | 2016 | 24.01157917 | 50.37191548 | 10.39035103 |
| DALYs | East Asia | Female | 15-49 years | Polycystic ovarian syndrome | Rate | 2017 | 24.43620403 | 51.10019537 | 10.52114146 |
| DALYs | East Asia | Female | 15-49 years | Polycystic ovarian syndrome | Rate | 2018 | 24.83142038 | 51.94115689 | 10.68851964 |
| DALYs | East Asia | Female | 15-49 years | Polycystic ovarian syndrome | Rate | 2019 | 25.13575767 | 52.34592958 | 10.82138872 |
| DALYs | East Asia | Female | 15-49 years | Polycystic ovarian syndrome | Rate | 2020 | 25.3091413 | 53.06117196 | 10.88113929 |
| DALYs | East Asia | Female | 15-49 years | Polycystic ovarian syndrome | Rate | 2021 | 25.59968223 | 53.06619474 | 11.20179266 |
| DALYs | Eastern Europe | Female | 15-49 years | Polycystic ovarian syndrome | Rate | 1990 | 3.557089665 | 7.494665319 | 1.471334569 |
| DALYs | Eastern Europe | Female | 15-49 years | Polycystic ovarian syndrome | Rate | 1991 | 3.577611 | 7.540562908 | 1.476502965 |
| DALYs | Eastern Europe | Female | 15-49 years | Polycystic ovarian syndrome | Rate | 1992 | 3.593911938 | 7.626819274 | 1.494185835 |
| DALYs | Eastern Europe | Female | 15-49 years | Polycystic ovarian syndrome | Rate | 1993 | 3.60527342 | 7.634499947 | 1.487883753 |
| DALYs | Eastern Europe | Female | 15-49 years | Polycystic ovarian syndrome | Rate | 1994 | 3.61788784 | 7.71626621 | 1.487034265 |
| DALYs | Eastern Europe | Female | 15-49 years | Polycystic ovarian syndrome | Rate | 1995 | 3.628492323 | 7.727698758 | 1.501916423 |
| DALYs | Eastern Europe | Female | 15-49 years | Polycystic ovarian syndrome | Rate | 1996 | 3.638775135 | 7.791416316 | 1.493820199 |
| DALYs | Eastern Europe | Female | 15-49 years | Polycystic ovarian syndrome | Rate | 1997 | 3.65511736 | 7.690843436 | 1.522981705 |
| DALYs | Eastern Europe | Female | 15-49 years | Polycystic ovarian syndrome | Rate | 1998 | 3.676400581 | 7.773269431 | 1.524959606 |
| DALYs | Eastern Europe | Female | 15-49 years | Polycystic ovarian syndrome | Rate | 1999 | 3.700267588 | 7.674986714 | 1.524421134 |
| DALYs | Eastern Europe | Female | 15-49 years | Polycystic ovarian syndrome | Rate | 2000 | 3.724274097 | 7.758276044 | 1.552074155 |
| DALYs | Eastern Europe | Female | 15-49 years | Polycystic ovarian syndrome | Rate | 2001 | 3.747602626 | 7.850002724 | 1.553248274 |
| DALYs | Eastern Europe | Female | 15-49 years | Polycystic ovarian syndrome | Rate | 2002 | 3.777594771 | 8.003561385 | 1.579408197 |
| DALYs | Eastern Europe | Female | 15-49 years | Polycystic ovarian syndrome | Rate | 2003 | 3.813939599 | 8.118416314 | 1.582578342 |
| DALYs | Eastern Europe | Female | 15-49 years | Polycystic ovarian syndrome | Rate | 2004 | 3.853735685 | 8.244562208 | 1.603209082 |
| DALYs | Eastern Europe | Female | 15-49 years | Polycystic ovarian syndrome | Rate | 2005 | 3.899451807 | 8.351197884 | 1.612781662 |
| DALYs | Eastern Europe | Female | 15-49 years | Polycystic ovarian syndrome | Rate | 2006 | 3.95413351 | 8.406694865 | 1.636111671 |
| DALYs | Eastern Europe | Female | 15-49 years | Polycystic ovarian syndrome | Rate | 2007 | 4.022898993 | 8.595287543 | 1.667670513 |
| DALYs | Eastern Europe | Female | 15-49 years | Polycystic ovarian syndrome | Rate | 2008 | 4.095171454 | 8.634195844 | 1.714599698 |
| DALYs | Eastern Europe | Female | 15-49 years | Polycystic ovarian syndrome | Rate | 2009 | 4.170180875 | 8.892339291 | 1.727442384 |
| DALYs | Eastern Europe | Female | 15-49 years | Polycystic ovarian syndrome | Rate | 2010 | 4.229587827 | 8.854627392 | 1.745864239 |
| DALYs | Eastern Europe | Female | 15-49 years | Polycystic ovarian syndrome | Rate | 2011 | 4.291791538 | 9.028103372 | 1.770276939 |
| DALYs | Eastern Europe | Female | 15-49 years | Polycystic ovarian syndrome | Rate | 2012 | 4.343167688 | 9.171650279 | 1.794889456 |
| DALYs | Eastern Europe | Female | 15-49 years | Polycystic ovarian syndrome | Rate | 2013 | 4.39410564 | 9.221338703 | 1.807816826 |
| DALYs | Eastern Europe | Female | 15-49 years | Polycystic ovarian syndrome | Rate | 2014 | 4.436675935 | 9.26562392 | 1.855269819 |
| DALYs | Eastern Europe | Female | 15-49 years | Polycystic ovarian syndrome | Rate | 2015 | 4.480140825 | 9.340375183 | 1.862675207 |
| DALYs | Eastern Europe | Female | 15-49 years | Polycystic ovarian syndrome | Rate | 2016 | 4.529836119 | 9.415186091 | 1.888031141 |
| DALYs | Eastern Europe | Female | 15-49 years | Polycystic ovarian syndrome | Rate | 2017 | 4.583819057 | 9.572374364 | 1.907180116 |
| DALYs | Eastern Europe | Female | 15-49 years | Polycystic ovarian syndrome | Rate | 2018 | 4.62777546 | 9.601091947 | 1.935025186 |
| DALYs | Eastern Europe | Female | 15-49 years | Polycystic ovarian syndrome | Rate | 2019 | 4.65214783 | 9.623005731 | 1.943866416 |
| DALYs | Eastern Europe | Female | 15-49 years | Polycystic ovarian syndrome | Rate | 2020 | 4.652172576 | 9.726589292 | 1.936260671 |
| DALYs | Eastern Europe | Female | 15-49 years | Polycystic ovarian syndrome | Rate | 2021 | 4.558111411 | 9.556177367 | 1.889089582 |
| DALYs | Eastern Sub-Saharan Africa | Female | 15-49 years | Polycystic ovarian syndrome | Rate | 1990 | 8.541444231 | 17.92148998 | 3.576758762 |
| DALYs | Eastern Sub-Saharan Africa | Female | 15-49 years | Polycystic ovarian syndrome | Rate | 1991 | 8.554176378 | 17.96132348 | 3.635892068 |
| DALYs | Eastern Sub-Saharan Africa | Female | 15-49 years | Polycystic ovarian syndrome | Rate | 1992 | 8.576673388 | 17.92680302 | 3.671092458 |
| DALYs | Eastern Sub-Saharan Africa | Female | 15-49 years | Polycystic ovarian syndrome | Rate | 1993 | 8.606471076 | 18.19412104 | 3.65373273 |
| DALYs | Eastern Sub-Saharan Africa | Female | 15-49 years | Polycystic ovarian syndrome | Rate | 1994 | 8.639473982 | 18.25504158 | 3.648844137 |
| DALYs | Eastern Sub-Saharan Africa | Female | 15-49 years | Polycystic ovarian syndrome | Rate | 1995 | 8.676240973 | 18.23295692 | 3.688719862 |
| DALYs | Eastern Sub-Saharan Africa | Female | 15-49 years | Polycystic ovarian syndrome | Rate | 1996 | 8.741038971 | 18.44573271 | 3.721839029 |
| DALYs | Eastern Sub-Saharan Africa | Female | 15-49 years | Polycystic ovarian syndrome | Rate | 1997 | 8.86770377 | 18.8073706 | 3.78541997 |
| DALYs | Eastern Sub-Saharan Africa | Female | 15-49 years | Polycystic ovarian syndrome | Rate | 1998 | 9.006162613 | 18.98382218 | 3.838183465 |
| DALYs | Eastern Sub-Saharan Africa | Female | 15-49 years | Polycystic ovarian syndrome | Rate | 1999 | 9.141834809 | 19.32030769 | 3.91075714 |
| DALYs | Eastern Sub-Saharan Africa | Female | 15-49 years | Polycystic ovarian syndrome | Rate | 2000 | 9.238994922 | 19.63098261 | 3.953772821 |
| DALYs | Eastern Sub-Saharan Africa | Female | 15-49 years | Polycystic ovarian syndrome | Rate | 2001 | 9.31645183 | 19.77305223 | 3.965801398 |
| DALYs | Eastern Sub-Saharan Africa | Female | 15-49 years | Polycystic ovarian syndrome | Rate | 2002 | 9.390433538 | 19.70784938 | 4.02149468 |
| DALYs | Eastern Sub-Saharan Africa | Female | 15-49 years | Polycystic ovarian syndrome | Rate | 2003 | 9.457399542 | 20.04892313 | 4.000766675 |
| DALYs | Eastern Sub-Saharan Africa | Female | 15-49 years | Polycystic ovarian syndrome | Rate | 2004 | 9.518115214 | 20.14325847 | 4.081171536 |
| DALYs | Eastern Sub-Saharan Africa | Female | 15-49 years | Polycystic ovarian syndrome | Rate | 2005 | 9.58284882 | 20.30241332 | 4.14749791 |
| DALYs | Eastern Sub-Saharan Africa | Female | 15-49 years | Polycystic ovarian syndrome | Rate | 2006 | 9.640682502 | 20.37328466 | 4.127148305 |
| DALYs | Eastern Sub-Saharan Africa | Female | 15-49 years | Polycystic ovarian syndrome | Rate | 2007 | 9.713663325 | 20.63560267 | 4.129264448 |
| DALYs | Eastern Sub-Saharan Africa | Female | 15-49 years | Polycystic ovarian syndrome | Rate | 2008 | 9.781617498 | 20.98379171 | 4.204859851 |
| DALYs | Eastern Sub-Saharan Africa | Female | 15-49 years | Polycystic ovarian syndrome | Rate | 2009 | 9.847327586 | 20.97189148 | 4.172503326 |
| DALYs | Eastern Sub-Saharan Africa | Female | 15-49 years | Polycystic ovarian syndrome | Rate | 2010 | 9.923033995 | 21.10485237 | 4.237220242 |
| DALYs | Eastern Sub-Saharan Africa | Female | 15-49 years | Polycystic ovarian syndrome | Rate | 2011 | 9.996614768 | 21.27005656 | 4.281926053 |
| DALYs | Eastern Sub-Saharan Africa | Female | 15-49 years | Polycystic ovarian syndrome | Rate | 2012 | 10.07078201 | 21.37624926 | 4.276429545 |
| DALYs | Eastern Sub-Saharan Africa | Female | 15-49 years | Polycystic ovarian syndrome | Rate | 2013 | 10.14154737 | 21.41365815 | 4.331371445 |
| DALYs | Eastern Sub-Saharan Africa | Female | 15-49 years | Polycystic ovarian syndrome | Rate | 2014 | 10.21731747 | 21.48415273 | 4.353833347 |
| DALYs | Eastern Sub-Saharan Africa | Female | 15-49 years | Polycystic ovarian syndrome | Rate | 2015 | 10.30882788 | 21.61914156 | 4.401527608 |
| DALYs | Eastern Sub-Saharan Africa | Female | 15-49 years | Polycystic ovarian syndrome | Rate | 2016 | 10.4270212 | 22.01243572 | 4.472189784 |
| DALYs | Eastern Sub-Saharan Africa | Female | 15-49 years | Polycystic ovarian syndrome | Rate | 2017 | 10.56238018 | 22.1164572 | 4.504656661 |
| DALYs | Eastern Sub-Saharan Africa | Female | 15-49 years | Polycystic ovarian syndrome | Rate | 2018 | 10.70353902 | 22.64587961 | 4.574442809 |
| DALYs | Eastern Sub-Saharan Africa | Female | 15-49 years | Polycystic ovarian syndrome | Rate | 2019 | 10.83889691 | 22.96488603 | 4.662338881 |
| DALYs | Eastern Sub-Saharan Africa | Female | 15-49 years | Polycystic ovarian syndrome | Rate | 2020 | 11.07215105 | 23.41077593 | 4.729589176 |
| DALYs | Eastern Sub-Saharan Africa | Female | 15-49 years | Polycystic ovarian syndrome | Rate | 2021 | 11.05657208 | 23.37949395 | 4.731015722 |
| DALYs | Andean Latin America | Female | 15-49 years | Polycystic ovarian syndrome | Rate | 1990 | 39.75148288 | 86.05576182 | 17.74341796 |
| DALYs | Andean Latin America | Female | 15-49 years | Polycystic ovarian syndrome | Rate | 1991 | 40.03045357 | 86.09789972 | 17.9249329 |
| DALYs | Andean Latin America | Female | 15-49 years | Polycystic ovarian syndrome | Rate | 1992 | 40.36226595 | 87.37458793 | 18.0056794 |
| DALYs | Andean Latin America | Female | 15-49 years | Polycystic ovarian syndrome | Rate | 1993 | 40.76771455 | 88.08278248 | 18.14813586 |
| DALYs | Andean Latin America | Female | 15-49 years | Polycystic ovarian syndrome | Rate | 1994 | 41.33015144 | 89.54127857 | 18.37571607 |
| DALYs | Andean Latin America | Female | 15-49 years | Polycystic ovarian syndrome | Rate | 1995 | 41.92095214 | 91.23867944 | 18.56898848 |
| DALYs | Andean Latin America | Female | 15-49 years | Polycystic ovarian syndrome | Rate | 1996 | 42.77586855 | 92.57560707 | 19.10067562 |
| DALYs | Andean Latin America | Female | 15-49 years | Polycystic ovarian syndrome | Rate | 1997 | 43.88767315 | 94.94059908 | 19.73402024 |
| DALYs | Andean Latin America | Female | 15-49 years | Polycystic ovarian syndrome | Rate | 1998 | 45.05127685 | 97.62434065 | 19.93769748 |
| DALYs | Andean Latin America | Female | 15-49 years | Polycystic ovarian syndrome | Rate | 1999 | 46.01317914 | 99.12571058 | 20.63272135 |
| DALYs | Andean Latin America | Female | 15-49 years | Polycystic ovarian syndrome | Rate | 2000 | 46.51674784 | 100.5880001 | 20.90370977 |
| DALYs | Andean Latin America | Female | 15-49 years | Polycystic ovarian syndrome | Rate | 2001 | 46.7359422 | 100.8109884 | 21.14881546 |
| DALYs | Andean Latin America | Female | 15-49 years | Polycystic ovarian syndrome | Rate | 2002 | 46.96400704 | 100.0147901 | 20.84093901 |
| DALYs | Andean Latin America | Female | 15-49 years | Polycystic ovarian syndrome | Rate | 2003 | 47.21161877 | 100.191866 | 21.08266676 |
| DALYs | Andean Latin America | Female | 15-49 years | Polycystic ovarian syndrome | Rate | 2004 | 47.41326253 | 102.0361823 | 21.38588328 |
| DALYs | Andean Latin America | Female | 15-49 years | Polycystic ovarian syndrome | Rate | 2005 | 47.63044223 | 102.6699496 | 21.34629074 |
| DALYs | Andean Latin America | Female | 15-49 years | Polycystic ovarian syndrome | Rate | 2006 | 48.07033831 | 103.6140338 | 21.43240012 |
| DALYs | Andean Latin America | Female | 15-49 years | Polycystic ovarian syndrome | Rate | 2007 | 48.8693188 | 104.1987805 | 21.86405055 |
| DALYs | Andean Latin America | Female | 15-49 years | Polycystic ovarian syndrome | Rate | 2008 | 49.80312163 | 105.3252059 | 21.98691877 |
| DALYs | Andean Latin America | Female | 15-49 years | Polycystic ovarian syndrome | Rate | 2009 | 50.70910689 | 107.2372636 | 22.77106238 |
| DALYs | Andean Latin America | Female | 15-49 years | Polycystic ovarian syndrome | Rate | 2010 | 51.29748416 | 106.8577055 | 23.00915416 |
| DALYs | Andean Latin America | Female | 15-49 years | Polycystic ovarian syndrome | Rate | 2011 | 51.64937173 | 109.0315197 | 23.08598188 |
| DALYs | Andean Latin America | Female | 15-49 years | Polycystic ovarian syndrome | Rate | 2012 | 51.90585829 | 109.6435301 | 23.38550663 |
| DALYs | Andean Latin America | Female | 15-49 years | Polycystic ovarian syndrome | Rate | 2013 | 52.0963967 | 109.6188085 | 23.20380652 |
| DALYs | Andean Latin America | Female | 15-49 years | Polycystic ovarian syndrome | Rate | 2014 | 52.32067776 | 110.782793 | 23.03947169 |
| DALYs | Andean Latin America | Female | 15-49 years | Polycystic ovarian syndrome | Rate | 2015 | 52.52472144 | 110.9913168 | 23.03209617 |
| DALYs | Andean Latin America | Female | 15-49 years | Polycystic ovarian syndrome | Rate | 2016 | 52.86850501 | 112.8287652 | 23.29826331 |
| DALYs | Andean Latin America | Female | 15-49 years | Polycystic ovarian syndrome | Rate | 2017 | 53.32904276 | 112.8229951 | 23.52202672 |
| DALYs | Andean Latin America | Female | 15-49 years | Polycystic ovarian syndrome | Rate | 2018 | 53.79873864 | 116.12225 | 24.10127852 |
| DALYs | Andean Latin America | Female | 15-49 years | Polycystic ovarian syndrome | Rate | 2019 | 54.25110445 | 116.8047776 | 24.36351369 |
| DALYs | Andean Latin America | Female | 15-49 years | Polycystic ovarian syndrome | Rate | 2020 | 54.74748498 | 119.2345045 | 24.55616872 |
| DALYs | Andean Latin America | Female | 15-49 years | Polycystic ovarian syndrome | Rate | 2021 | 54.70014257 | 114.5895814 | 24.0182136 |
| DALYs | High-income Asia Pacific | Female | 15-49 years | Polycystic ovarian syndrome | Rate | 1990 | 79.90952539 | 161.8673166 | 35.29997426 |
| DALYs | High-income Asia Pacific | Female | 15-49 years | Polycystic ovarian syndrome | Rate | 1991 | 80.55684671 | 162.825479 | 35.46320347 |
| DALYs | High-income Asia Pacific | Female | 15-49 years | Polycystic ovarian syndrome | Rate | 1992 | 81.12342252 | 163.924318 | 35.60112581 |
| DALYs | High-income Asia Pacific | Female | 15-49 years | Polycystic ovarian syndrome | Rate | 1993 | 81.6714966 | 164.3867632 | 35.87277562 |
| DALYs | High-income Asia Pacific | Female | 15-49 years | Polycystic ovarian syndrome | Rate | 1994 | 82.15465099 | 165.0718234 | 36.07620943 |
| DALYs | High-income Asia Pacific | Female | 15-49 years | Polycystic ovarian syndrome | Rate | 1995 | 82.52974655 | 165.8629072 | 36.09547807 |
| DALYs | High-income Asia Pacific | Female | 15-49 years | Polycystic ovarian syndrome | Rate | 1996 | 83.00976472 | 166.7603846 | 36.2695097 |
| DALYs | High-income Asia Pacific | Female | 15-49 years | Polycystic ovarian syndrome | Rate | 1997 | 83.54280391 | 167.9627218 | 36.55981252 |
| DALYs | High-income Asia Pacific | Female | 15-49 years | Polycystic ovarian syndrome | Rate | 1998 | 84.08103981 | 169.4371086 | 37.06104309 |
| DALYs | High-income Asia Pacific | Female | 15-49 years | Polycystic ovarian syndrome | Rate | 1999 | 84.53211055 | 170.5490614 | 37.23336477 |
| DALYs | High-income Asia Pacific | Female | 15-49 years | Polycystic ovarian syndrome | Rate | 2000 | 84.82467329 | 171.8096135 | 37.53662339 |
| DALYs | High-income Asia Pacific | Female | 15-49 years | Polycystic ovarian syndrome | Rate | 2001 | 85.02387141 | 172.0991541 | 38.12570439 |
| DALYs | High-income Asia Pacific | Female | 15-49 years | Polycystic ovarian syndrome | Rate | 2002 | 85.36856765 | 173.4727541 | 38.86176745 |
| DALYs | High-income Asia Pacific | Female | 15-49 years | Polycystic ovarian syndrome | Rate | 2003 | 85.65839161 | 174.6800673 | 39.39126306 |
| DALYs | High-income Asia Pacific | Female | 15-49 years | Polycystic ovarian syndrome | Rate | 2004 | 85.80831127 | 175.376568 | 40.08633395 |
| DALYs | High-income Asia Pacific | Female | 15-49 years | Polycystic ovarian syndrome | Rate | 2005 | 85.85006525 | 176.3180338 | 40.34377755 |
| DALYs | High-income Asia Pacific | Female | 15-49 years | Polycystic ovarian syndrome | Rate | 2006 | 85.58856305 | 175.1438829 | 40.31408373 |
| DALYs | High-income Asia Pacific | Female | 15-49 years | Polycystic ovarian syndrome | Rate | 2007 | 85.13632727 | 174.2993639 | 40.16149263 |
| DALYs | High-income Asia Pacific | Female | 15-49 years | Polycystic ovarian syndrome | Rate | 2008 | 84.59932434 | 172.9802655 | 39.90454191 |
| DALYs | High-income Asia Pacific | Female | 15-49 years | Polycystic ovarian syndrome | Rate | 2009 | 84.11312666 | 171.7871017 | 39.67730151 |
| DALYs | High-income Asia Pacific | Female | 15-49 years | Polycystic ovarian syndrome | Rate | 2010 | 83.78575307 | 170.715798 | 39.59646455 |
| DALYs | High-income Asia Pacific | Female | 15-49 years | Polycystic ovarian syndrome | Rate | 2011 | 84.02561286 | 172.086644 | 39.47988659 |
| DALYs | High-income Asia Pacific | Female | 15-49 years | Polycystic ovarian syndrome | Rate | 2012 | 84.7603016 | 173.7122678 | 39.71364003 |
| DALYs | High-income Asia Pacific | Female | 15-49 years | Polycystic ovarian syndrome | Rate | 2013 | 85.72128984 | 176.1018576 | 39.648075 |
| DALYs | High-income Asia Pacific | Female | 15-49 years | Polycystic ovarian syndrome | Rate | 2014 | 86.56663797 | 177.6487267 | 39.74853061 |
| DALYs | High-income Asia Pacific | Female | 15-49 years | Polycystic ovarian syndrome | Rate | 2015 | 86.97468576 | 178.8687343 | 39.61373355 |
| DALYs | High-income Asia Pacific | Female | 15-49 years | Polycystic ovarian syndrome | Rate | 2016 | 87.21108979 | 179.3985663 | 39.67351635 |
| DALYs | High-income Asia Pacific | Female | 15-49 years | Polycystic ovarian syndrome | Rate | 2017 | 87.66312886 | 180.5272763 | 39.89034479 |
| DALYs | High-income Asia Pacific | Female | 15-49 years | Polycystic ovarian syndrome | Rate | 2018 | 88.17466515 | 181.0848539 | 40.18303916 |
| DALYs | High-income Asia Pacific | Female | 15-49 years | Polycystic ovarian syndrome | Rate | 2019 | 88.57421432 | 182.0474042 | 40.1642057 |
| DALYs | High-income Asia Pacific | Female | 15-49 years | Polycystic ovarian syndrome | Rate | 2020 | 88.91489373 | 181.7108652 | 40.37932283 |
| DALYs | High-income Asia Pacific | Female | 15-49 years | Polycystic ovarian syndrome | Rate | 2021 | 88.65362948 | 179.9325359 | 39.90171618 |
| DALYs | High-income North America | Female | 15-49 years | Polycystic ovarian syndrome | Rate | 1990 | 51.66023311 | 107.4973337 | 22.85299382 |
| DALYs | High-income North America | Female | 15-49 years | Polycystic ovarian syndrome | Rate | 1991 | 52.05499834 | 108.6147201 | 22.97890295 |
| DALYs | High-income North America | Female | 15-49 years | Polycystic ovarian syndrome | Rate | 1992 | 52.34047891 | 109.1832647 | 23.13999142 |
| DALYs | High-income North America | Female | 15-49 years | Polycystic ovarian syndrome | Rate | 1993 | 52.57091307 | 109.5430761 | 23.13254414 |
| DALYs | High-income North America | Female | 15-49 years | Polycystic ovarian syndrome | Rate | 1994 | 52.7751479 | 109.3431883 | 23.07199659 |
| DALYs | High-income North America | Female | 15-49 years | Polycystic ovarian syndrome | Rate | 1995 | 52.91704702 | 109.4513587 | 23.19946732 |
| DALYs | High-income North America | Female | 15-49 years | Polycystic ovarian syndrome | Rate | 1996 | 53.11230654 | 110.0174361 | 23.23017739 |
| DALYs | High-income North America | Female | 15-49 years | Polycystic ovarian syndrome | Rate | 1997 | 53.43471897 | 111.5152721 | 23.65328638 |
| DALYs | High-income North America | Female | 15-49 years | Polycystic ovarian syndrome | Rate | 1998 | 53.729413 | 112.2938539 | 24.02686982 |
| DALYs | High-income North America | Female | 15-49 years | Polycystic ovarian syndrome | Rate | 1999 | 53.91564359 | 113.4333833 | 24.28177569 |
| DALYs | High-income North America | Female | 15-49 years | Polycystic ovarian syndrome | Rate | 2000 | 53.86477906 | 113.613748 | 24.31421382 |
| DALYs | High-income North America | Female | 15-49 years | Polycystic ovarian syndrome | Rate | 2001 | 53.53856806 | 112.907423 | 24.40204719 |
| DALYs | High-income North America | Female | 15-49 years | Polycystic ovarian syndrome | Rate | 2002 | 52.9774616 | 112.3898071 | 24.46112961 |
| DALYs | High-income North America | Female | 15-49 years | Polycystic ovarian syndrome | Rate | 2003 | 52.25027683 | 111.298595 | 24.3331441 |
| DALYs | High-income North America | Female | 15-49 years | Polycystic ovarian syndrome | Rate | 2004 | 51.38170079 | 109.5712076 | 23.95005196 |
| DALYs | High-income North America | Female | 15-49 years | Polycystic ovarian syndrome | Rate | 2005 | 50.44982489 | 107.0008319 | 23.59463608 |
| DALYs | High-income North America | Female | 15-49 years | Polycystic ovarian syndrome | Rate | 2006 | 48.57710179 | 102.0794267 | 22.77741532 |
| DALYs | High-income North America | Female | 15-49 years | Polycystic ovarian syndrome | Rate | 2007 | 45.42783231 | 96.5422812 | 21.46125213 |
| DALYs | High-income North America | Female | 15-49 years | Polycystic ovarian syndrome | Rate | 2008 | 42.00534495 | 86.76163938 | 19.69854269 |
| DALYs | High-income North America | Female | 15-49 years | Polycystic ovarian syndrome | Rate | 2009 | 39.24744491 | 78.45209182 | 18.51000876 |
| DALYs | High-income North America | Female | 15-49 years | Polycystic ovarian syndrome | Rate | 2010 | 38.1525669 | 75.30756185 | 17.88766053 |
| DALYs | High-income North America | Female | 15-49 years | Polycystic ovarian syndrome | Rate | 2011 | 38.32484822 | 75.62713943 | 17.99381565 |
| DALYs | High-income North America | Female | 15-49 years | Polycystic ovarian syndrome | Rate | 2012 | 38.70799946 | 76.23742381 | 18.2020373 |
| DALYs | High-income North America | Female | 15-49 years | Polycystic ovarian syndrome | Rate | 2013 | 39.27595523 | 77.34370687 | 18.46634009 |
| DALYs | High-income North America | Female | 15-49 years | Polycystic ovarian syndrome | Rate | 2014 | 39.93625493 | 78.42422532 | 18.92031228 |
| DALYs | High-income North America | Female | 15-49 years | Polycystic ovarian syndrome | Rate | 2015 | 40.63930997 | 79.607927 | 19.35864131 |
| DALYs | High-income North America | Female | 15-49 years | Polycystic ovarian syndrome | Rate | 2016 | 42.25177308 | 82.34048296 | 20.0356985 |
| DALYs | High-income North America | Female | 15-49 years | Polycystic ovarian syndrome | Rate | 2017 | 45.25310217 | 87.94756819 | 21.38659143 |
| DALYs | High-income North America | Female | 15-49 years | Polycystic ovarian syndrome | Rate | 2018 | 48.97463169 | 95.61324188 | 23.27406727 |
| DALYs | High-income North America | Female | 15-49 years | Polycystic ovarian syndrome | Rate | 2019 | 52.83327379 | 103.6838832 | 24.94587221 |
| DALYs | High-income North America | Female | 15-49 years | Polycystic ovarian syndrome | Rate | 2020 | 57.19811229 | 112.6042657 | 27.40402411 |
| DALYs | High-income North America | Female | 15-49 years | Polycystic ovarian syndrome | Rate | 2021 | 63.90261007 | 129.2798406 | 29.11950598 |
| DALYs | North Africa and Middle East | Female | 15-49 years | Polycystic ovarian syndrome | Rate | 1990 | 27.01703088 | 56.99406896 | 11.80844995 |
| DALYs | North Africa and Middle East | Female | 15-49 years | Polycystic ovarian syndrome | Rate | 1991 | 27.27797565 | 57.70737475 | 11.96363991 |
| DALYs | North Africa and Middle East | Female | 15-49 years | Polycystic ovarian syndrome | Rate | 1992 | 27.52200343 | 58.3390899 | 12.07003077 |
| DALYs | North Africa and Middle East | Female | 15-49 years | Polycystic ovarian syndrome | Rate | 1993 | 27.74722613 | 58.92572012 | 12.15465098 |
| DALYs | North Africa and Middle East | Female | 15-49 years | Polycystic ovarian syndrome | Rate | 1994 | 27.96594982 | 59.38170979 | 12.30192889 |
| DALYs | North Africa and Middle East | Female | 15-49 years | Polycystic ovarian syndrome | Rate | 1995 | 28.19219284 | 60.04837971 | 12.44256051 |
| DALYs | North Africa and Middle East | Female | 15-49 years | Polycystic ovarian syndrome | Rate | 1996 | 28.44499622 | 60.6149906 | 12.51208622 |
| DALYs | North Africa and Middle East | Female | 15-49 years | Polycystic ovarian syndrome | Rate | 1997 | 28.7158288 | 61.14352098 | 12.57982071 |
| DALYs | North Africa and Middle East | Female | 15-49 years | Polycystic ovarian syndrome | Rate | 1998 | 29.03093743 | 61.38859526 | 12.7952014 |
| DALYs | North Africa and Middle East | Female | 15-49 years | Polycystic ovarian syndrome | Rate | 1999 | 29.37458337 | 61.47763368 | 12.84884699 |
| DALYs | North Africa and Middle East | Female | 15-49 years | Polycystic ovarian syndrome | Rate | 2000 | 29.73598754 | 62.60539809 | 12.9512707 |
| DALYs | North Africa and Middle East | Female | 15-49 years | Polycystic ovarian syndrome | Rate | 2001 | 30.22742373 | 63.95442949 | 13.23459911 |
| DALYs | North Africa and Middle East | Female | 15-49 years | Polycystic ovarian syndrome | Rate | 2002 | 30.87230514 | 65.64055686 | 13.51304586 |
| DALYs | North Africa and Middle East | Female | 15-49 years | Polycystic ovarian syndrome | Rate | 2003 | 31.55179488 | 67.03241735 | 13.81011305 |
| DALYs | North Africa and Middle East | Female | 15-49 years | Polycystic ovarian syndrome | Rate | 2004 | 32.16344122 | 68.37843087 | 14.01783775 |
| DALYs | North Africa and Middle East | Female | 15-49 years | Polycystic ovarian syndrome | Rate | 2005 | 32.60973986 | 69.43005076 | 14.30440416 |
| DALYs | North Africa and Middle East | Female | 15-49 years | Polycystic ovarian syndrome | Rate | 2006 | 32.90157637 | 70.27107972 | 14.45747965 |
| DALYs | North Africa and Middle East | Female | 15-49 years | Polycystic ovarian syndrome | Rate | 2007 | 33.21496119 | 70.95960588 | 14.56265418 |
| DALYs | North Africa and Middle East | Female | 15-49 years | Polycystic ovarian syndrome | Rate | 2008 | 33.53105749 | 71.54753979 | 14.7790221 |
| DALYs | North Africa and Middle East | Female | 15-49 years | Polycystic ovarian syndrome | Rate | 2009 | 33.78606075 | 72.02368033 | 14.82712363 |
| DALYs | North Africa and Middle East | Female | 15-49 years | Polycystic ovarian syndrome | Rate | 2010 | 34.04739018 | 72.66339083 | 14.92539092 |
| DALYs | North Africa and Middle East | Female | 15-49 years | Polycystic ovarian syndrome | Rate | 2011 | 34.31251808 | 73.22989937 | 15.07678805 |
| DALYs | North Africa and Middle East | Female | 15-49 years | Polycystic ovarian syndrome | Rate | 2012 | 34.64675164 | 74.00175569 | 15.18551432 |
| DALYs | North Africa and Middle East | Female | 15-49 years | Polycystic ovarian syndrome | Rate | 2013 | 34.9872914 | 74.49158344 | 15.33583042 |
| DALYs | North Africa and Middle East | Female | 15-49 years | Polycystic ovarian syndrome | Rate | 2014 | 35.29739407 | 75.55656352 | 15.47439043 |
| DALYs | North Africa and Middle East | Female | 15-49 years | Polycystic ovarian syndrome | Rate | 2015 | 35.54417095 | 75.90773572 | 15.5829138 |
| DALYs | North Africa and Middle East | Female | 15-49 years | Polycystic ovarian syndrome | Rate | 2016 | 35.56455186 | 75.64671047 | 15.6890083 |
| DALYs | North Africa and Middle East | Female | 15-49 years | Polycystic ovarian syndrome | Rate | 2017 | 35.36905191 | 75.21256767 | 15.53875989 |
| DALYs | North Africa and Middle East | Female | 15-49 years | Polycystic ovarian syndrome | Rate | 2018 | 35.2279525 | 75.08642802 | 15.58370611 |
| DALYs | North Africa and Middle East | Female | 15-49 years | Polycystic ovarian syndrome | Rate | 2019 | 35.29502591 | 74.81573639 | 15.64388892 |
| DALYs | North Africa and Middle East | Female | 15-49 years | Polycystic ovarian syndrome | Rate | 2020 | 35.87825215 | 75.52899963 | 16.00121889 |
| DALYs | North Africa and Middle East | Female | 15-49 years | Polycystic ovarian syndrome | Rate | 2021 | 35.28142407 | 74.74153656 | 15.75831456 |
| DALYs | Oceania | Female | 15-49 years | Polycystic ovarian syndrome | Rate | 1990 | 21.41588518 | 44.9875655 | 9.721678414 |
| DALYs | Oceania | Female | 15-49 years | Polycystic ovarian syndrome | Rate | 1991 | 22.00873549 | 46.60971731 | 9.795342049 |
| DALYs | Oceania | Female | 15-49 years | Polycystic ovarian syndrome | Rate | 1992 | 22.61211023 | 46.92099752 | 9.925420308 |
| DALYs | Oceania | Female | 15-49 years | Polycystic ovarian syndrome | Rate | 1993 | 23.17429581 | 48.99247515 | 10.19081795 |
| DALYs | Oceania | Female | 15-49 years | Polycystic ovarian syndrome | Rate | 1994 | 23.74387688 | 49.81372873 | 10.36292517 |
| DALYs | Oceania | Female | 15-49 years | Polycystic ovarian syndrome | Rate | 1995 | 24.32930894 | 51.84522391 | 10.67656552 |
| DALYs | Oceania | Female | 15-49 years | Polycystic ovarian syndrome | Rate | 1996 | 24.92790127 | 52.72851527 | 10.98138722 |
| DALYs | Oceania | Female | 15-49 years | Polycystic ovarian syndrome | Rate | 1997 | 25.54717541 | 54.72993265 | 11.29058201 |
| DALYs | Oceania | Female | 15-49 years | Polycystic ovarian syndrome | Rate | 1998 | 26.12649935 | 55.66329387 | 11.56437542 |
| DALYs | Oceania | Female | 15-49 years | Polycystic ovarian syndrome | Rate | 1999 | 26.65961839 | 56.20941329 | 11.85981331 |
| DALYs | Oceania | Female | 15-49 years | Polycystic ovarian syndrome | Rate | 2000 | 27.06718027 | 57.10840225 | 11.96316101 |
| DALYs | Oceania | Female | 15-49 years | Polycystic ovarian syndrome | Rate | 2001 | 27.39020845 | 58.07857965 | 12.21663847 |
| DALYs | Oceania | Female | 15-49 years | Polycystic ovarian syndrome | Rate | 2002 | 27.72532552 | 58.32785714 | 12.33614047 |
| DALYs | Oceania | Female | 15-49 years | Polycystic ovarian syndrome | Rate | 2003 | 28.00453408 | 59.48875783 | 12.4311099 |
| DALYs | Oceania | Female | 15-49 years | Polycystic ovarian syndrome | Rate | 2004 | 28.18026802 | 60.08406654 | 12.47295341 |
| DALYs | Oceania | Female | 15-49 years | Polycystic ovarian syndrome | Rate | 2005 | 28.24163043 | 60.05077733 | 12.56303726 |
| DALYs | Oceania | Female | 15-49 years | Polycystic ovarian syndrome | Rate | 2006 | 28.21430015 | 59.724466 | 12.58390403 |
| DALYs | Oceania | Female | 15-49 years | Polycystic ovarian syndrome | Rate | 2007 | 28.1052311 | 58.90224299 | 12.4269763 |
| DALYs | Oceania | Female | 15-49 years | Polycystic ovarian syndrome | Rate | 2008 | 27.96507148 | 58.66386972 | 12.40651718 |
| DALYs | Oceania | Female | 15-49 years | Polycystic ovarian syndrome | Rate | 2009 | 27.87930487 | 58.94537763 | 12.42839621 |
| DALYs | Oceania | Female | 15-49 years | Polycystic ovarian syndrome | Rate | 2010 | 27.7789204 | 58.57290618 | 12.38389473 |
| DALYs | Oceania | Female | 15-49 years | Polycystic ovarian syndrome | Rate | 2011 | 27.7911175 | 58.28506183 | 12.44574932 |
| DALYs | Oceania | Female | 15-49 years | Polycystic ovarian syndrome | Rate | 2012 | 27.84720166 | 58.77145194 | 12.45437873 |
| DALYs | Oceania | Female | 15-49 years | Polycystic ovarian syndrome | Rate | 2013 | 27.96868098 | 58.79840139 | 12.479352 |
| DALYs | Oceania | Female | 15-49 years | Polycystic ovarian syndrome | Rate | 2014 | 28.10802637 | 58.97993892 | 12.56402756 |
| DALYs | Oceania | Female | 15-49 years | Polycystic ovarian syndrome | Rate | 2015 | 28.23280142 | 59.50297075 | 12.5708781 |
| DALYs | Oceania | Female | 15-49 years | Polycystic ovarian syndrome | Rate | 2016 | 28.43413437 | 59.81476226 | 12.57914082 |
| DALYs | Oceania | Female | 15-49 years | Polycystic ovarian syndrome | Rate | 2017 | 28.65114295 | 60.58960165 | 12.75896603 |
| DALYs | Oceania | Female | 15-49 years | Polycystic ovarian syndrome | Rate | 2018 | 28.89244679 | 61.21027977 | 12.88310983 |
| DALYs | Oceania | Female | 15-49 years | Polycystic ovarian syndrome | Rate | 2019 | 29.01146973 | 60.62221732 | 12.76272797 |
| DALYs | Oceania | Female | 15-49 years | Polycystic ovarian syndrome | Rate | 2020 | 29.0145342 | 60.81291316 | 12.53092806 |
| DALYs | Oceania | Female | 15-49 years | Polycystic ovarian syndrome | Rate | 2021 | 29.5535516 | 61.92117461 | 12.80201276 |
| DALYs | South Asia | Female | 15-49 years | Polycystic ovarian syndrome | Rate | 1990 | 10.89874799 | 23.06359932 | 4.743965118 |
| DALYs | South Asia | Female | 15-49 years | Polycystic ovarian syndrome | Rate | 1991 | 11.02197776 | 23.26767914 | 4.784862815 |
| DALYs | South Asia | Female | 15-49 years | Polycystic ovarian syndrome | Rate | 1992 | 11.15579497 | 23.65365244 | 4.849334007 |
| DALYs | South Asia | Female | 15-49 years | Polycystic ovarian syndrome | Rate | 1993 | 11.28997165 | 24.04382239 | 4.935807919 |
| DALYs | South Asia | Female | 15-49 years | Polycystic ovarian syndrome | Rate | 1994 | 11.43444385 | 24.42906218 | 4.946355434 |
| DALYs | South Asia | Female | 15-49 years | Polycystic ovarian syndrome | Rate | 1995 | 11.56942752 | 24.67132361 | 5.03369855 |
| DALYs | South Asia | Female | 15-49 years | Polycystic ovarian syndrome | Rate | 1996 | 11.71136666 | 25.0572083 | 5.097497358 |
| DALYs | South Asia | Female | 15-49 years | Polycystic ovarian syndrome | Rate | 1997 | 11.86071519 | 25.41154025 | 5.141529481 |
| DALYs | South Asia | Female | 15-49 years | Polycystic ovarian syndrome | Rate | 1998 | 12.02610017 | 25.69074726 | 5.189907852 |
| DALYs | South Asia | Female | 15-49 years | Polycystic ovarian syndrome | Rate | 1999 | 12.19411922 | 26.02202978 | 5.283242059 |
| DALYs | South Asia | Female | 15-49 years | Polycystic ovarian syndrome | Rate | 2000 | 12.36072932 | 26.40171494 | 5.376179204 |
| DALYs | South Asia | Female | 15-49 years | Polycystic ovarian syndrome | Rate | 2001 | 12.55410753 | 26.74944999 | 5.43242379 |
| DALYs | South Asia | Female | 15-49 years | Polycystic ovarian syndrome | Rate | 2002 | 12.77100567 | 27.03852185 | 5.512134414 |
| DALYs | South Asia | Female | 15-49 years | Polycystic ovarian syndrome | Rate | 2003 | 13.01351835 | 27.77007382 | 5.607411991 |
| DALYs | South Asia | Female | 15-49 years | Polycystic ovarian syndrome | Rate | 2004 | 13.28001443 | 28.32312409 | 5.744952812 |
| DALYs | South Asia | Female | 15-49 years | Polycystic ovarian syndrome | Rate | 2005 | 13.55869335 | 28.80786024 | 5.848819268 |
| DALYs | South Asia | Female | 15-49 years | Polycystic ovarian syndrome | Rate | 2006 | 13.96008155 | 29.68764819 | 5.987440946 |
| DALYs | South Asia | Female | 15-49 years | Polycystic ovarian syndrome | Rate | 2007 | 14.5661306 | 30.99651129 | 6.250868948 |
| DALYs | South Asia | Female | 15-49 years | Polycystic ovarian syndrome | Rate | 2008 | 15.23259411 | 32.37609939 | 6.516158153 |
| DALYs | South Asia | Female | 15-49 years | Polycystic ovarian syndrome | Rate | 2009 | 15.86922219 | 33.73886027 | 6.816166433 |
| DALYs | South Asia | Female | 15-49 years | Polycystic ovarian syndrome | Rate | 2010 | 16.35721117 | 34.59537223 | 7.023536641 |
| DALYs | South Asia | Female | 15-49 years | Polycystic ovarian syndrome | Rate | 2011 | 16.69963234 | 35.3489409 | 7.195265756 |
| DALYs | South Asia | Female | 15-49 years | Polycystic ovarian syndrome | Rate | 2012 | 16.99097158 | 35.92460174 | 7.30115242 |
| DALYs | South Asia | Female | 15-49 years | Polycystic ovarian syndrome | Rate | 2013 | 17.24874243 | 36.33511867 | 7.47165944 |
| DALYs | South Asia | Female | 15-49 years | Polycystic ovarian syndrome | Rate | 2014 | 17.49070791 | 36.80699424 | 7.586509542 |
| DALYs | South Asia | Female | 15-49 years | Polycystic ovarian syndrome | Rate | 2015 | 17.7248359 | 36.99330806 | 7.720114172 |
| DALYs | South Asia | Female | 15-49 years | Polycystic ovarian syndrome | Rate | 2016 | 18.00847679 | 37.69714087 | 7.831623512 |
| DALYs | South Asia | Female | 15-49 years | Polycystic ovarian syndrome | Rate | 2017 | 18.31217759 | 38.52903093 | 7.955894501 |
| DALYs | South Asia | Female | 15-49 years | Polycystic ovarian syndrome | Rate | 2018 | 18.60663193 | 39.15484397 | 8.053470585 |
| DALYs | South Asia | Female | 15-49 years | Polycystic ovarian syndrome | Rate | 2019 | 18.87212607 | 39.8564791 | 8.150482307 |
| DALYs | South Asia | Female | 15-49 years | Polycystic ovarian syndrome | Rate | 2020 | 19.12805517 | 40.3974194 | 8.245274845 |
| DALYs | South Asia | Female | 15-49 years | Polycystic ovarian syndrome | Rate | 2021 | 19.08936714 | 39.99407259 | 8.271964888 |
| DALYs | Southeast Asia | Female | 15-49 years | Polycystic ovarian syndrome | Rate | 1990 | 26.07297189 | 52.98070235 | 11.38780415 |
| DALYs | Southeast Asia | Female | 15-49 years | Polycystic ovarian syndrome | Rate | 1991 | 26.35614454 | 53.99736061 | 11.46152316 |
| DALYs | Southeast Asia | Female | 15-49 years | Polycystic ovarian syndrome | Rate | 1992 | 26.69167487 | 54.96364984 | 11.65774011 |
| DALYs | Southeast Asia | Female | 15-49 years | Polycystic ovarian syndrome | Rate | 1993 | 27.09816089 | 55.93594013 | 11.77268539 |
| DALYs | Southeast Asia | Female | 15-49 years | Polycystic ovarian syndrome | Rate | 1994 | 27.54408764 | 57.17070959 | 11.98442454 |
| DALYs | Southeast Asia | Female | 15-49 years | Polycystic ovarian syndrome | Rate | 1995 | 28.03949186 | 58.16293899 | 12.21507659 |
| DALYs | Southeast Asia | Female | 15-49 years | Polycystic ovarian syndrome | Rate | 1996 | 28.62125505 | 59.26839796 | 12.49008641 |
| DALYs | Southeast Asia | Female | 15-49 years | Polycystic ovarian syndrome | Rate | 1997 | 29.33360455 | 60.68445753 | 13.01566046 |
| DALYs | Southeast Asia | Female | 15-49 years | Polycystic ovarian syndrome | Rate | 1998 | 30.14780862 | 62.55954203 | 13.44954151 |
| DALYs | Southeast Asia | Female | 15-49 years | Polycystic ovarian syndrome | Rate | 1999 | 30.99707993 | 64.04566514 | 13.92347357 |
| DALYs | Southeast Asia | Female | 15-49 years | Polycystic ovarian syndrome | Rate | 2000 | 31.80088286 | 65.83360959 | 14.34618936 |
| DALYs | Southeast Asia | Female | 15-49 years | Polycystic ovarian syndrome | Rate | 2001 | 32.6742123 | 67.31708598 | 14.70597046 |
| DALYs | Southeast Asia | Female | 15-49 years | Polycystic ovarian syndrome | Rate | 2002 | 33.69325277 | 68.81854726 | 15.12148368 |
| DALYs | Southeast Asia | Female | 15-49 years | Polycystic ovarian syndrome | Rate | 2003 | 34.75968672 | 70.94476896 | 15.62162229 |
| DALYs | Southeast Asia | Female | 15-49 years | Polycystic ovarian syndrome | Rate | 2004 | 35.79447618 | 73.23371537 | 15.96579866 |
| DALYs | Southeast Asia | Female | 15-49 years | Polycystic ovarian syndrome | Rate | 2005 | 36.75828286 | 75.02819322 | 16.28088675 |
| DALYs | Southeast Asia | Female | 15-49 years | Polycystic ovarian syndrome | Rate | 2006 | 37.65633695 | 77.17212515 | 16.69087388 |
| DALYs | Southeast Asia | Female | 15-49 years | Polycystic ovarian syndrome | Rate | 2007 | 38.58492186 | 79.25612216 | 17.08448786 |
| DALYs | Southeast Asia | Female | 15-49 years | Polycystic ovarian syndrome | Rate | 2008 | 39.53579651 | 81.84065302 | 17.4960989 |
| DALYs | Southeast Asia | Female | 15-49 years | Polycystic ovarian syndrome | Rate | 2009 | 40.46406155 | 83.99220219 | 17.78726678 |
| DALYs | Southeast Asia | Female | 15-49 years | Polycystic ovarian syndrome | Rate | 2010 | 41.34535479 | 85.91625995 | 18.16742862 |
| DALYs | Southeast Asia | Female | 15-49 years | Polycystic ovarian syndrome | Rate | 2011 | 42.20067278 | 87.90905798 | 18.54603246 |
| DALYs | Southeast Asia | Female | 15-49 years | Polycystic ovarian syndrome | Rate | 2012 | 43.08371805 | 89.513139 | 18.95106828 |
| DALYs | Southeast Asia | Female | 15-49 years | Polycystic ovarian syndrome | Rate | 2013 | 43.97065715 | 91.24044576 | 19.36507506 |
| DALYs | Southeast Asia | Female | 15-49 years | Polycystic ovarian syndrome | Rate | 2014 | 44.77827216 | 93.04441652 | 19.70375176 |
| DALYs | Southeast Asia | Female | 15-49 years | Polycystic ovarian syndrome | Rate | 2015 | 45.4290167 | 94.35325277 | 20.00298075 |
| DALYs | Southeast Asia | Female | 15-49 years | Polycystic ovarian syndrome | Rate | 2016 | 45.98002759 | 95.05944883 | 20.208665 |
| DALYs | Southeast Asia | Female | 15-49 years | Polycystic ovarian syndrome | Rate | 2017 | 46.52832885 | 95.60023152 | 20.42207401 |
| DALYs | Southeast Asia | Female | 15-49 years | Polycystic ovarian syndrome | Rate | 2018 | 47.07533183 | 96.57178459 | 20.70010539 |
| DALYs | Southeast Asia | Female | 15-49 years | Polycystic ovarian syndrome | Rate | 2019 | 47.56180541 | 97.49921724 | 20.83470553 |
| DALYs | Southeast Asia | Female | 15-49 years | Polycystic ovarian syndrome | Rate | 2020 | 48.1578922 | 98.98886739 | 21.14035579 |
| DALYs | Southeast Asia | Female | 15-49 years | Polycystic ovarian syndrome | Rate | 2021 | 48.10286822 | 99.17192577 | 21.31546293 |
| DALYs | Southern Latin America | Female | 15-49 years | Polycystic ovarian syndrome | Rate | 1990 | 20.22109746 | 41.50390557 | 9.053866734 |
| DALYs | Southern Latin America | Female | 15-49 years | Polycystic ovarian syndrome | Rate | 1991 | 20.78827766 | 42.81772973 | 9.191497426 |
| DALYs | Southern Latin America | Female | 15-49 years | Polycystic ovarian syndrome | Rate | 1992 | 21.40241783 | 43.45896746 | 9.420144989 |
| DALYs | Southern Latin America | Female | 15-49 years | Polycystic ovarian syndrome | Rate | 1993 | 22.03266092 | 45.36001964 | 9.712711285 |
| DALYs | Southern Latin America | Female | 15-49 years | Polycystic ovarian syndrome | Rate | 1994 | 22.66850669 | 46.89621609 | 9.986233248 |
| DALYs | Southern Latin America | Female | 15-49 years | Polycystic ovarian syndrome | Rate | 1995 | 23.35812754 | 48.36260705 | 10.14431632 |
| DALYs | Southern Latin America | Female | 15-49 years | Polycystic ovarian syndrome | Rate | 1996 | 24.12765097 | 49.87351502 | 10.42638378 |
| DALYs | Southern Latin America | Female | 15-49 years | Polycystic ovarian syndrome | Rate | 1997 | 24.93448627 | 51.84874177 | 10.84640579 |
| DALYs | Southern Latin America | Female | 15-49 years | Polycystic ovarian syndrome | Rate | 1998 | 25.79517734 | 52.87002965 | 11.18143094 |
| DALYs | Southern Latin America | Female | 15-49 years | Polycystic ovarian syndrome | Rate | 1999 | 26.5865134 | 54.94493675 | 11.61121724 |
| DALYs | Southern Latin America | Female | 15-49 years | Polycystic ovarian syndrome | Rate | 2000 | 27.29999998 | 56.97164263 | 11.86579895 |
| DALYs | Southern Latin America | Female | 15-49 years | Polycystic ovarian syndrome | Rate | 2001 | 27.88660738 | 57.68169726 | 12.02066691 |
| DALYs | Southern Latin America | Female | 15-49 years | Polycystic ovarian syndrome | Rate | 2002 | 28.46844 | 58.9883333 | 12.43085611 |
| DALYs | Southern Latin America | Female | 15-49 years | Polycystic ovarian syndrome | Rate | 2003 | 28.98878217 | 59.54050129 | 12.69512422 |
| DALYs | Southern Latin America | Female | 15-49 years | Polycystic ovarian syndrome | Rate | 2004 | 29.41196963 | 60.56485392 | 12.94906259 |
| DALYs | Southern Latin America | Female | 15-49 years | Polycystic ovarian syndrome | Rate | 2005 | 29.77615134 | 60.46918583 | 13.03337828 |
| DALYs | Southern Latin America | Female | 15-49 years | Polycystic ovarian syndrome | Rate | 2006 | 30.05363483 | 61.39933858 | 13.19109186 |
| DALYs | Southern Latin America | Female | 15-49 years | Polycystic ovarian syndrome | Rate | 2007 | 30.33706635 | 62.64862485 | 13.43278082 |
| DALYs | Southern Latin America | Female | 15-49 years | Polycystic ovarian syndrome | Rate | 2008 | 30.63002203 | 63.79983574 | 13.38218579 |
| DALYs | Southern Latin America | Female | 15-49 years | Polycystic ovarian syndrome | Rate | 2009 | 30.89430344 | 64.44123839 | 13.6025839 |
| DALYs | Southern Latin America | Female | 15-49 years | Polycystic ovarian syndrome | Rate | 2010 | 31.06374989 | 64.36921111 | 13.73116543 |
| DALYs | Southern Latin America | Female | 15-49 years | Polycystic ovarian syndrome | Rate | 2011 | 31.20543243 | 63.77597767 | 13.70171204 |
| DALYs | Southern Latin America | Female | 15-49 years | Polycystic ovarian syndrome | Rate | 2012 | 31.33822267 | 64.27199259 | 13.58208646 |
| DALYs | Southern Latin America | Female | 15-49 years | Polycystic ovarian syndrome | Rate | 2013 | 31.46579971 | 64.59721664 | 13.72997201 |
| DALYs | Southern Latin America | Female | 15-49 years | Polycystic ovarian syndrome | Rate | 2014 | 31.53048933 | 65.16818658 | 13.67714035 |
| DALYs | Southern Latin America | Female | 15-49 years | Polycystic ovarian syndrome | Rate | 2015 | 31.61764202 | 64.97796967 | 13.6867117 |
| DALYs | Southern Latin America | Female | 15-49 years | Polycystic ovarian syndrome | Rate | 2016 | 31.74786714 | 65.26075135 | 13.64117662 |
| DALYs | Southern Latin America | Female | 15-49 years | Polycystic ovarian syndrome | Rate | 2017 | 31.86915789 | 65.35159787 | 13.82138937 |
| DALYs | Southern Latin America | Female | 15-49 years | Polycystic ovarian syndrome | Rate | 2018 | 32.05720859 | 65.66737651 | 14.03164831 |
| DALYs | Southern Latin America | Female | 15-49 years | Polycystic ovarian syndrome | Rate | 2019 | 32.2623083 | 66.6857325 | 14.04430361 |
| DALYs | Southern Latin America | Female | 15-49 years | Polycystic ovarian syndrome | Rate | 2020 | 32.55245123 | 65.99419393 | 14.15268015 |
| DALYs | Southern Latin America | Female | 15-49 years | Polycystic ovarian syndrome | Rate | 2021 | 32.34264539 | 67.17562582 | 14.05237045 |
| DALYs | Southern Sub-Saharan Africa | Female | 15-49 years | Polycystic ovarian syndrome | Rate | 1990 | 14.59181849 | 31.87798196 | 6.27628488 |
| DALYs | Southern Sub-Saharan Africa | Female | 15-49 years | Polycystic ovarian syndrome | Rate | 1991 | 14.63623096 | 31.52178797 | 6.310728282 |
| DALYs | Southern Sub-Saharan Africa | Female | 15-49 years | Polycystic ovarian syndrome | Rate | 1992 | 14.7118057 | 31.96632389 | 6.403155343 |
| DALYs | Southern Sub-Saharan Africa | Female | 15-49 years | Polycystic ovarian syndrome | Rate | 1993 | 14.82194373 | 31.74899601 | 6.494529026 |
| DALYs | Southern Sub-Saharan Africa | Female | 15-49 years | Polycystic ovarian syndrome | Rate | 1994 | 14.95918194 | 32.16044326 | 6.461366499 |
| DALYs | Southern Sub-Saharan Africa | Female | 15-49 years | Polycystic ovarian syndrome | Rate | 1995 | 15.12171601 | 32.83857538 | 6.60261425 |
| DALYs | Southern Sub-Saharan Africa | Female | 15-49 years | Polycystic ovarian syndrome | Rate | 1996 | 15.362948 | 32.98561316 | 6.732209819 |
| DALYs | Southern Sub-Saharan Africa | Female | 15-49 years | Polycystic ovarian syndrome | Rate | 1997 | 15.67691814 | 33.54662311 | 6.867835092 |
| DALYs | Southern Sub-Saharan Africa | Female | 15-49 years | Polycystic ovarian syndrome | Rate | 1998 | 16.00824385 | 34.13987111 | 6.995330273 |
| DALYs | Southern Sub-Saharan Africa | Female | 15-49 years | Polycystic ovarian syndrome | Rate | 1999 | 16.28910896 | 34.81442127 | 7.102431322 |
| DALYs | Southern Sub-Saharan Africa | Female | 15-49 years | Polycystic ovarian syndrome | Rate | 2000 | 16.4746326 | 35.15949985 | 7.187193734 |
| DALYs | Southern Sub-Saharan Africa | Female | 15-49 years | Polycystic ovarian syndrome | Rate | 2001 | 16.57996602 | 35.40789403 | 7.295195249 |
| DALYs | Southern Sub-Saharan Africa | Female | 15-49 years | Polycystic ovarian syndrome | Rate | 2002 | 16.67847456 | 35.81747221 | 7.263000006 |
| DALYs | Southern Sub-Saharan Africa | Female | 15-49 years | Polycystic ovarian syndrome | Rate | 2003 | 16.78319306 | 35.79377943 | 7.273636882 |
| DALYs | Southern Sub-Saharan Africa | Female | 15-49 years | Polycystic ovarian syndrome | Rate | 2004 | 16.87561881 | 36.25384656 | 7.330803479 |
| DALYs | Southern Sub-Saharan Africa | Female | 15-49 years | Polycystic ovarian syndrome | Rate | 2005 | 16.97130992 | 36.43773652 | 7.365134992 |
| DALYs | Southern Sub-Saharan Africa | Female | 15-49 years | Polycystic ovarian syndrome | Rate | 2006 | 17.09393161 | 36.99745493 | 7.391509176 |
| DALYs | Southern Sub-Saharan Africa | Female | 15-49 years | Polycystic ovarian syndrome | Rate | 2007 | 17.25086442 | 36.78407868 | 7.455914591 |
| DALYs | Southern Sub-Saharan Africa | Female | 15-49 years | Polycystic ovarian syndrome | Rate | 2008 | 17.41117249 | 37.2198367 | 7.544305268 |
| DALYs | Southern Sub-Saharan Africa | Female | 15-49 years | Polycystic ovarian syndrome | Rate | 2009 | 17.57500243 | 36.80756753 | 7.578031558 |
| DALYs | Southern Sub-Saharan Africa | Female | 15-49 years | Polycystic ovarian syndrome | Rate | 2010 | 17.68599467 | 37.48706729 | 7.679728646 |
| DALYs | Southern Sub-Saharan Africa | Female | 15-49 years | Polycystic ovarian syndrome | Rate | 2011 | 17.748187 | 37.49804415 | 7.675773298 |
| DALYs | Southern Sub-Saharan Africa | Female | 15-49 years | Polycystic ovarian syndrome | Rate | 2012 | 17.78226398 | 37.593417 | 7.750149556 |
| DALYs | Southern Sub-Saharan Africa | Female | 15-49 years | Polycystic ovarian syndrome | Rate | 2013 | 17.80099103 | 37.87011318 | 7.796492274 |
| DALYs | Southern Sub-Saharan Africa | Female | 15-49 years | Polycystic ovarian syndrome | Rate | 2014 | 17.81214185 | 37.60014175 | 7.708103133 |
| DALYs | Southern Sub-Saharan Africa | Female | 15-49 years | Polycystic ovarian syndrome | Rate | 2015 | 17.85555426 | 37.72230942 | 7.712918017 |
| DALYs | Southern Sub-Saharan Africa | Female | 15-49 years | Polycystic ovarian syndrome | Rate | 2016 | 17.94044148 | 38.08853203 | 7.800179634 |
| DALYs | Southern Sub-Saharan Africa | Female | 15-49 years | Polycystic ovarian syndrome | Rate | 2017 | 18.05165319 | 38.26276619 | 7.839088726 |
| DALYs | Southern Sub-Saharan Africa | Female | 15-49 years | Polycystic ovarian syndrome | Rate | 2018 | 18.18858763 | 38.35819087 | 7.861141136 |
| DALYs | Southern Sub-Saharan Africa | Female | 15-49 years | Polycystic ovarian syndrome | Rate | 2019 | 18.29311746 | 38.5195021 | 7.826756582 |
| DALYs | Southern Sub-Saharan Africa | Female | 15-49 years | Polycystic ovarian syndrome | Rate | 2020 | 18.45154788 | 39.15968182 | 7.990559619 |
| DALYs | Southern Sub-Saharan Africa | Female | 15-49 years | Polycystic ovarian syndrome | Rate | 2021 | 18.22282416 | 38.40210784 | 7.804463584 |
| DALYs | Tropical Latin America | Female | 15-49 years | Polycystic ovarian syndrome | Rate | 1990 | 9.405326059 | 19.85201114 | 4.018869039 |
| DALYs | Tropical Latin America | Female | 15-49 years | Polycystic ovarian syndrome | Rate | 1991 | 9.722128612 | 20.43285849 | 4.123592852 |
| DALYs | Tropical Latin America | Female | 15-49 years | Polycystic ovarian syndrome | Rate | 1992 | 9.987115825 | 21.09226112 | 4.254969475 |
| DALYs | Tropical Latin America | Female | 15-49 years | Polycystic ovarian syndrome | Rate | 1993 | 10.19882404 | 21.5987546 | 4.340148726 |
| DALYs | Tropical Latin America | Female | 15-49 years | Polycystic ovarian syndrome | Rate | 1994 | 10.34099521 | 22.04372439 | 4.375043705 |
| DALYs | Tropical Latin America | Female | 15-49 years | Polycystic ovarian syndrome | Rate | 1995 | 10.39848856 | 22.29915459 | 4.436227035 |
| DALYs | Tropical Latin America | Female | 15-49 years | Polycystic ovarian syndrome | Rate | 1996 | 10.38540524 | 22.23125624 | 4.359330572 |
| DALYs | Tropical Latin America | Female | 15-49 years | Polycystic ovarian syndrome | Rate | 1997 | 10.29918867 | 21.73336048 | 4.344397903 |
| DALYs | Tropical Latin America | Female | 15-49 years | Polycystic ovarian syndrome | Rate | 1998 | 10.18810103 | 21.70176488 | 4.322994199 |
| DALYs | Tropical Latin America | Female | 15-49 years | Polycystic ovarian syndrome | Rate | 1999 | 10.08853634 | 21.36562416 | 4.230948165 |
| DALYs | Tropical Latin America | Female | 15-49 years | Polycystic ovarian syndrome | Rate | 2000 | 10.02020506 | 21.18922942 | 4.243985354 |
| DALYs | Tropical Latin America | Female | 15-49 years | Polycystic ovarian syndrome | Rate | 2001 | 9.9798965 | 21.20439402 | 4.26387393 |
| DALYs | Tropical Latin America | Female | 15-49 years | Polycystic ovarian syndrome | Rate | 2002 | 9.936652315 | 20.90773716 | 4.242974426 |
| DALYs | Tropical Latin America | Female | 15-49 years | Polycystic ovarian syndrome | Rate | 2003 | 9.892212339 | 20.98340519 | 4.230683922 |
| DALYs | Tropical Latin America | Female | 15-49 years | Polycystic ovarian syndrome | Rate | 2004 | 9.84311208 | 20.79593076 | 4.24450815 |
| DALYs | Tropical Latin America | Female | 15-49 years | Polycystic ovarian syndrome | Rate | 2005 | 9.787549753 | 20.73966951 | 4.244777325 |
| DALYs | Tropical Latin America | Female | 15-49 years | Polycystic ovarian syndrome | Rate | 2006 | 9.64659419 | 20.27178128 | 4.15877128 |
| DALYs | Tropical Latin America | Female | 15-49 years | Polycystic ovarian syndrome | Rate | 2007 | 9.40620107 | 19.81207591 | 4.078495523 |
| DALYs | Tropical Latin America | Female | 15-49 years | Polycystic ovarian syndrome | Rate | 2008 | 9.143763083 | 18.99679948 | 3.959023833 |
| DALYs | Tropical Latin America | Female | 15-49 years | Polycystic ovarian syndrome | Rate | 2009 | 8.941769874 | 18.94825178 | 3.869162206 |
| DALYs | Tropical Latin America | Female | 15-49 years | Polycystic ovarian syndrome | Rate | 2010 | 8.87227421 | 18.54717412 | 3.880261336 |
| DALYs | Tropical Latin America | Female | 15-49 years | Polycystic ovarian syndrome | Rate | 2011 | 8.931629259 | 18.64308735 | 3.876108971 |
| DALYs | Tropical Latin America | Female | 15-49 years | Polycystic ovarian syndrome | Rate | 2012 | 9.024875478 | 18.89790832 | 3.909511809 |
| DALYs | Tropical Latin America | Female | 15-49 years | Polycystic ovarian syndrome | Rate | 2013 | 9.147343097 | 19.11777872 | 3.946276178 |
| DALYs | Tropical Latin America | Female | 15-49 years | Polycystic ovarian syndrome | Rate | 2014 | 9.278686496 | 19.26358738 | 4.024827831 |
| DALYs | Tropical Latin America | Female | 15-49 years | Polycystic ovarian syndrome | Rate | 2015 | 9.402620573 | 19.72746771 | 4.054313305 |
| DALYs | Tropical Latin America | Female | 15-49 years | Polycystic ovarian syndrome | Rate | 2016 | 9.557891033 | 20.12006158 | 4.079402225 |
| DALYs | Tropical Latin America | Female | 15-49 years | Polycystic ovarian syndrome | Rate | 2017 | 9.753373116 | 20.57920391 | 4.156898913 |
| DALYs | Tropical Latin America | Female | 15-49 years | Polycystic ovarian syndrome | Rate | 2018 | 9.936899911 | 20.9919406 | 4.221224333 |
| DALYs | Tropical Latin America | Female | 15-49 years | Polycystic ovarian syndrome | Rate | 2019 | 10.05729451 | 21.37470987 | 4.273946681 |
| DALYs | Tropical Latin America | Female | 15-49 years | Polycystic ovarian syndrome | Rate | 2020 | 10.08480468 | 21.12528641 | 4.358749296 |
| DALYs | Tropical Latin America | Female | 15-49 years | Polycystic ovarian syndrome | Rate | 2021 | 10.08747587 | 21.28580529 | 4.301700542 |
| DALYs | Western Europe | Female | 15-49 years | Polycystic ovarian syndrome | Rate | 1990 | 60.40588368 | 125.9896168 | 27.46697707 |
| DALYs | Western Europe | Female | 15-49 years | Polycystic ovarian syndrome | Rate | 1991 | 61.22244726 | 128.1429968 | 27.89185036 |
| DALYs | Western Europe | Female | 15-49 years | Polycystic ovarian syndrome | Rate | 1992 | 61.90402047 | 129.5232928 | 28.226819 |
| DALYs | Western Europe | Female | 15-49 years | Polycystic ovarian syndrome | Rate | 1993 | 62.55252483 | 130.1819431 | 28.50017822 |
| DALYs | Western Europe | Female | 15-49 years | Polycystic ovarian syndrome | Rate | 1994 | 63.09495366 | 130.4503372 | 28.80448374 |
| DALYs | Western Europe | Female | 15-49 years | Polycystic ovarian syndrome | Rate | 1995 | 63.54429867 | 130.5475136 | 29.03079366 |
| DALYs | Western Europe | Female | 15-49 years | Polycystic ovarian syndrome | Rate | 1996 | 64.02851737 | 131.8504474 | 29.13873521 |
| DALYs | Western Europe | Female | 15-49 years | Polycystic ovarian syndrome | Rate | 1997 | 64.5857466 | 133.1892683 | 29.46253782 |
| DALYs | Western Europe | Female | 15-49 years | Polycystic ovarian syndrome | Rate | 1998 | 65.12365623 | 134.7986101 | 29.66860344 |
| DALYs | Western Europe | Female | 15-49 years | Polycystic ovarian syndrome | Rate | 1999 | 65.57728759 | 135.7116664 | 29.85917899 |
| DALYs | Western Europe | Female | 15-49 years | Polycystic ovarian syndrome | Rate | 2000 | 65.89630643 | 137.0230173 | 30.02008587 |
| DALYs | Western Europe | Female | 15-49 years | Polycystic ovarian syndrome | Rate | 2001 | 65.99861087 | 137.2402123 | 30.06645716 |
| DALYs | Western Europe | Female | 15-49 years | Polycystic ovarian syndrome | Rate | 2002 | 65.96162504 | 137.1927018 | 30.18859848 |
| DALYs | Western Europe | Female | 15-49 years | Polycystic ovarian syndrome | Rate | 2003 | 65.86350653 | 137.5640449 | 30.06172365 |
| DALYs | Western Europe | Female | 15-49 years | Polycystic ovarian syndrome | Rate | 2004 | 65.7050559 | 137.2552059 | 29.89314777 |
| DALYs | Western Europe | Female | 15-49 years | Polycystic ovarian syndrome | Rate | 2005 | 65.48961948 | 137.2621713 | 29.63074726 |
| DALYs | Western Europe | Female | 15-49 years | Polycystic ovarian syndrome | Rate | 2006 | 65.24574116 | 136.7848781 | 29.5617339 |
| DALYs | Western Europe | Female | 15-49 years | Polycystic ovarian syndrome | Rate | 2007 | 64.9542089 | 136.3115635 | 29.4759255 |
| DALYs | Western Europe | Female | 15-49 years | Polycystic ovarian syndrome | Rate | 2008 | 64.7198366 | 135.4886597 | 29.26112734 |
| DALYs | Western Europe | Female | 15-49 years | Polycystic ovarian syndrome | Rate | 2009 | 64.58470884 | 135.6144845 | 29.36905324 |
| DALYs | Western Europe | Female | 15-49 years | Polycystic ovarian syndrome | Rate | 2010 | 64.55617065 | 135.0812882 | 29.45831661 |
| DALYs | Western Europe | Female | 15-49 years | Polycystic ovarian syndrome | Rate | 2011 | 64.67325091 | 135.4758839 | 29.51422725 |
| DALYs | Western Europe | Female | 15-49 years | Polycystic ovarian syndrome | Rate | 2012 | 64.8677675 | 135.3815526 | 29.69787224 |
| DALYs | Western Europe | Female | 15-49 years | Polycystic ovarian syndrome | Rate | 2013 | 65.0684813 | 136.1769211 | 29.8345946 |
| DALYs | Western Europe | Female | 15-49 years | Polycystic ovarian syndrome | Rate | 2014 | 65.27406811 | 136.1127203 | 29.98486204 |
| DALYs | Western Europe | Female | 15-49 years | Polycystic ovarian syndrome | Rate | 2015 | 65.38849942 | 136.3576774 | 30.01234214 |
| DALYs | Western Europe | Female | 15-49 years | Polycystic ovarian syndrome | Rate | 2016 | 65.54711705 | 136.943474 | 30.12760297 |
| DALYs | Western Europe | Female | 15-49 years | Polycystic ovarian syndrome | Rate | 2017 | 65.81931334 | 136.8661187 | 29.9736774 |
| DALYs | Western Europe | Female | 15-49 years | Polycystic ovarian syndrome | Rate | 2018 | 66.08185127 | 137.6093491 | 29.7574033 |
| DALYs | Western Europe | Female | 15-49 years | Polycystic ovarian syndrome | Rate | 2019 | 66.22434708 | 137.83206 | 29.6598061 |
| DALYs | Western Europe | Female | 15-49 years | Polycystic ovarian syndrome | Rate | 2020 | 66.47196274 | 138.7541993 | 29.94055159 |
| DALYs | Western Europe | Female | 15-49 years | Polycystic ovarian syndrome | Rate | 2021 | 66.74335755 | 138.6199939 | 30.23177254 |
| DALYs | Western Sub-Saharan Africa | Female | 15-49 years | Polycystic ovarian syndrome | Rate | 1990 | 8.283173343 | 17.37837046 | 3.509194135 |
| DALYs | Western Sub-Saharan Africa | Female | 15-49 years | Polycystic ovarian syndrome | Rate | 1991 | 8.639146268 | 18.18251909 | 3.679079667 |
| DALYs | Western Sub-Saharan Africa | Female | 15-49 years | Polycystic ovarian syndrome | Rate | 1992 | 8.986178046 | 18.80237796 | 3.836280374 |
| DALYs | Western Sub-Saharan Africa | Female | 15-49 years | Polycystic ovarian syndrome | Rate | 1993 | 9.306565922 | 19.59771686 | 3.954008233 |
| DALYs | Western Sub-Saharan Africa | Female | 15-49 years | Polycystic ovarian syndrome | Rate | 1994 | 9.596892307 | 20.10042922 | 4.075536184 |
| DALYs | Western Sub-Saharan Africa | Female | 15-49 years | Polycystic ovarian syndrome | Rate | 1995 | 9.85110009 | 20.56484622 | 4.182138483 |
| DALYs | Western Sub-Saharan Africa | Female | 15-49 years | Polycystic ovarian syndrome | Rate | 1996 | 10.09118668 | 21.11997322 | 4.289913154 |
| DALYs | Western Sub-Saharan Africa | Female | 15-49 years | Polycystic ovarian syndrome | Rate | 1997 | 10.32557008 | 21.5338486 | 4.399177551 |
| DALYs | Western Sub-Saharan Africa | Female | 15-49 years | Polycystic ovarian syndrome | Rate | 1998 | 10.54277312 | 22.17779671 | 4.512439718 |
| DALYs | Western Sub-Saharan Africa | Female | 15-49 years | Polycystic ovarian syndrome | Rate | 1999 | 10.73236708 | 22.56452849 | 4.613258212 |
| DALYs | Western Sub-Saharan Africa | Female | 15-49 years | Polycystic ovarian syndrome | Rate | 2000 | 10.88240683 | 22.74461387 | 4.685950443 |
| DALYs | Western Sub-Saharan Africa | Female | 15-49 years | Polycystic ovarian syndrome | Rate | 2001 | 11.00795675 | 23.151288 | 4.740575681 |
| DALYs | Western Sub-Saharan Africa | Female | 15-49 years | Polycystic ovarian syndrome | Rate | 2002 | 11.12807893 | 23.35236552 | 4.754411904 |
| DALYs | Western Sub-Saharan Africa | Female | 15-49 years | Polycystic ovarian syndrome | Rate | 2003 | 11.23517587 | 23.66220152 | 4.842493828 |
| DALYs | Western Sub-Saharan Africa | Female | 15-49 years | Polycystic ovarian syndrome | Rate | 2004 | 11.32583648 | 23.80930009 | 4.812353145 |
| DALYs | Western Sub-Saharan Africa | Female | 15-49 years | Polycystic ovarian syndrome | Rate | 2005 | 11.38057774 | 24.10851688 | 4.84274496 |
| DALYs | Western Sub-Saharan Africa | Female | 15-49 years | Polycystic ovarian syndrome | Rate | 2006 | 11.4198309 | 24.14021893 | 4.834879532 |
| DALYs | Western Sub-Saharan Africa | Female | 15-49 years | Polycystic ovarian syndrome | Rate | 2007 | 11.4598621 | 24.3133203 | 4.868987029 |
| DALYs | Western Sub-Saharan Africa | Female | 15-49 years | Polycystic ovarian syndrome | Rate | 2008 | 11.48630729 | 24.18752337 | 4.872913373 |
| DALYs | Western Sub-Saharan Africa | Female | 15-49 years | Polycystic ovarian syndrome | Rate | 2009 | 11.50826418 | 24.42815647 | 4.872153715 |
| DALYs | Western Sub-Saharan Africa | Female | 15-49 years | Polycystic ovarian syndrome | Rate | 2010 | 11.51652016 | 24.41003215 | 4.866475401 |
| DALYs | Western Sub-Saharan Africa | Female | 15-49 years | Polycystic ovarian syndrome | Rate | 2011 | 11.51209843 | 24.53183812 | 4.873270104 |
| DALYs | Western Sub-Saharan Africa | Female | 15-49 years | Polycystic ovarian syndrome | Rate | 2012 | 11.50688029 | 24.42546429 | 4.906281122 |
| DALYs | Western Sub-Saharan Africa | Female | 15-49 years | Polycystic ovarian syndrome | Rate | 2013 | 11.4934633 | 24.32218216 | 4.88950071 |
| DALYs | Western Sub-Saharan Africa | Female | 15-49 years | Polycystic ovarian syndrome | Rate | 2014 | 11.49522598 | 24.36636934 | 4.896053516 |
| DALYs | Western Sub-Saharan Africa | Female | 15-49 years | Polycystic ovarian syndrome | Rate | 2015 | 11.49857362 | 24.33751843 | 4.896533475 |
| DALYs | Western Sub-Saharan Africa | Female | 15-49 years | Polycystic ovarian syndrome | Rate | 2016 | 11.53660218 | 24.44912717 | 4.951429055 |
| DALYs | Western Sub-Saharan Africa | Female | 15-49 years | Polycystic ovarian syndrome | Rate | 2017 | 11.5989833 | 24.54750033 | 4.950184242 |
| DALYs | Western Sub-Saharan Africa | Female | 15-49 years | Polycystic ovarian syndrome | Rate | 2018 | 11.68962308 | 24.78854883 | 4.983827088 |
| DALYs | Western Sub-Saharan Africa | Female | 15-49 years | Polycystic ovarian syndrome | Rate | 2019 | 11.79073466 | 24.89604381 | 5.044290253 |
| DALYs | Western Sub-Saharan Africa | Female | 15-49 years | Polycystic ovarian syndrome | Rate | 2020 | 12.10379776 | 25.86246303 | 5.164194019 |
| DALYs | Western Sub-Saharan Africa | Female | 15-49 years | Polycystic ovarian syndrome | Rate | 2021 | 11.96402442 | 25.50857021 | 5.124847373 |
