# Supplementary material for: Global and Chinese epidemiologic study of polycystic ovary syndrome in women of childbearing age, 1990–2021, and projections to 2035: Based on the Global Burden of Disease 2021 study
Source: PLoS One. 2025 Aug 19;20(8):e0329090. doi: 10.1371/journal.pone.0329090 (PMC12364318; doi:10.1371/journal.pone.0329090)
Supplement: S4 Table — (DOCX) [file pone.0329090.s004.docx]

| **Supplementary Table 4** Global Age-Specific Incidence of Polycystic Ovary Syndrome in Women of Childbearing Age: 2021 Analysis | | | | | | | | | |
| --- | --- | --- | --- | --- | --- | --- | --- | --- | --- |
| **Measure** | **Location** | **Sex** | **Age** | **Cause** | **Metric** | **Year** | **Value** | **Upper** | **Lower** |
| Incidence | Global | Female | 15-19 | Polycystic ovarian syndrome | Rate | 2021 | 347.8838388 | 540.9528276 | 217.0667436 |
| Incidence | Global | Female | 20-24 | Polycystic ovarian syndrome | Rate | 2021 | 17.10195452 | 42.46314741 | 6.96978837 |
| Incidence | Global | Female | 25-29 | Polycystic ovarian syndrome | Rate | 2021 | 9.970961951 | 18.51889465 | 5.077731781 |
| Incidence | Global | Female | 30-34 | Polycystic ovarian syndrome | Rate | 2021 | 5.745086934 | 9.220708178 | 2.625157897 |
| Incidence | Global | Female | 35-39 | Polycystic ovarian syndrome | Rate | 2021 | 4.325016711 | 7.089551062 | 1.989440581 |
| Incidence | Global | Female | 40-44 | Polycystic ovarian syndrome | Rate | 2021 | 2.792062382 | 4.801866818 | 1.161843979 |
| Incidence | Global | Female | 45-49 | Polycystic ovarian syndrome | Rate | 2021 | 1.425049515 | 2.905830265 | 0.300142861 |
