# Supplementary material for: Global and Chinese epidemiologic study of polycystic ovary syndrome in women of childbearing age, 1990–2021, and projections to 2035: Based on the Global Burden of Disease 2021 study
Source: PLoS One. 2025 Aug 19;20(8):e0329090. doi: 10.1371/journal.pone.0329090 (PMC12364318; doi:10.1371/journal.pone.0329090)
Supplement: S5 Table — (DOCX) [file pone.0329090.s005.docx]

| **Supplementary Table 5** Global Age-Specific Prevalence of Polycystic Ovary Syndrome in Women of Childbearing Age: 2021 Analysis | | | | | | | | | |
| --- | --- | --- | --- | --- | --- | --- | --- | --- | --- |
| **Measure** | **Location** | **Sex** | **Age** | **Cause** | **Metric** | **Year** | **Value** | **Upper** | **Lower** |
| Prevalence | Global | Female | 15-19 | Polycystic ovarian syndrome | Rate | 2021 | 2523.762307 | 3613.353709 | 1707.001553 |
| Prevalence | Global | Female | 20-24 | Polycystic ovarian syndrome | Rate | 2021 | 3453.967098 | 4742.607514 | 2489.129465 |
| Prevalence | Global | Female | 25-29 | Polycystic ovarian syndrome | Rate | 2021 | 3581.85829 | 4968.037535 | 2571.900831 |
| Prevalence | Global | Female | 30-34 | Polycystic ovarian syndrome | Rate | 2021 | 3600.498921 | 5007.276724 | 2579.791261 |
| Prevalence | Global | Female | 35-39 | Polycystic ovarian syndrome | Rate | 2021 | 3644.971495 | 5067.778295 | 2588.053706 |
| Prevalence | Global | Female | 40-44 | Polycystic ovarian syndrome | Rate | 2021 | 3687.029486 | 5121.839746 | 2637.99138 |
| Prevalence | Global | Female | 45-49 | Polycystic ovarian syndrome | Rate | 2021 | 3182.497736 | 4407.064153 | 2297.848656 |
