# Supplementary material for: Global and Chinese epidemiologic study of polycystic ovary syndrome in women of childbearing age, 1990–2021, and projections to 2035: Based on the Global Burden of Disease 2021 study
Source: PLoS One. 2025 Aug 19;20(8):e0329090. doi: 10.1371/journal.pone.0329090 (PMC12364318; doi:10.1371/journal.pone.0329090)
Supplement: S6 Table — (DOCX) [file pone.0329090.s006.docx]

| **Supplementary Table 6** Global Age-Specific DALYs of Polycystic Ovary Syndrome in Women of Childbearing Age: 2021 Analysis | | | | | | | | | |
| --- | --- | --- | --- | --- | --- | --- | --- | --- | --- |
| **Measure** | **Location** | **Sex** | **Age** | **Cause** | **Metric** | **Year** | **Value** | **Upper** | **Lower** |
| DALYs | Global | Female | 15-19 | Polycystic ovarian syndrome | Rate | 2021 | 22.66166163 | 48.03119432 | 9.998393999 |
| DALYs | Global | Female | 20-24 | Polycystic ovarian syndrome | Rate | 2021 | 31.71089417 | 65.97175936 | 14.19723622 |
| DALYs | Global | Female | 25-29 | Polycystic ovarian syndrome | Rate | 2021 | 32.14130321 | 65.96346393 | 14.15096383 |
| DALYs | Global | Female | 30-34 | Polycystic ovarian syndrome | Rate | 2021 | 31.20068184 | 66.47592192 | 13.74606254 |
| DALYs | Global | Female | 35-39 | Polycystic ovarian syndrome | Rate | 2021 | 31.01636601 | 65.23136898 | 13.70400209 |
| DALYs | Global | Female | 40-44 | Polycystic ovarian syndrome | Rate | 2021 | 31.24477389 | 65.22702514 | 13.90479247 |
| DALYs | Global | Female | 45-49 | Polycystic ovarian syndrome | Rate | 2021 | 26.99285642 | 53.82979556 | 12.16960423 |
