# Supplementary material for: Global and Chinese epidemiologic study of polycystic ovary syndrome in women of childbearing age, 1990–2021, and projections to 2035: Based on the Global Burden of Disease 2021 study
Source: PLoS One. 2025 Aug 19;20(8):e0329090. doi: 10.1371/journal.pone.0329090 (PMC12364318; doi:10.1371/journal.pone.0329090)
Supplement: S7 Table — (DOCX) [file pone.0329090.s007.docx]

| **Supplementary Table 7** Global 2035 Projected Incidence Analysis of Polycystic Ovary Syndrome in Women of Reproductive Age | | | | |
| --- | --- | --- | --- | --- |
| Value | Time | Group | Low | Up |
| 273.9933695 | 1990 | 15-19 | 257.36495 | 290.621789 |
| 275.9613637 | 1991 | 15-19 | 259.3523456 | 292.5703817 |
| 278.0628363 | 1992 | 15-19 | 261.4744009 | 294.6512716 |
| 280.1082524 | 1993 | 15-19 | 263.5397642 | 296.6767406 |
| 282.0112848 | 1994 | 15-19 | 265.4612751 | 298.5612945 |
| 283.7516368 | 1995 | 15-19 | 267.218448 | 300.2848256 |
| 285.6273899 | 1996 | 15-19 | 269.1122304 | 302.1425493 |
| 287.8183566 | 1997 | 15-19 | 271.3241179 | 304.3125953 |
| 289.8788063 | 1998 | 15-19 | 273.4041674 | 306.3534452 |
| 291.3699249 | 1999 | 15-19 | 274.909484 | 307.8303658 |
| 292.0090174 | 2000 | 15-19 | 275.5547999 | 308.4632349 |
| 292.5781511 | 2001 | 15-19 | 276.1295341 | 309.0267681 |
| 293.7794563 | 2002 | 15-19 | 277.3423439 | 310.2165687 |
| 295.4137096 | 2003 | 15-19 | 278.9920783 | 311.8353409 |
| 297.26653 | 2004 | 15-19 | 280.8622803 | 313.6707798 |
| 298.9712404 | 2005 | 15-19 | 282.582849 | 315.3596318 |
| 300.8696319 | 2006 | 15-19 | 284.4986334 | 317.2406303 |
| 303.349956 | 2007 | 15-19 | 287.0013468 | 319.6985652 |
| 306.1209186 | 2008 | 15-19 | 289.797101 | 322.4447363 |
| 308.9752428 | 2009 | 15-19 | 292.6767688 | 325.2737169 |
| 311.5582243 | 2010 | 15-19 | 295.2825696 | 327.833879 |
| 314.0355449 | 2011 | 15-19 | 297.7817094 | 330.2893803 |
| 316.7596846 | 2012 | 15-19 | 300.52973 | 332.9896391 |
| 319.576871 | 2013 | 15-19 | 303.3714778 | 335.7822642 |
| 322.2301858 | 2014 | 15-19 | 306.0478822 | 338.4124893 |
| 324.6166867 | 2015 | 15-19 | 308.455258 | 340.7781154 |
| 327.6480588 | 2016 | 15-19 | 311.5130039 | 343.7831137 |
| 331.8470547 | 2017 | 15-19 | 315.7479938 | 347.9461156 |
| 336.4518495 | 2018 | 15-19 | 320.3916518 | 352.5120472 |
| 340.7541012 | 2019 | 15-19 | 324.7297409 | 356.7784615 |
| 346.3159574 | 2020 | 15-19 | 330.3367284 | 362.2951863 |
| 347.8888951 | 2021 | 15-19 | 331.9227116 | 363.8550786 |
| 356.3925414 | 2022 | 15-19 | 324.6446859 | 388.1403969 |
| 363.3913377 | 2023 | 15-19 | 323.8110003 | 402.9716751 |
| 370.549322 | 2024 | 15-19 | 318.19807 | 422.900574 |
| 377.8751479 | 2025 | 15-19 | 310.1121989 | 445.6380969 |
| 385.3780901 | 2026 | 15-19 | 301.9171531 | 468.8390272 |
| 393.0679938 | 2027 | 15-19 | 294.9127645 | 491.223223 |
| 400.9552272 | 2028 | 15-19 | 289.5007874 | 512.409667 |
| 409.0506935 | 2029 | 15-19 | 285.6070094 | 532.4943775 |
| 417.3658712 | 2030 | 15-19 | 282.9628524 | 551.7688901 |
| 425.9128654 | 2031 | 15-19 | 281.2457153 | 570.5800156 |
| 434.7044354 | 2032 | 15-19 | 280.1383853 | 589.2704856 |
| 443.7540588 | 2033 | 15-19 | 279.3505599 | 608.1575577 |
| 453.0760534 | 2034 | 15-19 | 278.6224871 | 627.5296197 |
| 462.6854225 | 2035 | 15-19 | 277.7290607 | 647.6417842 |
| 17.10159986 | 1990 | 20-24 | -7.52347899 | 41.72667871 |
| 17.13465111 | 1991 | 20-24 | -7.49985166 | 41.76915388 |
| 17.15126299 | 1992 | 20-24 | -7.474558352 | 41.77708433 |
| 17.15649064 | 1993 | 20-24 | -7.4670294 | 41.78001069 |
| 17.1491886 | 1994 | 20-24 | -7.48550013 | 41.78387734 |
| 17.12750578 | 1995 | 20-24 | -7.528150573 | 41.78316213 |
| 17.08507431 | 1996 | 20-24 | -7.579685407 | 41.74983402 |
| 17.02206912 | 1997 | 20-24 | -7.636337766 | 41.68047601 |
| 16.95480277 | 1998 | 20-24 | -7.686111376 | 41.59571691 |
| 16.8994167 | 1999 | 20-24 | -7.719675113 | 41.51850852 |
| 16.86993229 | 2000 | 20-24 | -7.729134538 | 41.46899913 |
| 16.87171526 | 2001 | 20-24 | -7.708351171 | 41.45178169 |
| 16.89928619 | 2002 | 20-24 | -7.659162176 | 41.45773456 |
| 16.94552428 | 2003 | 20-24 | -7.587797269 | 41.47884583 |
| 17.00504739 | 2004 | 20-24 | -7.504834381 | 41.51492915 |
| 17.06668611 | 2005 | 20-24 | -7.424529436 | 41.55790165 |
| 17.12500491 | 2006 | 20-24 | -7.352361593 | 41.60237141 |
| 17.17606899 | 2007 | 20-24 | -7.290444019 | 41.642582 |
| 17.20982813 | 2008 | 20-24 | -7.244420222 | 41.66407649 |
| 17.22143529 | 2009 | 20-24 | -7.218569831 | 41.6614404 |
| 17.21433418 | 2010 | 20-24 | -7.210990264 | 41.63965863 |
| 17.17877931 | 2011 | 20-24 | -7.226166119 | 41.58372474 |
| 17.1147207 | 2012 | 20-24 | -7.260977233 | 41.49041864 |
| 17.04547888 | 2013 | 20-24 | -7.314930441 | 41.40588821 |
| 16.99093965 | 2014 | 20-24 | -7.353039493 | 41.3349188 |
| 16.96964969 | 2015 | 20-24 | -7.354866399 | 41.29416579 |
| 17.00695585 | 2016 | 20-24 | -7.295197074 | 41.30910877 |
| 17.09085578 | 2017 | 20-24 | -7.185215159 | 41.36692671 |
| 17.18138669 | 2018 | 20-24 | -7.065718433 | 41.42849181 |
| 17.23860244 | 2019 | 20-24 | -6.976075935 | 41.45328081 |
| 17.16412787 | 2020 | 20-24 | -7.002821082 | 41.33107683 |
| 17.16948081 | 2021 | 20-24 | -6.952260301 | 41.29122193 |
| 18.55190236 | 2022 | 20-24 | -18.96126959 | 56.06507431 |
| 18.91654984 | 2023 | 20-24 | -17.72216456 | 55.55526425 |
| 19.29264324 | 2024 | 20-24 | -18.42114202 | 57.0064285 |
| 19.67372766 | 2025 | 20-24 | -20.58741497 | 59.93487029 |
| 20.05850502 | 2026 | 20-24 | -22.84060011 | 62.95761015 |
| 20.45151731 | 2027 | 20-24 | -24.90743739 | 65.810472 |
| 20.85309695 | 2028 | 20-24 | -30.49572595 | 72.20191985 |
| 21.26355435 | 2029 | 20-24 | -42.62940742 | 85.15651612 |
| 21.68327676 | 2030 | 20-24 | -60.33436355 | 103.7009171 |
| 22.11280187 | 2031 | 20-24 | -80.4005626 | 124.6261664 |
| 22.55280339 | 2032 | 20-24 | -100.0773525 | 145.1829592 |
| 23.00401394 | 2033 | 20-24 | -117.8839428 | 163.8919707 |
| 23.46717299 | 2034 | 20-24 | -133.2845402 | 180.2188861 |
| 23.94301436 | 2035 | 20-24 | -146.2528098 | 194.1388386 |
| 9.857615154 | 1990 | 25-29 | -16.52294285 | 36.23817316 |
| 9.935345019 | 1991 | 25-29 | -16.47897159 | 36.34966163 |
| 9.996632133 | 1992 | 25-29 | -16.37459749 | 36.36786176 |
| 10.03535716 | 1993 | 25-29 | -16.2602666 | 36.33098092 |
| 10.0574221 | 1994 | 25-29 | -16.15541753 | 36.27026173 |
| 10.06667598 | 1995 | 25-29 | -16.06712071 | 36.20047266 |
| 10.05727613 | 1996 | 25-29 | -16.00293357 | 36.11748584 |
| 10.03634372 | 1997 | 25-29 | -15.97576007 | 36.0484475 |
| 10.01329934 | 1998 | 25-29 | -15.99595443 | 36.02255311 |
| 9.990388842 | 1999 | 25-29 | -16.06004232 | 36.04082 |
| 9.972702168 | 2000 | 25-29 | -16.15123934 | 36.09664368 |
| 9.965961668 | 2001 | 25-29 | -16.21735427 | 36.1492776 |
| 9.966258195 | 2002 | 25-29 | -16.24455422 | 36.17707061 |
| 9.968789027 | 2003 | 25-29 | -16.23575575 | 36.17333381 |
| 9.969734731 | 2004 | 25-29 | -16.20573865 | 36.14520811 |
| 9.963267274 | 2005 | 25-29 | -16.16506529 | 36.09159984 |
| 9.948110439 | 2006 | 25-29 | -16.11862793 | 36.01484881 |
| 9.932116808 | 2007 | 25-29 | -16.07165086 | 35.93588448 |
| 9.920139656 | 2008 | 25-29 | -16.02245323 | 35.86273254 |
| 9.919529275 | 2009 | 25-29 | -15.98123595 | 35.8202945 |
| 9.934018645 | 2010 | 25-29 | -15.95616999 | 35.82420728 |
| 9.952136154 | 2011 | 25-29 | -15.93982202 | 35.84409432 |
| 9.960633281 | 2012 | 25-29 | -15.92458794 | 35.8458545 |
| 9.960352442 | 2013 | 25-29 | -15.90922839 | 35.82993327 |
| 9.955836794 | 2014 | 25-29 | -15.90209482 | 35.81376841 |
| 9.954636547 | 2015 | 25-29 | -15.89648036 | 35.80575345 |
| 9.976685956 | 2016 | 25-29 | -15.87659969 | 35.8299716 |
| 10.02092819 | 2017 | 25-29 | -15.85464428 | 35.89650065 |
| 10.06473223 | 2018 | 25-29 | -15.83551673 | 35.96498119 |
| 10.08507027 | 2019 | 25-29 | -15.82182301 | 35.99196355 |
| 10.00667317 | 2020 | 25-29 | -15.80021807 | 35.81356441 |
| 9.990743398 | 2021 | 25-29 | -15.77448207 | 35.75596886 |
| 10.24233076 | 2022 | 25-29 | -30.6791899 | 51.16385141 |
| 10.33482726 | 2023 | 25-29 | -29.9981645 | 50.66781903 |
| 10.44575141 | 2024 | 25-29 | -31.39852614 | 52.29002895 |
| 10.58130624 | 2025 | 25-29 | -34.51674367 | 55.67935616 |
| 10.74866953 | 2026 | 25-29 | -38.88210998 | 60.37944905 |
| 10.94796327 | 2027 | 25-29 | -44.16847797 | 66.06440451 |
| 11.16295603 | 2028 | 25-29 | -50.74198142 | 73.06789347 |
| 11.38466002 | 2029 | 25-29 | -58.36954559 | 81.13886564 |
| 11.60933257 | 2030 | 25-29 | -65.15630282 | 88.37496796 |
| 11.83639317 | 2031 | 25-29 | -67.01264133 | 90.68542767 |
| 12.06861025 | 2032 | 25-29 | -62.29204489 | 86.42926539 |
| 12.3059416 | 2033 | 25-29 | -57.5424797 | 82.1543629 |
| 12.54837653 | 2034 | 25-29 | -59.83175126 | 84.92850431 |
| 12.79608807 | 2035 | 25-29 | -70.87193013 | 96.46410627 |
| 5.589340201 | 1990 | 30-34 | -22.87392045 | 34.05260085 |
| 5.590614997 | 1991 | 30-34 | -22.89804699 | 34.07927698 |
| 5.604657009 | 1992 | 30-34 | -22.80125701 | 34.01057102 |
| 5.632255275 | 1993 | 30-34 | -22.70048335 | 33.9649939 |
| 5.667066335 | 1994 | 30-34 | -22.59035893 | 33.9244916 |
| 5.703044438 | 1995 | 30-34 | -22.46667809 | 33.87276697 |
| 5.732565847 | 1996 | 30-34 | -22.32994965 | 33.79508135 |
| 5.748894079 | 1997 | 30-34 | -22.1596397 | 33.65742786 |
| 5.753472571 | 1998 | 30-34 | -21.97747184 | 33.48441698 |
| 5.752738725 | 1999 | 30-34 | -21.80836801 | 33.31384546 |
| 5.753384445 | 2000 | 30-34 | -21.6844567 | 33.19122559 |
| 5.759602272 | 2001 | 30-34 | -21.64806799 | 33.16727254 |
| 5.773141922 | 2002 | 30-34 | -21.7157176 | 33.26200144 |
| 5.789011289 | 2003 | 30-34 | -21.86380175 | 33.44182432 |
| 5.800021041 | 2004 | 30-34 | -22.05811218 | 33.65815427 |
| 5.798137505 | 2005 | 30-34 | -22.23674225 | 33.83301727 |
| 5.782019823 | 2006 | 30-34 | -22.30100423 | 33.86504388 |
| 5.755560513 | 2007 | 30-34 | -22.25616411 | 33.76728514 |
| 5.726464593 | 2008 | 30-34 | -22.14977922 | 33.6027084 |
| 5.700043021 | 2009 | 30-34 | -22.02392407 | 33.42401011 |
| 5.682737171 | 2010 | 30-34 | -21.94530881 | 33.31078315 |
| 5.668554445 | 2011 | 30-34 | -21.88025216 | 33.21736105 |
| 5.653620444 | 2012 | 30-34 | -21.80226592 | 33.1095068 |
| 5.642305474 | 2013 | 30-34 | -21.73367313 | 33.01828408 |
| 5.640857294 | 2014 | 30-34 | -21.72297609 | 33.00469067 |
| 5.652451664 | 2015 | 30-34 | -21.74379315 | 33.04869648 |
| 5.688622472 | 2016 | 30-34 | -21.90038374 | 33.27762868 |
| 5.74400987 | 2017 | 30-34 | -22.12793225 | 33.61595199 |
| 5.79761383 | 2018 | 30-34 | -22.33104089 | 33.92626855 |
| 5.827791003 | 2019 | 30-34 | -22.46383882 | 34.11942082 |
| 5.770034304 | 2020 | 30-34 | -22.23757245 | 33.77764106 |
| 5.744327828 | 2021 | 30-34 | -22.09076057 | 33.57941623 |
| 5.75643766 | 2022 | 30-34 | -37.73353046 | 49.24640578 |
| 5.774335445 | 2023 | 30-34 | -36.69403872 | 48.24270961 |
| 5.795918495 | 2024 | 30-34 | -37.96045589 | 49.55229288 |
| 5.822750768 | 2025 | 30-34 | -41.38213122 | 53.02763276 |
| 5.857434852 | 2026 | 30-34 | -46.48599189 | 58.2008616 |
| 5.901571635 | 2027 | 30-34 | -52.830409 | 64.63355227 |
| 5.954619174 | 2028 | 30-34 | -60.6469211 | 72.55615945 |
| 6.018222153 | 2029 | 30-34 | -69.87744061 | 81.91388492 |
| 6.095954837 | 2030 | 30-34 | -80.0150591 | 92.20696878 |
| 6.191978035 | 2031 | 30-34 | -90.25330788 | 102.637264 |
| 6.30639562 | 2032 | 30-34 | -99.97013561 | 112.5829268 |
| 6.429819967 | 2033 | 30-34 | -110.0269186 | 122.8865586 |
| 6.55707604 | 2034 | 30-34 | -120.3225398 | 133.4366919 |
| 6.686185196 | 2035 | 30-34 | -127.8693475 | 141.2417179 |
| 4.257320973 | 1990 | 35-39 | -25.32883056 | 33.84347251 |
| 4.264656885 | 1991 | 35-39 | -25.53440493 | 34.0637187 |
| 4.267469368 | 1992 | 35-39 | -25.54493502 | 34.07987376 |
| 4.261914506 | 1993 | 35-39 | -25.39303307 | 33.91686208 |
| 4.253070993 | 1994 | 35-39 | -25.1910292 | 33.69717119 |
| 4.244713628 | 1995 | 35-39 | -24.98194282 | 33.47137008 |
| 4.23907719 | 1996 | 35-39 | -24.77237877 | 33.25053315 |
| 4.239306907 | 1997 | 35-39 | -24.5899483 | 33.06856212 |
| 4.248795336 | 1998 | 35-39 | -24.48427947 | 32.98187014 |
| 4.264797987 | 1999 | 35-39 | -24.39254723 | 32.9221432 |
| 4.28655866 | 2000 | 35-39 | -24.31477336 | 32.88789068 |
| 4.315365326 | 2001 | 35-39 | -24.32293445 | 32.9536651 |
| 4.345937863 | 2002 | 35-39 | -24.36624852 | 33.05812425 |
| 4.372005225 | 2003 | 35-39 | -24.37162755 | 33.115638 |
| 4.391217305 | 2004 | 35-39 | -24.33244572 | 33.11488033 |
| 4.399180258 | 2005 | 35-39 | -24.21400103 | 33.01236154 |
| 4.392905782 | 2006 | 35-39 | -24.0223464 | 32.80815796 |
| 4.379009293 | 2007 | 35-39 | -23.87200323 | 32.63002182 |
| 4.362634235 | 2008 | 35-39 | -23.83916403 | 32.5644325 |
| 4.34633689 | 2009 | 35-39 | -23.95136443 | 32.64403821 |
| 4.333029313 | 2010 | 35-39 | -24.21130864 | 32.87736727 |
| 4.319424134 | 2011 | 35-39 | -24.4236945 | 33.06254276 |
| 4.300325709 | 2012 | 35-39 | -24.49263975 | 33.09329117 |
| 4.280732096 | 2013 | 35-39 | -24.47895584 | 33.04042004 |
| 4.265066335 | 2014 | 35-39 | -24.45286889 | 32.98300156 |
| 4.25774357 | 2015 | 35-39 | -24.47788231 | 32.99336945 |
| 4.267886261 | 2016 | 35-39 | -24.67176543 | 33.20753796 |
| 4.294327037 | 2017 | 35-39 | -25.00822102 | 33.5968751 |
| 4.324755926 | 2018 | 35-39 | -25.33781447 | 33.98732632 |
| 4.347510827 | 2019 | 35-39 | -25.59689919 | 34.29192085 |
| 4.320466331 | 2020 | 35-39 | -25.31260955 | 33.95354222 |
| 4.31516528 | 2021 | 35-39 | -25.12027051 | 33.75060107 |
| 4.294126612 | 2022 | 35-39 | -40.67116066 | 49.25941389 |
| 4.306578208 | 2023 | 35-39 | -39.35894742 | 47.97210384 |
| 4.317828616 | 2024 | 35-39 | -40.2845698 | 48.92022704 |
| 4.327696921 | 2025 | 35-39 | -43.3694698 | 52.02486364 |
| 4.337575715 | 2026 | 35-39 | -48.25462192 | 56.92977335 |
| 4.348768172 | 2027 | 35-39 | -54.58396468 | 63.28150102 |
| 4.362026656 | 2028 | 35-39 | -62.5152372 | 71.23929051 |
| 4.378013341 | 2029 | 35-39 | -72.04226404 | 80.79829072 |
| 4.397907546 | 2030 | 35-39 | -82.70506451 | 91.5008796 |
| 4.423702622 | 2031 | 35-39 | -93.58662645 | 102.4340317 |
| 4.456633807 | 2032 | 35-39 | -104.1664619 | 113.0797295 |
| 4.496259011 | 2033 | 35-39 | -115.2898141 | 124.2823322 |
| 4.543808753 | 2034 | 35-39 | -127.1633654 | 136.2509829 |
| 4.602007712 | 2035 | 35-39 | -139.2525468 | 148.4565622 |
| 2.740383398 | 1990 | 40-44 | -28.24181893 | 33.72258572 |
| 2.747636125 | 1991 | 40-44 | -28.57614501 | 34.07141726 |
| 2.757698199 | 1992 | 40-44 | -28.7787629 | 34.2941593 |
| 2.768833895 | 1993 | 40-44 | -28.96411467 | 34.50178246 |
| 2.775633611 | 1994 | 40-44 | -28.95261189 | 34.50387911 |
| 2.781935437 | 1995 | 40-44 | -29.02203931 | 34.58591018 |
| 2.779955805 | 1996 | 40-44 | -28.86447243 | 34.42438404 |
| 2.772254262 | 1997 | 40-44 | -28.59464373 | 34.13915226 |
| 2.759876654 | 1998 | 40-44 | -28.20608869 | 33.725842 |
| 2.747388048 | 1999 | 40-44 | -27.83202096 | 33.32679705 |
| 2.739702689 | 2000 | 40-44 | -27.60586187 | 33.08526724 |
| 2.740212972 | 2001 | 40-44 | -27.57764154 | 33.05806748 |
| 2.750532886 | 2002 | 40-44 | -27.74602843 | 33.2470942 |
| 2.768485042 | 2003 | 40-44 | -27.99033935 | 33.52730944 |
| 2.789189634 | 2004 | 40-44 | -28.13981714 | 33.71819641 |
| 2.80737075 | 2005 | 40-44 | -28.08692067 | 33.70166217 |
| 2.819290763 | 2006 | 40-44 | -27.85625165 | 33.49483317 |
| 2.823946383 | 2007 | 40-44 | -27.47796765 | 33.12586042 |
| 2.822922125 | 2008 | 40-44 | -27.04135449 | 32.68719874 |
| 2.8198818 | 2009 | 40-44 | -26.66680032 | 32.30656392 |
| 2.81814024 | 2010 | 40-44 | -26.49039368 | 32.12667416 |
| 2.814039472 | 2011 | 40-44 | -26.43359738 | 32.06167633 |
| 2.807401812 | 2012 | 40-44 | -26.52602751 | 32.14083114 |
| 2.799793366 | 2013 | 40-44 | -26.82119687 | 32.42078361 |
| 2.792451152 | 2014 | 40-44 | -27.32790693 | 32.91280924 |
| 2.787125856 | 2015 | 40-44 | -27.9986064 | 33.57285811 |
| 2.790133458 | 2016 | 40-44 | -28.8032974 | 34.38356432 |
| 2.799677454 | 2017 | 40-44 | -29.6143297 | 35.21368461 |
| 2.809568291 | 2018 | 40-44 | -30.29148815 | 35.91062473 |
| 2.813052597 | 2019 | 40-44 | -30.75870595 | 36.38481114 |
| 2.790342445 | 2020 | 40-44 | -30.36990132 | 35.95058621 |
| 2.775377523 | 2021 | 40-44 | -29.8252412 | 35.37599624 |
| 2.744557881 | 2022 | 40-44 | -44.45733685 | 49.94645261 |
| 2.749478299 | 2023 | 40-44 | -43.01060632 | 48.50956292 |
| 2.755847949 | 2024 | 40-44 | -43.90725955 | 49.41895545 |
| 2.763018637 | 2025 | 40-44 | -47.00637608 | 52.53241336 |
| 2.770794203 | 2026 | 40-44 | -51.88207605 | 57.42366446 |
| 2.778793965 | 2027 | 40-44 | -58.10815301 | 63.66574094 |
| 2.786594798 | 2028 | 40-44 | -65.76039138 | 71.33358098 |
| 2.793564194 | 2029 | 40-44 | -74.9099546 | 80.49708299 |
| 2.799576182 | 2030 | 40-44 | -85.30928455 | 90.90843692 |
| 2.805549264 | 2031 | 40-44 | -96.22755936 | 101.8386579 |
| 2.812346163 | 2032 | 40-44 | -107.1932797 | 112.817972 |
| 2.820425783 | 2033 | 40-44 | -118.8578235 | 124.4986751 |
| 2.830206157 | 2034 | 40-44 | -131.3796971 | 137.0401094 |
| 2.842477779 | 2035 | 40-44 | -144.1955806 | 149.8805362 |
| 1.38462987 | 1990 | 45-49 | -32.76789369 | 35.53715343 |
| 1.388338342 | 1991 | 45-49 | -32.73280178 | 35.50947847 |
| 1.390638332 | 1992 | 45-49 | -33.56605046 | 36.34732712 |
| 1.39230144 | 1993 | 45-49 | -33.98647996 | 36.77108284 |
| 1.394698382 | 1994 | 45-49 | -34.23654726 | 37.02594403 |
| 1.395246302 | 1995 | 45-49 | -34.00045152 | 36.79094413 |
| 1.396068366 | 1996 | 45-49 | -33.8103663 | 36.60250303 |
| 1.396535885 | 1997 | 45-49 | -33.66305759 | 36.45612936 |
| 1.396970243 | 1998 | 45-49 | -33.59639845 | 36.39033893 |
| 1.396655111 | 1999 | 45-49 | -33.4596879 | 36.25299812 |
| 1.397257667 | 2000 | 45-49 | -33.56447932 | 36.35899466 |
| 1.397757738 | 2001 | 45-49 | -33.70907779 | 36.50459326 |
| 1.398646481 | 2002 | 45-49 | -33.98002035 | 36.77731332 |
| 1.398273205 | 2003 | 45-49 | -34.03255279 | 36.8290992 |
| 1.397233377 | 2004 | 45-49 | -33.98309754 | 36.77756429 |
| 1.395345806 | 2005 | 45-49 | -33.75043521 | 36.54112683 |
| 1.393168948 | 2006 | 45-49 | -33.32620841 | 36.11254631 |
| 1.391681652 | 2007 | 45-49 | -32.74426063 | 35.52762394 |
| 1.392263887 | 2008 | 45-49 | -32.22180382 | 35.00633159 |
| 1.394723238 | 2009 | 45-49 | -31.73624326 | 34.52568973 |
| 1.399661542 | 2010 | 45-49 | -31.58940162 | 34.3887247 |
| 1.405029133 | 2011 | 45-49 | -31.65775858 | 34.46781684 |
| 1.409079457 | 2012 | 45-49 | -31.82191309 | 34.640072 |
| 1.411273641 | 2013 | 45-49 | -31.97802236 | 34.80056965 |
| 1.41278444 | 2014 | 45-49 | -32.20657578 | 35.03214466 |
| 1.414058526 | 2015 | 45-49 | -32.45970782 | 35.28782487 |
| 1.416963233 | 2016 | 45-49 | -33.08594608 | 35.91987255 |
| 1.42182554 | 2017 | 45-49 | -34.24678203 | 37.09043311 |
| 1.425822595 | 2018 | 45-49 | -35.70883754 | 38.56048273 |
| 1.425906395 | 2019 | 45-49 | -37.26439246 | 40.11620525 |
| 1.415739705 | 2020 | 45-49 | -37.84519354 | 40.67667295 |
| 1.405867436 | 2021 | 45-49 | -37.48715235 | 40.29888722 |
| 1.38348761 | 2022 | 45-49 | -49.13806171 | 51.90503692 |
| 1.381015653 | 2023 | 45-49 | -47.4250486 | 50.1870799 |
| 1.379564032 | 2024 | 45-49 | -47.94088452 | 50.70001258 |
| 1.378955221 | 2025 | 45-49 | -50.67301882 | 53.43092926 |
| 1.379246304 | 2026 | 45-49 | -55.3635951 | 58.12208771 |
| 1.380475129 | 2027 | 45-49 | -61.69925901 | 64.46020927 |
| 1.382685835 | 2028 | 45-49 | -69.71631318 | 72.48168485 |
| 1.385561745 | 2029 | 45-49 | -79.35170162 | 82.12282511 |
| 1.388772792 | 2030 | 45-49 | -90.17848525 | 92.95603084 |
| 1.392241937 | 2031 | 45-49 | -101.3581632 | 104.142647 |
| 1.395800728 | 2032 | 45-49 | -112.3553827 | 115.1469842 |
| 1.399210752 | 2033 | 45-49 | -123.7793387 | 126.5777602 |
| 1.402134501 | 2034 | 45-49 | -135.9685064 | 138.7727754 |
| 1.404518119 | 2035 | 45-49 | -148.6701758 | 151.4792121 |
