# Supplementary material for: Global and Chinese epidemiologic study of polycystic ovary syndrome in women of childbearing age, 1990–2021, and projections to 2035: Based on the Global Burden of Disease 2021 study
Source: PLoS One. 2025 Aug 19;20(8):e0329090. doi: 10.1371/journal.pone.0329090 (PMC12364318; doi:10.1371/journal.pone.0329090)
Supplement: S8 Table — (DOCX) [file pone.0329090.s008.docx]

| **Supplementary Table 8** Analysis of the Projected global overall age-standardized incidence of polycystic ovary syndrome in women of reproductive age in 2035 | | | | |
| --- | --- | --- | --- | --- |
| Value | Time | Group | Low | Up |
| 52.10395023 | 1990 | ASR | 51.98955605 | 52.21834441 |
| 52.45386822 | 1991 | ASR | 52.33934584 | 52.5683906 |
| 52.82249989 | 1992 | ASR | 52.70765106 | 52.93734871 |
| 53.17736366 | 1993 | ASR | 53.06223226 | 53.29249507 |
| 53.50394537 | 1994 | ASR | 53.38871411 | 53.61917663 |
| 53.79895144 | 1995 | ASR | 53.68385738 | 53.91404549 |
| 54.10894105 | 1996 | ASR | 53.99412072 | 54.22376138 |
| 54.46489131 | 1997 | ASR | 54.35041286 | 54.57936975 |
| 54.79689473 | 1998 | ASR | 54.68290799 | 54.91088146 |
| 55.03528828 | 1999 | ASR | 54.92200163 | 55.14857493 |
| 55.13732471 | 2000 | ASR | 55.02498099 | 55.24966842 |
| 55.23705849 | 2001 | ASR | 55.12576639 | 55.34835059 |
| 55.45061591 | 2002 | ASR | 55.3402487 | 55.56098313 |
| 55.74064949 | 2003 | ASR | 55.63099652 | 55.85030246 |
| 56.06782513 | 2004 | ASR | 55.95863813 | 56.17701213 |
| 56.36548769 | 2005 | ASR | 56.25653209 | 56.4744433 |
| 56.68891826 | 2006 | ASR | 56.57985645 | 56.79798007 |
| 57.10531318 | 2007 | ASR | 56.99581477 | 57.21481158 |
| 57.567145 | 2008 | ASR | 57.45700256 | 57.67728744 |
| 58.0415656 | 2009 | ASR | 57.93066869 | 58.15246251 |
| 58.47200985 | 2010 | ASR | 58.36035035 | 58.58366935 |
| 58.88094906 | 2011 | ASR | 58.76862089 | 58.99327723 |
| 59.32397649 | 2012 | ASR | 59.21105593 | 59.43689705 |
| 59.78058437 | 2013 | ASR | 59.6671251 | 59.89404364 |
| 60.21333127 | 2014 | ASR | 60.09940394 | 60.32725861 |
| 60.61038869 | 2015 | ASR | 60.49609507 | 60.7246823 |
| 61.13575809 | 2016 | ASR | 61.021029 | 61.25048718 |
| 61.87394676 | 2017 | ASR | 61.75864973 | 61.98924378 |
| 62.68143836 | 2018 | ASR | 62.565609 | 62.79726773 |
| 63.42353146 | 2019 | ASR | 63.30734358 | 63.53971935 |
| 64.31668342 | 2020 | ASR | 64.20008277 | 64.43328407 |
| 64.5715234 | 2021 | ASR | 64.4551581 | 64.68788869 |
| 66.23026656 | 2022 | ASR | 63.35793954 | 69.10259359 |
| 67.47356877 | 2023 | ASR | 63.99911398 | 70.94802356 |
| 68.74500916 | 2024 | ASR | 64.50501629 | 72.98500203 |
| 70.04504559 | 2025 | ASR | 64.88523506 | 75.20485612 |
| 71.37554336 | 2026 | ASR | 65.14927747 | 77.60180925 |
| 72.7380208 | 2027 | ASR | 65.30389436 | 80.17214725 |
| 74.13046634 | 2028 | ASR | 65.35113556 | 82.90979713 |
| 75.55223012 | 2029 | ASR | 65.29192873 | 85.81253151 |
| 77.00386838 | 2030 | ASR | 65.1265997 | 88.88113705 |
| 78.48674388 | 2031 | ASR | 64.85505356 | 92.11843421 |
| 80.00203072 | 2032 | ASR | 64.47633123 | 95.5277302 |
| 81.54889305 | 2033 | ASR | 63.98748137 | 99.11030473 |
| 83.12732649 | 2034 | ASR | 63.38565938 | 102.8689936 |
| 84.73803914 | 2035 | ASR | 62.66814659 | 106.8079317 |
