# Supplementary material for: Global and Chinese epidemiologic study of polycystic ovary syndrome in women of childbearing age, 1990–2021, and projections to 2035: Based on the Global Burden of Disease 2021 study
Source: PLoS One. 2025 Aug 19;20(8):e0329090. doi: 10.1371/journal.pone.0329090 (PMC12364318; doi:10.1371/journal.pone.0329090)
Supplement: S9 Table — (DOCX) [file pone.0329090.s009.docx]

| **Supplementary Table 9:** Global 2035 Projected Prevalence Analysis of Polycystic Ovary Syndrome in Women of Reproductive Age. | | | | |
| --- | --- | --- | --- | --- |
| Value | Time | Group | Low | Up |
| 1925.117406 | 1990 | 15-19 | 1913.806217 | 1936.428596 |
| 1944.050249 | 1991 | 15-19 | 1932.764253 | 1955.336245 |
| 1964.753419 | 1992 | 15-19 | 1953.49466 | 1976.012179 |
| 1985.243562 | 1993 | 15-19 | 1974.011704 | 1996.475421 |
| 2004.148278 | 1994 | 15-19 | 1992.941487 | 2015.355068 |
| 2021.087297 | 1995 | 15-19 | 2009.903465 | 2032.271129 |
| 2040.295617 | 1996 | 15-19 | 2029.137866 | 2051.453368 |
| 2063.956826 | 1997 | 15-19 | 2052.83075 | 2075.082903 |
| 2087.62949 | 1998 | 15-19 | 2076.535057 | 2098.723922 |
| 2106.952251 | 1999 | 15-19 | 2095.884034 | 2118.020468 |
| 2118.742236 | 2000 | 15-19 | 2107.691072 | 2129.7934 |
| 2127.600738 | 2001 | 15-19 | 2116.563221 | 2138.638255 |
| 2139.771042 | 2002 | 15-19 | 2128.751046 | 2150.791038 |
| 2154.835846 | 2003 | 15-19 | 2143.836399 | 2165.835293 |
| 2172.112989 | 2004 | 15-19 | 2161.136037 | 2183.089941 |
| 2189.206798 | 2005 | 15-19 | 2178.251279 | 2200.162317 |
| 2204.430277 | 2006 | 15-19 | 2193.493015 | 2215.367539 |
| 2218.211939 | 2007 | 15-19 | 2207.290514 | 2229.133364 |
| 2231.829585 | 2008 | 15-19 | 2220.923286 | 2242.735885 |
| 2246.821551 | 2009 | 15-19 | 2235.931628 | 2257.711475 |
| 2264.941832 | 2010 | 15-19 | 2254.071623 | 2275.812041 |
| 2286.145923 | 2011 | 15-19 | 2275.298944 | 2296.992902 |
| 2309.015745 | 2012 | 15-19 | 2298.193923 | 2319.837566 |
| 2331.871722 | 2013 | 15-19 | 2321.07496 | 2342.668485 |
| 2353.556348 | 2014 | 15-19 | 2342.783228 | 2364.329468 |
| 2372.789019 | 2015 | 15-19 | 2362.036841 | 2383.541198 |
| 2395.064937 | 2016 | 15-19 | 2384.336897 | 2405.792978 |
| 2423.348975 | 2017 | 15-19 | 2412.651353 | 2434.046597 |
| 2452.407039 | 2018 | 15-19 | 2441.740557 | 2463.073521 |
| 2477.375266 | 2019 | 15-19 | 2466.735667 | 2488.014865 |
| 2514.932879 | 2020 | 15-19 | 2504.332871 | 2525.532887 |
| 2523.769638 | 2021 | 15-19 | 2513.180221 | 2534.359055 |
| 2548.888863 | 2022 | 15-19 | 2477.117862 | 2620.659865 |
| 2570.343259 | 2023 | 15-19 | 2473.292118 | 2667.394399 |
| 2592.106678 | 2024 | 15-19 | 2463.635593 | 2720.577764 |
| 2614.218019 | 2025 | 15-19 | 2449.407358 | 2779.028681 |
| 2636.717397 | 2026 | 15-19 | 2431.254818 | 2842.179976 |
| 2659.646198 | 2027 | 15-19 | 2409.494113 | 2909.798282 |
| 2683.047154 | 2028 | 15-19 | 2384.298715 | 2981.795593 |
| 2706.964422 | 2029 | 15-19 | 2355.778827 | 3058.150016 |
| 2731.44366 | 2030 | 15-19 | 2324.009371 | 3138.877949 |
| 2756.532119 | 2031 | 15-19 | 2289.039499 | 3224.02474 |
| 2782.278727 | 2032 | 15-19 | 2250.896164 | 3313.661291 |
| 2808.734194 | 2033 | 15-19 | 2209.585744 | 3407.882643 |
| 2835.951116 | 2034 | 15-19 | 2165.094893 | 3506.807339 |
| 2863.9841 | 2035 | 15-19 | 2117.39099 | 3610.577211 |
| 2600.468405 | 1990 | 20-24 | 2589.918253 | 2611.018557 |
| 2638.107126 | 1991 | 20-24 | 2627.59438 | 2648.619872 |
| 2672.05286 | 1992 | 20-24 | 2661.57326 | 2682.532459 |
| 2704.858235 | 1993 | 20-24 | 2694.410069 | 2715.306401 |
| 2733.525087 | 1994 | 20-24 | 2723.103875 | 2743.9463 |
| 2756.17868 | 1995 | 20-24 | 2745.778468 | 2766.578891 |
| 2780.701138 | 1996 | 20-24 | 2770.323065 | 2791.07921 |
| 2814.131104 | 1997 | 20-24 | 2803.782635 | 2824.479574 |
| 2850.013634 | 1998 | 20-24 | 2839.696818 | 2860.33045 |
| 2882.855127 | 1999 | 20-24 | 2872.567678 | 2893.142576 |
| 2909.186567 | 2000 | 20-24 | 2898.923466 | 2919.449668 |
| 2933.545996 | 2001 | 20-24 | 2923.305911 | 2943.786081 |
| 2959.967252 | 2002 | 20-24 | 2949.752187 | 2970.182317 |
| 2983.549564 | 2003 | 20-24 | 2973.357514 | 2993.741614 |
| 3000.638111 | 2004 | 20-24 | 2990.46409 | 3010.812131 |
| 3009.143681 | 2005 | 20-24 | 2998.981032 | 3019.306331 |
| 3011.974918 | 2006 | 20-24 | 3001.81938 | 3022.130455 |
| 3015.421091 | 2007 | 20-24 | 3005.273003 | 3025.56918 |
| 3024.20888 | 2008 | 20-24 | 3014.072008 | 3034.345751 |
| 3041.018686 | 2009 | 20-24 | 3030.898646 | 3051.138725 |
| 3064.485317 | 2010 | 20-24 | 3054.386158 | 3074.584476 |
| 3096.135049 | 2011 | 20-24 | 3086.061705 | 3106.208393 |
| 3135.217861 | 2012 | 20-24 | 3125.174883 | 3145.260839 |
| 3176.492764 | 2013 | 20-24 | 3166.480702 | 3186.504827 |
| 3214.584342 | 2014 | 20-24 | 3204.599873 | 3224.568812 |
| 3245.486758 | 2015 | 20-24 | 3235.524009 | 3255.449508 |
| 3275.754229 | 2016 | 20-24 | 3265.812971 | 3285.695488 |
| 3312.529857 | 2017 | 20-24 | 3302.615178 | 3322.444537 |
| 3350.820477 | 2018 | 20-24 | 3340.93347 | 3360.707484 |
| 3386.630038 | 2019 | 20-24 | 3376.768828 | 3396.491248 |
| 3436.608153 | 2020 | 20-24 | 3426.7827 | 3446.433606 |
| 3453.970832 | 2021 | 20-24 | 3444.158 | 3463.783664 |
| 3486.668706 | 2022 | 20-24 | 3412.684815 | 3560.652596 |
| 3517.209998 | 2023 | 20-24 | 3434.360788 | 3600.059209 |
| 3547.875038 | 2024 | 20-24 | 3456.770446 | 3638.97963 |
| 3578.539112 | 2025 | 20-24 | 3478.861389 | 3678.216835 |
| 3608.455675 | 2026 | 20-24 | 3497.174865 | 3719.736484 |
| 3638.708234 | 2027 | 20-24 | 3507.765271 | 3769.651197 |
| 3669.348456 | 2028 | 20-24 | 3508.328966 | 3830.367946 |
| 3700.429975 | 2029 | 20-24 | 3499.50428 | 3901.35567 |
| 3732.008293 | 2030 | 20-24 | 3482.782982 | 3981.233603 |
| 3764.140698 | 2031 | 20-24 | 3459.353779 | 4068.927618 |
| 3796.886306 | 2032 | 20-24 | 3430.009032 | 4163.763581 |
| 3830.30615 | 2033 | 20-24 | 3395.256338 | 4265.355962 |
| 3864.463284 | 2034 | 20-24 | 3355.424083 | 4373.502484 |
| 3899.422906 | 2035 | 20-24 | 3310.727847 | 4488.117964 |
| 2740.287305 | 1990 | 25-29 | 2729.845066 | 2750.729545 |
| 2745.160064 | 1991 | 25-29 | 2734.728704 | 2755.591424 |
| 2762.699511 | 1992 | 25-29 | 2752.288937 | 2773.110085 |
| 2785.763108 | 1993 | 25-29 | 2775.376399 | 2796.149817 |
| 2813.03461 | 1994 | 25-29 | 2802.674204 | 2823.395017 |
| 2840.841598 | 1995 | 25-29 | 2830.507247 | 2851.175949 |
| 2885.272774 | 1996 | 25-29 | 2874.978443 | 2895.567105 |
| 2931.350949 | 1997 | 25-29 | 2921.097226 | 2941.604673 |
| 2978.007724 | 1998 | 25-29 | 2967.7943 | 2988.221148 |
| 3017.103051 | 1999 | 25-29 | 3006.922876 | 3027.283226 |
| 3043.40164 | 2000 | 25-29 | 3033.243589 | 3053.559691 |
| 3062.816562 | 2001 | 25-29 | 3052.674393 | 3072.95873 |
| 3085.653722 | 2002 | 25-29 | 3075.529766 | 3095.777678 |
| 3109.071909 | 2003 | 25-29 | 3098.966769 | 3119.17705 |
| 3130.81944 | 2004 | 25-29 | 3120.732495 | 3140.906386 |
| 3148.637017 | 2005 | 25-29 | 3138.56609 | 3158.707945 |
| 3161.219058 | 2006 | 25-29 | 3151.160683 | 3171.277433 |
| 3170.490237 | 2007 | 25-29 | 3160.442369 | 3180.538104 |
| 3179.042807 | 2008 | 25-29 | 3169.0055 | 3189.080114 |
| 3188.423777 | 2009 | 25-29 | 3178.398146 | 3198.449408 |
| 3200.055782 | 2010 | 25-29 | 3190.043809 | 3210.067756 |
| 3216.450972 | 2011 | 25-29 | 3206.456587 | 3226.445358 |
| 3238.172132 | 2012 | 25-29 | 3228.199144 | 3248.145119 |
| 3264.707932 | 2013 | 25-29 | 3254.759079 | 3274.656785 |
| 3294.468928 | 2014 | 25-29 | 3284.545285 | 3304.392571 |
| 3324.127453 | 2015 | 25-29 | 3314.22735 | 3334.027555 |
| 3360.404325 | 2016 | 25-29 | 3350.530785 | 3370.277866 |
| 3407.828279 | 2017 | 25-29 | 3397.987796 | 3417.668763 |
| 3458.494183 | 2018 | 25-29 | 3448.687753 | 3468.300614 |
| 3504.6039 | 2019 | 25-29 | 3494.827439 | 3514.380362 |
| 3558.866126 | 2020 | 25-29 | 3549.124699 | 3568.607554 |
| 3581.87294 | 2021 | 25-29 | 3572.145725 | 3591.600154 |
| 3626.055753 | 2022 | 25-29 | 3550.726499 | 3701.385006 |
| 3659.060387 | 2023 | 25-29 | 3574.631023 | 3743.489751 |
| 3691.604991 | 2024 | 25-29 | 3598.426439 | 3784.783542 |
| 3724.116815 | 2025 | 25-29 | 3622.334383 | 3825.899248 |
| 3756.351929 | 2026 | 25-29 | 3645.799321 | 3866.904537 |
| 3789.164396 | 2027 | 25-29 | 3669.510441 | 3908.81835 |
| 3822.367176 | 2028 | 25-29 | 3693.977135 | 3950.757216 |
| 3855.704569 | 2029 | 25-29 | 3719.051793 | 3992.357345 |
| 3889.040974 | 2030 | 25-29 | 3743.922005 | 4034.159942 |
| 3921.565089 | 2031 | 25-29 | 3765.558184 | 4077.571994 |
| 3954.454649 | 2032 | 25-29 | 3780.475264 | 4128.434035 |
| 3987.765757 | 2033 | 25-29 | 3785.462884 | 4190.068629 |
| 4021.556604 | 2034 | 25-29 | 3780.027901 | 4263.085308 |
| 4055.8875 | 2035 | 25-29 | 3765.321872 | 4346.453128 |
| 2824.882162 | 1990 | 30-34 | 2814.477502 | 2835.286821 |
| 2861.157002 | 1991 | 30-34 | 2850.786475 | 2871.52753 |
| 2889.820591 | 1992 | 30-34 | 2879.479709 | 2900.161472 |
| 2905.926695 | 1993 | 30-34 | 2895.607508 | 2916.245882 |
| 2917.762809 | 1994 | 30-34 | 2907.462441 | 2928.063178 |
| 2928.510838 | 1995 | 30-34 | 2918.227739 | 2938.793938 |
| 2939.379071 | 1996 | 30-34 | 2929.111978 | 2949.646163 |
| 2971.736021 | 1997 | 30-34 | 2961.500994 | 2981.971049 |
| 3012.064063 | 1998 | 30-34 | 3001.865412 | 3022.262715 |
| 3053.307145 | 1999 | 30-34 | 3043.144077 | 3063.470212 |
| 3086.509374 | 2000 | 30-34 | 3076.374703 | 3096.644045 |
| 3123.598643 | 2001 | 30-34 | 3113.495209 | 3133.702077 |
| 3151.622748 | 2002 | 30-34 | 3141.543157 | 3161.702339 |
| 3176.380692 | 2003 | 30-34 | 3166.322089 | 3186.439295 |
| 3195.321601 | 2004 | 30-34 | 3185.279071 | 3205.364131 |
| 3207.502541 | 2005 | 30-34 | 3197.470375 | 3217.534707 |
| 3213.002872 | 2006 | 30-34 | 3202.975408 | 3223.030336 |
| 3217.229053 | 2007 | 30-34 | 3207.205049 | 3227.253056 |
| 3224.120051 | 2008 | 30-34 | 3214.101836 | 3234.138265 |
| 3236.660645 | 2009 | 30-34 | 3226.653302 | 3246.667987 |
| 3256.88848 | 2010 | 30-34 | 3246.898558 | 3266.878402 |
| 3285.130011 | 2011 | 30-34 | 3275.163789 | 3295.096232 |
| 3317.896729 | 2012 | 30-34 | 3307.95785 | 3327.835608 |
| 3350.034773 | 2013 | 30-34 | 3340.123155 | 3359.946391 |
| 3376.278704 | 2014 | 30-34 | 3366.390426 | 3386.166983 |
| 3394.007659 | 2015 | 30-34 | 3384.136999 | 3403.878319 |
| 3412.977007 | 2016 | 30-34 | 3403.125235 | 3422.828778 |
| 3441.498479 | 2017 | 30-34 | 3431.672014 | 3451.324944 |
| 3476.460548 | 2018 | 30-34 | 3466.662871 | 3486.258224 |
| 3514.073003 | 2019 | 30-34 | 3504.304483 | 3523.841523 |
| 3567.182691 | 2020 | 30-34 | 3557.452023 | 3576.913359 |
| 3600.514987 | 2021 | 30-34 | 3590.806671 | 3610.223302 |
| 3655.793837 | 2022 | 30-34 | 3580.457944 | 3731.12973 |
| 3699.666162 | 2023 | 30-34 | 3614.946886 | 3784.385438 |
| 3741.928228 | 2024 | 30-34 | 3648.096054 | 3835.760403 |
| 3781.758066 | 2025 | 30-34 | 3679.017178 | 3884.498954 |
| 3819.026876 | 2026 | 30-34 | 3707.373855 | 3930.679898 |
| 3855.138134 | 2027 | 30-34 | 3734.393438 | 3975.88283 |
| 3890.240274 | 2028 | 30-34 | 3760.673959 | 4019.80659 |
| 3924.853779 | 2029 | 30-34 | 3786.735006 | 4062.972552 |
| 3959.432835 | 2030 | 30-34 | 3812.84864 | 4106.017031 |
| 3993.718137 | 2031 | 30-34 | 3838.492823 | 4148.943451 |
| 4028.617782 | 2032 | 30-34 | 3864.3807 | 4192.854863 |
| 4063.932481 | 2033 | 30-34 | 3890.873977 | 4236.990986 |
| 4099.390156 | 2034 | 30-34 | 3917.840465 | 4280.939847 |
| 4134.846572 | 2035 | 30-34 | 3944.579014 | 4325.11413 |
| 2840.404473 | 1990 | 35-39 | 2829.987014 | 2850.821933 |
| 2855.680002 | 1991 | 35-39 | 2845.282201 | 2866.077803 |
| 2881.716119 | 1992 | 35-39 | 2871.345338 | 2892.086901 |
| 2921.784055 | 1993 | 35-39 | 2911.449524 | 2932.118585 |
| 2969.084505 | 1994 | 35-39 | 2958.790223 | 2979.378788 |
| 3013.597275 | 1995 | 35-39 | 3003.34042 | 3023.85413 |
| 3054.689166 | 1996 | 35-39 | 3044.468324 | 3064.910008 |
| 3092.622942 | 1997 | 35-39 | 3082.437816 | 3102.808069 |
| 3120.378692 | 1998 | 35-39 | 3110.22419 | 3130.533194 |
| 3142.820445 | 1999 | 35-39 | 3132.693253 | 3152.947636 |
| 3160.10705 | 2000 | 35-39 | 3150.002456 | 3170.211644 |
| 3166.248727 | 2001 | 35-39 | 3156.156739 | 3176.340714 |
| 3183.788596 | 2002 | 35-39 | 3173.716061 | 3193.86113 |
| 3203.54183 | 2003 | 35-39 | 3193.488331 | 3213.595329 |
| 3224.006787 | 2004 | 35-39 | 3213.971617 | 3234.041957 |
| 3240.452019 | 2005 | 35-39 | 3230.431621 | 3250.472417 |
| 3260.7982 | 2006 | 35-39 | 3250.795492 | 3270.800907 |
| 3267.348682 | 2007 | 35-39 | 3257.353364 | 3277.344 |
| 3273.434726 | 2008 | 35-39 | 3263.446235 | 3283.423217 |
| 3282.373376 | 2009 | 35-39 | 3272.393348 | 3292.353404 |
| 3298.349866 | 2010 | 35-39 | 3288.38271 | 3308.317022 |
| 3322.573917 | 2011 | 35-39 | 3312.624733 | 3332.523101 |
| 3352.744779 | 2012 | 35-39 | 3342.817359 | 3362.672198 |
| 3385.399068 | 2013 | 35-39 | 3375.495254 | 3395.302882 |
| 3417.420304 | 2014 | 35-39 | 3407.54031 | 3427.300297 |
| 3445.852066 | 2015 | 35-39 | 3435.994289 | 3455.709844 |
| 3477.796693 | 2016 | 35-39 | 3467.963991 | 3487.629395 |
| 3517.868085 | 2017 | 35-39 | 3508.066296 | 3527.669873 |
| 3558.044624 | 2018 | 35-39 | 3548.274155 | 3567.815093 |
| 3590.813828 | 2019 | 35-39 | 3581.070045 | 3600.557611 |
| 3631.649996 | 2020 | 35-39 | 3621.938436 | 3641.361557 |
| 3644.977628 | 2021 | 35-39 | 3635.280668 | 3654.674588 |
| 3674.004887 | 2022 | 35-39 | 3598.62685 | 3749.382923 |
| 3703.319493 | 2023 | 35-39 | 3618.875167 | 3787.763819 |
| 3737.897343 | 2024 | 35-39 | 3644.513708 | 3831.280979 |
| 3777.365378 | 2025 | 35-39 | 3675.06769 | 3879.663067 |
| 3820.662589 | 2026 | 35-39 | 3709.284072 | 3932.041106 |
| 3866.842023 | 2027 | 35-39 | 3746.080734 | 3987.603312 |
| 3913.259859 | 2028 | 35-39 | 3783.283141 | 4043.236578 |
| 3957.974867 | 2029 | 35-39 | 3819.044589 | 4096.905145 |
| 4000.11756 | 2030 | 35-39 | 3852.421201 | 4147.81392 |
| 4039.551779 | 2031 | 35-39 | 3883.07822 | 4196.025338 |
| 4077.762189 | 2032 | 35-39 | 3912.283608 | 4243.24077 |
| 4114.90541 | 2033 | 35-39 | 3940.548771 | 4289.26205 |
| 4151.531854 | 2034 | 35-39 | 3968.444832 | 4334.618876 |
| 4188.121974 | 2035 | 35-39 | 3996.307793 | 4379.936154 |
| 3041.466567 | 1990 | 40-44 | 3031.131868 | 3051.801267 |
| 3051.932393 | 1991 | 40-44 | 3041.624232 | 3062.240554 |
| 3031.034758 | 1992 | 40-44 | 3020.724164 | 3041.345352 |
| 3003.166488 | 1993 | 40-44 | 2992.848082 | 3013.484893 |
| 2989.832983 | 1994 | 40-44 | 2979.514156 | 3000.15181 |
| 2982.03561 | 1995 | 40-44 | 2971.723127 | 2992.348092 |
| 3007.605125 | 1996 | 40-44 | 2997.319316 | 3017.890934 |
| 3048.569093 | 1997 | 40-44 | 3038.320192 | 3058.817995 |
| 3103.337534 | 1998 | 40-44 | 3093.133534 | 3113.541533 |
| 3159.589708 | 1999 | 40-44 | 3149.42975 | 3169.749666 |
| 3203.106256 | 2000 | 40-44 | 3192.980699 | 3213.231814 |
| 3232.569883 | 2001 | 40-44 | 3222.470418 | 3242.669347 |
| 3251.933722 | 2002 | 40-44 | 3241.855871 | 3262.011574 |
| 3259.54065 | 2003 | 40-44 | 3249.479098 | 3269.602203 |
| 3263.468021 | 2004 | 40-44 | 3253.420923 | 3273.515118 |
| 3268.319554 | 2005 | 40-44 | 3258.286614 | 3278.352493 |
| 3259.79671 | 2006 | 40-44 | 3249.766483 | 3269.826938 |
| 3257.716588 | 2007 | 40-44 | 3247.691887 | 3267.741289 |
| 3259.356416 | 2008 | 40-44 | 3249.337751 | 3269.37508 |
| 3269.442287 | 2009 | 40-44 | 3259.434502 | 3279.450072 |
| 3287.884817 | 2010 | 40-44 | 3277.893284 | 3297.876349 |
| 3325.771814 | 2011 | 40-44 | 3315.810407 | 3335.73322 |
| 3361.18299 | 2012 | 40-44 | 3351.249409 | 3371.11657 |
| 3397.90846 | 2013 | 40-44 | 3388.003081 | 3407.813839 |
| 3430.69185 | 2014 | 40-44 | 3420.811167 | 3440.572533 |
| 3456.61212 | 2015 | 40-44 | 3446.75053 | 3466.47371 |
| 3485.780537 | 2016 | 40-44 | 3475.939294 | 3495.621779 |
| 3526.28614 | 2017 | 40-44 | 3516.472292 | 3536.099989 |
| 3570.055075 | 2018 | 40-44 | 3560.270748 | 3579.839402 |
| 3610.272481 | 2019 | 40-44 | 3600.516036 | 3620.028926 |
| 3662.292901 | 2020 | 40-44 | 3652.572712 | 3672.013089 |
| 3687.016699 | 2021 | 40-44 | 3677.315782 | 3696.717616 |
| 3710.461348 | 2022 | 40-44 | 3634.589588 | 3786.333108 |
| 3740.133886 | 2023 | 40-44 | 3655.1479 | 3825.119871 |
| 3766.79759 | 2024 | 40-44 | 3672.998236 | 3860.596945 |
| 3790.59196 | 2025 | 40-44 | 3688.208056 | 3892.975864 |
| 3813.549793 | 2026 | 40-44 | 3702.594152 | 3924.505435 |
| 3839.352297 | 2027 | 40-44 | 3719.629518 | 3959.075077 |
| 3869.998928 | 2028 | 40-44 | 3741.610611 | 3998.387246 |
| 3906.146018 | 2029 | 40-44 | 3769.153777 | 4043.13826 |
| 3947.403728 | 2030 | 40-44 | 3801.742059 | 4093.065398 |
| 3992.663426 | 2031 | 40-44 | 3838.088091 | 4147.238762 |
| 4040.935698 | 2032 | 40-44 | 3877.063565 | 4204.807832 |
| 4089.457354 | 2033 | 40-44 | 3916.312497 | 4262.602211 |
| 4136.199644 | 2034 | 40-44 | 3953.937218 | 4318.462069 |
| 4180.253954 | 2035 | 40-44 | 3988.995305 | 4371.512603 |
| 2587.916107 | 1990 | 45-49 | 2577.138721 | 2598.693494 |
| 2601.23277 | 1991 | 45-49 | 2590.473021 | 2611.99252 |
| 2649.820874 | 1992 | 45-49 | 2639.115748 | 2660.526 |
| 2692.793896 | 1993 | 45-49 | 2682.138578 | 2703.449214 |
| 2712.801044 | 1994 | 45-49 | 2702.181065 | 2723.421023 |
| 2744.63533 | 1995 | 45-49 | 2734.055952 | 2755.214707 |
| 2763.401277 | 1996 | 45-49 | 2752.854267 | 2773.948287 |
| 2761.790768 | 1997 | 45-49 | 2751.253916 | 2772.32762 |
| 2756.288695 | 1998 | 45-49 | 2745.759182 | 2766.818208 |
| 2758.251469 | 1999 | 45-49 | 2747.732461 | 2768.770478 |
| 2756.98049 | 2000 | 45-49 | 2746.472082 | 2767.488898 |
| 2769.129814 | 2001 | 45-49 | 2758.638549 | 2779.62108 |
| 2782.457917 | 2002 | 45-49 | 2771.983779 | 2792.932055 |
| 2800.852923 | 2003 | 45-49 | 2790.398491 | 2811.307354 |
| 2820.854861 | 2004 | 45-49 | 2810.420375 | 2831.289348 |
| 2836.57182 | 2005 | 45-49 | 2826.153685 | 2846.989955 |
| 2841.355346 | 2006 | 45-49 | 2830.946151 | 2851.764541 |
| 2834.54994 | 2007 | 45-49 | 2824.142436 | 2844.957444 |
| 2822.223376 | 2008 | 45-49 | 2811.815383 | 2832.631369 |
| 2816.021043 | 2009 | 45-49 | 2805.618172 | 2826.423914 |
| 2825.339408 | 2010 | 45-49 | 2814.953895 | 2835.724922 |
| 2839.253623 | 2011 | 45-49 | 2828.887663 | 2849.619583 |
| 2866.121727 | 2012 | 45-49 | 2855.784749 | 2876.458706 |
| 2896.146132 | 2013 | 45-49 | 2885.838682 | 2906.453582 |
| 2926.66792 | 2014 | 45-49 | 2916.389003 | 2936.946836 |
| 2951.723859 | 2015 | 45-49 | 2941.468206 | 2961.979512 |
| 2992.635101 | 2016 | 45-49 | 2982.415466 | 3002.854736 |
| 3040.964947 | 2017 | 45-49 | 3030.78642 | 3051.143473 |
| 3091.284877 | 2018 | 45-49 | 3081.148106 | 3101.421648 |
| 3131.087174 | 2019 | 45-49 | 3120.98302 | 3141.191327 |
| 3163.145161 | 2020 | 45-49 | 3153.06673 | 3173.223592 |
| 3182.46624 | 2021 | 45-49 | 3172.402854 | 3192.529627 |
| 3191.570727 | 2022 | 45-49 | 3125.746333 | 3257.39512 |
| 3218.36763 | 2023 | 45-49 | 3144.515453 | 3292.219808 |
| 3246.327928 | 2024 | 45-49 | 3164.513917 | 3328.141938 |
| 3274.732726 | 2025 | 45-49 | 3185.034003 | 3364.43145 |
| 3302.988898 | 2026 | 45-49 | 3205.423031 | 3400.554764 |
| 3330.949645 | 2027 | 45-49 | 3225.49744 | 3436.401849 |
| 3357.598237 | 2028 | 45-49 | 3244.527243 | 3470.669231 |
| 3381.545996 | 2029 | 45-49 | 3261.134967 | 3501.957025 |
| 3402.918081 | 2030 | 45-49 | 3275.351966 | 3530.484195 |
| 3423.539439 | 2031 | 45-49 | 3288.826669 | 3558.252208 |
| 3446.714946 | 2032 | 45-49 | 3304.678462 | 3588.75143 |
| 3474.239302 | 2033 | 45-49 | 3324.825897 | 3623.652707 |
| 3506.701674 | 2034 | 45-49 | 3349.818211 | 3663.585138 |
| 3543.752178 | 2035 | 45-49 | 3379.2265 | 3708.277856 |
