# Supplementary material for: Global and Chinese epidemiologic study of polycystic ovary syndrome in women of childbearing age, 1990–2021, and projections to 2035: Based on the Global Burden of Disease 2021 study
Source: PLoS One. 2025 Aug 19;20(8):e0329090. doi: 10.1371/journal.pone.0329090 (PMC12364318; doi:10.1371/journal.pone.0329090)
Supplement: S10 Table — (DOCX) [file pone.0329090.s010.docx]

| **Supplementary Table 10:** Analysis of the Projected global overall age-standardized Prevalence of polycystic ovary syndrome in women of reproductive age in 2035. | | | | |
| --- | --- | --- | --- | --- |
| **Value** | **Time** | **Group** | **Low** | **Up** |
| 2627.921511 | 1990 | ASR | 2627.040951 | 2628.802071 |
| 2648.04679 | 1991 | ASR | 2647.172333 | 2648.921248 |
| 2670.222266 | 1992 | ASR | 2669.353215 | 2671.091316 |
| 2691.615963 | 1993 | ASR | 2690.75244 | 2692.479486 |
| 2712.344256 | 1994 | ASR | 2711.486223 | 2713.20229 |
| 2733.326365 | 1995 | ASR | 2732.473428 | 2734.179302 |
| 2759.767375 | 1996 | ASR | 2758.918308 | 2760.616441 |
| 2791.0046 | 1997 | ASR | 2790.157922 | 2791.851278 |
| 2823.777654 | 1998 | ASR | 2822.933282 | 2824.622026 |
| 2854.593531 | 1999 | ASR | 2853.751279 | 2855.435783 |
| 2877.314106 | 2000 | ASR | 2876.475587 | 2878.152625 |
| 2896.967523 | 2001 | ASR | 2896.132615 | 2897.802431 |
| 2917.136859 | 2002 | ASR | 2916.305531 | 2917.968187 |
| 2936.360732 | 2003 | ASR | 2935.533072 | 2937.188392 |
| 2953.630545 | 2004 | ASR | 2952.806668 | 2954.454423 |
| 2966.991739 | 2005 | ASR | 2966.17196 | 2967.811518 |
| 2975.013733 | 2006 | ASR | 2974.198607 | 2975.828858 |
| 2979.809679 | 2007 | ASR | 2978.999474 | 2980.619884 |
| 2985.491545 | 2008 | ASR | 2984.686033 | 2986.297056 |
| 2995.75472 | 2009 | ASR | 2994.953048 | 2996.556392 |
| 3012.804022 | 2010 | ASR | 3012.004728 | 3013.603317 |
| 3037.633091 | 2011 | ASR | 3036.834566 | 3038.431616 |
| 3067.357027 | 2012 | ASR | 3066.558186 | 3068.155868 |
| 3098.928061 | 2013 | ASR | 3098.128199 | 3099.727924 |
| 3128.903724 | 2014 | ASR | 3128.102727 | 3129.70472 |
| 3154.105322 | 2015 | ASR | 3153.303622 | 3154.907022 |
| 3183.563175 | 2016 | ASR | 3182.760152 | 3184.366197 |
| 3221.529447 | 2017 | ASR | 3220.723935 | 3222.33496 |
| 3261.955432 | 2018 | ASR | 3261.147127 | 3262.763737 |
| 3298.368287 | 2019 | ASR | 3297.557765 | 3299.178809 |
| 3344.391816 | 2020 | ASR | 3343.577969 | 3345.205664 |
| 3364.126281 | 2021 | ASR | 3363.312349 | 3364.940212 |
| 3396.187319 | 2022 | ASR | 3354.230589 | 3438.14405 |
| 3426.788828 | 2023 | ASR | 3372.815327 | 3480.762328 |
| 3457.609609 | 2024 | ASR | 3392.58091 | 3522.638308 |
| 3488.458958 | 2025 | ASR | 3412.439443 | 3564.478474 |
| 3519.196584 | 2026 | ASR | 3431.478715 | 3606.914453 |
| 3550.632063 | 2027 | ASR | 3449.811645 | 3651.452482 |
| 3582.533507 | 2028 | ASR | 3467.183377 | 3697.883636 |
| 3614.600301 | 2029 | ASR | 3483.054036 | 3746.146565 |
| 3646.724021 | 2030 | ASR | 3496.917542 | 3796.5305 |
| 3678.783366 | 2031 | ASR | 3508.148696 | 3849.418037 |
| 3711.532073 | 2032 | ASR | 3517.011956 | 3906.052191 |
| 3744.823362 | 2033 | ASR | 3523.415891 | 3966.230833 |
| 3778.508767 | 2034 | ASR | 3527.108831 | 4029.908702 |
| 3812.481135 | 2035 | ASR | 3527.731606 | 4097.230664 |
