# Supplementary figures and images for: Global and Chinese epidemiologic study of polycystic ovary syndrome in women of childbearing age, 1990–2021, and projections to 2035: Based on the Global Burden of Disease 2021 study
Source: PLoS One. 2025 Aug 19;20(8):e0329090. doi: 10.1371/journal.pone.0329090 (PMC12364318; doi:10.1371/journal.pone.0329090)

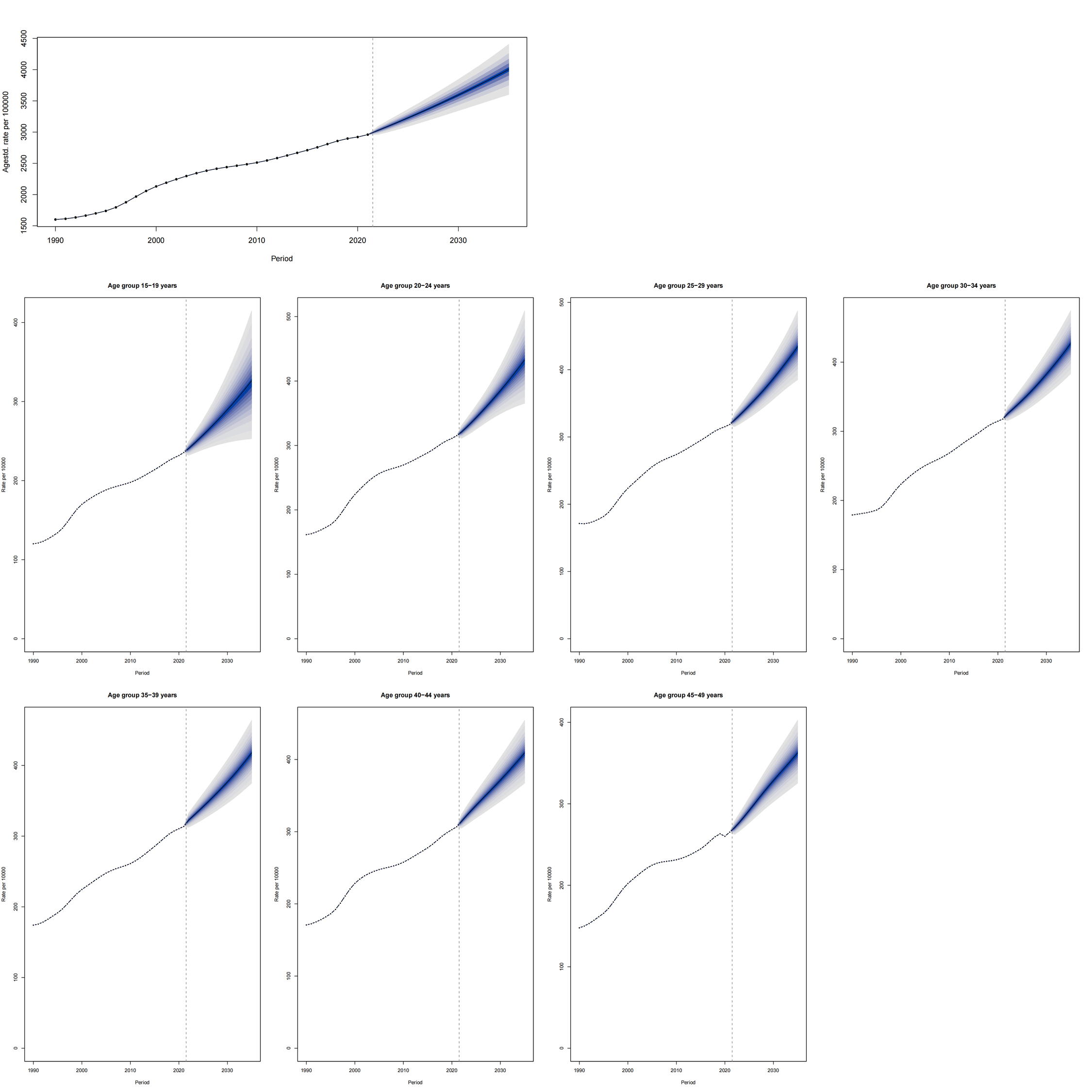

Supplement: S1 Fig — (TIF) [file pone.0329090.s011.tif]

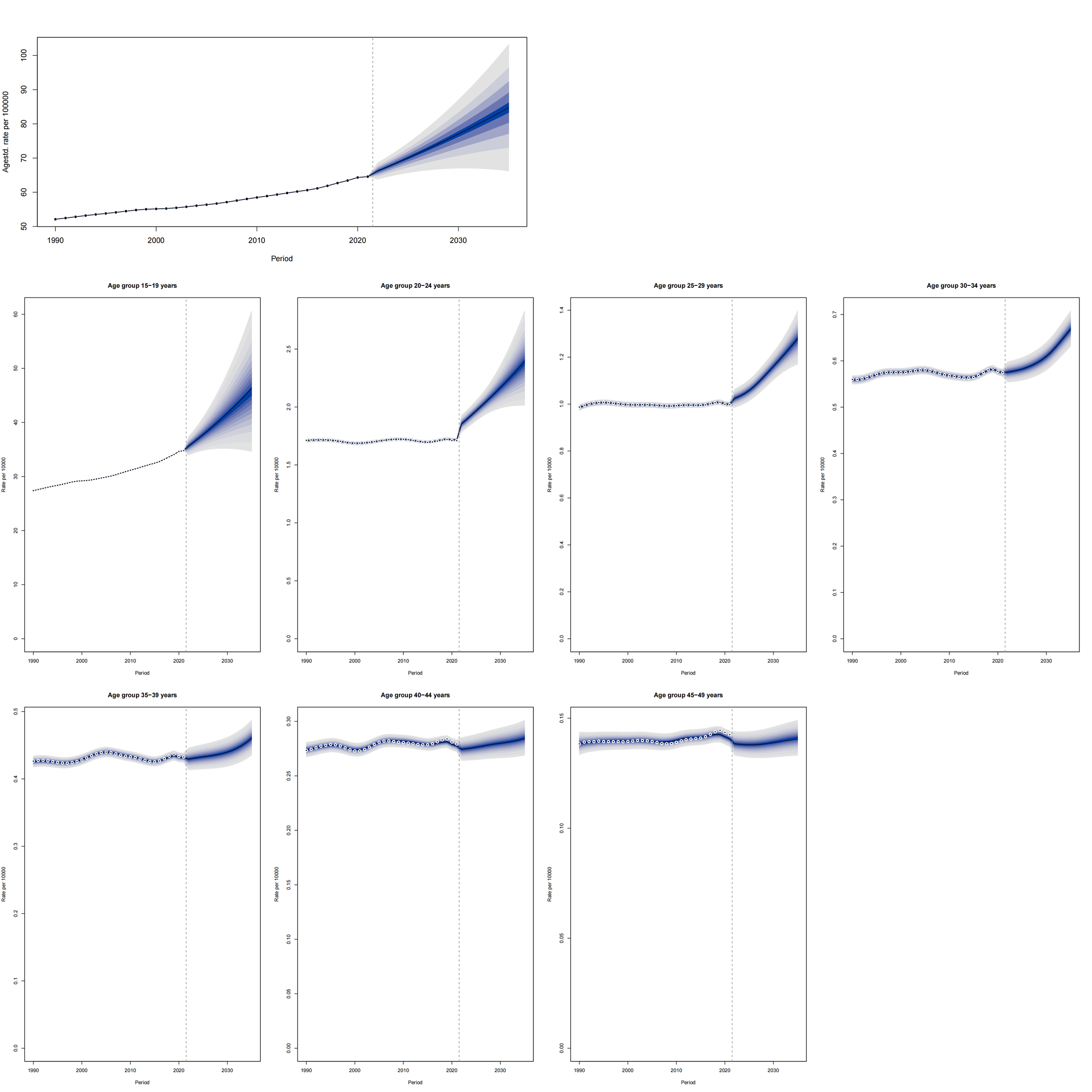

Supplement: S2 Fig — (TIF) [file pone.0329090.s012.tif]

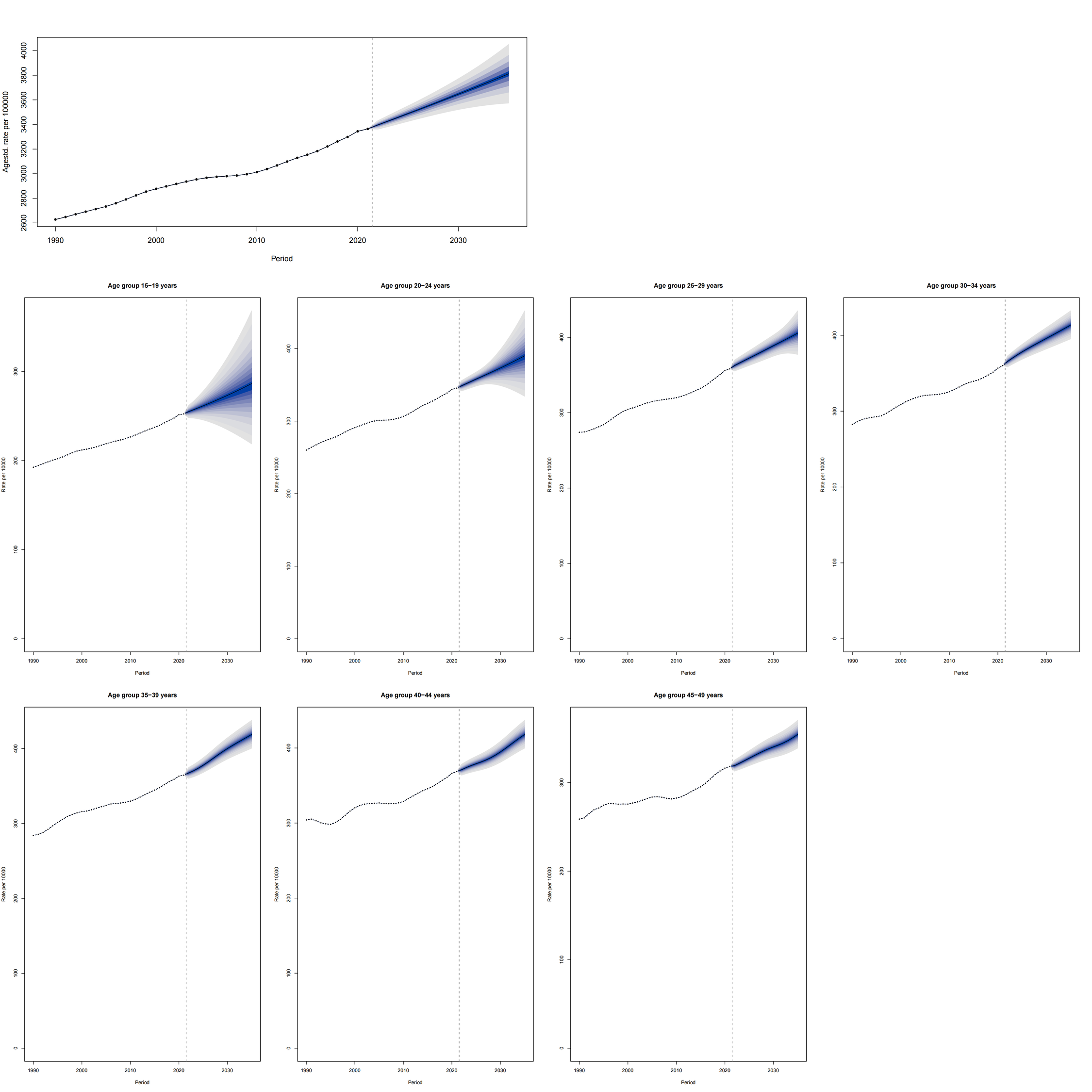

Supplement: S3 Fig — (TIF) [file pone.0329090.s013.tif]

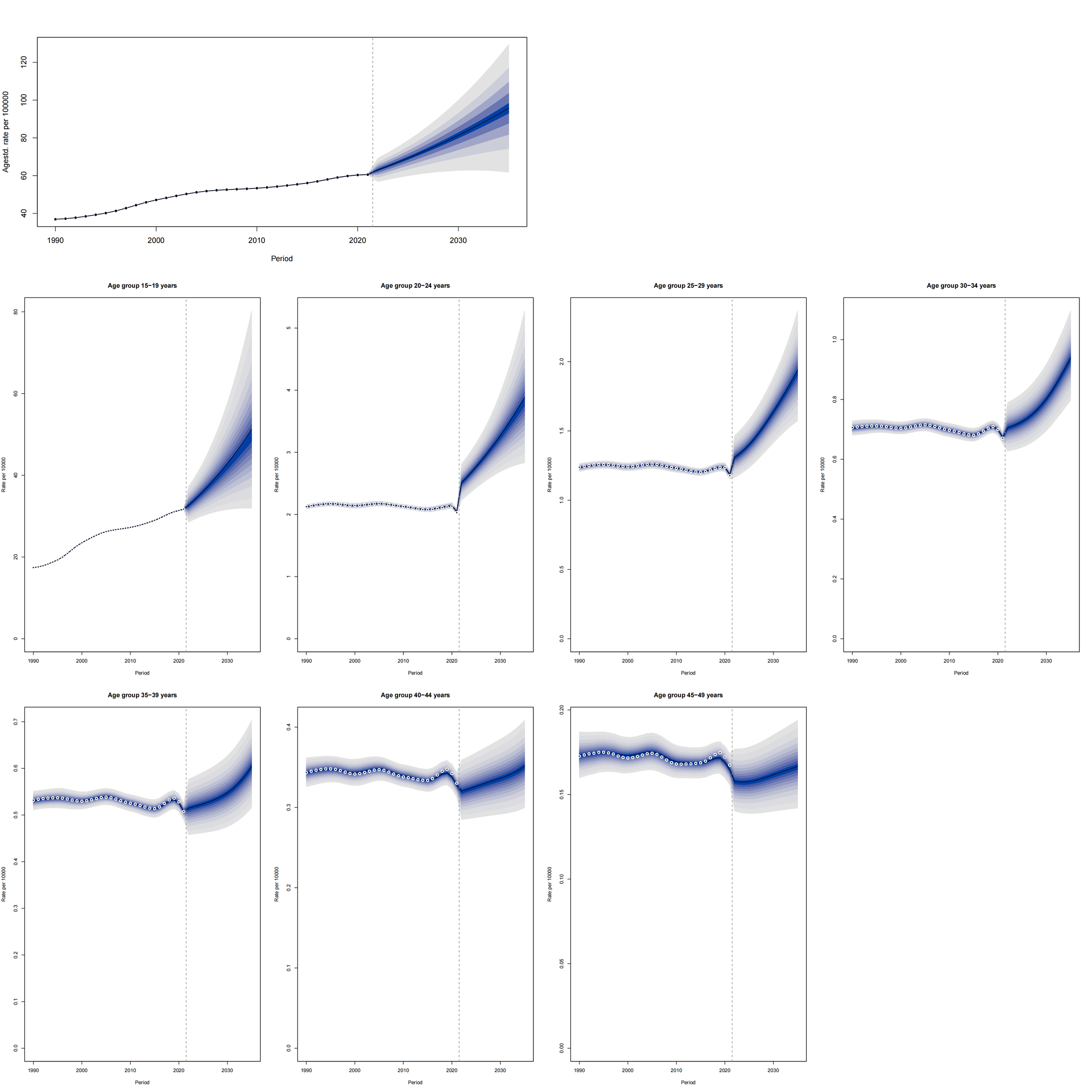

Supplement: S4 Fig — (TIF) [file pone.0329090.s014.tif]
