# Supplementary material for: Global and Chinese epidemiologic study of polycystic ovary syndrome in women of childbearing age, 1990–2021, and projections to 2035: Based on the Global Burden of Disease 2021 study
Source: PLoS One. 2025 Aug 19;20(8):e0329090. doi: 10.1371/journal.pone.0329090 (PMC12364318; doi:10.1371/journal.pone.0329090)
Supplement: Attachment — Submitted filename: Response to Reviewers.docx [file pone.0329090.s017.docx]

**Dear Reviewer:**

Greetings! Thank you very much for your valuable comments on our paper. We have carefully revised the relevant questions, and the following are the responses to your comments:

**For the first question:**

We have explored in detail the relationship between PCOS and environmental endocrine disruptors (EDCs) and other environmental pollution. Specifically, we added that although the incidence of PCOS in China has decreased, its prevalence and disease burden have increased significantly by 86.95% and 86.56%, respectively. We propose that this increase may be related to the rise in EDCs.

EDCs are widely present in various products and pollutants, such as plastics, pesticides, cosmetics, and industrial pollutants, and can adversely affect the endocrine system. For instance:

Exposure to bisphenol A (BPA) can cause hyperandrogenemia and ovarian polycystic changes. The underlying mechanism involves the abnormal activation of estrogen receptor β and the dysregulation of the insulin signaling pathway.

The concentration of phthalate metabolites in the urine of PCOS patients is significantly higher than that in healthy individuals and is positively correlated with insulin resistance and oxidative stress.

In a group of women with long-term exposure to industrial pollutants, such as polychlorinated biphenyls (PCBs), the occurrence of ovarian cysts may be associated with an increase in EDCs. Additionally, in a female cohort exposed to industrial pollutants like PCBs, the risk of ovarian cysts and the levels of inflammatory factors were significantly higher.

It should also be noted that during China's rapid industrialization, the continuous increase in EDCs may partially account for the rising trend in PCOS prevalence.

**For the second question:**

As you suggested, we have defined PCOS as "hyperandrogenism, oligoovulation, and polycystic ovarian morphology" and supplemented this definition with the 2023 international evidence-based guidelines.

Furthermore, we have refined the information on the impacts and risks of PCOS. PCOS is the leading cause of anovulatory infertility and is associated with an increased risk of cardiovascular disease, endometrial cancer, and significant psychosocial impairment. It is also accompanied by skin manifestations such as hirsutism, acne, and alopecia.

**Regarding the similarity check:**

We have checked the similarity rate of the manuscript. The result of the thesis check is 13%, and the file name of the check report is "similarity rate.pdf". We have uploaded the similarity check result.

Thank you again for your interest and support of our paper, and please feel free to advise us of any further comments and suggestions.

XiaoDan ZHANG
