# Supplementary material for: Global and Chinese epidemiologic study of polycystic ovary syndrome in women of childbearing age, 1990–2021, and projections to 2035: Based on the Global Burden of Disease 2021 study
Source: PLoS One. 2025 Aug 19;20(8):e0329090. doi: 10.1371/journal.pone.0329090 (PMC12364318; doi:10.1371/journal.pone.0329090)
Supplement: Attachment — Submitted filename: Response_to_Reviewers_auresp_2.docx [file pone.0329090.s018.docx]

**Dear Editors，**

Thank you for reviewing our manuscript. We have addressed all reviewers' comments in the revised manuscript.

Response to Journal Requirements Regarding References:

As requested by the journal, we have thoroughly reviewed the reference list in our manuscript to verify its completeness and correctness. We specifically checked for any potential retracted papers cited within our references and can confirm that no retracted articles are cited in our manuscript. Therefore, no changes were required to the reference list in accordance with this specific point.

All cited references remain appropriate and support the claims made in the text.”

**Dear Reviewer 2,**

Thank you very much for your additional feedback and for highlighting the confusion in the usage of prevalence and incidence in the manuscript. We sincerely appreciate your meticulous review and apologize for the oversight in addressing this point in our previous response. Your comment is highly valuable for improving the clarity and accuracy of our work.

We have carefully reviewed the entire manuscript to identify all instances where prevalence and incidence were interchanged. As you correctly pointed out (e.g., in line 200 of the original version), prevalence was used incorrectly in place of incidence. To address this:

①We have systematically replaced "prevalence" with "incidence" in the relevant sections, including line 200, to ensure consistency and proper terminology usage.

②This correction has been highlighted in the Revised Manuscript with Track Changes, marked in red text for easy reference (specifically, in the tracked changes version). We have ensured that the distinction between prevalence (as a measure of existing cases) and incidence (as a measure of new cases) is now clear throughout the text.

With these revisions, we believe the concern you raised has been fully resolved. Your suggestion has significantly enhanced the quality of our article, and we are grateful for your expertise. Thank you once again for your time, constructive criticism, and for considering our study as "very interesting." We hope that the manuscript now meets the standards for publication and look forward to your final evaluation.

Sincerely

XiaoDan ZHANG
